# Nickel-Catalyzed Enantioselective Coupling of Aldehydes and Electron-Deficient 1,3-Dienes Following an Inverse Regiochemical Course

**DOI:** 10.1021/jacs.2c09328

**Published:** 2022-10-04

**Authors:** Thomas Q. Davies, Jae Yeon Kim, Alois Fürstner

**Affiliations:** Max-Planck-Institut für Kohlenforschung, 45470 Mülheim/Ruhr, Germany

## Abstract

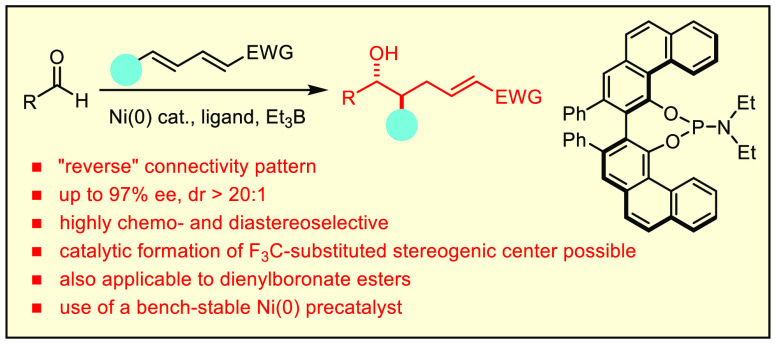

The nickel catalyzed reductive coupling of aldehydes with sorbate esters and related electron-deficient 1,3-dienes are known in the literature to occur at the π-bond proximal to the ester to afford aldol-type products. In stark contrast to this established path, a VAPOL-derived phosphoramidite ligand in combination with a bench-stable nickel precatalyst brokers a regiocomplementary course in that C–C bond formation proceeds exclusively at the distal alkene site to give deoxypropionate type products carrying an acrylate handle; they can be made in either *anti*- or *syn*-configured form. In addition to this enabling reverse pathway, the reaction is distinguished by excellent levels of chemo-, diastereo-, and enantioselectivity; moreover, it can be extended to the catalytic formation of F_3_C-substituted stereogenic centers. The use of a dienyl pinacolboronate instead of a sorbate ester is also possible, which opens access to valuable chiral borylated building blocks in optically active form.

The nickel catalyzed reductive coupling of aldehydes with 1,3-dienes mediated by BEt_3_ or ZnEt_2_, as pioneered by the groups of Mori and Tamaru in the early 1990s, is distinguished by a broad scope with regard to all reaction partners.^1−9^ Most notably, variously substituted dienes of largely different electronic character participate uniformly well and usually result in excellent levels of regio- and diastereoselectivity. This aspect is illustrated by the prototype examples compiled in Scheme 1A:^6^ isoprene reacts at the more highly substituted and hence more electron rich alkene site to give **1**, and methyl sorbate affords product **2** exclusively, in which the new C–C bond was formed α to the ester group in analogy to an aldol reaction.^6^ The exquisite *anti*-selectivity in both cases is another characteristic trait of reductive homoallylations of this type.^1−8^

**Scheme 1:**
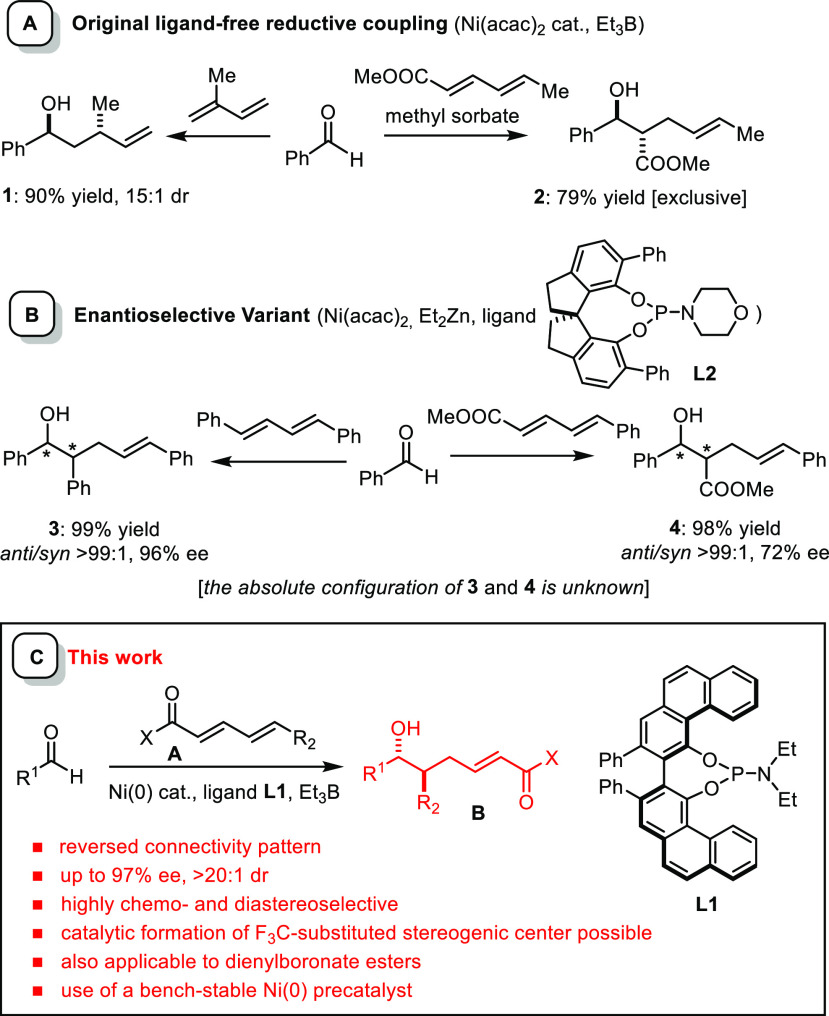
(A) Prototype Nickel-Catalyzed “Tamaru Reactions”, (B) Enantioselective Variant: State-of-the-Art (ref (13)), and (C) This Work

These chemical virtues, however, are partly offset by the difficulty of devising enantioselective versions of these reactions.^10,11^ Apart from a few special cases,^12^ 1,4-diphenylbuta-1,3-diene remains the only substrate known to date for which high levels of induction were reached in reactions with aromatic aldehydes using the spirocyclic phosphoramidite **L2** as ligand to the nickel catalyst (Scheme 1B);^13,14^ when applied to an electronically biased dienylester derivative, however, the resulting product **4** showed a much more modest ee.^13^ It is against this backdrop that the dramatic consequences of the use of the VAPOL-derived phosphoramidite **L1**(15,16) presented in this Communication have to be seen (Scheme 1C). Under its auspices, the nickel catalyzed reactions of sorbate esters or related substrates **A** follow an “inverse” regiochemical course: rather than affording aldol-type products such as **2** and **4**, it is the distal double bond that engages in C–C bond formation, leading to products of type **B**. This striking change of the connectivity pattern comes along with generally excellent levels of asymmetric induction.

The VAPOL-derived phosphoramidite **L1**, which is made in one step from commercial materials, had originally been developed during our study on the nickel catalyzed formation of predifferentiated diols from aldehydes and electron-rich dienol ethers or silylethers;^15^ it was found to be unique among a set of ≈50 chiral ligands in that it ensured excellent regiocontrol and respectable asymmetric induction, while affording meaningful chemical yields. To this end, however, catalyst loadings of 10 mol % and long reaction times at low temperature were mandatory in most cases.^15^ We were therefore pleased to find that reactivity is much less of an issue when electron deficient dienes such as methyl sorbate ((*E,E*)-**5**) are used as the substrates (Scheme 2). The reaction works well with the bench-stable Ni(0) stilbene complex Ni(*t*Bu-stb)_3_ as precatalyst,^17^ thus obviating the need to handle highly air-sensitive Ni(cod)_2_. With 2.5 mol % each of this convenient and commercial nickel source and the chiral ligand **L1** in combination with BEt_3_ as the promoter,^18^ (*E,E*)-**5** was coupled with benzaldehyde at ambient temperature to give the *anti*-configured alcohol **6a** in 90% yield and 94% ee, virtually as a single regio- and diastereomer (dr ≥ 20:1, rr ≥ 20:1). To the best of our knowledge, this course is unparalleled in the literature.^19^ The stereochemical assignment was based on the comparison of the spectral and chiroptical properties of the analogous ethyl ester derivative **6b** with the data of its literature-known antipode (see also below).^20^ The reaction also scales well (see below) and is therefore deemed enabling and practical alike.

**Scheme 2:**
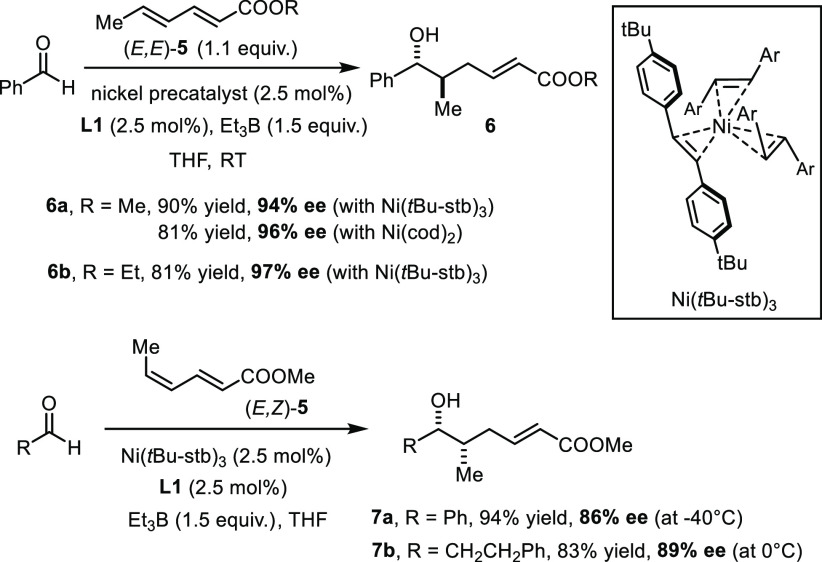
Regiospecific “Inverse” Coupling of Sorbate Esters with Aldehydes

As one might expect, the reductive coupling occurs stereospecifically: changing the geometry of the reacting distal double bond of methyl sorbate from *E* to *Z* switches of the product stereostructure from *anti* to *syn*, although the level of asymmetric induction in the resulting products **7** was slightly lower.

The chemoselectivity profile of this new transformation is excellent. Aryl aldehydes of largely different electronic character and steric demand were found to react well (Figure 1A): they range from compounds as electron-rich as 3,4,5-trimethoxybenzaldehyde or 4-dimethylaminobenzaldehyde to their electron-deficient cousins bearing a −COOMe, −CN, −Bpin, or −CF_3_ substituent on the *para*-position of the aromatic ring; all of them furnished the corresponding products with ee’s ≥ 90%. The compliance of *p*-trifluoromethylbenzaldehyde is particularly noteworthy, as it had been one of the least selective substrates in our previous study on the nickel catalyzed reductive diol synthesis.^15^ The successful use of 2-methylbenzaldehyde shows that an *ortho*-substituent does not bring the reaction to a halt, and furan-2-carbaldehyde was also well-accommodated. From the chemical point of view, it is remarkable that the aryl chloride and even aryl bromide groups in products **13** and **14** proved compatible, suggesting that this Ni(0)-based catalyst system is poor at undergoing oxidative addition; to rigorously scrutinize this aspect, the formation of **14** was repeated on 1 mmol scale without any serious detriment to yield and optical purity; 4-iodobenzaldehyde, however, remained beyond reach. The tolerance of the −CN and the −NMe_2_ groups, as manifested in the formation of **8b** and **8e**, respectively, is equally noteworthy since these functionalities are potential ligands to Ni(0) that could either bring the conversion to a halt and/or could compete with the chiral phosphoramidite and thus entail a racemic background reaction; neither problem was encountered. Limitations, however, are reached with pyridine-3-carbaldehyde, 3-nitrobenzaldehyde, and enals, which likely block or destroy the catalyst (for details, see the Supporting Information (SI)).

**Figure 1:**
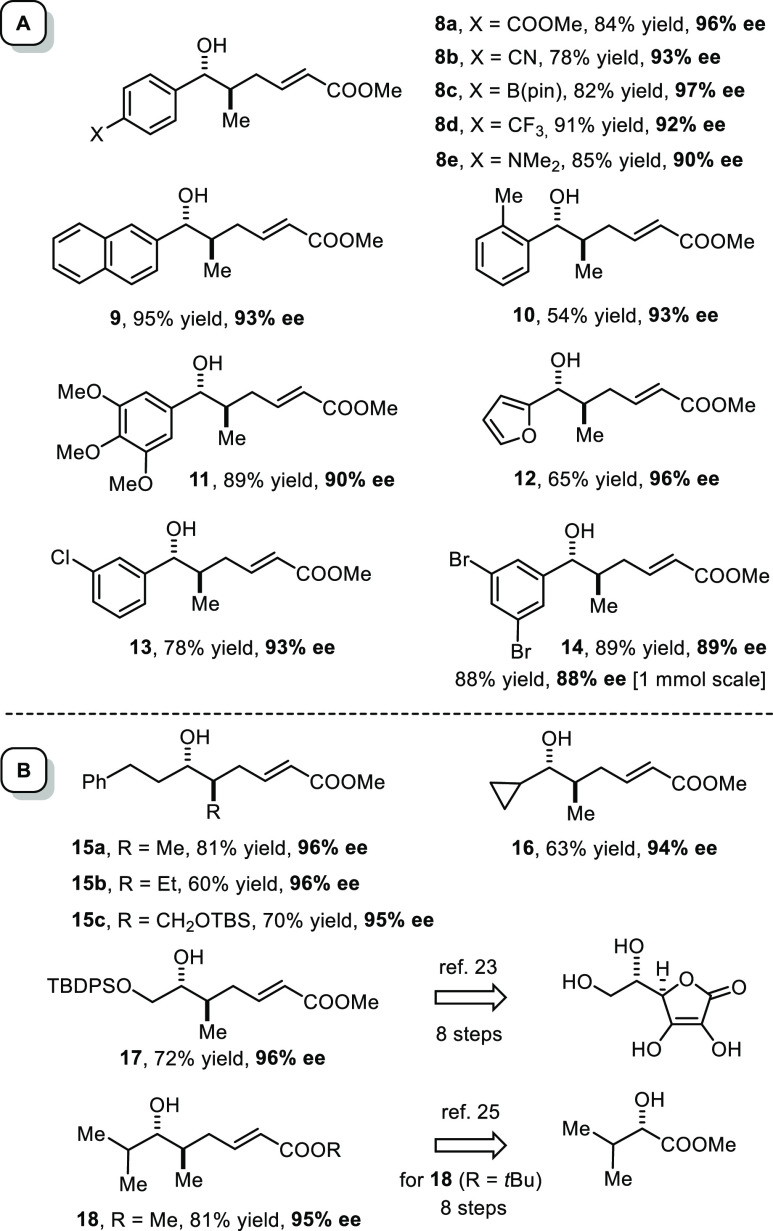
Scope of the nickel catalyzed enantioselective reverse coupling reaction of (*E,E*)-**5** and related α,β,γ,δ-diunsaturated ester derivatives under the conditions specified in Scheme 2; in all cases, the dr was >20:1

The standard reaction conditions also apply to aliphatic aldehydes, all of which afforded “inverse” adducts with ee’s well above 90% (Figure 1B). Products **15a**–**c** featuring a methyl, ethyl, or protected hydroxymethyl branch were formed with uniformly high selectivity; this finding suggests that there is ample scope with regard to the terminus on the reacting dienoate; a more systematic exploration of this aspect will follow.^21^ X-ray diffraction analysis of an osmate ester derived from **15b** allowed the absolute and relative configuration of this product to be unambiguously determined (see the SI).^22^ In the same context, we refer to compound **17**, which is literature-known and hence represents yet another independent reference point for structure assignment.^23^ Compound **17** has served in the past as a building block for the synthesis of the antibiotic (−)-cochleamycin A;^23,24^ it had been made starting from l-ascorbic acid in a linear sequence comprising no less than eight steps, whereas it is now available in a single operation starting from (*tert*-butyldiphenylsilyloxy)acetaldehyde. Equally facile is the preparation of product **18** (R = Me), again in one step from isobutyraldehyde. The analogous *tert*-butyl ester derivative (R = *t*Bu) is a valuable deoxypropionate synthon that had previously been accessed in eight steps starting from 2-hydroxy-3-methylbutyrate.^25,26^ These examples demonstrate the significance of such “inversely-connected” adducts, not least since their acrylate subunit provides a valuable handle for downstream functionalization. At the same time, the comparisons showcase the advance in step- and atom economy that the new nickel catalyzed procedure does enable.

The excellent functional group tolerance, which had already surfaced in the study of differently substituted aldehydes, suggested that the method should not be limited to sorbate esters either (Scheme 3). Particularly striking is the compatibility of the diunsaturated acid fluoride **19**, which reacted with benzaldehyde to give product **20** in good optical purity. The fact that the acyl fluoride group itself goes uncompromised is yet another illustration of the striking chemoselectivity of the active catalyst, which—in contrast to most other low-valent nickel species—is surprisingly resistant to oxidative insertion into polarized C–X bonds.^27^ In view of the rich chemistry of acyl fluorides in general, this result is arguably enabling.

**Scheme 3:**
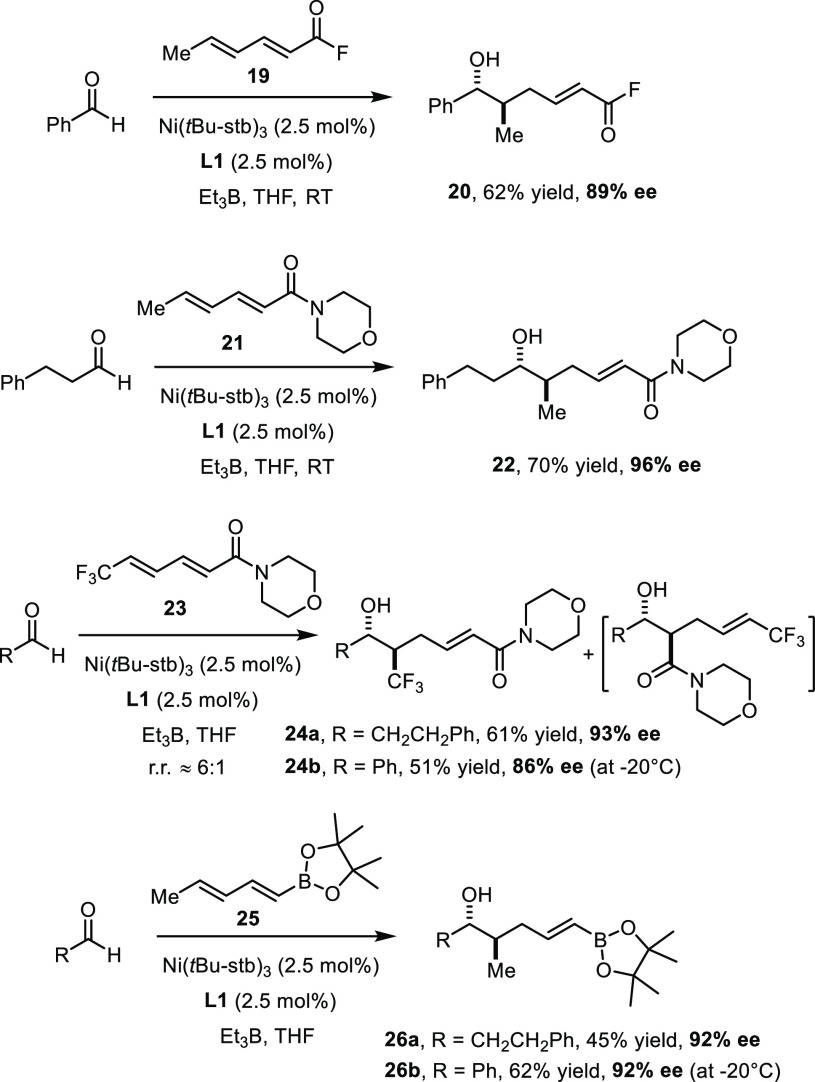
Variation of the Diene

The versatility of morpholine amides^28^ prompted us to test the corresponding sorbate derivative **21**. As expected, this substrate was fully compliant, providing product **22** with 96% ee.

Next, the analogous amide **23**(29) with a terminal trifluoromethyl group was made and coupled with hydrocinnamaldehyde. In contrast to essentially all examples described above,^21^ NMR inspection of the crude material showed that the reaction was not fully regioselective in this case (rr ≈ 6:1), probably because electron-withdrawing substituents are present on either end of the 1,3-diene subunit; this pattern seems to impact on the relative orientation of the reaction partners in the coordination sphere of the loaded catalyst. Gratifyingly, however, the resulting regioisomers are very easy to separate, such that the desired adduct **24a** was obtained in analytically pure form by ordinary flash chromatography in 61% yield with no less than 93% ee. Benzaldehyde gave a similar outcome, although the reaction had to be performed at −20 °C to reach a useful level of induction. From the conceptual point of view, the new method falls into the rare class of catalytic transformations that allow a −CF_3_ group to be introduced into a target compound with concomitant formation of a stereogenic center in optically active form.^30^ More specifically, it opens a currently unique catalytic entry into *anti*-configured β-trifluoromethyl alcohol derivatives in a single operation.^31^ Such compounds represent bioisosteres of natural products of polyketide origin and as such are highly valuable building blocks for the life sciences, which are difficult to make otherwise. Therefore, a more comprehensive investigation into the scope of this unprecedented reductive trifluoromethylation is warranted, which will be reported in due time.^32^

Finally, an even more profound change was made by formally replacing the ester (amide) group on the diene by a pinacolboronate entity. Once again, the preliminary results obtained with **25** are highly encouraging, not least because of the apparent versatility of alkenylboronate derivatives such as **26**.

Despite the unprecedented regioselective course, the new reaction is thought to pass through the same elementary steps as the literature-known nickel-catalyzed reductive couplings (Scheme 4).^6,8^ Thus, the formation of π-complex **A** by the coordination of the aldehyde and the diene to a monoligated [LNi^0^] species precedes oxidative cyclization to form a nickelacycle **B**; this critical (but potentially reversible)^8^ C–C bond formation benefits from the LUMO-lowering effect of Et_3_B bound to the carbonyl O atom.^33^ Ethyl transfer to the Ni(+2) center followed by β-hydride elimination affords an allylnickel hydride intermediate **C**, which evolves into the enoate upon reductive coupling. The dissociation of product **E** and ethylene from adduct **D** thus formed regenerates the catalyst. The placement of both the aldehyde substituent and the methyl terminus of the diene in pseudoequatorial orientation in the actual coupling step **A** to **B** explains the exquisite 1,2-*anti* selectivity. The absolute configuration of the resulting product can be rationalized by embedding the reactants into the deep chiral binding site of [**L1**Ni^0^] as drawn in **A**; secondary interactions likely assist in positioning the partners such that the “inverted” course of the reaction does ensue (see the SI). Considering the exceptional complexity and dynamic nature of the system and because of potential reversibility issues,^8^ extensive experimental and computational scrutiny will be necessary to prove or disprove the validity of this tentative stereochemical model.^34−36^

**Scheme 4:**
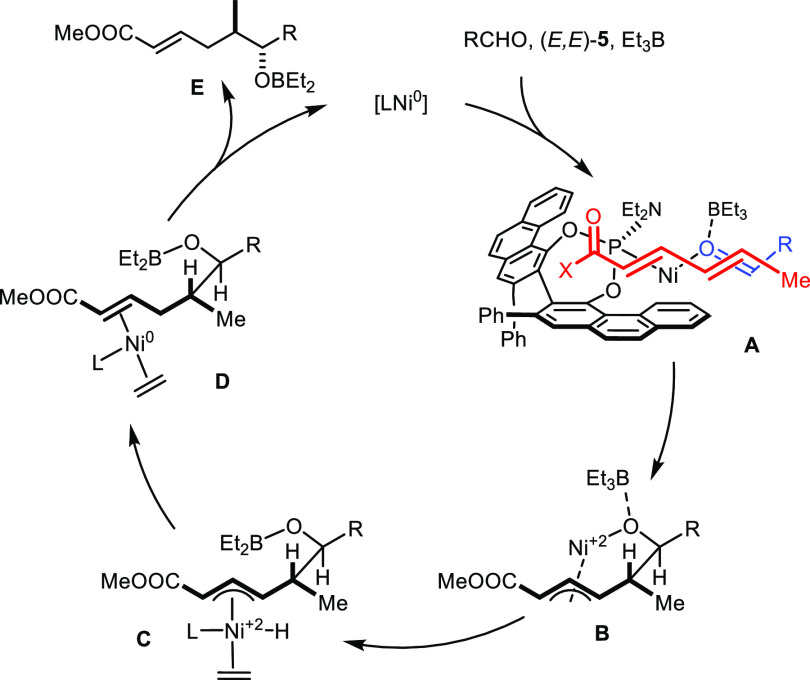
Proposed Mechanism

In summary, this study shows that the VAPOL-derived phosphoramidite **L1** is a true “game-changer” in the context of nickel catalyzed reductive coupling of dienes with aldehyde partners. This particular ligand imparts unique reactivity and selectivity onto the catalyst generated in situ, leading to a reaction course that is without precedent in the literature on related nickel catalyzed transformations. The new Ni(0)/**L1** system accommodates various substitution patterns in both partners and is able to broker reactions of dienes of a largely different character: they can be as electron-rich as dienyl silylethers used in the new diol synthesis previously reported by our group,^15^ or they can be electron-deficient such as the sorbate derivatives and dienylboronates described herein. In addition, the use of a bench-stable Ni(0) stilbene complex in lieu of Ni(cod)_2_ as precatalyst marks an important advance in practical terms. Further explorations of the scope, more profound studies into the mechanism, and applications of the reaction to target-oriented synthesis^37^ are subject to ongoing investigations in this laboratory.

## Supporting Information Available

Figures of crystallographic data, typical diffraction frame and crystal morphology, and NMR spectra, discussions of X-ray crystal data, experimental details, and procedures and characterization data, and tables of atomic coordinates and equivalent isotropic displacement parameters, bond lengths and angles, anisotropic displacement parameters, and hydrogen coordinates and isotropic displacement parameters (PDF)

## References

[ref1] Sato Y.; Takimoto M.; Hayashi K.; Katsuhara T.; Takagi K.; Mori M. Novel Stereoselective Cyclization via π-Allylnickel Complex Generated from 1,3-Diene and Hydride Nickel Complex. J. Am. Chem. Soc. 1994, 116 (21), 9771–9772. 10.1021/ja00100a061.

[ref2] Takimoto M.; Hiraga Y.; Sato Y.; Mori M. Nickel-catalyzed regio- and stereoselective synthesis of homoallylic alcohol derivatives from dienes and aldehydes. Tetrahedron Lett. 1998, 39 (25), 4543–4546. 10.1016/S0040-4039(98)00827-2.

[ref3] Kimura M.; Ezoe A.; Shibata K.; Tamaru Y. Novel and Highly Regio- and Stereoselective Nickel-Catalyzed Homoallylation of Benzaldehyde with 1,3-Dienes. J. Am. Chem. Soc. 1998, 120 (16), 4033–4034. 10.1021/ja973847c. 16802822

[ref4] Kimura M.; Matsuo S.; Shibata K.; Tamaru Y. Nickel(0)-Catalyzed Three-Component Connection Reaction of Dimethylzinc, 1,3-Dienes, and Carbonyl Compounds. Angew. Chem., Int. Ed. 1999, 38 (22), 3386–3388. 10.1002/(SICI)1521-3773(19991115)38:22<3386::AID-ANIE3386>3.0.CO;2-W. 10602204

[ref5] Kimura M.; Ezoe A.; Tanaka S.; Tamaru Y. Nickel-Catalyzed Homoallylation of Aldehydes in the Presence of Water and Alcohols. Angew. Chem., Int. Ed. 2001, 40 (19), 3600–3602. 10.1002/1521-3773(20011001)40:19<3600::AID-ANIE3600>3.0.CO;2-N. 11592193

[ref6] Kimura M.; Ezoe A.; Mori M.; Iwata K.; Tamaru Y. Regio- and Stereoselective Nickel-Catalyzed Homoallylation of Aldehydes with 1,3-Dienes. J. Am. Chem. Soc. 2006, 128 (26), 8559–8568. 10.1021/ja0608904. 16802822

[ref7] aSato Y.; Takimoto M.; Mori M. Further Studies on Nickel-Promoted or –Catalyzed Cyclization of 1,3-Diene and a Tethered Carbonyl Group. J. Am. Chem. Soc. 2000, 122 (8), 1624–1634. 10.1021/ja991241d.

[ref8] Ogoshi S.; Tonomori K.; Oka M.; Kurosawa H. Reversible Carbon–Carbon Bond Formation between 1,3-Dienes and Aldehyde or Ketone on Nickel(0). J. Am. Chem. Soc. 2006, 128 (21), 7077–7086. 10.1021/ja060580l. 16719489

[ref9] aJackson E. P.; Malik H. A.; Sormunen G. J.; Baxter R. D.; Liu P.; Wang H.; Shareef A.-R.; Montgomery J. Mechanistic Basis for Regioselection and Regiodivergence in Nickel-Catalyzed Reductive Couplings. Acc. Chem. Res. 2015, 48 (6), 1736–1745. 10.1021/acs.accounts.5b00096. 25965694 PMC4470851

[ref10] aHolmes M.; Schwartz L. A.; Krische M. J. Intermolecular Metal-Catalyzed Reductive Coupling of Dienes, Allenes, and Enynes with Carbonyl Compounds and Imines. Chem. Rev. 2018, 118 (12), 6026–6052. 10.1021/acs.chemrev.8b00213. 29897740 PMC6203947

[ref11] aZbieg J. R.; Moran J.; Krische M. J. Diastereo- and Enantioselective Ruthenium-Catalyzed Hydro-hydroxyalkylation of 2-Silyl-butadienes: Carbonyl *syn*-Crotylation from the Alcohol Oxidation Level. J. Am. Chem. Soc. 2011, 133 (27), 10582–10586. 10.1021/ja2046028. 21627316 PMC3131435

[ref12] Saito N.; Kobayashi A.; Sato Y. Nickel-Catalyzed Enantio- and Diastereoselective Three-Component Coupling of 1,3-Dienes, Aldehydes, and a Silylborane Leading to α-Chiral Allylsilanes. Angew. Chem., Int. Ed. 2012, 51 (5), 1228–1231. 10.1002/anie.201107360. 22173932

[ref13] Yang Y.; Zhu S.-F.; Duan H.-F.; Zhou C.-Y.; Wang L.-X.; Zhou Q.-L. Asymmetric Reductive Coupling of Dienes and Aldehydes Catalyzed by Nickel Complexes of Spiro Phosphoramidites: Highly Enantioselective Synthesis of Chiral Bishomoallylic Alcohols. J. Am. Chem. Soc. 2007, 129 (8), 2248–2249. 10.1021/ja0693183. 17269780

[ref14] Sato Y.; Hinata Y.; Seki R.; Oonishi Y.; Saito N. Nickel-Catalyzed Enantio- and Diastereoselective Three-Component Coupling of 1,3-Dienes, Aldehydes, and Silanes Using Chiral N-Heterocyclic Carbenes as Ligands. Org. Lett. 2007, 9 (26), 5597–5599. 10.1021/ol702543m. 18020355

[ref15] Davies T. Q.; Murphy J. J.; Dousset M.; Fürstner A. Nickel-Catalyzed Enantioselective Synthesis of Pre-Differentiated Homoallylic *syn*- or *anti*-1,2-Diols from Aldehydes and Dienol Ethers. J. Am. Chem. Soc. 2021, 143 (34), 13489–13494. 10.1021/jacs.1c07042. 34410708 PMC8414482

[ref16] Bao J.; Wulff W. D.; Dominy J. B.; Fumo M. J.; Grant E. B.; Rob A. C.; Whitcomb M. C.; Yeung S.-M.; Ostrander R. L.; Rheingold A. L. Synthesis, Resolution, and Determination of Absolute Configuration of a Vaulted 2,2′-Binaphthol and a Vaulted 3,3′-Biphenanthrol (VAPOL). J. Am. Chem. Soc. 1996, 118 (14), 3392–3405. 10.1021/ja952018t.

[ref17] Nattmann L.; Cornella J. Ni(4-*t*Bustb)_3_: A Robust 16-Electron Ni(0) Olefin Complex for Catalysis. Organometallics 2020, 39 (18), 3295–3300. 10.1021/acs.organomet.0c00485.

[ref18] For reasons that are not entirely clear, BEt_3_ gave much better results than ZnEt_2_ in reactions of this general type; part of the problem, however, is the fact that the use of ZnEt_2_ resulted in notable ethyl transfer to the aldehyde.

[ref19] Köpfer A.; Sam B.; Breit B.; Krische M. J. Regiodivergent reductive coupling of 2-substituted dienes to formaldehyde employing ruthenium or nickel catalyst: hydrohydroxymethylation via transfer hydrogenation. Chem. Sci. 2013, 4 (4), 1876–1880. 10.1039/c3sc22051f.

[ref20] Liang T.; Zhang W.; Chen T.-Y.; Nguyen K. D.; Krische M. J. Ruthenium Catalyzed Diastereo- and Enantioselective Coupling of Propargyl Ethers with Alcohols: Siloxy-Crotylation via Hydride Shift Enabled Conversion of Alkynes to π-Allyls. J. Am. Chem. Soc. 2015, 137 (40), 13066–13071. 10.1021/jacs.5b08019. 26418572 PMC4688008

[ref21] A phenyl group at the diene terminus leads to a ca. 1:1 mixture of regioisomers, see the Supporting Information.

[ref22] Burns A. S.; Dooley C.; Carlson P. R.; Ziller J. W.; Rychnovsky S. D. Relative and Absolute Structure Assignments of Alkenes Using Crystalline Osmate Derivatives for X-ray Analysis. Org. Lett. 2019, 21 (24), 10125–10129. 10.1021/acs.orglett.9b04133. 31820648

[ref23] Paquette L. A.; Chang J.; Liu Z. Synthetic Studies Aimed at (−)-Cochleamycin A. Evaluation of Late-Stage Macrocyclization Alternatives. J. Org. Chem. 2004, 69 (19), 6441–6448. 10.1021/jo049084a. 15357606

[ref24] Hanessian S.; Yang; Giroux S.; Mascitti V.; Ma J.; Raeppel F. Application of Conformation Design in Acyclic Stereoselection: Total Synthesis of Borrelidin as the Crystalline Benzene Solvate. J. Am. Chem. Soc. 2003, 125 (45), 13784–13792. 10.1021/ja030139k. 14599218

[ref25] Hanessian S.; Chahal N.; Giroux S. Iterative Synthesis of Deoxypropionate Units: The Inductor Effect in Acyclic Conformation Design. J. Org. Chem. 2006, 71 (19), 7403–7411. 10.1021/jo061098o. 16958535

[ref26] *tert*-Butyl sorbate proved to be much less reactive and selective than methyl or ethyl sorbate; for details, see the SI.

[ref27] Ogiwara Y.; Sakai N. Acyl Fluorides in Late-Transition-Metal Catalysis. Angew. Chem., Int. Ed. 2020, 59 (2), 574–594. 10.1002/anie.201902805. 30969455

[ref28] aMartín R.; Romea P.; Tey C.; Urpí F.; Vilarrasa J. Simple and Efficient Preparation of Ketones from Morpholine Amides. Synlett 1997, 12 (12), 1414–1416. 10.1055/s-1997-1050.

[ref29] Yoshimoto R.; Usuki Y.; Satoh T. Rhodium(III)-catalyzed β-Arylation and -Alkenylation of α-Trifluoromethylacrylic Acid. Chem. Lett. 2019, 48 (5), 461–464. 10.1246/cl.190024.

[ref30] aNie J.; Guo H.-C.; Cahard D.; Ma J.-A. Asymmetric Construction of Stereogenic Carbon Centers Featuring a Trifluoromethyl Group from Prochiral Trifluoromethylated Substrates. Chem. Rev. 2011, 111 (2), 455–529. 10.1021/cr100166a. 21117644

[ref31] Wu B.-B.; Xu J.; Bian K.-J.; Gao Q.; Wang X.-S. Enantioselective Synthesis of Secondary β-Trifluoromethyl Alcohols via Catalytic Asymmetric Reductive Trifluoroalkylation and Diastereoselective Reduction. J. Am. Chem. Soc. 2022, 144 (14), 6543–6550. 10.1021/jacs.2c01422. 35378033

[ref32] Buchsteiner M.; Martinez-Rodriguez L.; Jerabek P.; Pozo I.; Patzer M.; Nöthling N.; Lehmann C.; Fürstner A. Catalytic Asymmetric Fluorination of Copper Carbene Complexes: Preparative Advances and a Mechanistic Rationale. Chem.—Eur. J. 2020, 26, 2509 10.1002/chem.202000081. 31916634 PMC7065061

[ref33] McCarren P. R.; Liu P.; Cheong P. H.-Y.; Jamison T. F.; Houk K. N. Mechanism and Transition-State Structures for Nickel-Catalyzed Reductive Alkyne–Aldehyde Coupling Reactions. J. Am. Chem. Soc. 2009, 131 (19), 6654–6655. 10.1021/ja900701g. 19397371 PMC2824658

[ref34] Additional experimental data with potential mechanistic implications are contained in the SI. Factors complicating their interpretation include but are not limited to: (i) the possible reversibility of the actual C–C bond forming step (see ref (8)); (ii) the unknown rate of the retro-reaction relative to the first irreversible step; (iii) an unusually complex speciation in solution even in the absence of Et_3_B; (iv) the question of whether the diene binds to the Ni-center in an s-*trans* rather than s-*cis* manner (see refs (6) and (8),); (v) the floppiness of the seven-membered core of the phosphoramidite ligand; (vi) the multitude of conceivable secondary interactions within the loaded catalyst (ref (35)); and (viii) the fact that secondary interactions in the ligand sphere of phosphoramidites are known to exist but have only been studied for special cases (for a case study, see ref (36)).

[ref35] Neel A. J.; Hilton M. J.; Sigman M. S.; Toste F. D. Exploiting Non-Covalent π-Interactions for Catalyst Design. Nature 2017, 543, 637 10.1038/nature21701. 28358089 PMC5907483

[ref36] Mikhel I. S.; Rüegger H.; Butti P.; Camponovo F.; Huber D.; Mezzetti A. A Chiral Phosphoramidite beyond Monodentate Coordination: Secondary π-Interactions Turn a Dangling Aryl into a Two-, Four-, or Six-Electron Donor in d^6^ and d^8^ Complexes. Organometallics 2008, 27 (13), 2937–2948. 10.1021/om800064r.

[ref37] Heinrich M.; Murphy J. J.; Ilg M. K.; Letort A.; Flasz J. T.; Philipps P.; Fürstner A. Chagosensine: A Riddle Wrapped in a Mystery Inside an Enigma. J. Am. Chem. Soc. 2020, 142, 6409–6422. 10.1021/jacs.0c01700. 32142305 PMC7307910

