# Supplementary material for: Nickel-Catalyzed Enantioselective Coupling of Aldehydes and Electron-Deficient 1,3-Dienes Following an Inverse Regiochemical Course
Source: J Am Chem Soc. 2022 Oct 4;144(41):18817–22. doi: 10.1021/jacs.2c09328 (PMC9585590; doi:10.1021/jacs.2c09328)
Supplement: Supplementary file 1 — ja2c09328_si_001.pdf [file ja2c09328_si_001.pdf]

Supporting Information

Nickel-Catalyzed Enantioselective Coupling of Aldehydes and Electron-Deficient 1,3-Dienes Following an Inverse Regiochemical Course

Thomas Q. Davies,§ Jae Yeon Kim,§ and Alois Fürstner*

Max-Planck-Institut für Kohlenforschung, 45470 Mülheim/Ruhr, Germany

Email: fuerstner@kofo.mpg.de

§ These authors contributed equally

Table of Content

Supporting Crystallographic Information S2

Experimental S17

General Considerations S17

Procedures and Characterisation Data S18

Ligand Synthesis S18

Ni-catalysed Reductive Coupling of Dienes and Aldehydes S19

Preparation of the Diene Substrates S64

Assignment of the Absolute and Relative Configuration S69

NMR Spectra S70

References S108

Supporting Crystallographic Information

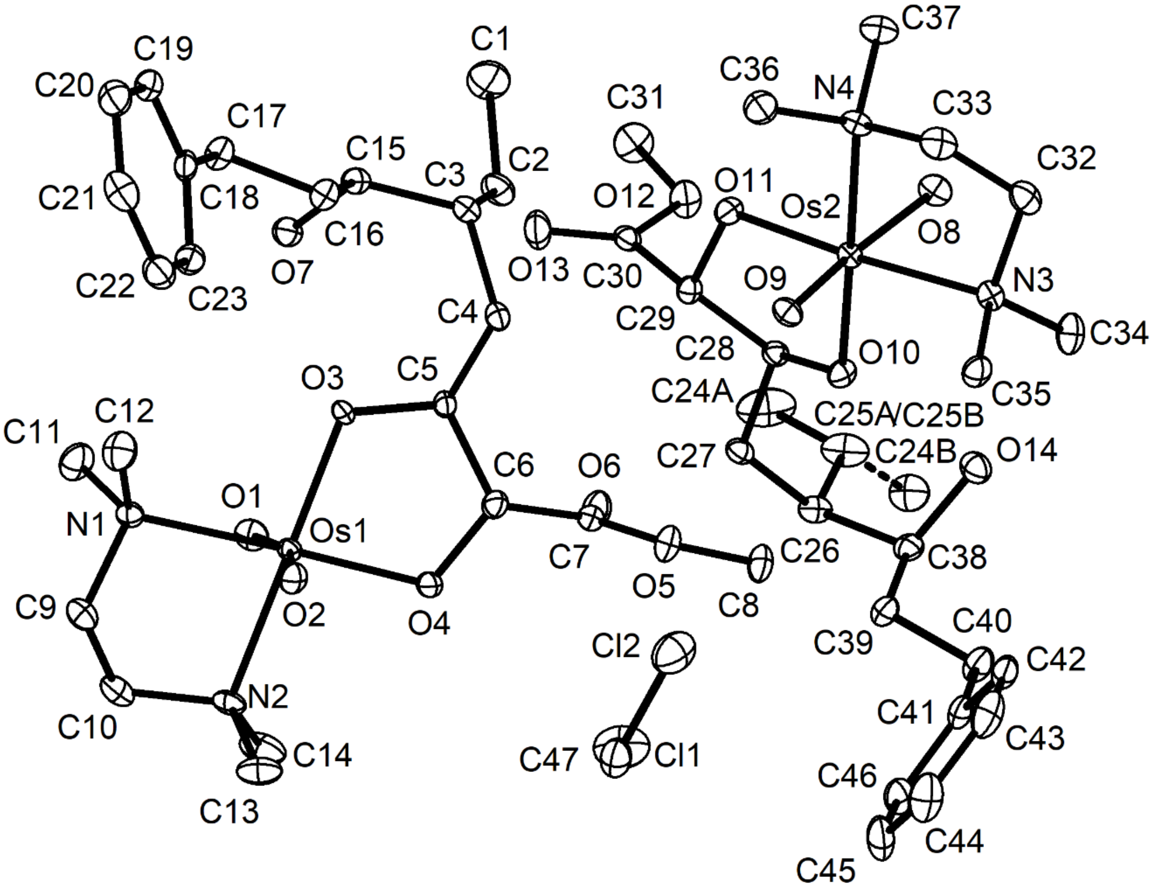

Figure S1. Structure of the osmate ester **S6** derived from product **15b** on treatment with OsO_4 and tmeda as described below: Contents of the triclinic unit cell with crystallographic numbering scheme. Atomic displacement ellipsoids are shown at the 50% probablility level, H-atoms omitted for clarity.

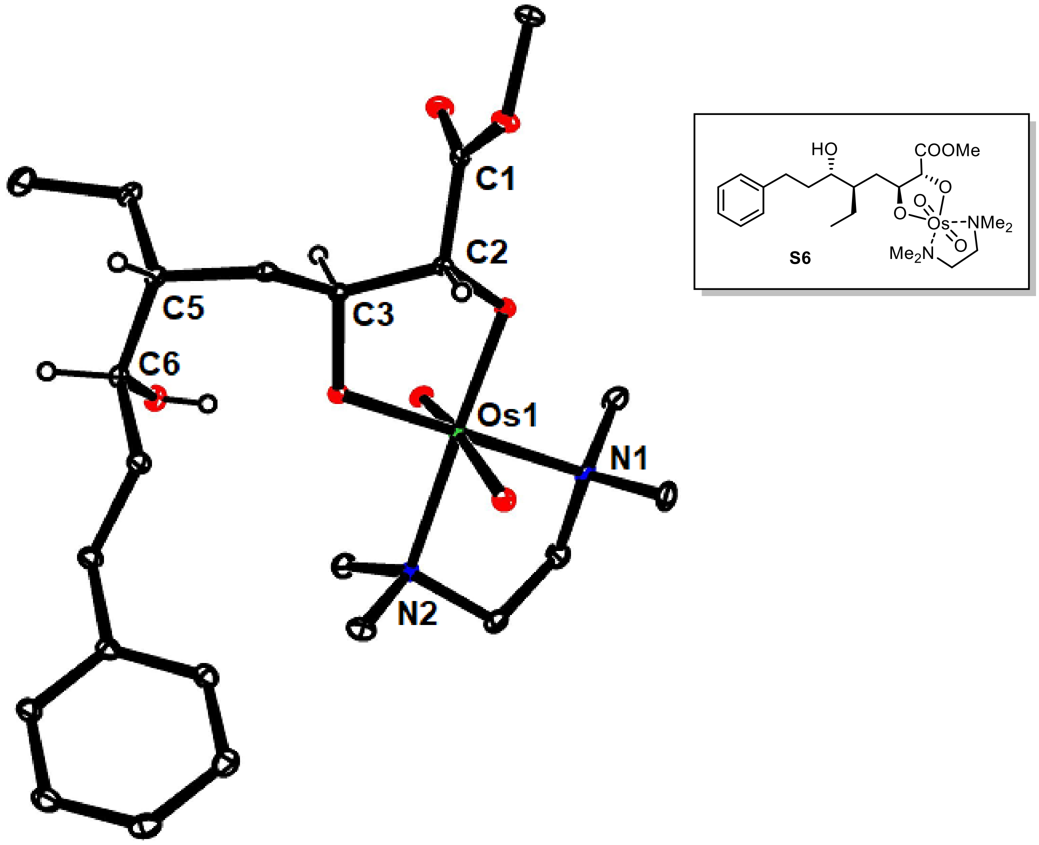

Figure S2. Structure of one of the two independent molecules of osmate ester **S6** contained in the unit cell, which has the relative and absolute configuration shown in the Insert; H-atoms (except those on the stereogenic centers and the –OH) omitted for clarity; chemical numbering scheme, with the ester carbonyl as C1.

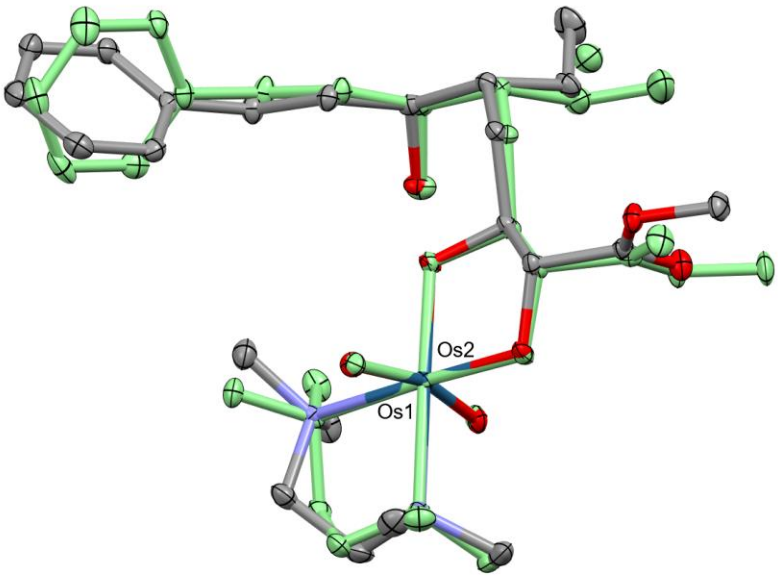

Figure S3. Overlay of the osmium coordination spheres of the two independent molecules of osmate ester **S6** in the unit cell, proving that they differ only in conformational detail (molecule 1: C grey, N blue, O red, Os Prussian blue; molecule 2: entirely in green); H-atoms omitted for clarity.

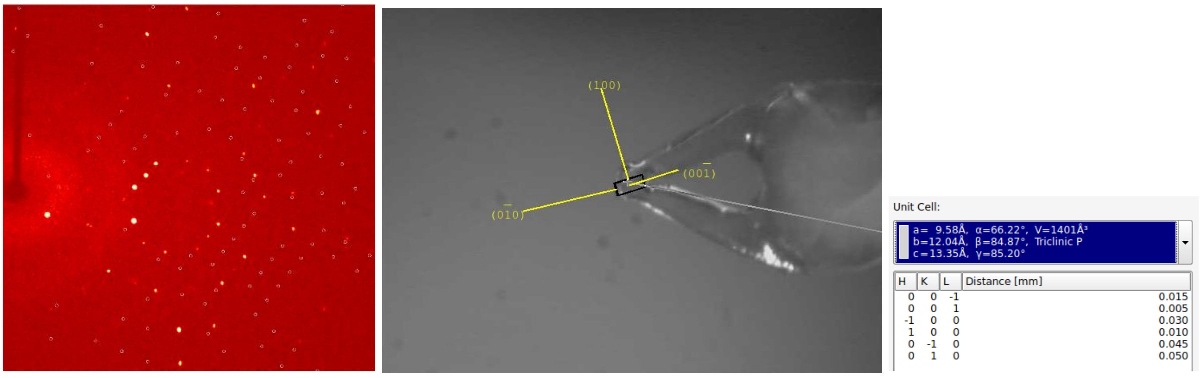

Figure S4. Typical diffraction frame and crystal morphology of **S6**.

X-ray crystal data for compound S6: $2(C_{23}H_{40}N_2O_7Os)\cdot CH_2Cl_2$, *Mr* = 1378.46 g · mol^{-1}, yellow plate, crystal size 0.032 x 0.065 x 0.116 mm^3, triclinic, space group *P*1, *a* = 9.4864(6) Å, *b* = 11.9267(7) Å, *c* = 13.2110(8) Å, *α* = 66.150(2)°, *β* = 85.028(2)°, *γ* = 85.393(2)°, *V* = 1360.25(14) Å^3, *T* = 100(2) K, *Z* = 1, d_{calc} = 1.683 g · cm^3, *λ* = 0.71073 Å, *μ*(Mo-*K*α) = 4.829 mm^{-1}, Gaussian absorption correction (*T*min = 0.63424, *T*max = 0.91242), Bruker AXS Enraf-Nonius Mach3 Apex II IμS diffractometer, 1.688 < *ϑ* < 35.033°, 54191 measured reflections, 21516 independent reflections, 19814 reflections with *I* > 2*σ*(*I*), *R*int = 0.0261.

INTENSITY STATISTICS FOR DATASET (Friedel pairs not merged)

Resolution	#Data	#Theory	%Complete	Redundancy	Mean I	Mean I/s	Rmerge	Rsigma
Inf - 2.58	331	336	98.5	4.66	227.82	76.98	0.0199	0.0121
2.58 - 1.73	753	768	98.0	4.68	157.82	69.62	0.0187	0.0130
1.73 - 1.37	1084	1106	98.0	4.67	121.58	62.52	0.0189	0.0141
1.37 - 1.19	1138	1158	98.3	4.56	94.87	56.64	0.0212	0.0157
1.19 - 1.09	1014	1030	98.4	4.22	71.37	46.69	0.0257	0.0185
1.09 - 1.01	1091	1136	96.0	3.10	65.95	37.46	0.0271	0.0250
1.01 - 0.95	1055	1118	94.4	2.57	56.90	30.50	0.0309	0.0306
0.95 - 0.90	1109	1192	93.0	2.23	52.72	26.48	0.0313	0.0359
0.90 - 0.85	1312	1426	92.0	2.00	45.57	22.90	0.0330	0.0422
0.85 - 0.82	989	1070	92.4	1.90	40.17	20.32	0.0354	0.0477
0.82 - 0.79	1093	1200	91.1	1.82	36.39	18.60	0.0386	0.0541
0.79 - 0.76	1324	1460	90.7	1.74	34.20	17.00	0.0407	0.0581
0.76 - 0.74	944	1054	89.6	1.69	29.76	15.22	0.0437	0.0660
0.74 - 0.72	1109	1230	90.2	1.61	28.18	13.98	0.0463	0.0728
0.72 - 0.70	1153	1316	87.6	1.56	26.88	13.44	0.0505	0.0774
0.70 - 0.69	663	734	90.3	1.53	25.03	12.40	0.0551	0.0824
0.69 - 0.67	1409	1620	87.0	1.47	22.80	11.24	0.0596	0.0922
0.67 - 0.66	730	848	86.1	1.44	21.13	10.41	0.0681	0.0990
0.66 - 0.64	1662	1944	85.5	1.38	19.64	9.83	0.0717	0.1080
0.64 - 0.63	829	994	83.4	1.32	17.05	8.69	0.0790	0.1218
0.63 - 0.62	724	1288	56.2	0.81	17.44	8.47	0.0851	0.1249
0.72 - 0.62	7170	8744	82.0	1.35	21.55	10.71	0.0634	0.0976
Inf - 0.62	21516	24028	89.5	2.26	50.90	26.01	0.0260	0.0379

The structure was solved by direct methods and refined by full-matrix least-squares against F^2 to $R1$ = 0.0234 [$I > 2\sigma(I)$], $wR2$ = 0.0425, 650 parameters and 3 restraints. The methyl group of the ethyl substituent of one of the two independent molecules in the unit cell is partially disordered over two positions. The occupancies of the two components were fixed at 0.6 and 0.4, respectively. The pivot atoms were constrained to have equal atomic positions and atomic displacement parameters because the diffraction data were not of sufficient accuracy to resolve the difference between the two positions. Hydroxyl H-atoms were refined using a $U_H = 1.5xU_{eqO}$. Otherwise, H-atoms were refined using a riding model with $U_H = 1.2xU_{eqC}$ for the methylene groups and with $U_H = 1.5xU_{eqC}$ for the methyl groups.

The absolute structure was determined and the Flack parameter is –0.019(2) based on 8224 quotients $[(I^+)-(I^-)]/[(I^+)+(I^-)]$. S = 1.019, residual electron density 1.84 (0.75 Å from Os2/ -1.41 (0.43 Å from Os1) e Å^{-3}. **CCDC 2203171**.

Table S2. Atomic coordinates and equivalent isotropic displacement parameters (Å^2).

U_{eq} is defined as one third of the trace of the orthogonalized U_{ij} tensor.

	x	y	z	U_{eq}
C(1)	0.5083(5)	0.7979(3)	0.0873(3)	0.031(1)
C(2)	0.5296(4)	0.6807(3)	0.1904(3)	0.019(1)
C(3)	0.5218(3)	0.5620(3)	0.1723(2)	0.013(1)
C(4)	0.5159(3)	0.4501(3)	0.2842(2)	0.013(1)
C(5)	0.6464(3)	0.4161(3)	0.3515(2)	0.012(1)
C(6)	0.6194(3)	0.3214(3)	0.4691(2)	0.014(1)
C(7)	0.5381(3)	0.3779(3)	0.5428(2)	0.014(1)
C(8)	0.3467(3)	0.3586(3)	0.6716(3)	0.019(1)
C(9)	1.1506(4)	0.1271(3)	0.3538(3)	0.021(1)
C(10)	1.1811(4)	0.1383(3)	0.4595(3)	0.023(1)
C(11)	1.1401(4)	0.3427(3)	0.2248(3)	0.023(1)
C(12)	0.9941(4)	0.2022(3)	0.2030(3)	0.023(1)
C(13)	0.9988(4)	0.0153(3)	0.5920(3)	0.024(1)
C(14)	1.0755(4)	0.1938(3)	0.6068(3)	0.026(1)
C(15)	0.6348(3)	0.5521(3)	0.0850(3)	0.014(1)
C(16)	0.6234(3)	0.4381(3)	0.0608(3)	0.015(1)
C(17)	0.7234(4)	0.4380(3)	-0.0364(3)	0.017(1)
C(18)	0.7152(3)	0.3263(3)	-0.0629(3)	0.015(1)
C(19)	0.6962(3)	0.3372(3)	-0.1693(3)	0.018(1)
C(20)	0.6932(4)	0.2340(3)	-0.1933(3)	0.022(1)
C(21)	0.7039(4)	0.1177(3)	-0.1100(3)	0.022(1)
C(22)	0.7219(4)	0.1047(3)	-0.0031(3)	0.021(1)
C(23)	0.7282(3)	0.2077(3)	0.0196(3)	0.017(1)
C(26)	0.4537(3)	0.7604(3)	0.7235(3)	0.015(1)
C(27)	0.4860(3)	0.7382(3)	0.6166(2)	0.013(1)
C(28)	0.3736(3)	0.7811(3)	0.5316(2)	0.012(1)
C(29)	0.4298(3)	0.7845(3)	0.4188(2)	0.012(1)
C(30)	0.5239(3)	0.8903(3)	0.3535(2)	0.013(1)
C(31)	0.5485(4)	1.1026(3)	0.2941(3)	0.025(1)
C(32)	-0.1568(3)	0.6753(3)	0.4518(3)	0.018(1)
C(33)	-0.0993(3)	0.6643(3)	0.3463(3)	0.019(1)
C(34)	-0.0931(4)	0.6715(4)	0.6268(3)	0.021(1)
C(35)	-0.0368(3)	0.4947(3)	0.5863(3)	0.017(1)

C(36)	0.1133(4)	0.6870(3)	0.2251(3)	0.021(1)
C(37)	-0.0109(4)	0.8669(3)	0.2326(3)	0.021(1)
C(38)	0.3274(3)	0.6924(3)	0.7966(3)	0.015(1)
C(39)	0.3387(3)	0.5543(3)	0.8276(3)	0.015(1)
C(40)	0.2268(4)	0.4861(3)	0.9183(3)	0.020(1)
C(41)	0.2184(3)	0.3540(3)	0.9350(3)	0.016(1)
C(42)	0.1019(3)	0.3152(3)	0.9025(3)	0.019(1)
C(43)	0.0944(5)	0.1945(4)	0.9162(4)	0.025(1)
C(44)	0.2033(4)	0.1097(4)	0.9625(3)	0.027(1)
C(45)	0.3210(4)	0.1465(3)	0.9947(3)	0.026(1)
C(46)	0.3267(3)	0.2669(3)	0.9823(3)	0.019(1)
C(47)	0.9420(4)	0.7583(4)	0.9092(4)	0.023(1)
Cl(1)	0.9981(1)	0.8868(1)	0.9275(1)	0.038(1)
Cl(2)	0.7935(1)	0.8005(1)	0.8269(1)	0.033(1)
N(1)	1.0567(3)	0.2336(2)	0.2864(2)	0.013(1)
N(2)	1.0468(3)	0.1406(2)	0.5274(2)	0.015(1)
N(3)	-0.0472(3)	0.6302(2)	0.5364(2)	0.013(1)
N(4)	0.0302(3)	0.7367(2)	0.2998(2)	0.013(1)
O(1)	0.9970(2)	0.3753(2)	0.3986(2)	0.016(1)
O(2)	0.8370(2)	0.1200(2)	0.4123(2)	0.015(1)
O(3)	0.7568(2)	0.3631(2)	0.3006(2)	0.014(1)
O(4)	0.7514(2)	0.2731(2)	0.5181(2)	0.017(1)
O(5)	0.4247(2)	0.3143(2)	0.5951(2)	0.017(1)
O(6)	0.5731(2)	0.4663(2)	0.5551(2)	0.019(1)
O(7)	0.7759(2)	0.5602(2)	0.1117(2)	0.016(1)
O(8)	0.0530(2)	0.8488(2)	0.4376(2)	0.016(1)
O(9)	0.2004(2)	0.5717(2)	0.4450(2)	0.014(1)
O(10)	0.2586(2)	0.6996(2)	0.5683(2)	0.014(1)
O(11)	0.3139(2)	0.7993(2)	0.3517(2)	0.014(1)
O(12)	0.4713(2)	0.9950(2)	0.3595(2)	0.019(1)
O(13)	0.6306(2)	0.8842(2)	0.2994(2)	0.023(1)
O(14)	0.1955(3)	0.7435(2)	0.7494(2)	0.018(1)
Os(1)	0.8932(1)	0.2569(1)	0.4081(1)	0.010(1)
Os(2)	0.1490(1)	0.7163(1)	0.4418(1)	0.010(1)
C(24A)	0.5483(8)	0.9793(6)	0.6314(6)	0.037(2)
C(25A)	0.4382(4)	0.8976(3)	0.7003(3)	0.025(1)
C(24B)	0.4175(10)	0.9397(9)	0.7887(8)	0.024(2)
C(25B)	0.4382(4)	0.8976(3)	0.7003(3)	0.025(1)

Table S3. Bond lengths [Å] and angles [°].

C(1)-C(2)	1.521(5)	C(1)-H(1A)	0.9800
C(1)-H(1B)	0.9800	C(1)-H(1C)	0.9800
C(2)-C(3)	1.536(5)	C(2)-H(2A)	0.9900
C(2)-H(2B)	0.9900	C(3)-C(4)	1.539(4)
C(3)-C(15)	1.540(4)	C(3)-H(3)	1.0000
C(4)-C(5)	1.518(4)	C(4)-H(4A)	0.9900
C(4)-H(4B)	0.9900	C(5)-O(3)	1.437(3)
C(5)-C(6)	1.521(4)	C(5)-H(5)	1.0000
C(6)-O(4)	1.429(4)	C(6)-C(7)	1.520(4)
C(6)-H(6)	1.0000	C(7)-O(6)	1.205(4)
C(7)-O(5)	1.337(4)	C(8)-O(5)	1.446(4)
C(8)-H(8A)	0.9800	C(8)-H(8B)	0.9800
C(8)-H(8C)	0.9800	C(9)-N(1)	1.497(4)
C(9)-C(10)	1.509(5)	C(9)-H(9A)	0.9900
C(9)-H(9B)	0.9900	C(10)-N(2)	1.498(4)
C(10)-H(10A)	0.9900	C(10)-H(10B)	0.9900
C(11)-N(1)	1.476(5)	C(11)-H(11A)	0.9800
C(11)-H(11B)	0.9800	C(11)-H(11C)	0.9800
C(12)-N(1)	1.479(4)	C(12)-H(12A)	0.9800
C(12)-H(12B)	0.9800	C(12)-H(12C)	0.9800
C(13)-N(2)	1.476(4)	C(13)-H(13A)	0.9800
C(13)-H(13B)	0.9800	C(13)-H(13C)	0.9800
C(14)-N(2)	1.480(4)	C(14)-H(14A)	0.9800
C(14)-H(14B)	0.9800	C(14)-H(14C)	0.9800
C(15)-O(7)	1.433(4)	C(15)-C(16)	1.530(4)
C(15)-H(15)	1.0000	C(16)-C(17)	1.529(4)
C(16)-H(16A)	0.9900	C(16)-H(16B)	0.9900
C(17)-C(18)	1.517(5)	C(17)-H(17A)	0.9900
C(17)-H(17B)	0.9900	C(18)-C(19)	1.384(5)
C(18)-C(23)	1.398(4)	C(19)-C(20)	1.393(5)
C(19)-H(19)	0.9500	C(20)-C(21)	1.382(5)
C(20)-H(20)	0.9500	C(21)-C(22)	1.382(5)
C(21)-H(21)	0.9500	C(22)-C(23)	1.384(5)
C(22)-H(22)	0.9500	C(23)-H(23)	0.9500
C(26)-C(38)	1.533(5)	C(26)-C(25B)	1.535(5)
C(26)-C(25A)	1.535(5)	C(26)-C(27)	1.540(4)
C(26)-H(26)	1.0000	C(27)-C(28)	1.521(4)
C(27)-H(27A)	0.9900	C(27)-H(27B)	0.9900
C(28)-O(10)	1.438(4)	C(28)-C(29)	1.524(4)
C(28)-H(28)	1.0000	C(29)-O(11)	1.428(4)
C(29)-C(30)	1.517(4)	C(29)-H(29)	1.0000
C(30)-O(13)	1.202(4)	C(30)-O(12)	1.337(4)
C(31)-O(12)	1.440(4)	C(31)-H(31A)	0.9800
C(31)-H(31B)	0.9800	C(31)-H(31C)	0.9800
C(32)-N(3)	1.499(4)	C(32)-C(33)	1.504(5)
C(32)-H(32A)	0.9900	C(32)-H(32B)	0.9900
C(33)-N(4)	1.498(4)	C(33)-H(33A)	0.9900
C(33)-H(33B)	0.9900	C(34)-N(3)	1.486(4)

C(34)-H(34A)	0.9800	C(34)-H(34B)	0.9800
C(34)-H(34C)	0.9800	C(35)-N(3)	1.477(4)
C(35)-H(35A)	0.9800	C(35)-H(35B)	0.9800
C(35)-H(35C)	0.9800	C(36)-N(4)	1.485(4)
C(36)-H(36A)	0.9800	C(36)-H(36B)	0.9800
C(36)-H(36C)	0.9800	C(37)-N(4)	1.484(4)
C(37)-H(37A)	0.9800	C(37)-H(37B)	0.9800
C(37)-H(37C)	0.9800	C(38)-O(14)	1.425(4)
C(38)-C(39)	1.525(4)	C(38)-H(38)	1.0000
C(39)-C(40)	1.534(4)	C(39)-H(39A)	0.9900
C(39)-H(39B)	0.9900	C(40)-C(41)	1.508(5)
C(40)-H(40A)	0.9900	C(40)-H(40B)	0.9900
C(41)-C(46)	1.393(5)	C(41)-C(42)	1.394(5)
C(42)-C(43)	1.384(6)	C(42)-H(42)	0.9500
C(43)-C(44)	1.380(6)	C(43)-H(43)	0.9500
C(44)-C(45)	1.390(6)	C(44)-H(44)	0.9500
C(45)-C(46)	1.383(5)	C(45)-H(45)	0.9500
C(46)-H(46)	0.9500	C(47)-Cl(2)	1.769(4)
C(47)-Cl(1)	1.773(4)	C(47)-H(47A)	0.9900
C(47)-H(47B)	0.9900	N(1)-Os(1)	2.220(3)
N(2)-Os(1)	2.199(2)	N(3)-Os(2)	2.223(3)
N(4)-Os(2)	2.192(3)	O(1)-Os(1)	1.743(2)
O(2)-Os(1)	1.737(2)	O(3)-Os(1)	1.974(2)
O(4)-Os(1)	1.951(2)	O(7)-H(7)	0.74(4)
O(8)-Os(2)	1.742(2)	O(9)-Os(2)	1.740(2)
O(10)-Os(2)	1.978(2)	O(11)-Os(2)	1.954(2)
O(14)-H(14)	0.72(4)	C(24A)-C(25A)	1.467(8)
C(24A)-H(24A)	0.9800	C(24A)-H(24B)	0.9800
C(24A)-H(24C)	0.9800	C(25A)-H(25A)	0.9900
C(25A)-H(25B)	0.9900	C(24B)-C(25B)	1.440(10)
C(24B)-H(24D)	0.9800	C(24B)-H(24E)	0.9800
C(24B)-H(24F)	0.9800	C(25B)-H(25C)	0.9900
C(25B)-H(25D)	0.9900		
C(2)-C(1)-H(1A)	109.5	C(2)-C(1)-H(1B)	109.5
H(1A)-C(1)-H(1B)	109.5	C(2)-C(1)-H(1C)	109.5
H(1A)-C(1)-H(1C)	109.5	H(1B)-C(1)-H(1C)	109.5
C(1)-C(2)-C(3)	114.5(3)	C(1)-C(2)-H(2A)	108.6
C(3)-C(2)-H(2A)	108.6	C(1)-C(2)-H(2B)	108.6
C(3)-C(2)-H(2B)	108.6	H(2A)-C(2)-H(2B)	107.6
C(2)-C(3)-C(4)	110.5(3)	C(2)-C(3)-C(15)	112.3(3)
C(4)-C(3)-C(15)	115.7(2)	C(2)-C(3)-H(3)	105.8
C(4)-C(3)-H(3)	105.8	C(15)-C(3)-H(3)	105.8
C(5)-C(4)-C(3)	117.7(3)	C(5)-C(4)-H(4A)	107.9
C(3)-C(4)-H(4A)	107.9	C(5)-C(4)-H(4B)	107.9
C(3)-C(4)-H(4B)	107.9	H(4A)-C(4)-H(4B)	107.2
O(3)-C(5)-C(4)	110.1(2)	O(3)-C(5)-C(6)	106.3(2)
C(4)-C(5)-C(6)	113.1(3)	O(3)-C(5)-H(5)	109.1
C(4)-C(5)-H(5)	109.1	C(6)-C(5)-H(5)	109.1
O(4)-C(6)-C(7)	106.3(2)	O(4)-C(6)-C(5)	109.6(2)

C(7)-C(6)-C(5)	111.8(2)	O(4)-C(6)-H(6)	109.7
C(7)-C(6)-H(6)	109.7	C(5)-C(6)-H(6)	109.7
O(6)-C(7)-O(5)	124.2(3)	O(6)-C(7)-C(6)	124.6(3)
O(5)-C(7)-C(6)	111.1(3)	O(5)-C(8)-H(8A)	109.5
O(5)-C(8)-H(8B)	109.5	H(8A)-C(8)-H(8B)	109.5
O(5)-C(8)-H(8C)	109.5	H(8A)-C(8)-H(8C)	109.5
H(8B)-C(8)-H(8C)	109.5	N(1)-C(9)-C(10)	110.0(3)
N(1)-C(9)-H(9A)	109.7	C(10)-C(9)-H(9A)	109.7
N(1)-C(9)-H(9B)	109.7	C(10)-C(9)-H(9B)	109.7
H(9A)-C(9)-H(9B)	108.2	N(2)-C(10)-C(9)	110.9(3)
N(2)-C(10)-H(10A)	109.5	C(9)-C(10)-H(10A)	109.5
N(2)-C(10)-H(10B)	109.5	C(9)-C(10)-H(10B)	109.5
H(10A)-C(10)-H(10B)	108.0	N(1)-C(11)-H(11A)	109.5
N(1)-C(11)-H(11B)	109.5	H(11A)-C(11)-H(11B)	109.5
N(1)-C(11)-H(11C)	109.5	H(11A)-C(11)-H(11C)	109.5
H(11B)-C(11)-H(11C)	109.5	N(1)-C(12)-H(12A)	109.5
N(1)-C(12)-H(12B)	109.5	H(12A)-C(12)-H(12B)	109.5
N(1)-C(12)-H(12C)	109.5	H(12A)-C(12)-H(12C)	109.5
H(12B)-C(12)-H(12C)	109.5	N(2)-C(13)-H(13A)	109.5
N(2)-C(13)-H(13B)	109.5	H(13A)-C(13)-H(13B)	109.5
N(2)-C(13)-H(13C)	109.5	H(13A)-C(13)-H(13C)	109.5
H(13B)-C(13)-H(13C)	109.5	N(2)-C(14)-H(14A)	109.5
N(2)-C(14)-H(14B)	109.5	H(14A)-C(14)-H(14B)	109.5
N(2)-C(14)-H(14C)	109.5	H(14A)-C(14)-H(14C)	109.5
H(14B)-C(14)-H(14C)	109.5	O(7)-C(15)-C(16)	110.9(2)
O(7)-C(15)-C(3)	112.6(2)	C(16)-C(15)-C(3)	113.2(3)
O(7)-C(15)-H(15)	106.5	C(16)-C(15)-H(15)	106.5
C(3)-C(15)-H(15)	106.5	C(17)-C(16)-C(15)	112.5(3)
C(17)-C(16)-H(16A)	109.1	C(15)-C(16)-H(16A)	109.1
C(17)-C(16)-H(16B)	109.1	C(15)-C(16)-H(16B)	109.1
H(16A)-C(16)-H(16B)	107.8	C(18)-C(17)-C(16)	114.1(3)
C(18)-C(17)-H(17A)	108.7	C(16)-C(17)-H(17A)	108.7
C(18)-C(17)-H(17B)	108.7	C(16)-C(17)-H(17B)	108.7
H(17A)-C(17)-H(17B)	107.6	C(19)-C(18)-C(23)	117.3(3)
C(19)-C(18)-C(17)	121.7(3)	C(23)-C(18)-C(17)	121.0(3)
C(18)-C(19)-C(20)	121.3(3)	C(18)-C(19)-H(19)	119.4
C(20)-C(19)-H(19)	119.4	C(21)-C(20)-C(19)	120.4(3)
C(21)-C(20)-H(20)	119.8	C(19)-C(20)-H(20)	119.8
C(20)-C(21)-C(22)	119.3(3)	C(20)-C(21)-H(21)	120.4
C(22)-C(21)-H(21)	120.4	C(21)-C(22)-C(23)	119.9(3)
C(21)-C(22)-H(22)	120.0	C(23)-C(22)-H(22)	120.0
C(22)-C(23)-C(18)	121.8(3)	C(22)-C(23)-H(23)	119.1
C(18)-C(23)-H(23)	119.1	C(38)-C(26)-C(25B)	110.7(3)
C(38)-C(26)-C(25A)	110.7(3)	C(38)-C(26)-C(27)	114.5(3)
C(25B)-C(26)-C(27)	112.2(3)	C(25A)-C(26)-C(27)	112.2(3)
C(38)-C(26)-H(26)	106.2	C(25A)-C(26)-H(26)	106.2
C(27)-C(26)-H(26)	106.2	C(28)-C(27)-C(26)	117.6(2)
C(28)-C(27)-H(27A)	107.9	C(26)-C(27)-H(27A)	107.9
C(28)-C(27)-H(27B)	107.9	C(26)-C(27)-H(27B)	107.9
H(27A)-C(27)-H(27B)	107.2	O(10)-C(28)-C(27)	110.4(2)

O(10)-C(28)-C(29)	106.8(2)	C(27)-C(28)-C(29)	112.9(2)
O(10)-C(28)-H(28)	108.9	C(27)-C(28)-H(28)	108.9
C(29)-C(28)-H(28)	108.9	O(11)-C(29)-C(30)	105.9(2)
O(11)-C(29)-C(28)	109.5(2)	C(30)-C(29)-C(28)	114.0(3)
O(11)-C(29)-H(29)	109.1	C(30)-C(29)-H(29)	109.1
C(28)-C(29)-H(29)	109.1	O(13)-C(30)-O(12)	123.0(3)
O(13)-C(30)-C(29)	125.4(3)	O(12)-C(30)-C(29)	111.6(2)
O(12)-C(31)-H(31A)	109.5	O(12)-C(31)-H(31B)	109.5
H(31A)-C(31)-H(31B)	109.5	O(12)-C(31)-H(31C)	109.5
H(31A)-C(31)-H(31C)	109.5	H(31B)-C(31)-H(31C)	109.5
N(3)-C(32)-C(33)	110.3(3)	N(3)-C(32)-H(32A)	109.6
C(33)-C(32)-H(32A)	109.6	N(3)-C(32)-H(32B)	109.6
C(33)-C(32)-H(32B)	109.6	H(32A)-C(32)-H(32B)	108.1
N(4)-C(33)-C(32)	111.0(3)	N(4)-C(33)-H(33A)	109.4
C(32)-C(33)-H(33A)	109.4	N(4)-C(33)-H(33B)	109.4
C(32)-C(33)-H(33B)	109.4	H(33A)-C(33)-H(33B)	108.0
N(3)-C(34)-H(34A)	109.5	N(3)-C(34)-H(34B)	109.5
H(34A)-C(34)-H(34B)	109.5	N(3)-C(34)-H(34C)	109.5
H(34A)-C(34)-H(34C)	109.5	H(34B)-C(34)-H(34C)	109.5
N(3)-C(35)-H(35A)	109.5	N(3)-C(35)-H(35B)	109.5
H(35A)-C(35)-H(35B)	109.5	N(3)-C(35)-H(35C)	109.5
H(35A)-C(35)-H(35C)	109.5	H(35B)-C(35)-H(35C)	109.5
N(4)-C(36)-H(36A)	109.5	N(4)-C(36)-H(36B)	109.5
H(36A)-C(36)-H(36B)	109.5	N(4)-C(36)-H(36C)	109.5
H(36A)-C(36)-H(36C)	109.5	H(36B)-C(36)-H(36C)	109.5
N(4)-C(37)-H(37A)	109.5	N(4)-C(37)-H(37B)	109.5
H(37A)-C(37)-H(37B)	109.5	N(4)-C(37)-H(37C)	109.5
H(37A)-C(37)-H(37C)	109.5	H(37B)-C(37)-H(37C)	109.5
O(14)-C(38)-C(39)	110.4(3)	O(14)-C(38)-C(26)	112.3(2)
C(39)-C(38)-C(26)	113.8(3)	O(14)-C(38)-H(38)	106.7
C(39)-C(38)-H(38)	106.7	C(26)-C(38)-H(38)	106.7
C(38)-C(39)-C(40)	112.4(3)	C(38)-C(39)-H(39A)	109.1
C(40)-C(39)-H(39A)	109.1	C(38)-C(39)-H(39B)	109.1
C(40)-C(39)-H(39B)	109.1	H(39A)-C(39)-H(39B)	107.9
C(41)-C(40)-C(39)	113.1(3)	C(41)-C(40)-H(40A)	109.0
C(39)-C(40)-H(40A)	109.0	C(41)-C(40)-H(40B)	109.0
C(39)-C(40)-H(40B)	109.0	H(40A)-C(40)-H(40B)	107.8
C(46)-C(41)-C(42)	117.7(3)	C(46)-C(41)-C(40)	121.4(3)
C(42)-C(41)-C(40)	120.9(3)	C(43)-C(42)-C(41)	121.2(3)
C(43)-C(42)-H(42)	119.4	C(41)-C(42)-H(42)	119.4
C(44)-C(43)-C(42)	120.3(4)	C(44)-C(43)-H(43)	119.9
C(42)-C(43)-H(43)	119.9	C(43)-C(44)-C(45)	119.6(4)
C(43)-C(44)-H(44)	120.2	C(45)-C(44)-H(44)	120.2
C(46)-C(45)-C(44)	119.8(3)	C(46)-C(45)-H(45)	120.1
C(44)-C(45)-H(45)	120.1	C(45)-C(46)-C(41)	121.4(3)
C(45)-C(46)-H(46)	119.3	C(41)-C(46)-H(46)	119.3
Cl(2)-C(47)-Cl(1)	110.4(2)	Cl(2)-C(47)-H(47A)	109.6
Cl(1)-C(47)-H(47A)	109.6	Cl(2)-C(47)-H(47B)	109.6
Cl(1)-C(47)-H(47B)	109.6	H(47A)-C(47)-H(47B)	108.1
C(11)-N(1)-C(12)	107.0(3)	C(11)-N(1)-C(9)	110.6(3)

C(12)-N(1)-C(9)	108.4(3)	C(11)-N(1)-Os(1)	114.2(2)
C(12)-N(1)-Os(1)	111.85(19)	C(9)-N(1)-Os(1)	104.73(19)
C(13)-N(2)-C(14)	107.8(3)	C(13)-N(2)-C(10)	111.0(3)
C(14)-N(2)-C(10)	108.2(3)	C(13)-N(2)-Os(1)	112.6(2)
C(14)-N(2)-Os(1)	111.62(19)	C(10)-N(2)-Os(1)	105.52(18)
C(35)-N(3)-C(34)	107.3(3)	C(35)-N(3)-C(32)	110.2(2)
C(34)-N(3)-C(32)	108.5(3)	C(35)-N(3)-Os(2)	114.24(18)
C(34)-N(3)-Os(2)	112.6(2)	C(32)-N(3)-Os(2)	103.83(19)
C(37)-N(4)-C(36)	107.6(3)	C(37)-N(4)-C(33)	110.1(2)
C(36)-N(4)-C(33)	108.7(3)	C(37)-N(4)-Os(2)	112.0(2)
C(36)-N(4)-Os(2)	111.79(19)	C(33)-N(4)-Os(2)	106.49(19)
C(5)-O(3)-Os(1)	112.06(17)	C(6)-O(4)-Os(1)	110.57(17)
C(7)-O(5)-C(8)	114.2(3)	C(15)-O(7)-H(7)	112(3)
C(28)-O(10)-Os(2)	110.67(17)	C(29)-O(11)-Os(2)	111.08(17)
C(30)-O(12)-C(31)	115.8(3)	C(38)-O(14)-H(14)	106(4)
O(2)-Os(1)-O(1)	163.51(10)	O(2)-Os(1)-O(4)	96.41(10)
O(1)-Os(1)-O(4)	96.98(11)	O(2)-Os(1)-O(3)	95.29(10)
O(1)-Os(1)-O(3)	95.63(10)	O(4)-Os(1)-O(3)	84.03(9)
O(2)-Os(1)-N(2)	85.39(10)	O(1)-Os(1)-N(2)	83.66(10)
O(4)-Os(1)-N(2)	96.11(9)	O(3)-Os(1)-N(2)	179.29(10)
O(2)-Os(1)-N(1)	82.79(10)	O(1)-Os(1)-N(1)	83.58(10)
O(4)-Os(1)-N(1)	178.43(9)	O(3)-Os(1)-N(1)	97.39(9)
N(2)-Os(1)-N(1)	82.48(10)	O(9)-Os(2)-O(8)	164.79(10)
O(9)-Os(2)-O(11)	96.25(10)	O(8)-Os(2)-O(11)	96.12(10)
O(9)-Os(2)-O(10)	95.45(10)	O(8)-Os(2)-O(10)	94.54(10)
O(11)-Os(2)-O(10)	84.33(9)	O(9)-Os(2)-N(4)	84.74(10)
O(8)-Os(2)-N(4)	85.45(10)	O(11)-Os(2)-N(4)	94.73(9)
O(10)-Os(2)-N(4)	179.06(10)	O(9)-Os(2)-N(3)	85.69(10)
O(8)-Os(2)-N(3)	81.51(10)	O(11)-Os(2)-N(3)	176.41(9)
O(10)-Os(2)-N(3)	98.51(9)	N(4)-Os(2)-N(3)	82.42(10)
C(25A)-C(24A)-H(24A)	109.5	C(25A)-C(24A)-H(24B)	109.5
H(24A)-C(24A)-H(24B)	109.5	C(25A)-C(24A)-H(24C)	109.5
H(24A)-C(24A)-H(24C)	109.5	H(24B)-C(24A)-H(24C)	109.5
C(24A)-C(25A)-C(26)	119.5(4)	C(24A)-C(25A)-H(25A)	107.5
C(26)-C(25A)-H(25A)	107.5	C(24A)-C(25A)-H(25B)	107.5
C(26)-C(25A)-H(25B)	107.5	H(25A)-C(25A)-H(25B)	107.0
C(25B)-C(24B)-H(24D)	109.5	C(25B)-C(24B)-H(24E)	109.5
H(24D)-C(24B)-H(24E)	109.5	C(25B)-C(24B)-H(24F)	109.5
H(24D)-C(24B)-H(24F)	109.5	H(24E)-C(24B)-H(24F)	109.5
C(24B)-C(25B)-C(26)	121.7(5)	C(24B)-C(25B)-H(25C)	106.9
C(26)-C(25B)-H(25C)	106.9	C(24B)-C(25B)-H(25D)	106.9
C(26)-C(25B)-H(25D)	106.9	H(25C)-C(25B)-H(25D)	106.7

Table S4. Anisotropic displacement parameters (Å^2).

The anisotropic displacement factor exponent takes the form:
$-2\pi^2[h^2a^{*2}U_{11} + ... + 2 h k a^* b^* U_{12}]$.

	U_{11}	U_{22}	U_{33}	U_{23}	U_{13}	U_{12}
C(1)	0.041(2)	0.015(2)	0.031(2)	-0.004(2)	0.002(2)	0.004(2)
C(2)	0.020(2)	0.013(1)	0.023(2)	-0.007(1)	-0.003(1)	0.002(1)
C(3)	0.012(1)	0.012(1)	0.015(1)	-0.005(1)	-0.003(1)	0.001(1)
C(4)	0.011(1)	0.014(1)	0.015(1)	-0.007(1)	0.000(1)	0.001(1)
C(5)	0.010(1)	0.012(1)	0.013(1)	-0.005(1)	0.001(1)	0.001(1)
C(6)	0.016(1)	0.014(1)	0.013(1)	-0.006(1)	0.001(1)	0.001(1)
C(7)	0.014(1)	0.013(1)	0.013(1)	-0.004(1)	-0.001(1)	0.001(1)
C(8)	0.017(1)	0.025(2)	0.019(2)	-0.014(1)	0.005(1)	-0.001(1)
C(9)	0.019(2)	0.018(2)	0.023(2)	-0.006(1)	-0.002(1)	0.007(1)
C(10)	0.017(2)	0.027(2)	0.023(2)	-0.008(1)	-0.007(1)	0.007(1)
C(11)	0.027(2)	0.014(2)	0.023(2)	-0.004(2)	0.007(1)	-0.004(2)
C(12)	0.024(2)	0.025(2)	0.023(2)	-0.015(1)	0.001(1)	-0.003(1)
C(13)	0.035(2)	0.014(2)	0.020(2)	-0.001(1)	-0.009(1)	0.000(1)
C(14)	0.035(2)	0.021(2)	0.027(2)	-0.014(2)	-0.017(2)	0.009(2)
C(15)	0.014(1)	0.013(1)	0.013(1)	-0.004(1)	-0.002(1)	-0.001(1)
C(16)	0.016(1)	0.015(1)	0.015(1)	-0.007(1)	0.001(1)	-0.003(1)
C(17)	0.019(2)	0.017(2)	0.016(2)	-0.007(1)	0.003(1)	-0.003(1)
C(18)	0.010(1)	0.020(2)	0.015(1)	-0.008(1)	0.002(1)	-0.001(1)
C(19)	0.017(1)	0.023(2)	0.015(1)	-0.008(1)	0.001(1)	0.002(1)
C(20)	0.022(2)	0.030(2)	0.020(2)	-0.015(1)	-0.002(1)	0.002(1)
C(21)	0.019(2)	0.024(2)	0.031(2)	-0.018(2)	-0.005(1)	0.002(1)
C(22)	0.018(2)	0.019(2)	0.024(2)	-0.007(1)	-0.001(1)	0.001(1)
C(23)	0.015(1)	0.021(2)	0.015(1)	-0.008(1)	-0.001(1)	-0.002(1)
C(26)	0.016(1)	0.013(1)	0.015(1)	-0.004(1)	-0.004(1)	-0.004(1)
C(27)	0.011(1)	0.013(1)	0.014(1)	-0.004(1)	-0.003(1)	0.000(1)
C(28)	0.011(1)	0.012(1)	0.013(1)	-0.004(1)	-0.001(1)	-0.001(1)
C(29)	0.012(1)	0.011(1)	0.013(1)	-0.005(1)	0.001(1)	-0.002(1)
C(30)	0.011(1)	0.014(1)	0.013(1)	-0.005(1)	-0.003(1)	0.001(1)
C(31)	0.024(2)	0.014(2)	0.033(2)	-0.004(1)	0.002(1)	-0.010(1)
C(32)	0.013(1)	0.017(2)	0.024(2)	-0.007(1)	-0.002(1)	-0.002(1)
C(33)	0.018(2)	0.017(2)	0.022(2)	-0.007(1)	-0.006(1)	-0.005(1)
C(34)	0.018(2)	0.023(2)	0.025(2)	-0.016(2)	0.005(1)	-0.003(2)
C(35)	0.019(2)	0.012(1)	0.017(1)	-0.004(1)	0.004(1)	-0.003(1)
C(36)	0.023(2)	0.028(2)	0.017(2)	-0.013(1)	-0.004(1)	0.003(1)
C(37)	0.024(2)	0.016(2)	0.019(2)	-0.003(1)	-0.006(1)	0.002(1)
C(38)	0.017(1)	0.016(1)	0.012(1)	-0.007(1)	-0.002(1)	0.000(1)
C(39)	0.017(1)	0.014(1)	0.013(1)	-0.005(1)	0.003(1)	-0.003(1)

C(40)	0.025(2)	0.015(1)	0.018(2)	-0.006(1)	0.007(1)	-0.003(1)
C(41)	0.015(1)	0.016(1)	0.016(1)	-0.006(1)	0.006(1)	-0.004(1)
C(42)	0.016(1)	0.027(2)	0.015(1)	-0.009(1)	0.003(1)	-0.001(1)
C(43)	0.020(2)	0.036(2)	0.027(2)	-0.020(2)	0.008(2)	-0.017(2)
C(44)	0.026(2)	0.022(2)	0.038(2)	-0.016(2)	0.011(2)	-0.011(1)
C(45)	0.020(2)	0.022(2)	0.036(2)	-0.012(2)	0.010(2)	0.001(1)
C(46)	0.015(1)	0.018(2)	0.023(2)	-0.007(1)	0.002(1)	-0.006(1)
C(47)	0.022(2)	0.020(2)	0.026(2)	-0.009(2)	0.006(2)	-0.003(1)
Cl(1)	0.054(1)	0.030(1)	0.035(1)	-0.016(1)	-0.013(1)	-0.006(1)
Cl(2)	0.031(1)	0.040(1)	0.026(1)	-0.011(1)	0.000(1)	-0.013(1)
N(1)	0.014(1)	0.009(1)	0.014(1)	-0.003(1)	-0.001(1)	0.000(1)
N(2)	0.020(1)	0.009(1)	0.015(1)	-0.005(1)	-0.006(1)	0.005(1)
N(3)	0.012(1)	0.012(1)	0.017(1)	-0.007(1)	-0.001(1)	-0.001(1)
N(4)	0.014(1)	0.011(1)	0.015(1)	-0.005(1)	-0.005(1)	0.001(1)
O(1)	0.021(1)	0.011(1)	0.020(1)	-0.009(1)	-0.003(1)	0.001(1)
O(2)	0.014(1)	0.014(1)	0.017(1)	-0.005(1)	0.001(1)	-0.004(1)
O(3)	0.011(1)	0.018(1)	0.012(1)	-0.005(1)	-0.001(1)	0.007(1)
O(4)	0.018(1)	0.020(1)	0.011(1)	-0.006(1)	-0.001(1)	0.008(1)
O(5)	0.016(1)	0.019(1)	0.018(1)	-0.011(1)	0.005(1)	-0.005(1)
O(6)	0.020(1)	0.018(1)	0.022(1)	-0.011(1)	0.005(1)	-0.004(1)
O(7)	0.013(1)	0.016(1)	0.017(1)	-0.004(1)	-0.001(1)	-0.003(1)
O(8)	0.019(1)	0.010(1)	0.020(1)	-0.008(1)	-0.003(1)	-0.001(1)
O(9)	0.015(1)	0.011(1)	0.018(1)	-0.007(1)	-0.003(1)	0.002(1)
O(10)	0.013(1)	0.014(1)	0.013(1)	-0.004(1)	0.000(1)	-0.005(1)
O(11)	0.012(1)	0.016(1)	0.012(1)	-0.005(1)	-0.001(1)	-0.004(1)
O(12)	0.019(1)	0.010(1)	0.027(1)	-0.007(1)	0.004(1)	-0.004(1)
O(13)	0.015(1)	0.023(1)	0.027(1)	-0.009(1)	0.006(1)	0.000(1)
O(14)	0.018(1)	0.020(1)	0.019(1)	-0.011(1)	-0.004(1)	0.004(1)
Os(1)	0.012(1)	0.009(1)	0.010(1)	-0.004(1)	-0.001(1)	0.002(1)
Os(2)	0.010(1)	0.009(1)	0.013(1)	-0.005(1)	-0.002(1)	-0.001(1)
C(24A)	0.054(5)	0.024(3)	0.033(4)	-0.005(3)	-0.013(3)	-0.014(3)
C(25A)	0.036(2)	0.015(2)	0.025(2)	-0.009(1)	-0.012(2)	-0.002(1)
C(24B)	0.030(5)	0.022(4)	0.024(4)	-0.012(4)	-0.004(4)	-0.001(4)
C(25B)	0.036(2)	0.015(2)	0.025(2)	-0.009(1)	-0.012(2)	-0.002(1)

Table S5. Hydrogen coordinates and isotropic displacement parameters (Å^2).

	x	y	z	U_{eq}
H(1A)	0.4990	0.8685	0.1079	0.046
H(1B)	0.4222	0.7939	0.0532	0.046
H(1C)	0.5900	0.8068	0.0344	0.046
H(2A)	0.4565	0.6808	0.2486	0.023
H(2B)	0.6232	0.6808	0.2181	0.023
H(3)	0.4283	0.5682	0.1402	0.016
H(4A)	0.4352	0.4651	0.3306	0.016
H(4B)	0.4947	0.3781	0.2699	0.016
H(5)	0.6805	0.4919	0.3545	0.014
H(6)	0.5655	0.2534	0.4677	0.017
H(8A)	0.2695	0.3043	0.7104	0.029
H(8B)	0.3071	0.4419	0.6306	0.029
H(8C)	0.4104	0.3598	0.7257	0.029
H(9A)	1.2406	0.1251	0.3105	0.025
H(9B)	1.1036	0.0496	0.3719	0.025
H(10A)	1.2440	0.0680	0.5032	0.028
H(10B)	1.2308	0.2146	0.4412	0.028
H(11A)	1.0765	0.4125	0.1839	0.035
H(11B)	1.2092	0.3257	0.1726	0.035
H(11C)	1.1899	0.3624	0.2771	0.035
H(12A)	0.9364	0.2727	0.1555	0.034
H(12B)	0.9345	0.1319	0.2409	0.034
H(12C)	1.0700	0.1810	0.1576	0.034
H(13A)	0.9091	0.0195	0.6334	0.036
H(13B)	1.0704	-0.0326	0.6441	0.036
H(13C)	0.9853	-0.0241	0.5416	0.036
H(14A)	0.9866	0.2048	0.6461	0.039
H(14B)	1.1176	0.2735	0.5664	0.039
H(14C)	1.1414	0.1383	0.6603	0.039
H(15)	0.6169	0.6247	0.0143	0.017
H(16A)	0.6456	0.3639	0.1278	0.018
H(16B)	0.5247	0.4342	0.0439	0.018
H(17A)	0.7010	0.5126	-0.1031	0.021
H(17B)	0.8218	0.4425	-0.0193	0.021
H(19)	0.6850	0.4166	-0.2270	0.022
H(20)	0.6838	0.2437	-0.2675	0.027
H(21)	0.6990	0.0474	-0.1261	0.027
H(22)	0.7299	0.0251	0.0548	0.025
H(23)	0.7418	0.1974	0.0933	0.020
H(26)	0.5387	0.7270	0.7683	0.018
H(27A)	0.5743	0.7790	0.5797	0.016
H(27B)	0.5053	0.6489	0.6381	0.016

H(28)	0.3362	0.8652	0.5225	0.014
H(29)	0.4838	0.7054	0.4294	0.014
H(31A)	0.5056	1.1729	0.3081	0.038
H(31B)	0.5451	1.1196	0.2153	0.038
H(31C)	0.6473	1.0885	0.3144	0.038
H(32A)	-0.1851	0.7621	0.4363	0.022
H(32B)	-0.2419	0.6265	0.4812	0.022
H(33A)	-0.0754	0.5769	0.3613	0.022
H(33B)	-0.1727	0.6945	0.2912	0.022
H(34A)	-0.0250	0.6388	0.6849	0.031
H(34B)	-0.1869	0.6414	0.6582	0.031
H(34C)	-0.0975	0.7614	0.5970	0.031
H(35A)	0.0299	0.4663	0.6453	0.025
H(35B)	-0.0031	0.4643	0.5293	0.025
H(35C)	-0.1303	0.4635	0.6175	0.025
H(36A)	0.1988	0.7333	0.1947	0.032
H(36B)	0.0556	0.6948	0.1644	0.032
H(36C)	0.1401	0.6003	0.2671	0.032
H(37A)	0.0739	0.9151	0.2091	0.031
H(37B)	-0.0765	0.8990	0.2771	0.031
H(37C)	-0.0573	0.8727	0.1671	0.031
H(38)	0.3265	0.7041	0.8675	0.018
H(39A)	0.4342	0.5219	0.8538	0.018
H(39B)	0.3273	0.5386	0.7608	0.018
H(40A)	0.2493	0.4889	0.9890	0.024
H(40B)	0.1331	0.5290	0.8984	0.024
H(42)	0.0263	0.3727	0.8704	0.023
H(43)	0.0141	0.1698	0.8937	0.030
H(44)	0.1978	0.0267	0.9723	0.033
H(45)	0.3973	0.0891	1.0252	0.032
H(46)	0.4062	0.2907	1.0066	0.023
H(47A)	1.0204	0.7255	0.8728	0.028
H(47B)	0.9169	0.6929	0.9824	0.028
H(7)	0.796(4)	0.508(4)	0.163(4)	0.024
H(14)	0.194(5)	0.728(4)	0.702(4)	0.027
H(24A)	0.5397	1.0558	0.6431	0.056
H(24B)	0.5370	0.9978	0.5532	0.056
H(24C)	0.6419	0.9391	0.6521	0.056
H(25A)	0.3473	0.9299	0.6650	0.030
H(25B)	0.4295	0.9045	0.7728	0.030
H(24D)	0.4181	1.0295	0.7576	0.036
H(24E)	0.4941	0.9049	0.8395	0.036
H(24F)	0.3264	0.9134	0.8292	0.036
H(25C)	0.5241	0.9356	0.6553	0.030
H(25D)	0.3574	0.9325	0.6525	0.030

Experimental

General Considerations

Unless stated otherwise, all reactions were carried out under argon atmosphere in flame-dried Schlenk glassware. Solvents were purified by distillation over the indicated drying agents under argon: toluene (CaH_2), THF (Mg/anthracene), Et_2O (Mg/anthacene), pentane (Na/K), CH_2Cl_2 (CaH_2). MeCN and Et_3N were dried by an absorption solvent purification system based on molecular sieves. Flash chromatography: VWR Chemicals silica gel 40 – 63 µm.

NMR spectra were recorded on Bruker DPX 300 or AV 400 spectrometers in the solvents indicated; chemical shifts are given in ppm relative to TMS, coupling constants (*J*) in Hz. The solvent signals were used as references and the chemical shifts converted to the TMS scale ($CDCl_3$: δ_C = 77.16 ppm; δ_H = 7.26 ppm; C_6D_6: δ_C = 128.06 ppm; δ_H = 7.16 ppm; CD_2Cl_2: δ_C = 54.0 ppm; δ_H = 5.32 ppm). Proton and carbon assignments were established using HSQC, HMBC and NOESY experiments where necessary.

IR: Alpha Platinum ATR (Bruker), wavenumbers ($\tilde{\nu}$) in cm^{-1}.

MS (EI): Finnigan MAT 8200 (70 eV), ESI-MS: ESQ3000 (Bruker), Thermo Scientific LTQ-FT, or Thermo Scientific Exactive. HRMS: Bruker APEX III FT-MS (7T magnet), MAT 95 (Finnigan),Thermo Scientific LTQ-FT, or Thermo Scientific Exactive. GC-MS: Shimadzu GCMS-QP2010 Ultra instrument.

HPLC analyses for the determination of enantiomeric excesses were conducted on a Shimadzu LC 2020 instrument equipped with a Shimadzu SPD-M20A UV/VIS detector. Solvents (HPLC grade) were purchased and used as received. The exact conditions are stated separately for each compound.

Optical rotations were measured with an A-Krüss Otronic Model P8000-t polarimeter at a wavelength of 589 nm. The values are given as specific optical rotation with exact temperature, concentration (c/(10 mg/mL)) and solvent.

Aldehydes were purchased from commercial suppliers and distilled, except for solid aldehydes, which were typically used as received after checking purity by ^{1}H NMR spectroscopy. Unless stated otherwise, all other commercially available compounds (abcr, Acros, TCI, Aldrich, Alfa Aesar, Fluorochem) were used as received.

Procedures and Characterisation Data

Ligand Synthesis

Phosphoramidite (*R*)-VAPhos-NEt$_2$ (L1). (*R*)-VAPOL (470 mg, 0.873 mmol) was added to an oven-dried reaction tube under argon and dissolved in anhydrous toluene (4.2 mL, 0.21 M). The resulting suspension was sparged with argon for 10 min. Tris(diethylamino)phosphine (0.24 mL, 0.873 mmol) was added, the tube was sealed, and the reaction mixture was stirred at 110 °C for 16 h. After cooling to room temperature, the solution was concentrated *in vacuo* to give an orange oil. Purification by flash chromatography (SiO_2, hexane/ethyl acetate 98:2) gave phosphoramidite **L1** as a white solid (559 mg, quant.).

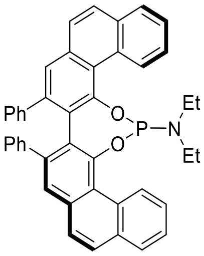

$[\alpha]_D^{20}$ = –580 (c = 0.1, CH_2Cl_2); ^{1}H (400 MHz, C_6D_6) δ = 10.24 (d, *J* = 8.5 Hz, 1H), 10.18 (d, *J* = 8.5 Hz, 1H), 7.74-7.69 (m, 2H), 7.65-7.60 (m, 1H), 7.56-7.38 (m, 9H), 6.93-6.87 (m, 2H), 6.84-6.80 (m, 2H), 6.79-6.73 (m, 6H), 2.89 (ddq, *J* = 14.0, 9.5, 7.0 Hz, 2H), 2.73-2.59 (m, 2H), 0.66 (t, *J* = 7.0 Hz, 6H); ^{13}C NMR (101 MHz, C_6D_6) Note: due to the complexity of the spectrum, a simple list of peaks without couplings (except for the alkyl signals) is given. δ = 150.9, 150.81, 150.76, 142.02, 142.00, 141.9, 140.8, 140.7, 135.0, 134.8, 134.0, 133.6, 130.7, 130.5, 130.03, 130.02, 129.47, 129.42, 129.35, 128.89, 128.84, 128.7, 128.2, 127.9, 127.7, 127.5, 127.3, 127.2, 127.1, 126.9, 126.8, 126.72, 126.67, 126.2, 122.81, 122.79, 122.7, 39.6 (d, *J* = 22.3 Hz), 14.8 (d, *J* = 3.4 Hz); ^{31}P NMR (162 MHz, C_6D_6) δ = 143.3 (s); IR (ATR): ṽ = 3051, 2967, 1594, 1556, 1369, 1327, 1173, 1124, 1016, 974, 874, 812, 741, 696, 497; LRMS *m/z* (EI$^+$) [M]$^+$ 639; HRMS (ESI$^+$, *m/z*) calculated for $[C_{44}H_{35}NO_2P]^+$ ([M$^+$]) 640.23942, found 640.23999.

Notes:

1. **L1** has not shown evidence of air-instability but is stored in the freezer at –20 °C as a precaution since the related *N*,*N*-dimethyl derivative appears to be slightly air-sensitive. Furthermore, rotary evaporation was performed at 30 °C.
2. (*R*)-VAPOL (CAS 147702-16-7) bought from Sigma-Aldrich was determined as 99.8% ee by chiral HPLC.
3. The reaction was performed in a crimp-capped vial as this was more convenient on small scale. However, many very similar reactions on BINOL derivatives have been performed under reflux in open flasks under Ar, see the Supporting Information of ref.[1]; therefore, it can be expected that this would also work well for this substrate.
4. Once it solidifies after purification and evaporation of the solvent, **L1** cannot readily be dissolved in hexane or ethyl acetate. If the isolated solid needs to be re-dissolved, toluene is more appropriate as it is well-soluble in this solvent.

Ni-catalysed Reductive Coupling of Dienes and Aldehydes

Representative Procedure: Methyl (5*R*,6*R*,*E*)-6-hydroxy-5-methyl-6-phenylhex-2-enoate

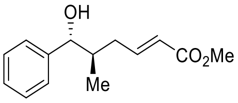

(6a). Tris(*trans*-1,2-bis(4-*tert*-butylphenyl)ethene)nickel(0) ((Ni(*t*Bu-stb)$_3$) (6.8 mg, 0.0072 mmol, 2.5 mol%)[2] and phosphoramidite **L1** (4.6 mg, 0.0072 mmol, 2.5 mol%) were added to a flame-dried thick-walled Schlenk flask under argon and dissolved in THF (0.6 mL). Methyl sorbate *E,E*-**5** (42 µL, 0.32 mmol) and triethylborane (1 M in THF, 0.44 mL, 0.44 mmol) were added. Benzaldehyde (29 µL, 0.29 mmol) was then added, the flask was sealed under argon and the mixture was stirred at room temperature for 16 h. Oxidative work-up as described below (procedure **A**) gave a light yellow oil. Purification by flash chromatography (SiO_2, hexane/ethyl acetate 9:1 to 5:1) afforded the title compound as a colourless oil (61 mg, 90% yield, >20:1 dr, 94% ee).

$[\alpha]_D^{20}$ = 11.8 (c = 0.55, $CHCl_3$); ^{1}H NMR (400 MHz, $CDCl_3$) δ = 7.37-7.26 (m, 5H), 6.99 (ddd, *J* = 15.5, 8.5, 6.5 Hz, 1H), 5.86 (dt, *J* = 15.5, 1.5 Hz, 1H), 4.42 (d, *J* = 7.5 Hz, 1H), 3.72 (s, 3H), 2.56 (dddd, *J* = 14.0, 6.5, 4.0, 1.5 Hz, 1H), 2.16 (app. dtd, *J* = 14.0, 8.5, 1.5 Hz, 1H), 2.02 (m, 1H), 1.89 (br. s, 1H, O-H), 0.77 (d, *J* = 7.0 Hz, 3H); ^{13}C NMR (101 MHz, $CDCl_3$) δ = 167.2, 148.4, 143.2, 128.5, 127.9, 126.7, 122.5, 78.5, 51.5, 39.8, 35.3, 16.1; IR (ATR): ṽ = 3468, 3029, 2954, 1419, 1703, 1653, 1436, 1271, 1168, 1023, 980, 762, 700; HRMS (EI^+, *m*/*z*) calculated for $[C_{14}H_{18}O_3]^+$ ($[M^+]$) 234.1250, found 234.1253.

NOTE: In this and all cases described below, the corresponding racemic sample needed for calibration purposes for accurate ee determination was prepared analogously using a racemic sample of ligand **L1**

The ee was determined by 2D HPLC analysis

Step 1: Purification: 100 mm Zorbax RX-Sil 1.8 µm, 4.6 mm i.D., *n*-heptane/isopropanol = 99:1, v = 1.0 mL/min, λ = 220 nm, t(major) = 6.43 min, 298 K.

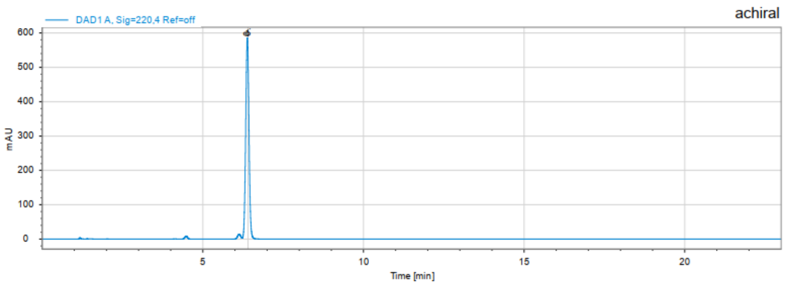

Sampling table (¹D)

Cut group	Cut #	¹D Cut start [min]	¹D Ret. time [min]	¹D Duration [min]	Trigger	²D Run start [min]
	1	6.37	6.383	0.04	Time	6.43

6a: separation of impurities on achiral column

Step 2: Chiral resolution of the major diastereomer: Daicel 150 mm Chiralpak IB-N3, 4.6 mm i.D., *n*-heptane/isopropanol = 95:5, v = 1.0 mL/min, λ = 220 nm, t(minor) = 9.92 min, t(major) = 11.93 min.

Cut# : 1

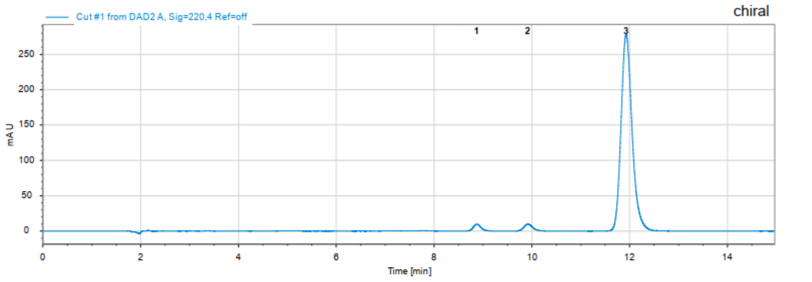

Signal: DAD2 A, Sig=220,4 Ref=off

Compound	Cut	Ret.Time	Area	Width	Height	Symmetry	
1	1	8.876	109.515<br>**109.515**	0.173	9.703	0.802	
2	1	9.921	128.653<br>**128.653**	0.200	9.860	0.838	1st enantiomer
3	1	11.928	4364.754<br>**4364.754**	0.241	278.141	0.800	2nd enantiomer

Component table

Signal: DAD2 A, Sig=220,4 Ref=off

Component	¹D Sampling range [min]	Ret.Time ²D [min]	Area	Area%		
1	6.37 - 6.41	8.876	109.515	2.379		
2	6.37 - 6.41	9.921	128.653	2.795	1st enantiomer	ee = 94.3%
3	6.37 - 6.41	11.928	4364.754	94.826	2nd enantiomer	

6a: ee determination of pure major diastereomer

Cut# : 1

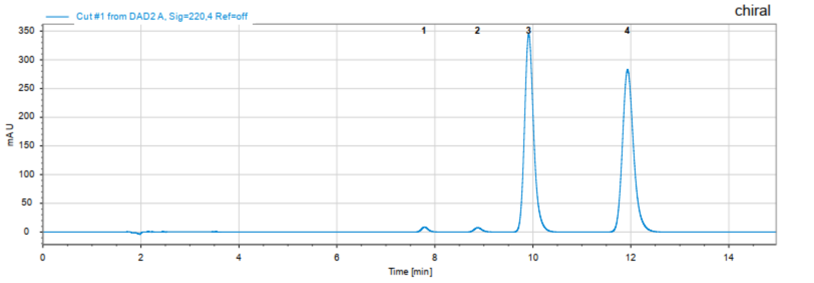

Signal: DAD2 A, Sig=220,4 Ref=off

Compound	Cut	Ret.Time	Area	Width	Height	Symmetry	
1	1	7.789	85.558<br>**85.558**	0.150	8.759	0.812	
2	1	8.876	85.901<br>**85.901**	0.171	7.674	0.814	
3	1	9.916	4463.338<br>**4463.338**	0.199	344.903	0.803	1st enantiomer
4	1	11.933	4468.042<br>**4468.042**	0.242	283.086	0.802	2nd enantiomer

rac-**6a**: separation of enantiomers of pure major diastereomer

Work-up Procedures

The initial product of the reaction retains the BEt_2 group on the alcohol. The O-B bond is relatively stable towards hydrolysis but can be cleaved under oxidative, acidic or basic conditions as described below. This gives the desired product containing a free hydroxy group. In general, the oxidative work-up **A** is the most effective at cleaving the O–B bond and can be used when no oxidatively sensitive functionality (such as an amine) is present. The acidic work-up **B** generally also leads to complete O–B cleavage. The basic work-up **C** tends to be less efficient and a long period of stirring is typically required for complete conversion. It is therefore used only for substrates which are sensitive to acid and/or oxidants.

Oxidative work-up (A): The reaction mixture was cooled to 0 °C and pH 7 phosphate buffer solution (1 mL) and aq. H_2O_2 (30% in water, ~0.5 mL) were added. After stirring for 1 h at 0 °C, the mixture was diluted with ethyl acetate (10 mL) and water (10 mL) and transferred to a separating funnel. The organic layer was separated and the aqueous layer was extracted with ethyl acetate (2 × 5 mL). The combined organic layers were dried over anhydrous Na_2SO_4, filtered and concentrated *in vacuo*.

Acidic work-up (B): The reaction mixture was diluted with ethyl acetate (10 mL) and transferred to a separating funnel, then successively washed with HCl solution (2 M, 10 mL) (shake vigorously!), sat. aq. $NaHCO_3$ solution (10 mL) and brine (10 mL). The organic layer was dried over anhydrous Na_2SO_4, filtered and concentrated *in vacuo*.

Basic work-up (C): Sat. aq. $NaHCO_3$ solution (0.5 mL) was added to the reaction mixture and stirring was continued at rt for 1-5 h. The mixture was diluted with ethyl acetate (10 mL) and water (10 mL) and transferred to a separating funnel. The organic layer was separated and the aqueous layer was extracted with ethyl acetate (2 × 5 mL). The combined organic layers were dried over anhydrous Na_2SO_4, filtered and concentrated *in vacuo*.

In our hands, this reaction proved robust and reproducible. However, care should be taken to ensure the reaction is air-free and that all reaction components are of high purity. The following notes are provided to prevent potential issues and help prospective users obtain good results.

Notes on solvents and reagents:

1. THF used in the reaction was distilled over Mg/anthracene under argon. It is stored in a Schlenk flask under argon for long-term use.
2. The presence of residual chlorinated solvents ($CHCl_3$, CH_2Cl_2 etc.) in any reaction component will lead to catalyst decomposition.
3. Tris(*trans*-1,2-bis(4-*tert*-butylphenyl)ethene)nickel(0) ((Ni(*t*Bu-stb)$_3$)[2] can be purchased from Strem Chemicals (CAS: 2468315-70-8). Unlike Ni(cod)$_2$, this Ni(0) source can be

weighed out in air without any special precautions or decomposition. It is an orange-red solid and is stored in the freezer at –20 °C. It is weighed out after the container has warmed to room temperature. The same batch can be used for several months without any observable loss of catalytic activity.

The related tris(*trans*-1,2-bis(4-(trifluoromethyl)phenyl)ethene)nickel(0)[3] was found to be similarly effective in our catalytic reaction but was not investigated in detail due to the higher air-stability of the *tert*-butyl analogue.

4. Liquid aldehydes were distilled under vacuum and stored in the freezer (–20 °C) under argon in a crimp capped vial prior to use. Carboxylic acid impurities have a deleterious effect on reactivity and so the purity of aldehydes was checked by ^{1}H NMR spectroscopy after distillation, as well as before use in the reaction if they had been stored for a long time.
5. Solid aldehydes such as 3,5-dibromobenzaldehyde were typically used as received after checking purity by ^{1}H NMR spectroscopy. These compounds were stored on the bench at room temperature.
6. Triethylborane (1 M in THF) was stored in the freezer at –20 °C under argon. Although triethylborane itself is very pyrophoric and any solution of it must be handled carefully under oxygen-free conditions, we had no safety problems using the THF solution.
7. Methyl sorbate and ethyl sorbate were ordered from commercial suppliers, stored at room temperature and used as received. Commercial samples are rather viscous liquids that contain impurities when analysed by ^{1}H NMR spectroscopy; these do not appear to affect the reaction. However, batches stored for years may have undergone significant decomposition and can therefore perform worse in the reaction.
8. Other dienes (see section below) were purified by flash chromatography or distillation under vacuum and typically stored at –20 °C under argon in a crimp-capped vial to minimise decomposition.

Notes on reaction set-up and purification:

9. Although tris(*trans*-1,2-bis(4-*tert*-butylphenyl)ethene)nickel(0) is air-stable in the solid state, care should be taken once it has been dissolved in solvent as it will likely be more oxygen-sensitive. The ligand is always added before the solvent as it coordinates and helps to stabilise the Ni(0) catalyst if any residual oxygen is present in the solution. The diene also seems to help stabilise the nickel, while triethylborane will react with any excess oxygen. The addition of these two components soon after the solvent is therefore recommended.
10. The reaction is run in a sealed Schlenk flask rather than under a flow of argon due to the volatility of triethylborane (bp 95 °C). However, ethylene gas is given off from

reduction of the nickel catalyst with triethylborane over time, and care must therefore be taken to use reaction vessels which can withstand the increase in pressure caused by this gas evolution, especially if scaling up.

11. TLC run on silica gel plates using hexane/ethyl acetate mixtures (85:15 hexane/ethyl acetate is often an appropriate eluent for reactions of methyl sorbate with aldehydes not bearing strongly polar substituents) sometimes gave poor separation between the product spot and other spots. In these cases, toluene/*tert*-butyl methyl ether mixtures (95:5 toluene/*tert*-butyl methyl ether is often appropriate) generally gave markedly better separation.

Note on HPLC analysis:

12. The minor diastereomer could not be seen in the ^{1}H NMR spectrum for most compounds; the dr is therefore recorded simply as >20:1. However, it was often observed in a ≈1:50 ratio relative to the major diastereomer during HPLC analysis.
13. 2D HPLC analysis was sometimes employed to ensure accurate ee determination. This involved first running the isolated product down an achiral column to separate the diastereomers, before subsequent resolution of the pure major diastereomer using a column with a chiral stationary phase.

Typical reaction colours:

Nickel-catalysed reductive coupling reactions of this type have distinctive colours. Most reactions start out as dark red, red-brown or orange, but often become somewhat lighter over time. The wine-red colour of reaction of furfural immediately after aldehyde addition (left photo), and yellow-orange colour after 16 hours reaction time (complete conversion, right photo) are representative:

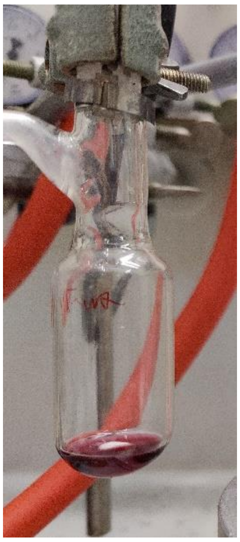

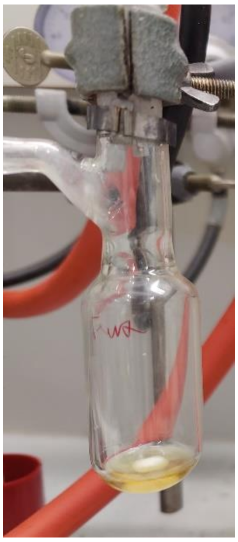

For reactions of substrates with coordinating functionality (such as a nitrile), the colour may be notably different. This reaction also became lighter after stirring overnight:

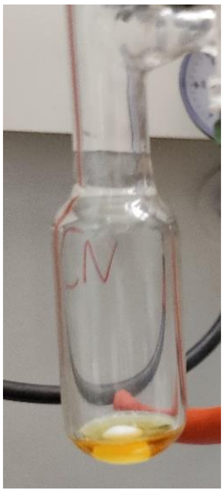

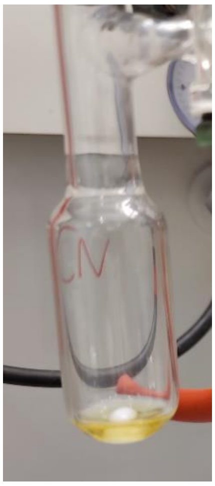

If the mixture goes colourless or transparent light yellow soon after addition of all components this is usually a sign that the catalyst is no longer active and the reaction has most likely failed (see next picture). This may happen if the starting materials are impure or oxygen has not been effectively excluded.

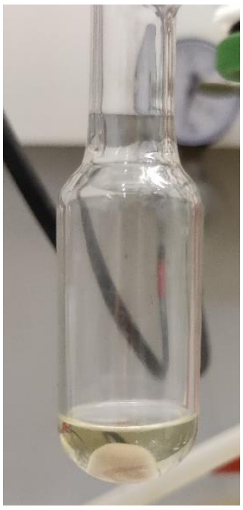

Limitations and Discussion of Results with Potential Mechanistic Implications

(1) Attempted reductive coupling of methyl sorbate (*E,E*-**5**) with the following aldehydes failed under the standard conditions described in the representative procedure, resulting in very low conversion or even no reaction:

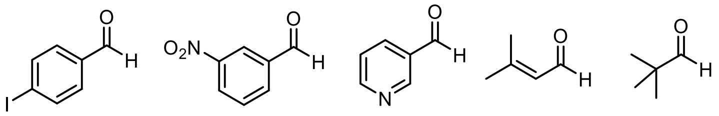

(2) Cyclic dienes such as 1,3-cyclohexadiene or 2-pyrone led to marginal conversion. This observation is taken as an indication that the diene must be able to bind to the nickel catalyst in an s-*trans* manner, as had already been proposed in the literature.[4,5] It cannot be ruled out, however, that competitive binding in the s-*cis* form takes place to give off-cycle intermediates.

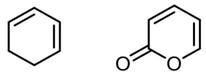

(3) As shown in Figure 1B of the main text, dienoic acid esters **5**, **S2** and **S5** reacted similarly well to give products **15a**-**c** in good yield and excellent stereoselectivity. Although this finding suggests that there is ample scope with regard to the terminus of the reacting dienoate, the corresponding phenyl-substituted derivative furnished a mixture of regioisomers; the markedly different ee's of the two isomers are noteworthy and currently unexplained.

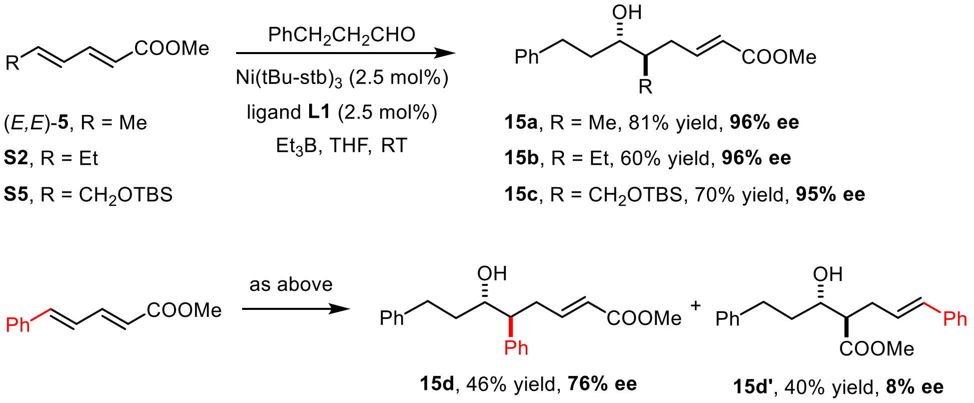

(4) Sorbate esters comprising unbranched alkoxy groups are excellent substrates, whereas the analogous *tert*-butyl ester gave a 1:1 mixture of regioisomers in low yield.

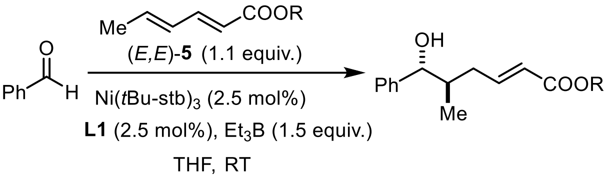

6a, R = Me, 90% yield, **95% ee**
6b, R = Et, 81% yield, **97% ee**

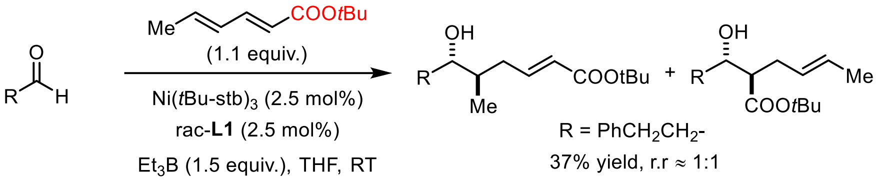

This experimental finding is thought to be of mechanistic relevance as the transition state passed through during the reactions following the "inverted" regiochemical course is obviously incapable of accommodating a bulky ester group. The tentative stereochemical model **A** proposed in the main text accounts for this result, because the ester –OR substituent resides directly above an aromatic wing of the chiral ligand; if –OR is large, it clashes with the π-system and disrupts the array. This size-effect speaks against an otherwise conceivable alternative model **A'**, in which the ester points away from the ligand backbone and variations should therefore have comparatively little impact on regioselectivity and yield.

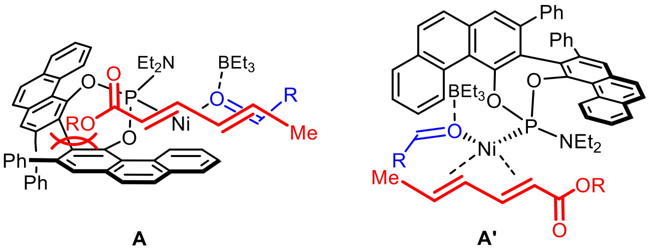

A **A'**

(5) The currently favored model **A** also explains why changing the configuration of the reacting distal double bond switches the product stereochemistry from *anti* to *syn* while the sense and approximate level of induction are maintained.

(6) The results obtained with the VAPOL-derived phosphoramidite **L1** differ from those obtained with the spirocyclic phosphoramidite **L2** as reported in the literature (see also Scheme 1B, main text).[6] Importantly, the difference is most likely not the result of the different amide substituents: our previous investigations had shown that changing the –NEt_2 group in **L1** for a morpholineamide has little impact on selectivity and yield (see the SI of ref.[1]). Since **L1** and **L2** are of very similar electronic character, the deep cleft of **L1** seems to be a critical determinant. We tentatively ascribe the "inverse"

regiochemical course enforced by **L1** to secondary effects between the incoming 1,3-diene and the extended π-system of the ligand backbone.[7,8]

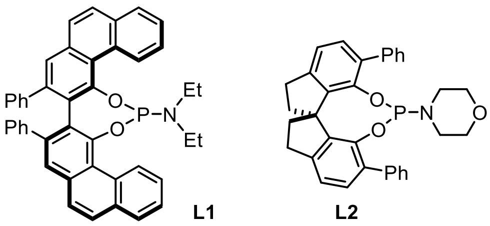

(7) The notion that a deep binding site is decisive for the "inverse" coupling of sorbate esters and related substrates to proceed is supported by the complete lack of reactivity in the attempted reaction of benzaldehyde with *E,E*-**5** using an otherwise closely related BINOL-derived phosphoramidite ligand.

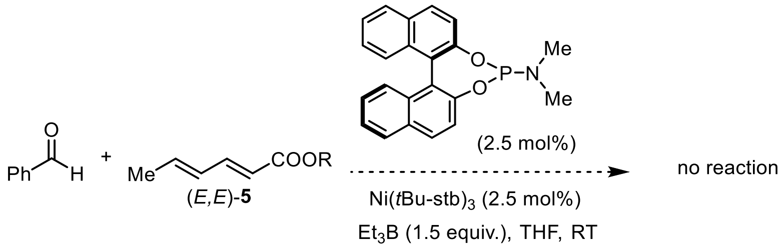

(8) Formal replacement of –NEt_3 by –OiPr or –Ph proved detrimental for the yield and the ee of reactions of this type. Therefore it cannot be excluded that the –NEt_2 group of **L1** also plays a role in positioning the reaction partners within the chiral pocket such that "inverse" coupling will ensue. Since **L1** proved optimal for electron-deficient sorbate esters (this work) as well as for electron-rich dienyl ethers (ref.[1]), direct contacts with the respective polar groups of these substrates seem unlikely. However, one might conceive of transient secondary interactions of the [**L1**Ni] catalyst complex with Et_3B, which in turn could determine the coordination site at which the aldehyde binds to the nickel catalyst and eventually gets activated by Et_3B.

(9) Any attempt at studying the reaction by NMR faces a number of serious challenges:

- Even [D_8]-THF is a competitive ligand able to replace one or more stilbene units from the precatalyst [Ni(*t*Bu-stb)$_3$]. At –78°C, the major complex in solution is believed to be [Ni(*t*Bu-stb)$_2$([D_8]-THF)];[2] at RT, at which most reductive coupling reactions of sorbate esters are performed, the solution of the precatalyst comprises more than one species of unknown composition.

- Upon addition of **L1** (1 equivalent relative to [Ni]), at least three phosphorous-containing species are detected in addition to unbound ligand; line broadening indicates dynamic processes.
- When 1 equivalent of methyl sorbate is added to this mixture, all peaks in the ^{1}H and ^{31}P NMR spectra become extremely broad and almost featureless, rendering an accurate structure assignment impossible.
- Benzaldehyde competes with phosphoramidite **L1** for the binding sites at nickel. Upon its addition (1 equiv.), ≈50% of originally ligated **L1** is released into the solution, which implies that substantial amounts of one or more phosphorous-free nickel species of unknown constitution must also be present, which may or may not constitute an off-cycle reservoir.
- For the various exchange processes and the massive line broadening, the speciation of a truly catalytic set-up, in which sorbate and benzaldehyde are present in large excess relative to [Ni] and **L1**, at least in the early phases of the reaction, is even less amenable to NMR spectroscopic investigation.

Larger Scale Reaction. Methyl (5*R*,6*R*,*E*)-6-(3,5-dibromophenyl)-6-hydroxy-5-methylhex-2-enoate (14).

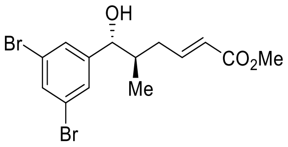

Tris(*trans*-1,2-bis(4-*tert*-butylphenyl)ethene)nickel(0) (Ni(*t*Bu-stb)$_3$) (23.4 mg, 0.025 mmol, 2.5 mol%)[2] and phosphoramidite **L1** (16.0 mg, 0.025 mmol, 2.5 mol%) were added to a flame-dried thick-walled Schlenk flask (see Note 9) under argon and dissolved in THF (2.0 mL). Methyl sorbate *E,E*-**5** (143 µL, 0.32 mmol) and triethylborane (1 M in THF, 1.50 mL, 1.50 mmol) were added, followed by 3,5-dibromobenzaldehyde (264 mg, 1.00 mmol). The flask was sealed under argon and the mixture was stirred at room temperature for 16 h. The mixture was cooled to 0 °C and pH 7 phosphate buffer solution (3 mL) and aq. H_2O_2 (30% in water, ~1.5 mL) were added slowly (Note: bubbling is observed when the reaction is quenched – addition of aqueous solutions should be performed carefully). After stirring for 1 h at 0 °C, the mixture was diluted with ethyl acetate (10 mL) and water (20 mL) and transferred to a separating funnel. The organic layer was separated and the aqueous phase was extracted with ethyl acetate (2 × 10 mL). The combined organic layers were washed with sat. aq. $Na_2S_2O_3$ solution and brine and then dried over anhydrous Na_2SO_4, filtered and concentrated *in vacuo* to give a yellow oil. Purification by flash chromatography (SiO_2, hexane/ethyl acetate 9:1 to 85:15) gave the title compound as a light yellow oil (346 mg, 88% yield, >20:1 dr, 88% ee).

For the analytical and spectral data, see below.

The ee was determined by HPLC analysis: Chiralpak 150 mm IC-3, 4.6 mm i.D., *n*-heptane/isopropanol = 95:5, v = 1.0 mL/min, λ = 220 nm, t(major) = 6.79 min, t(minor) = 9.28 min.

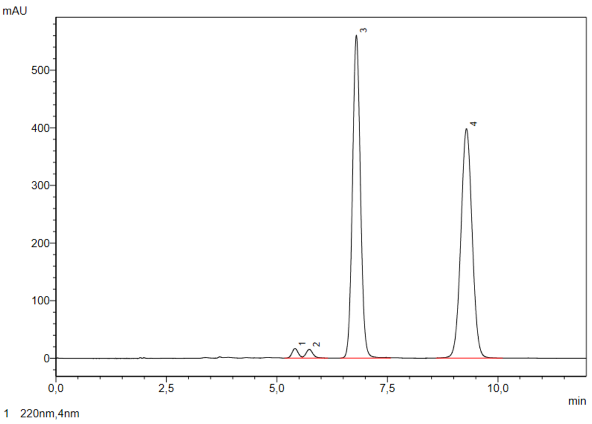

PDA Ch1 220nm

Peak #	Ret. Time	Area %	Name
1	5,41	1,12	
2	5,73	1,15	
3	6,80	48,75	1. Enantiomer
4	9,29	48,98	2. Enantiomer
Total		100,00	

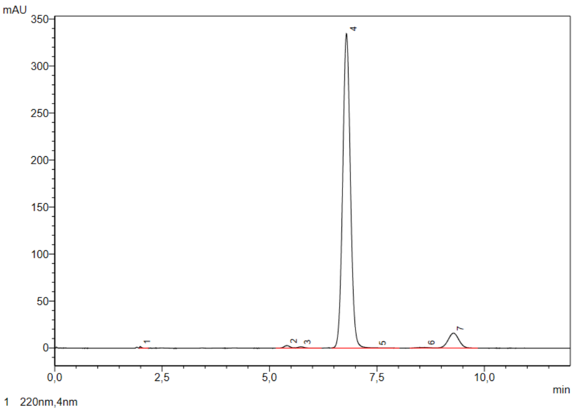

PDA Ch1 220nm

Peak #	Ret. Time	Area %	Name
1	1,99	0,11	
2	5,40	0,57	
3	5,72	0,30	
4	6,79	92,52	1. Enantiomer 87.6 % ee
5	7,47	0,12	
6	8,61	0,25	
7	9,28	6,13	2. Enantiomer
Total		100,00	

Ethyl (5*R*,6*R*,*E*)-6-hydroxy-5-methyl-6-phenylhex-2-enoate (6b). Prepared analogously according to the representative procedure followed by an oxidative work-up. Purified by flash chromatography (SiO_2, hexane/ethyl acetate 9:1 to 84:16) to give the title compound as a colourless oil (58 mg, 81% yield, >20:1 dr, 97% ee).

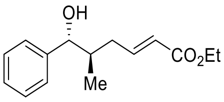

$[\alpha]_D^{25}$ = +14.8 (c = 1.2, $CHCl_3$) ($[\alpha]_D^{25}$ of the antipode: –11.8);[9] ^{1}H NMR (400 MHz, $CDCl_3$) δ = 7.37-7.25 (m, 5H), 6.97 (ddd, *J* = 15.5, 8.5, 6.5 Hz, 1H), 5.85 (dt, *J* = 15.5, 1.5 Hz, 1H), 4.42 (d, *J* = 7.5 Hz, 1H), 4.17 (q, *J* = 7.0 Hz, 2H), 2.56 (dddd, *J* = 14.0, 6.5, 4.0, 1.5 Hz, 1H), 2.15 (app. dtd, *J* = 14.0, 8.5, 1.5 Hz, 1H), 2.02 (app. dddd, *J* = 11.0, 9.0, 7.0, 4.0 Hz, 1H), 1.92 (br. s, 1H, –OH), 1.28 (t, *J* = 7.0 Hz, 3H), 0.77 (d, *J* = 7.0 Hz, 3H); ^{13}C NMR (101 MHz, $CDCl_3$) δ = 166.7, 148.0, 143.2, 128.5, 127.9, 126.7, 122.9, 78.5, 60.3, 39.8, 35.3, 16.1, 14.4; IR (ATR): ṽ = 3465, 2977, 2933, 1715, 1698, 1650, 1453, 1369, 1311, 1268, 1169, 1039, 981, 762, 700; HRMS (EI, *m*/*z*) calculated for $[C_{15}H_{20}O_3]^+$ ($[M^+]$) 248.1409, found 248.1407.

The ee was determined by HPLC analysis: Chiralcel 150 mm OD-3, 4.6 mm i.D., *n*-heptane/isopropanol = 90:10, v = 1.0 mL/min, λ = 220 nm, t(minor) =4.94 min, t(major) = 6.81 min.

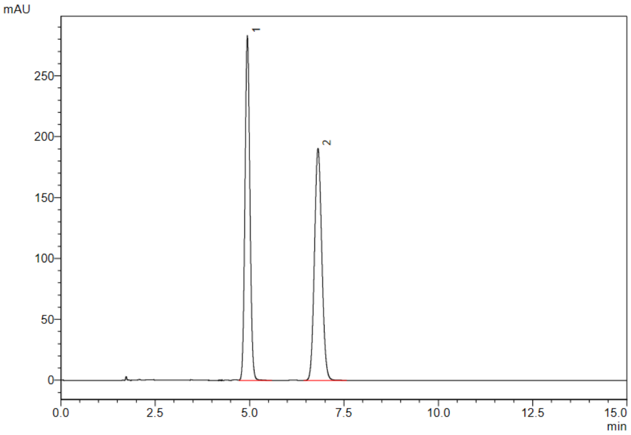

1 220nm,4nm

PDA Ch1 220nm

Peak #	Ret. Time	Area %	Name
1	4.94	49.87	1. Enantiomer
2	6.81	50.13	2. Enantiomer
Total		100.00	

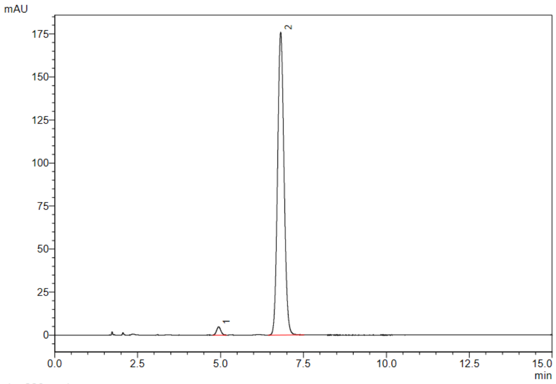

1 220nm,4nm

PDA Ch1 220nm

Peak #	Ret. Time	Area %	Name
1	4.94	1.80	1. Enantiomer
2	6.81	98.20	2. Enantiomer
Total		100.00	

ee = 96,6 %

Methyl (5*S*,6*R*,*E*)-6-hydroxy-5-methyl-6-phenylhex-2-enoate (7a). Prepared analogously according to the representative procedure (except that the reaction mixture was stirred at –40 °C for 3 d) followed by an oxidative work-up. Purified by flash chromatography (SiO_2, hexane/ethyl acetate 30:1 to 6:1) to give the title compound as a colorless oil (64 mg, 94% yield, >20:1 dr, 86% ee).

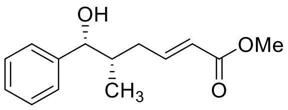

$[\alpha]_D^{20}$ = –1.0 (c = 0.5, $CHCl_3$); ^1H NMR (400 MHz, $CDCl_3$) δ 7.38-7.26 (m, 5H), 6.93 (ddd, *J* = 15.6, 7.6, 6.9 Hz, 1H), 5.80 (dt, *J* = 15.6, 1.4 Hz, 1H), 4.57 (d, *J* = 5.1 Hz, 1H), 3.72 (s, 3H), 2.39-2.25 (m, 1H), 2.05-1.94 (m, 2H), 1.83 (br s, 1H), 0.94 (d, *J* = 6.6 Hz, 2H); ^{13}C NMR (101 MHz, $CDCl_3$) δ 167.1, 148.1, 143.2, 128.5, 127.8, 126.5, 122.5, 77.6, 51.6, 39.9, 36.2, 14.6; IR (ATR): ṽ = 3467, 3025, 2953, 2906, 1705, 1654, 1493, 1437, 1314, 1275, 1217, 1169, 1109, 1043, 980, 750, 701, 666; HRMS (ESI^+, *m/z*) calculated for $[C_{14}H_{18}O_3]^+$ ($[M^+]$) 234.1250, found 234.1251.

The ee was determined by HPLC analysis: Chiralpak 150 mm IB-N-3, 4.6 mm i.D., *n*-heptane/*i*-propanol = 95:5, v = 1.0 mL/min, λ = 220 nm, t(minor) = 8.45 min, t(major) = 11.88 min.

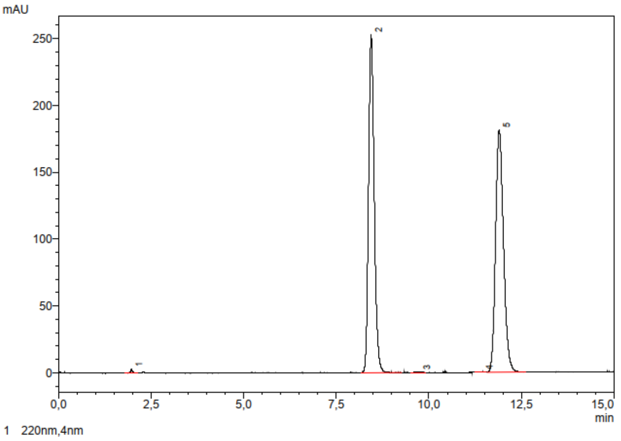

PDA Ch1 220nm

Peak #	Ret. Time	Area %	Name
1	1,97	0,23	
2	8,44	49,73	1. Enantiomer Diastereomer 1
3	9,75	0,15	1. Enantiomer Diastereomer 2
4	11,43	0,13	2. Enantiomer Diastereomer 2
5	11,90	49,76	2. Enantiomer Diastereomer 1
Total		100,00	

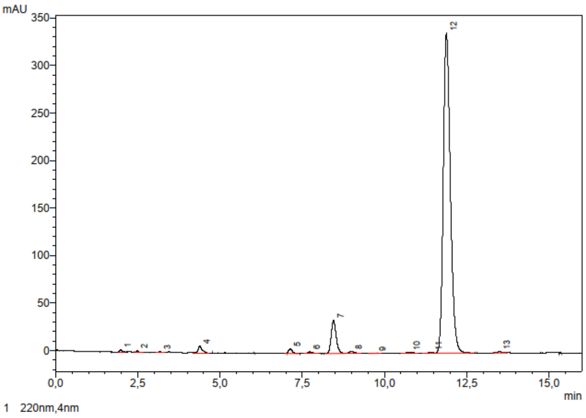

PDA Ch1 220nm

Peak #	Ret. Time	Area %	Name
1	1,97	0,20	
2	2,48	0,10	
3	3,18	0,14	
4	4,38	1,07	
5	7,13	0,72	
6	7,73	0,34	
7	8,45	6,53	1. Enantiomer Diastereomer 1
8	9,00	0,46	
9	9,72	0,05	1. Enantiomer Diastereomer 2
10	10,76	0,27	
11	11,43	0,17	2. Enantiomer Diastereomer 2
12	11,88	89,60	2. Enantiomer Diastereomer 1 86.4 % ee
13	13,50	0,36	
Total		100,00	

Methyl (5*S*,6*S*,*E*)-6-hydroxy-5-methyl-8-phenyloct-2-enoate (7b). Prepared analogously according to the representative procedure (except that the mixture was stirred at 0 °C overnight) followed by an oxidative work-up. Purified by flash chromatography (SiO_2, hexane/ethyl acetate 40:1 to 8:1) to give the title compound as a yellow oil (63 mg, 83% yield, >20:1 dr, 89% ee).

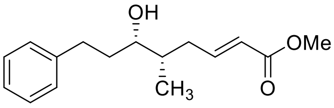

$[\alpha]_D^{20}$ = –48.4 (c = 0.5, $CHCl_3$); ^{1}H NMR (400 MHz, $CDCl_3$) δ 7.34-7.24 (m, 2H), 7.23-7.15 (m, 3H), 6.95 (ddd, *J* = 15.3, 7.8, 7.2 Hz, 1H), 5.85 (dt, *J* = 15.6, 1.5 Hz, 1H), 3.73 (s, 3H), 3.57 (ddd, *J* = 7.2, 5.8, 3.7 Hz, 1H), 2.82 (dt, *J* = 13.6, 7.7 Hz, 1H), 2.65 (dt, *J* = 13.7, 8.0 Hz, 1H), 2.38 (dddd, *J* = 14.3, 7.2, 5.6, 1.6 Hz, 1H), 2.10 (app. dtd, *J* = 14.1, 8.0, 1.4 Hz, 1H), 1.80 – 1.70 (m, 2H), 0.91 (d, *J* = 6.8 Hz, 3H). ^{13}C NMR (101 MHz, $CDCl_3$) δ 167.1, 148.3, 142.0, 128.6, 128.5, 126.1, 122.4, 74.1, 51.6, 38.2, 36.4, 36.4, 32.8, 13.6; IR (ATR): ṽ = 3435, 3026, 2949, 1704, 1654, 1495, 1454, 1436, 1314, 1270, 1218, 1171, 1113, 1040, 980, 921, 847, 749, 698, 666; HRMS (ESI+, *m/z*) calculated for $[C_{16}H_{22}O_3+Na]^+$ ($[M+Na]^+$) 285.1461, found 285.1458.

The ee was determined by HPLC analysis: Chiralpak 150 mm IA-3, 4.6 mm i.D., acetonitrile/water = 45:55, v = 1.0 mL/min, λ = 220 nm, t(major) = 18.39 min, t(minor) = 20.29 min.

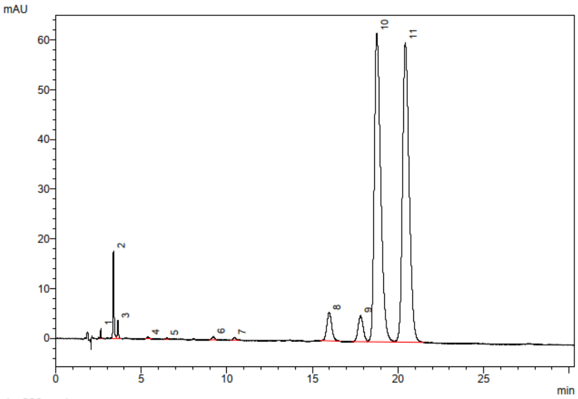

1 220nm,4nm

PDA Ch1 220nm

Peak #	Ret. Time	Area %	Name
1	2.64	0.15	
2	3.37	1.98	
3	3.63	0.41	
4	5.39	0.09	
5	6.50	0.04	
6	9.22	0.19	
7	10.44	0.16	
8	15.98	3.19	1st diastereomer, 1st enantiomer
9	17.81	3.03	1st diastereomer, 2nd enantiomer
10	18.76	45.35	2nd diastereomer, 1st enantiomer
11	20.43	45.40	2nd diastereomer, 2nd enantiomer
Total		100.00	

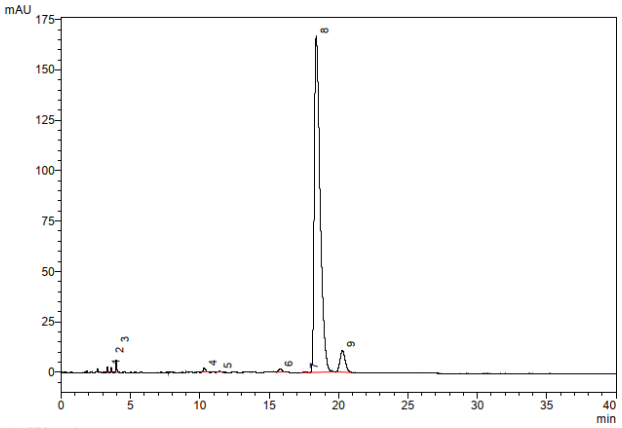

1 220nm,4nm

PDA Ch1 220nm

Peak #	Ret. Time	Area %	Name
1	3.35	0.18	
2	3.61	0.16	
3	3.99	0.52	
4	10.33	0.48	
5	11.41	0.12	
6	15.81	0.47	1. diastereomer 1. enantiomer
7	17.62	0.18	1. diastereomer 2. enantiomer
8	18.39	92.41	2. diastereomer 1. enantiomer
9	20.29	5.47	2. diastereomer 2. enantiomer
Total		100.00	

ee 1 = 44.6%

ee 2 = 88.8%

Methyl 4-((1*R*,2*R*,*E*)-1-hydroxy-6-methoxy-2-methyl-6-oxohex-4-en-1-yl)benzoate (8a).

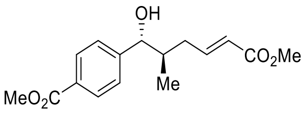

Prepared analogously according to representative procedure followed by an oxidative work-up. Purified by flash chromatography (SiO_2, hexane/ethyl acetate 6:1 to 4:1) to give the title compound as a light yellow oil (71 mg, 84% yield, >20:1 dr, 96% ee).

$[\alpha]_D^{20}$ = –1.4 (c = 0.5, $CHCl_3$); ^{1}H NMR (400 MHz, $CDCl_3$) δ = 7.97 (d, *J* = 8.5 Hz, 2H), 7.36 (d, *J* = 8.5 Hz, 2H), 6.93 (ddd, *J* = 15.5, 8.5, 7.0 Hz, 1H), 5.82 (dt, *J* = 15.5, 1.5 Hz, 1H), 4.49 (d, *J* = 7.0 Hz, 1H), 3.89 (s, 3H), 3.69 (s, 3H), 2.48 (dddd, *J* = 14.0, 7.0, 4.0, 1.5 Hz, 1H), 2.14 (dtd, *J* = 14.0, 8.5, 1.5 Hz, 1H), 2.03-1.94 (m, 1H), 0.76 (d, *J* = 7.0 Hz, 3H); ^{13}C NMR (101 MHz, $CDCl_3$) δ = 167.1, 167.0, 148.4, 148.0, 129.8, 129.5, 126.7, 122.6, 77.9, 52.2, 51.6, 39.8, 35.0, 16.0; IR (ATR): ṽ = 3488, 2954, 1719, 1654, 1611, 1436, 1276, 1174, 1111, 1040, 1018, 982, 774, 711; HRMS (CI, *m*/*z*) calculated for $[C_{16}H_{21}O_5]^+$ ($[M+H]^+$) 293.1386, found 293.1384.

The ee was determined by HPLC analysis: Chiralcel 150 mm OJ-3R, 4.6 mm i.D., methanol/water = 85:15, v = 1.0 mL/min, λ = 220 nm, t(minor) = 5.68 min, t(major) = 6.12 min.

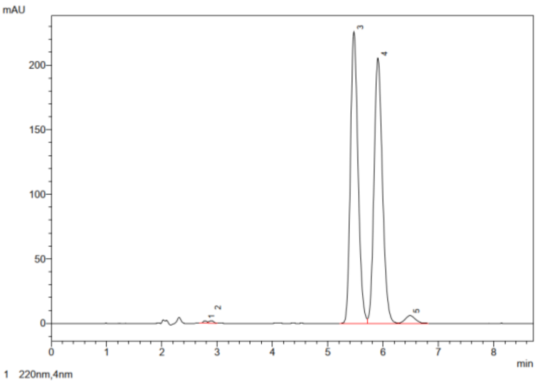

PDA Ch1 220nm

Peak #	Ret. Time	Area %	Name
1	2,78	0,20	
2	2,90	0,26	
3	5,47	48,90	1. Enantiomer
4	5,91	48,95	2. Enantiomer
5	6,49	1,68	
Total		100,00	

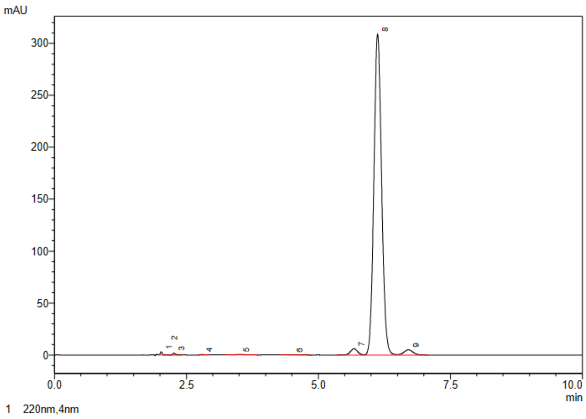

PDA Ch1 220nm

Peak #	Ret. Time	Area %	Name
1	2.02	0.23	
2	2.13	0.01	
3	2.27	0.20	
4	2.79	0.05	
5	3.49	0.16	
6	4.50	0.13	
7	5.68	1.74	1. enantiomer
8	6.12	95.65	2. enantiomer
9	6.71	1.83	
Total		100.00	

ee = 96.4%

Methyl (5*R*,6*R*,*E*)-6-(4-cyanophenyl)-6-hydroxy-5-methylhex-2-enoate (8b). Prepared analogously according to the representative procedure followed by an oxidative work-up. Purified by flash chromatography (SiO_2, hexane/ethyl acetate 4:1 to 7:3) to give the title compound as a light yellow oil (59 mg, 78% yield, >20:1 dr, 93% ee).

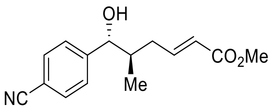

$[\alpha]_D^{20}$ = –1.6 (c = 0.54, $CHCl_3$); ^{1}H NMR (400 MHz, $CDCl_3$) δ = 7.59 (m, 2H), 7.39-7.35 (m, 2H), 6.86 (ddd, *J* = 15.5, 8.5, 7.0 Hz, 1H), 5.77 (dt, *J* = 15.5, 1.5 Hz, 1H), 4.47 (d, *J* = 6.5 Hz, 1H), 3.66 (s, 3H), 2.38 (dddd, *J* = 14.5, 6.5, 4.0, 1.5 Hz, 1H), 2.10 (dtd, *J* = 14.5, 8.5, 1.5 Hz, 1H), 1.92 (dddd, *J* = 13.5, 8.5, 7.0, 4.0 Hz, 1H), 0.74 (d, *J* = 7.0 Hz, 3H); ^{13}C NMR (101 MHz, $CDCl_3$) δ = 167.0, 148.5, 147.5, 132.3, 127.4, 122.9, 118.8, 111.1, 77.6, 51.6, 39.9, 34.8, 16.1; IR (ATR): ṽ = 3477, 2956, 2228, 1701, 1653, 1436, 1317, 1273, 1168, 1036, 981, 832, 573; HRMS (EI, *m*/*z*) calculated for $[C_{15}H_{17}NO_3]^+$ ($[M^+]$) 259.1205, found 259.1203.

The ee was determined by HPLC analysis: Chiralpak 150 mm IA-3, 4.6 mm i.D., *n*-heptane/isopropanol = 95:5, v = 1.0 mL/min, λ = 230 nm, t(major) = 20.81 min, t(minor) = 22.94 min.

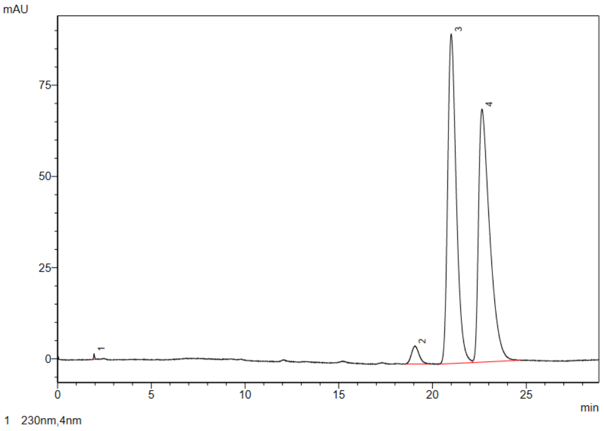

PDA Ch1 230nm

Peak #	Ret. Time	Area %	Name
1	1,94	0,10	
2	19,07	2,36	
3	21,00	48,82	1. Enantiomer
4	22,64	48,71	2. Enantiomer
Total		100,00	

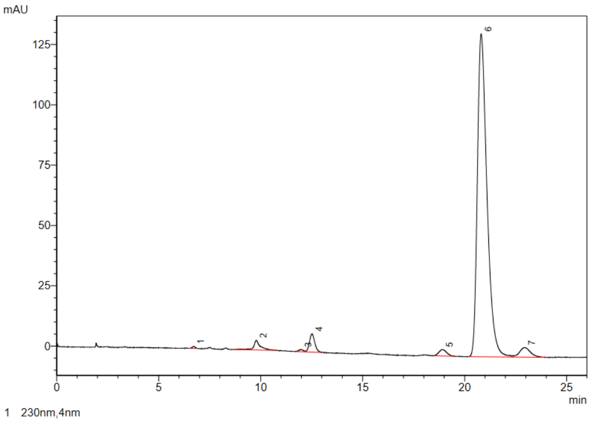

PDA Ch1 230nm

Peak #	Ret. Time	Area %	Name
1	6,71	0,17	
2	9,78	1,93	
3	11,97	0,28	
4	12,52	2,82	
5	18,91	1,45	
6	20,81	90,25	1. Enantiomer 93.3 % ee
7	22,94	3,11	2. Enantiomer
Total		100,00	

Methyl (5*S*,6*S*,*E*)-6-hydroxy-5-methyl-6-(4-(4,4,5,5-tetramethyl-1,3,2-dioxaborolan-2-yl)phenyl)hex-2-enoate (8c). Prepared analogously according to the representative procedure followed by a basic work-up (stirring with sat. aq. $NaHCO_3$ at 0 °C). Purified by flash chromatography (SiO_2, hexane/ethyl acetate 4:1 to 7:3) to give the title compound as a colourless oil (86 mg, 82% yield, >20:1 dr, 97% ee).

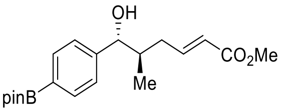

$[\alpha]_D^{25}$ = 9.4 (c = 0.53, $CHCl_3$); ^{1}H NMR (400 MHz, $CDCl_3$) δ = 7.79 (d, *J* = 8.0 Hz, 2H), 7.31 (d, *J* = 8.0 Hz, 2H), 6.97 (ddd, *J* = 15.5, 8.5, 6.5 Hz, 1H), 5.85 (dt, *J* = 15.5, 1.5 Hz, 1H), 4.44 (d, *J* = 7.0 Hz, 1H), 3.72 (s, 3H), 2.53 (dddd, *J* = 14.0, 6.5, 4.0, 1.5 Hz, 1H), 2.15 (app. dtd, *J* = 14.0, 8.5, 1.5 Hz, 1H), 2.05-1.96 (m, 1H), 1.88 (br. s, 1H, –OH), 1.34 (s, 12H), 0.76 (d, *J* = 7.0 Hz, 3H); ^{13}C NMR (101 MHz, $CDCl_3$) δ = 167.1, 148.3, 146.3, 135.0, 126.1, 122.6, 84.0, 78.4, 51.5, 39.8, 35.2, 25.0, 16.1 (one sp^2 carbon missing due to overlap); IR (ATR): ṽ = 3647, 2977, 1721, 1654, 1621, 1358, 1318, 1269, 1087, 1019, 858, 659; HRMS (EI, *m*/*z*) calculated for $[C_{20}H_{29}O_5B]^+$ ($[M^+]$) 360.2105, found 360.2103.

The ee was determined by HPLC analysis: Chiralcel 150 mm OJ-3R, 4.6 mm i.D., acetonitrile/water = 50:50, v = 1.0 mL/min, λ = 220 nm, t(major) = 7.68 min, t(minor) = 16.68 min.

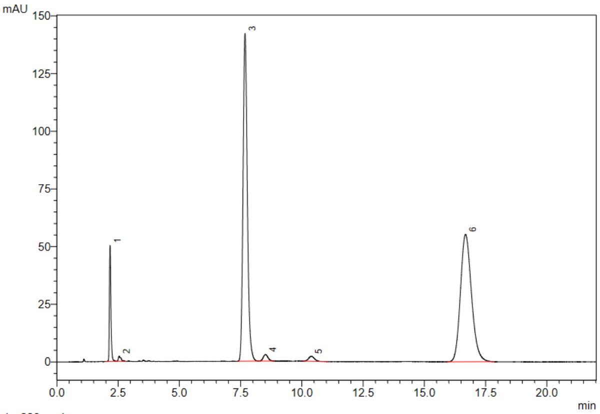

1 220nm,4nm

PDA Ch1 220nm

Peak #	Ret. Time	Area %	Name
1	2.17	5.73	
2	2.54	0.46	
3	7.68	45.90	1.Diastereomer, 1.Enantiomer
4	8.52	1.04	2.Diastereomer, 1.Enantiomer
5	10.39	0.96	2.Diastereomer, 2.Enantiomer
6	16.68	45.90	1.Diastereomer, 2.Enantiomer
Total		100.00	

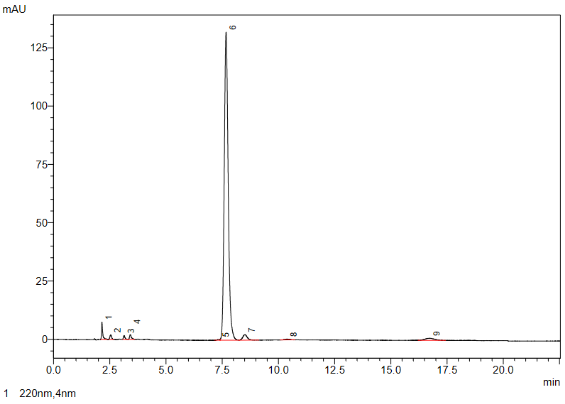

1 220nm,4nm

PDA Ch1 220nm

Peak #	Ret. Time	Area %	Name
1	2.15	1.56	
2	2.54	0.58	
3	3.14	0.43	
4	3.42	0.66	
5	7.36	0.15	
6	7.67	92.93	1.Diastereomer, 1.Enantiomer
7	8.51	1.87	2.Diastereomer, 1.Enantiomer
8	10.37	0.31	2.Diastereomer, 2.Enantiomer
9	16.74	1.51	1.Diastereomer, 2.Enantiomer
Total		100.00	

ee = 96.8 %

Methyl (5*R*,6*R*,*E*)-6-hydroxy-5-methyl-6-(4-(trifluoromethyl)phenyl)hex-2-enoate (8d).

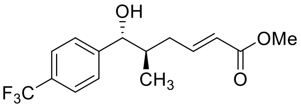

Prepared analogously according to the representative procedure followed by an oxidative work-up. Purified by flash chromatography (SiO_2, hexane/ethyl acetate 25:1 to 5:1) to give the title compound as a colorless oil (80 mg, 91% yield, >20:1 dr, 92% ee).

$[\alpha]_D^{20}$ = –11.2 (c = 0.52, $CHCl_3$); ^{1}H NMR (300 MHz, $CDCl_3$) δ 7.62-7.52 (m, 2H), 7.49-7.38 (m, 2H), 6.93 (ddd, *J* = 15.6, 8.2, 6.7 Hz, 1H), 5.83 (dt, *J* = 15.6, 1.5 Hz, 1H), 4.50 (d, *J* = 6.9 Hz, 1H), 3.70 (s, 3H), 2.48 (dddd, *J* = 14.1, 6.6, 4.0, 1.7 Hz, 1H), 2.34 (s, 1H), 2.15 (app. dtd, *J* = 14.0, 8.4, 1.3 Hz, 1H), 2.09-1.90 (m, 1H), 0.79 (d, *J* = 6.8 Hz, 3H); ^{13}C NMR (75 MHz, $CDCl_3$) δ 167.1, 147.9, 147.3, 130.0 (q, $^2J_{CF}$ = 32.3 Hz), 127.0, 125.4 (q, $^3J_{CF}$ = 3.8 Hz), 124.2 (q, $^1J_{CF}$ = 272.0 Hz), 122.7, 77.7, 51.6, 39.9, 34.9, 16.1; ^{19}F NMR (282 MHz, $CDCl_3$) δ –62.5; IR (ATR): ṽ = 3391, 2945, 2897, 1716, 1651, 1619, 1435, 1394, 1356, 1337, 1322, 1281, 1252, 1194, 1177, 1157, 1144, 1114, 1070, 1032, 1014, 991, 922, 901, 836, 814, 763, 745, 719; HRMS (ESI^+, *m*/*z*) calculated for $[C_{15}H_{17}F_3O_3+Na]^+$ ($[M+Na]^+$) 325.1022, found 325.1023.

The ee was determined by HPLC analysis: Chiralcel 150 mm OZ-3R, 4.6 mm i.D., acetonitrile/water = 35:65, v = 1.0 mL/min, λ = 220 nm, t(minor) = 20.73 min, t(major) = 23.41 min.

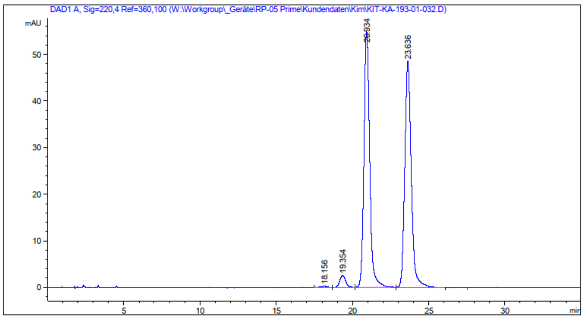

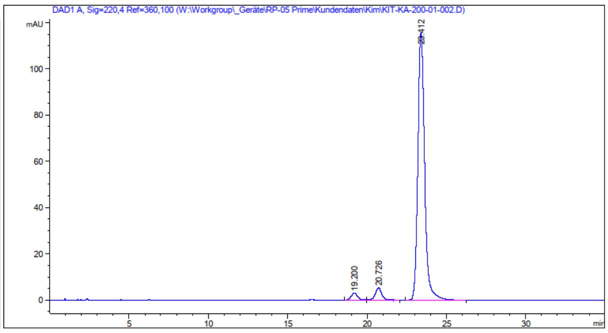

Signal 1: DAD1 A, Sig=220,4 Ref=360,100

Peak #	RetTime [min]	Type	Width [min]	Area [mAU*s]	Height [mAU]	Area %	
1	18.156	BB	0.3971	7.79553	2.86825e-1	0.2636	
2	19.354	BV	0.4378	71.62704	2.56483	2.4217	minor diastereomer
3	20.934	VB	0.4022	1441.63184	54.93133	48.7408	major diastereomer, 1st enantiomer
4	23.636	BB	0.4570	1436.69763	48.58456	48.5740	major diastereomer, 2nd enantiomer
Totals :				2957.75205	106.36755		

Signal 1: DAD1 A, Sig=220,4 Ref=360,100

Peak #	RetTime [min]	Type	Width [min]	Area [mAU*s]	Height [mAU]	Area %	
1	19.200	BV	0.4631	85.03361	2.96007	2.3153	minor diastereomer
2	20.726	VB	0.4977	149.84795	5.17762	4.0801	major diastereomer, 1st enantiomer
3	23.412	BB	0.5126	3437.80225	113.74638	93.6046	major diastereomer, 2nd enantiomer
Totals :				3672.68380	121.88407		

ee = 91.6 %

Methyl (5*R*,6*R*,*E*)-6-(4-(dimethylamino)phenyl)-6-hydroxy-5-methylhex-2-enoate (8e).

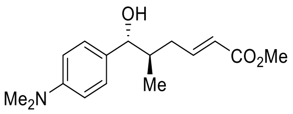

Prepared analogously according to the representative procedure followed by an basic work-up. Purified by flash chromatography (SiO_2, hexane/ethyl acetate 85:15 to 3:1) to give the title compound as a light yellow oil (68 mg, 85% yield, >20:1 dr, 90% ee).

$[\alpha]_D^{20}$ = 26.6 (c = 0.5, $CHCl_3$); ^{1}H NMR (400 MHz, $CDCl_3$) δ = 7.20-7.15 (m, 2H), 7.02 (ddd, *J* = 15.5, 8.5, 6.5 Hz, 1H), 6.73-6.68 (m, 2H), 5.87 (dt, *J* = 15.5, 1.5 Hz, 1H), 4.30 (d, *J* = 7.5 Hz, 1H), 3.73 (s, 3H), 2.95 (s, 6H), 2.61 (dddd, *J* = 14.0, 6.5, 4.0, 1.5 Hz, 1H), 2.15 (app. dtd, *J* = 14.0, 8.5, 1.5 Hz, 1H), 2.06-1.95 (m, 1H), 1.76 (br. s, 1H, –OH), 0.73 (d, *J* = 7.0 Hz, 3H); ^{13}C NMR (101 MHz, $CDCl_3$) δ = 167.2, 150.4, 148.7, 131.0, 127.6, 122.4, 112.5, 78.3, 51.6, 40.8, 39.7, 35.8, 16.1; IR (ATR): ṽ = 3326, 2954, 2878, 2851, 1715, 1611, 1517, 1309, 1162, 1021, 983, 815; HRMS (ESI, *m*/*z*) calculated for $[C_{16}H_{24}NO_3]^+$ ($[M+H]^+$) 278.1748, found 278.1571.

The ee was determined by 2D HPLC analysis

Step 1: Purification: 50 mm Eclipse Plus C18, 4.6 mm i.D., methanol/water 60:40, v = 1.0 mL/min, λ = 220 nm, t(major) = 3.24 min, 308 K.

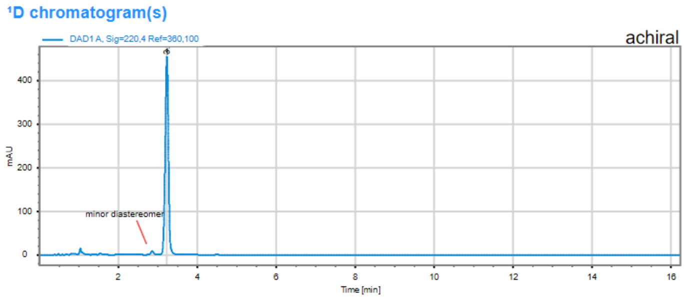

Sampling table (¹D)

Cut group	Cut #	¹D Cut start [min]	¹D Ret. time [min]	¹D Duration [min]	Trigger	²D Run start [min]	
	1	3.20	3.238	0.04	Peak	3.25	major diastereomer

8e: separation of impurities on achiral column

Step 2: Chiral resolution of the major diastereomer: Chiralcel 150 mm OJ-3R, 4.6 mm i.D., acetonitrile/water = 50:50, v = 1.0 mL/min, λ = 220 nm, t(major) = 5.64 min, t(minor) = 6.30 min.

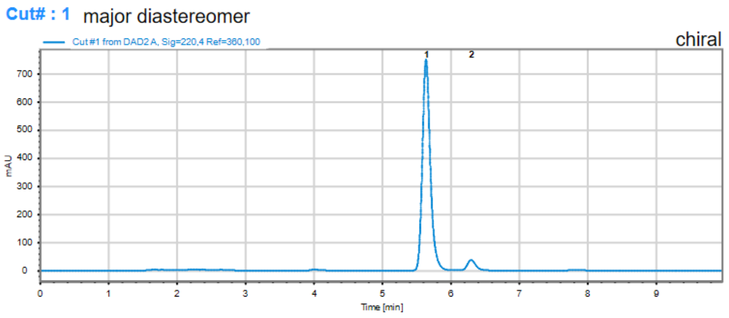

Signal: DAD2 A, Sig=220,4 Ref=360,100

Compound	Cut	Ret.Time	Area	Width	Height	Symmetry	
1	1	5.641	6045.066<br>**6045.066**	0.122	750.469	0.786	1st enantiomer
2	1	6.301	333.063<br>**333.063**	0.136	37.391	0.813	2nd enantiomer

ee = 89.6 %

Component table

Signal: DAD2 A, Sig=220,4 Ref=360,100

Component	¹D Sampling range [min]	Ret.Time ²D [min]	Area	Area%	
1	3.20 - 3.24	5.641	6045.066	94.778	1st enantiomer
2	3.20 - 3.24	6.301	333.063	5.222	2nd enantiomer

8e: ee determination of pure major diastereomer

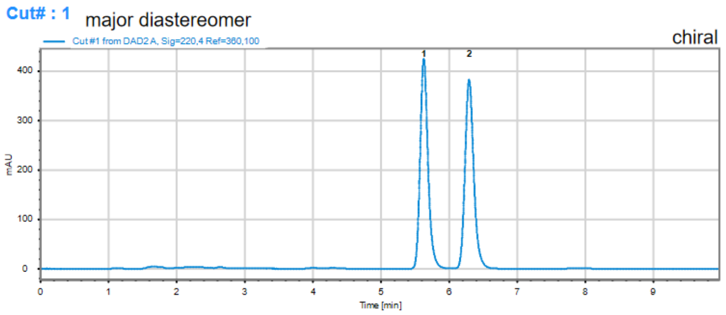

Signal: DAD2 A, Sig=220,4 Ref=360,100

Compound	Cut	Ret.Time	Area	Width	Height	Symmetry	
1	1	5.632	3395.165<br>**3395.165**	0.121	425.007	0.799	1st enantiomer
2	1	6.298	3392.091<br>**3392.091**	0.135	382.410	0.835	2nd enantiomer

rac-**8e**: separation of enantiomers of pure major diastereomer

Methyl (5*R*,6*R*,*E*)-6-hydroxy-5-methyl-6-(naphthalen-2-yl)hex-2-enoate (9). Prepared analogously according to the representative procedure followed by an oxidative work-up. Purified by flash chromatography (SiO_2, hexane/ethyl acetate 25:1 to 5:1) to give the title compound as a colorless oil (78 mg, 95% yield, >20:1 dr, 93% ee).

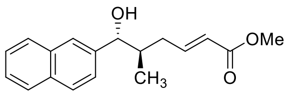

$[\alpha]_D^{20}$ = –28.1 (c = 0.53, $CHCl_3$); ^{1}H NMR (400 MHz, $CDCl_3$) δ 7.85-7.80 (m, 3H), 7.74 (dd, *J* = 1.7, 0.8 Hz, 1H), 7.50-7.47 (m, 2H), 7.45 (dd, *J* = 8.6, 1.7 Hz, 1H), 7.01 (ddd, *J* = 15.6, 8.2, 6.7 Hz, 1H), 5.87 (dt, *J* = 15.6, 1.4 Hz, 1H), 4.59 (d, *J* = 7.2 Hz, 1H), 3.72 (s, 3H), 2.60 (dddd, *J* = 13.9, 6.6, 3.6, 1.8 Hz, 1H), 2.21 (app. dtd, *J* = 13.7, 8.4, 1.2 Hz, 1H), 2.12 (app. dtd, *J* = 8.7, 6.8, 3.6 Hz, 1H), 0.80 (d, *J* = 6.7 Hz, 3H); ^{13}C NMR (101 MHz, $CDCl_3$) δ 167.1, 148.3, 140.6, 133.3, 133.2, 128.4, 128.1, 127.8, 126.4, 126.1, 125.7, 124.5, 122.6, 78.6, 51.5, 39.7, 35.3, 16.2; IR (ATR): ṽ = 3471, 3055, 2955, 2930, 1718, 1653, 1601, 1508, 1435, 1374, 1316, 1269, 1242, 1215, 1167, 1123, 1040, 980, 896, 858, 818, 747, 698; HRMS (ESI$^+$, *m*/*z*) calculated for $[C_{18}H_{20}O_3]^+$ ([M$^+$]) 284.1407, found 284.1405.

The ee was determined by HPLC analysis: Chiralpak 150 mm IB-N-3, 4.6 mm i.D., acetonitrile/water = 60:40, v = 0.5 mL/min, λ = 225 nm, t(minor) = 12.98 min, t(major) = 14.49 min.

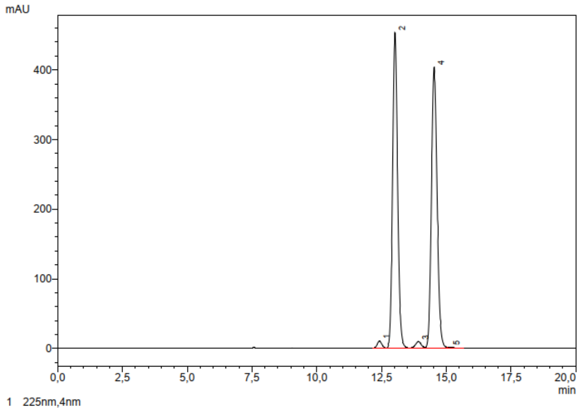

1 225nm,4nm

PDA Ch1 225nm

Peak #	Ret. Time	Area %	Name
1	12,42	1,02	1. Enantiomer Diastereomer 1
2	13,02	48,76	1. Enantiomer Diastereomer 2
3	13,93	1,19	2. Enantiomer Diastereomer 1
4	14,53	48,83	2. Enantiomer Diastereomer 2
5	15,12	0,20	
Total		100,00	

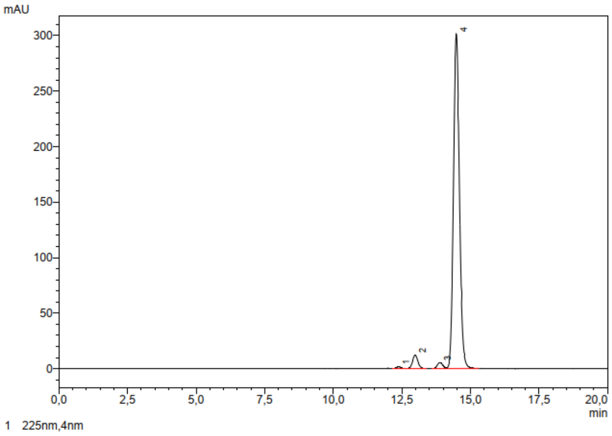

1 225nm,4nm

PDA Ch1 225nm

Peak #	Ret. Time	Area %	Name
1	12,38	0,39	1. Enantiomer Diastereomer 1
2	12,98	3,45	1. Enantiomer Diastereomer 2
3	13,89	1,57	2. Enantiomer Diastereomer 1
4	14,49	94,59	2. Enantiomer Diastereomer 2 93.0 % ee
Total		100,00	

Methyl (5*R*,6*R*,*E*)-6-hydroxy-5-methyl-6-(2-methylphenyl)hex-2-enoate (10). Prepared

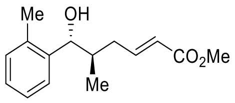

analogously according to the representative procedure followed by an oxidative work-up. Purified by flash chromatography (SiO_2, hexane/ethyl acetate 9:1 to 85:15) to give the title compound as a light yellow oil (39 mg, 54% yield, >20:1 dr, 93% ee).

$[\alpha]_D^{20}$ = 32.6 (c = 0.5, $CHCl_3$); ^{1}H NMR (400 MHz, $CDCl_3$) δ = 7.41 (dd, *J* = 7.5, 1.5 Hz, 1H), 7.22 (td, *J* = 7.5, 2.0 Hz, 1H), 7.17 (td, *J* = 7.5, 1.5 Hz, 1H), 7.13 (dd, *J* = 7.5, 1.5 Hz, 1H), 6.98 (ddd, *J* = 15.5, 8.5, 6.5 Hz, 1H), 5.86 (ddd, *J* = 15.5, 1.5, 1.0 Hz, 1H), 4.70 (d, *J* = 7.5 Hz, 1H), 3.72 (s, 3H), 2.65 (dddd, *J* = 14.0, 6.5, 3.5, 2.0 Hz, 1H), 2.34 (s, 3H), 2.20 (dtd, *J* = 14.0, 9.0, 1.5 Hz, 1H), 2.07-1.96 (m, 1H), 0.80 (d, *J* = 7.0 Hz, 3H); ^{13}C NMR (101 MHz, $CDCl_3$) δ = 167.1, 148.6, 141.7, 135.2, 130.6, 127.5, 126.4, 126.2, 122.4, 74.6, 51.5, 39.2, 35.0, 19.5, 16.3; IR (ATR): ṽ = 3467, 3022, 2954, 1720, 1703, 1436, 1314, 1271, 1216, 1167, 1037, 980, 757; HRMS (EI, *m*/*z*) calculated for $[C_{15}H_{20}O_3]^+$ ($[M^+]$) 248.1409, found 248.1407.

The ee was determined by HPLC analysis: Chiralpak 150 mm IB-N-3, 4.6 mm i.D., *n*-heptane/isopropanol = 95:5, v = 1.0 mL/min, λ = 220 nm, t(minor) = 8.18 min, t(major) = 9.51 min.

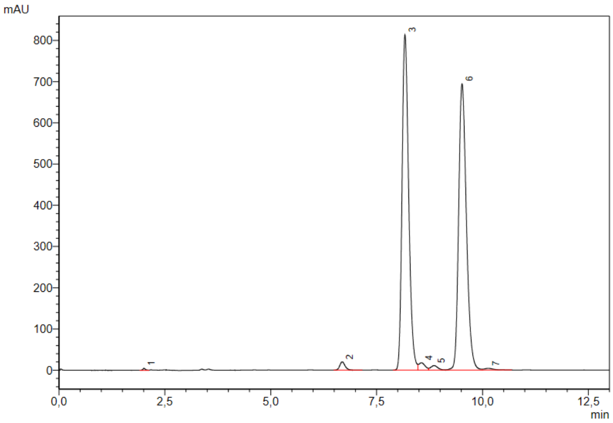

1 220nm,4nm

PDA Ch1 220nm

Peak #	Ret. Time	Area %	Name
1	2,00	0,12	
2	6,69	0,95	
3	8,17	48,41	1. Enantiomer
4	8,56	1,02	
5	8,86	0,70	
6	9,52	48,45	2. Enantiomer
7	10,14	0,35	
Total		100,00	

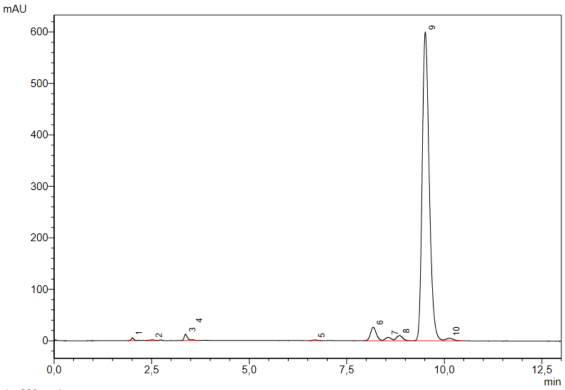

1 220nm,4nm

PDA Ch1 220nm

Peak #	Ret. Time	Area %	Name
1	2,00	0,31	
2	2,51	0,22	
3	3,37	0,87	
4	3,54	0,14	
5	6,68	0,16	
6	8,18	3,33	1. Enantiomer
7	8,56	0,88	
8	8,85	1,41	
9	9,51	91,82	2. Enantiomer 93.0 % ee
10	10,13	0,86	
Total		100,00	

Methyl (5*R*,6*R*,*E*)-6-hydroxy-5-methyl-6-(3,4,5-trimethoxyphenyl)hex-2-enoate (11).

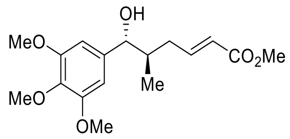

Prepared analogously according to the representative procedure followed by an oxidative work-up. Purified by flash chromatography (SiO_2, toluene/*tert*-butyl methyl ether, 4:1 to 3:1) to give the title compound as a yellow oil (84 mg, 89% yield, >20:1 dr, 90% ee).

$[\alpha]_D^{20}$ = 2.6 (c = 0.5, $CHCl_3$); ^{1}H NMR (400 MHz, $CDCl_3$) δ = 6.99 (ddd, *J* = 15.5, 8.5, 6.5 Hz, 1H), 6.53 (s, 2H), 5.87 (dt, *J* = 15.5, 1.5 Hz, 1H), 4.33 (d, *J* = 7.5 Hz, 1H), 3.85 (s, 6H), 3.83 (s, 3H), 3.72 (s, 3H), 2.56 (dddd, *J* = 14.0, 6.5, 4.0, 1.5 Hz, 1H), 2.19 (app. dtd, *J* = 14.0, 8.5, 1.5 Hz, 1H), 2.04-1.92 (m, 1H), 1.85 (br. s, 1H, -OH), 0.78 (s, 3H); ^{13}C NMR (101 MHz, $CDCl_3$) δ = 167.1, 153.3, 148.2, 139.1, 137.5, 122.6, 103.5, 78.7, 61.0, 56.3, 51.6, 39.8, 35.3, 16.3; IR (ATR): ṽ = 3495, 2935, 2839, 1719, 1652, 1591, 1457, 14020, 1323, 1231, 1122, 1004, 839, 701; HRMS (ESI, *m*/*z*) calculated for $[C_{17}H_{24}O_6+Na]^+$ ($[M+Na]^+$) 347.1465, found 347.1465.

The ee was determined by 2D HPLC analysis:

Step 1: Purification: 50 mm Zorbax Eclipse Plus C18, 4.6 mm i.D., acetonitrile/water gradient 20% to 60% over 5 minutes, v = 1.0 mL/min, λ = 220 nm, t(major) = 3.93 min, 308 K.

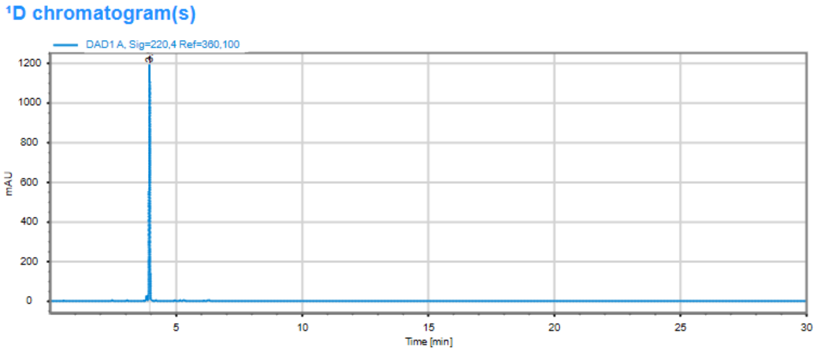

Sampling table (1D)

Cut group	Cut #	1D Cut start [min]	1D Ret. time [min]	1D Duration [min]	Trigger	2D Run start [min]
	1	3.93	3.961	0.04	Peak	3.98

11: separation of impurities on achiral column

Step 2: Chiral resolution of the major diastereomer: Daicel 150 mm Chiralpak IB-N-3, 4.6 mm i.D., methanol/water = 75:25, v = 1.0 mL/min, λ = 220 nm, t(minor) = 3.99 min, t(major) = 4.48 min.

Cut# : 1

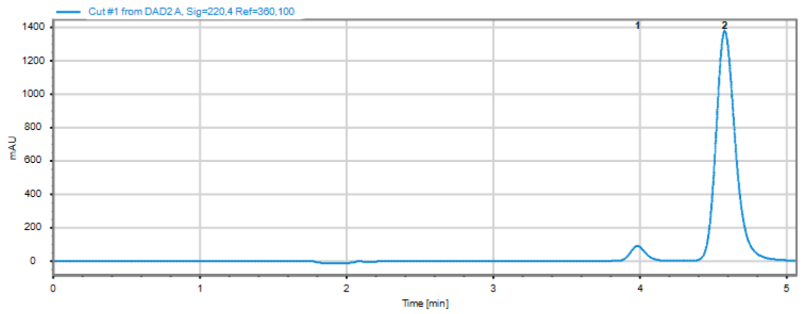

Signal: DAD2 A, Sig=220,4 Ref=360,100

Compound	Cut	Ret.Time	Area	Width	Height	Symmetry
1	1	3.985	649.282<br>**649.282**	0.113	89.013	0.824
2	1	4.579	12586.084<br>**12586.084**	0.141	1375.462	0.760

Component table

Signal: DAD2 A, Sig=220,4 Ref=360,100

Component	¹D Sampling range [min]	Ret.Time ²D [min]	Area	Area%	
1	3.93 - 3.97	3.985	649.282	4.906	1. enantiomer
2	3.93 - 3.97	4.579	12586.084	95.094	2. enantiomer

ee = 90.2%

11: ee determination of pure major diastereomer

Cut# : 1

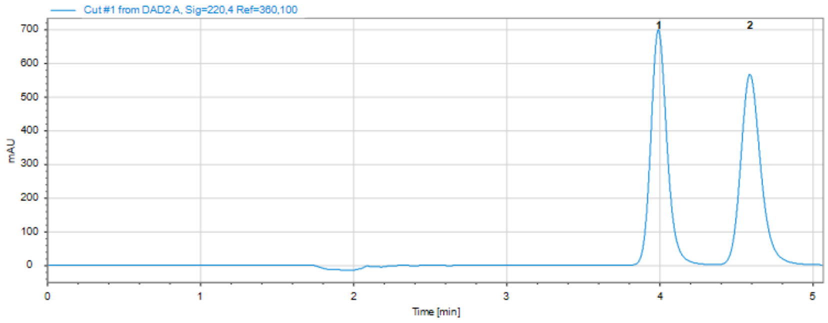

Signal: DAD2 A, Sig=220,4 Ref=360,100

Compound	Cut	Ret.Time	Area	Width	Height	Symmetry
1	1	3.992	5172.499<br>**5172.499**	0.113	698.618	0.811
2	1	4.590	5176.303<br>**5176.303**	0.139	566.361	0.793

rac-**11**: separation of enantiomers of pure major diastereomer

Methyl (5*R*,6*R*,*E*)-6-(furan-2-yl)-6-hydroxy-5-methylhex-2-enoate (12). Prepared analogously according to the representative procedure followed by an oxidative work-up. Purified by flash chromatography (SiO_2, toluene/*tert*-butyl methyl ether, 98:2 to 95:5 to 9:1) to give the title compound as a light yellow oil (42 mg, 65% yield, >20:1 dr, 96% ee).

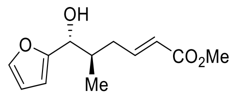

$[\alpha]_D^{20}$ = 38.4 (c = 1.0, $CHCl_3$); ^{1}H NMR (400 MHz, $CDCl_3$) δ = 7.37 (dd, *J* = 2.0, 1.0 Hz, 1H), 6.98 (ddd, *J* = 15.5, 8.0, 7.0 Hz, 1H), 6.33 (dd, *J* = 3.0, 2.0 Hz, 1H), 6.24 (app. dt, *J* = 3.0, 1.0 Hz, 1H), 5.86 (dt, *J* = 15.5, 1.5 Hz, 1H), 4.46 (d, *J* = 7.0 Hz, 1H), 3.72 (s, 3H), 2.58-2.49 (m, 1H), 2.21-2.11 (m, 2H), 1.96 (br. s, 1H, –OH), 0.84 (d, *J* = 6.5 Hz, 3H); ^{13}C NMR (101 MHz, $CDCl_3$) δ = 167.1, 155.5, 147.9, 142.1, 122.7, 110.3, 107.1, 71.9, 51.6, 37.9, 35.3, 15.8; IR (ATR): ṽ = 3435, 2955, 2932, 1719, 1654, 1436, 1272, 1169, 1108, 981, 735; HRMS (EI, *m*/*z*) calculated for $[C_{12}H_{16}O_4]^+$ ($[M^+]$) 224.1043, found 224.1043.

The ee was determined by 2D HPLC analysis:

Step 1: Purification: 50 mm Eclipse Plus C18, 4.6 mm i.D., methanol/water 50:50, v = 1.0 mL/min, λ = 220 nm, t(major) = 3.28 min, 308 K.

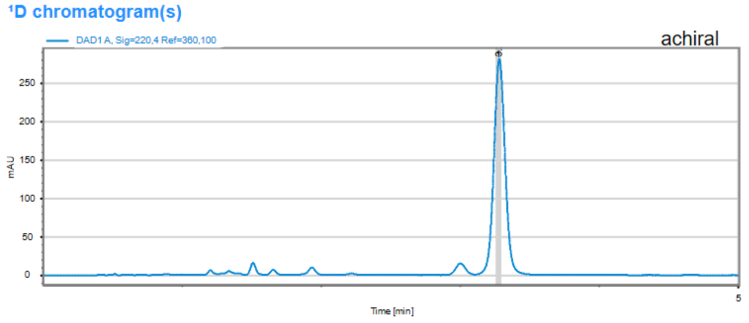

Sampling table (¹D)

Cut group	Cut #	¹D Cut start [min]	¹D Ret. time [min]	¹D Duration [min]	Trigger	²D Run start [min]	
	1	3.25	3.283	0.04	Peak	3.30	major diastereomer

12: separation of impurities on achiral column

Step 2: Chiral resolution of the major diastereomer: Chiralcel 150 mm IG-3, 4.6 mm i.D., acetonitrile/water = 80:20, v = 1.0 mL/min, λ = 220 nm, t(major) = 4.08 min, t(minor) = 6.97 min.

Cut# : 1

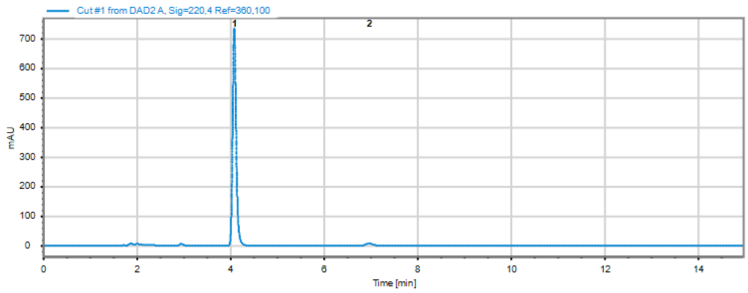

Signal: DAD2 A, Sig=220,4 Ref=360,100

Compound	Cut	Ret.Time	Area	Width	Height	Symmetry	
1	1	4.083	3871.155<br>**3871.155**	0.081	734.094	0.810	1st enantiomer
2	1	6.968	84.748<br>**84.748**	0.142	7.963	0.892	2nd enantiomer

Component table

chiral

Signal: DAD2 A, Sig=220,4 Ref=360,100

Component	¹D Sampling range [min]	Ret.Time ²D [min]	Area	Area%	
1	3.25 - 3.29	4.083	3871.155	97.858	1st enantiomer
2	3.25 - 3.29	6.968	84.748	2.142	2nd enantiomer

= 95.7 % ee

12: ee determination of pure major diastereomer

Cut# : 1

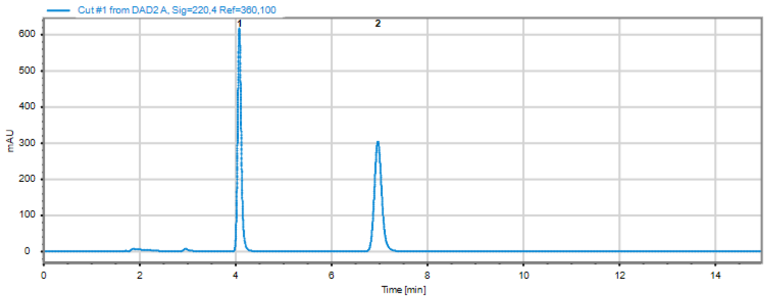

Signal: DAD2 A, Sig=220,4 Ref=360,100

Compound	Cut	Ret.Time	Area	Width	Height	Symmetry	
1	1	4.083	3244.120<br>**3244.120**	0.081	615.922	0.809	1st enantiomer
2	1	6.971	3245.106<br>**3245.106**	0.165	303.816	0.861	2nd enantiomer

rac-**12**: separation of enantiomers of pure major diastereomer

Methyl (5*R*,6*R*,*E*)-6-(3-chlorophenyl)-6-hydroxy-5-methylhex-2-enoate (13). Prepared analogously according to the representative procedure followed by an oxidative work-up. Purified by flash chromatography (SiO_2, hexane/ethyl acetate 9:1 to 87:13) to give the title compound as a light yellow oil (61 mg, 78% yield, >20:1 dr, 93% ee).

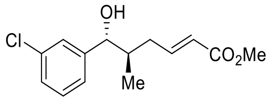

$[\alpha]_D^{20}$ = 6.6 (c = 0.5, $CHCl_3$); ^{1}H NMR (400 MHz, $CDCl_3$) δ = 7.35-7.33 (m, 1H), 7.32-7.26 (m, 2H), 7.23-7.18 (m, 1H), 6.98 (ddd, *J* = 15.5, 8.5, 6.5 Hz, 1H), 5.87 (dd, *J* = 15.5, 1.5 Hz, 1H), 4.44 (d, *J* = 7.0 Hz, 1H), 3.74 (s, 3H), 2.53 (dddd, *J* = 14.0, 6.5, 4.0, 1.5 Hz, 1H), 2.17 (app. dtd, *J* = 14.0, 8.5, 1.5 Hz, 1H), 2.06-1.94 (m, 2H incl. -OH), 0.81 (d, *J* = 7.0 Hz, 3H); ^{13}C NMR (101 MHz, $CDCl_3$) δ = 167.1, 148.0, 145.4, 134.5, 129.8, 128.0, 126.9, 124.9, 122.7, 77.8, 51.6, 39.8, 35.0, 16.1; IR (ATR): ṽ = 3439, 2954, 1701, 1652, 1435, 1272, 1168, 1034, 980, 784, 694; HRMS (ESI, *m*/*z*) calculated for $[C_{14}H_{17}ClO_3+Na]^+$ ($[M+Na]^+$) 291.0758, found 291.0758.

The ee as determined by HPLC analysis: Chiralpak 150 mm IC-3, 4.6 mm i.D., *n*-heptane/isopropanol = 95:5, v = 1.0 mL/min, λ = 230 nm, t(major) = 9.86 min, t(minor) = 12.72 min.

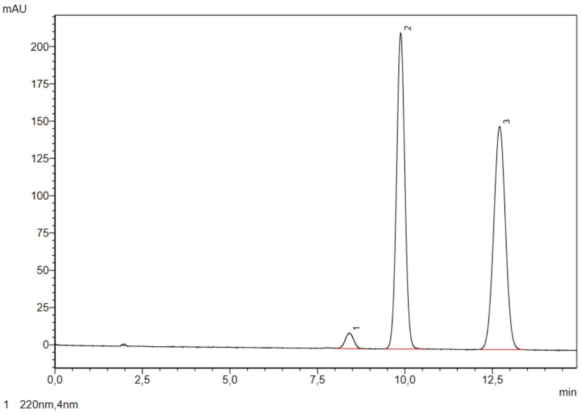

PDA Ch1 220nm

Peak #	Ret. Time	Area %	Name
1	8,41	2,45	
2	9,88	48,80	1. Enantiomer
3	12,70	48,75	2. Enantiomer
Total		100,00	

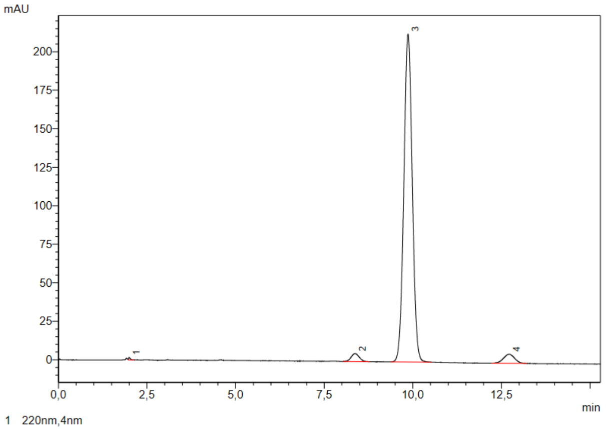

PDA Ch1 220nm

Peak #	Ret. Time	Area %	Name
1	1,99	0,15	
2	8,37	2,26	
3	9,86	94,10	1. Enantiomer 92.8 % ee
4	12,72	3,49	2. Enantiomer
Total		100,00	

Methyl (5*R*,6*R*,*E*)-6-(3,5-dibromophenyl)-6-hydroxy-5-methylhex-2-enoate (14). Prepared

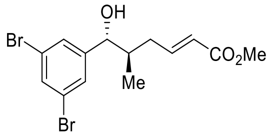

analogously according to the representative procedure followed by an oxidative work-up. Purified by flash chromatography (SiO_2, hexane/ethyl acetate 9:1 to 85:15) to give the title compound as a light yellow oil (101 mg, 89% yield, >20:1 dr, 89% ee).

When prepared on larger scale, as described above: 346 mg, 88% yield, >20:1 dr, 88% ee

$[\alpha]_D^{20}$ = 1.3 (c = 0.5, $CHCl_3$); ^{1}H NMR (400 MHz, $CDCl_3$) δ = 7.57 (t, *J* = 2.0 Hz, 1H), 7.41-7.39 (m, 2H), 6.92 (ddd, *J* = 15.5, 8.5, 6.5 Hz, 1H), 5.85 (dt, *J* = 15.5, 1.5 Hz, 1H), 4.39 (d, *J* = 7.0 Hz, 1H), 3.72 (s, 3H), 2.46 (dddd, *J* = 14.0, 6.4, 4.0, 1.5 Hz, 1H), 2.15 (dtd, 14.0, 8.5, 1.5 Hz, 1H), 2.06 (br. s, 1H, O-H), 1.95 (dtd, *J* = 8.5, 7.0, 4. Hz, 1H), 0.81 (d, *J* = 7.0 Hz, 3H); ^{13}C NMR (101 MHz, $CDCl_3$) δ = 167.0, 147.6, 147.3, 133.4, 128.6, 123.1, 122.9, 77.2, 51.6, 39.8, 34.8, 16.2; IR (ATR): ṽ = 3444, 2952, 1700, 1652, 1556, 1424, 1273, 1169, 1036, 979, 885, 739; HRMS (ESI, *m*/*z*) calculated for $[C_{14}H_{16}Br_2O_3+Na]^+$ ($[M+Na]^+$) 412.9355, found 412.9359.

The ee was determined by HPLC analysis: Chiralpak 150 mm IC-3, 4.6 mm i.D., *n*-heptane/isopropanol = 95:5, v = 1.0 mL/min, λ = 220 nm, t(major) = 7.03 min, t(minor) = 10.00 min.

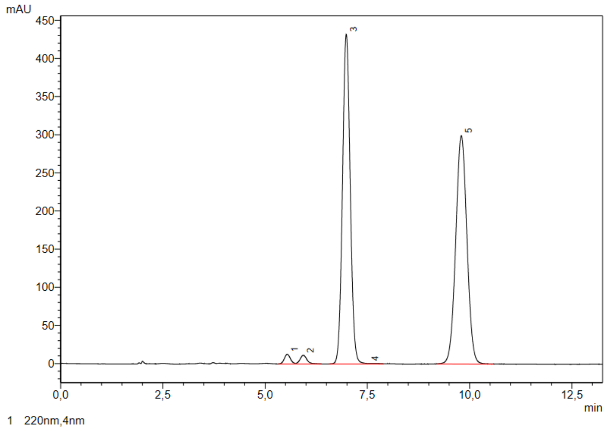

PDA Ch1 220nm

Peak #	Ret. Time	Area %	Name
1	5,54	1,11	
2	5,93	1,12	
3	6,98	48,71	1. Enantiomer
4	7,51	0,10	
5	9,80	48,95	2. Enantiomer
Total		100,00	

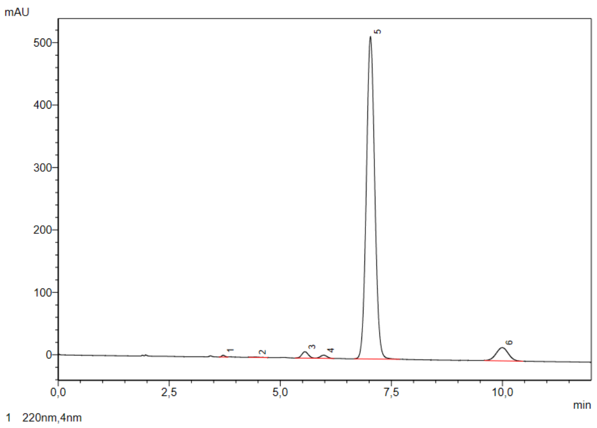

PDA Ch1 220nm

Peak #	Ret. Time	Area %	Name
1	3,72	0,20	
2	4,44	0,11	
3	5,55	1,41	
4	5,97	0,68	
5	7,03	92,04	1. Enantiomer 88.6 % ee
6	10,00	5,56	2. Enantiomer
Total		100,00	

Methyl (5*R*,6*R*,*E*)-6-hydroxy-5-methyl-8-phenyl)oct-2-enoate (15a). Prepared analogously according to the representative procedure followed by an oxidative work-up. Purified by flash chromatography (SiO_2, hexane/ethyl acetate 9:1 to 85:15) to give the title compound as a light yellow oil (62 mg, 81% yield, >20:1 dr, 96% ee).

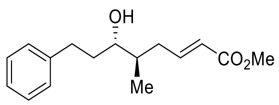

$[\alpha]_D^{20}$ = –46.8 (c = 0.5, $CHCl_3$); ^1H NMR (400 MHz, $CDCl_3$) δ = 7.32-7.26 (m, 2H), 7.22-7.17 (m, 3H), 6.95 (ddd, *J* = 15.5, 8.0, 7.0 Hz, 1H), 5.83 (dt, *J* = 15.5, 1.5 Hz, 1H), 3.73 (s, 3H), 3.47 (ddd, *J* = 9.0, 6.0, 3.0 Hz, 1H), 2.85 (ddd, *J* = 13.5, 10.0, 5.5 Hz, 1H), 2.65 (ddd, *J* = 13.5, 9.5, 6.5 Hz, 1H), 2.43 (dddd, *J* = 14.5, 6.5, 4.5, 1.5 Hz, 1H), 2.12-2.03 (m, 1H), 1.82 (dddd, *J* = 14.0, 10.0, 7.0, 3.0 Hz, 1H), 1.77-1.67 (m, 2H), 0.91 (d, *J* = 7.0 Hz, 3H); ^{13}C NMR (101 MHz, $CDCl_3$) δ = 167.1, 148.4, 142.1, 128.6, 128.5, 126.1, 122.4, 74.9, 51.6, 38.7, 35.8, 35.2, 32.4, 15.8; IR (ATR): ṽ = 3474, 3026, 2950, 1721, 1705, 1655, 1436, 1314, 1271, 1172, 1039, 981, 700; LRMS *m*/*z* (ESI$^+$) [M+Na]$^+$ 285; HRMS (ESI$^+$, *m*/*z*) calculated for $[C_{16}H_{22}O_3+Na]^+$ ($[M+Na]^+$) 285.1461, found 285.1462.

The ee was determined by HPLC analysis: Chiralpak 150 mm IC-3, 4.6 mm i.D., *n*-heptane/isopropanol = 95:5, v = 1.0 mL/min, λ = 220 nm, t(major) = 13.48 min, t(minor) = 19.62 min.

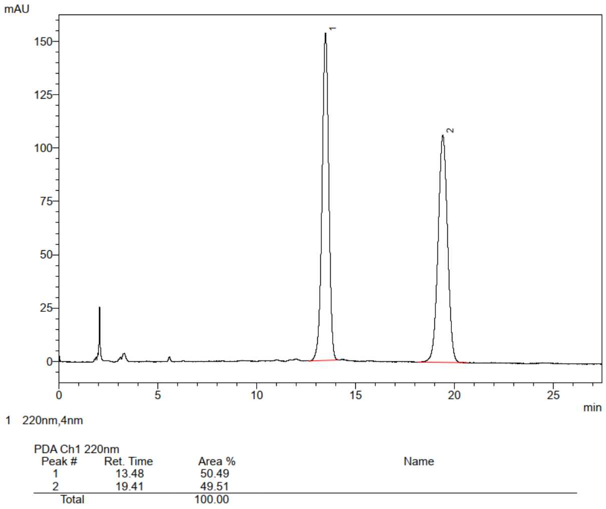

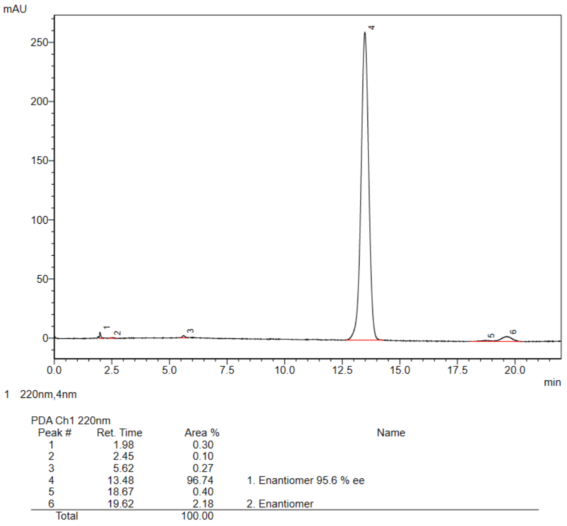

Methyl (5*R*,6*S*,*E*)-5-ethyl-6-hydroxy-8-phenyloct-2-enoate (15b). Prepared analogously

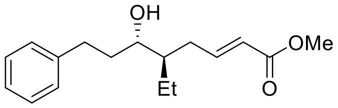

according to the representative procedure followed by an oxidative work-up. Purified by flash chromatography (SiO_2, hexane/ethyl acetate 20:1 to 4:1) to give the title compound as a colorless oil (48 mg, 60% yield, >20:1 dr, 96% ee).

$[\alpha]_D^{20}$ = –62.1 (c = 0.5, $CHCl_3$); ^1H NMR (400 MHz, $CDCl_3$) δ 7.23-7.17 (m, 2H), 7.15-7.06 (m, 3H), 6.89 (ddd, *J* = 15.3, 7.8, 7.2 Hz, 1H), 5.74 (dt, *J* = 15.6, 1.5 Hz, 1H), 3.64 (s, 3H), 3.56 (dt, *J* = 8.5, 4.4 Hz, 1H), 2.75 (ddd, *J* = 13.7, 8.8, 6.5 Hz, 1H), 2.57 (ddd, *J* = 13.7, 8.9, 7.1 Hz, 1H), 2.26 (dddd, *J* = 14.1, 7.0, 5.1, 1.6 Hz, 1H), 2.16 (app. dtd, *J* = 14.8, 7.5, 1.5 Hz, 1H), 1.74-1.65 (m, 2H), 1.48-1.32 (m, 3H), 1.30-1.19 (m, 1H), 0.81 (t, *J* = 7.3 Hz, 3H); ^{13}C NMR (101 MHz, $CDCl_3$) δ 167.1, 149.0, 142.0, 128.6, 128.5, 126.1, 122.1, 72.5, 51.6, 45.1, 36.3, 32.6, 32.0, 22.9, 11.6; IR (ATR): ṽ = 3471, 3026, 2953, 2932, 2875, 1722, 1706, 1654, 1496, 1454, 1436, 1324, 1273, 1214, 1170, 1124, 1041, 986, 926, 749, 700; HRMS (ESI^+, *m*/*z*) calculated for $[C_{17}H_{24}O_3]^+$ ($[M^+]$) 276.1720, found 276.1721.

The ee was determined by HPLC analysis: Chiralpak 150 mm IB-N-3, 4.6 mm i.D., *n*-heptane/*i*-propanol = 90:10, v = 1.0 mL/min, λ = 210 nm, t(major) = 4.91 min, t(minor) = 7.07 min.

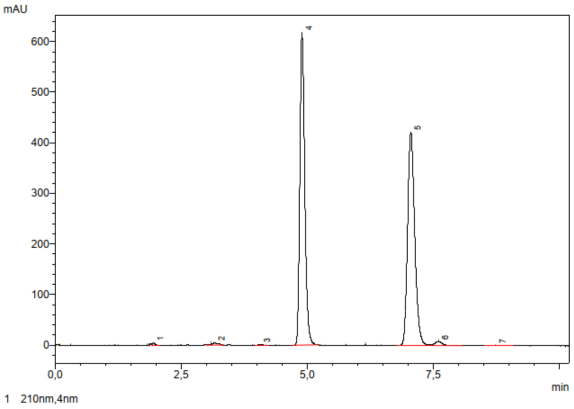

PDA Ch1 210nm

Peak #	Ret. Time	Area %	Name
1	1,95	0,19	
2	3,16	0,50	
3	4,06	0,14	
4	4,90	49,20	1. Enantiomer
5	7,05	48,78	2. Enantiomer
6	7,60	1,03	
7	8,74	0,15	
Total		100,00	

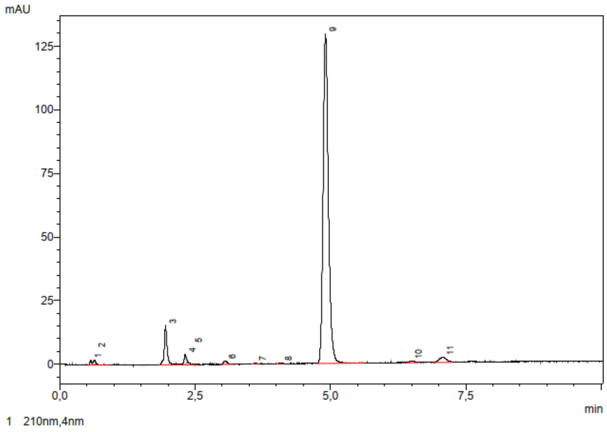

PDA Ch1 210nm

Peak #	Ret. Time	Area %	Name
1	0,57	0,44	
2	0,64	0,72	
3	1,95	5,87	
4	2,32	1,59	
5	2,43	0,18	
6	3,05	0,71	
7	3,61	0,16	
8	4,09	0,14	
9	4,91	87,63	1. Enantiomer 95.5 % ee
10	6,49	0,55	
11	7,07	2,00	2. Enantiomer
Total		100,00	

Methyl (5*S*,6*S*,*E*)-5-(((*tert*-butyldimethylsilyl)oxy)methyl)-6-hydroxy-8-phenyloct-2-enoate (15c). Prepared analogously according to the representative procedure followed by an oxidative work-up. Purified by flash chromatography (SiO_2, hexane/ethyl acetate 50:1 to 10:1) to give the title compound as a colorless oil (80 mg, 70% yield, >20:1 dr, 95% ee).

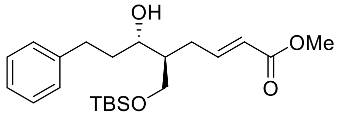

$[\alpha]_D^{20}$ = –2.0 (c = 0.50, $CHCl_3$); ^{1}H NMR (400 MHz, $CDCl_3$) δ 7.31-7.27 (m, 2H), 7.23-7.18 (m, 3H), 6.92 (ddd, *J* = 15.3, 8.3, 6.7 Hz, 1H), 5.84 (dt, *J* = 15.5, 1.5 Hz, 1H), 3.92-3.86 (m, 1H), 3.73 (d, *J* = 4.1 Hz, 2H), 3.73 (s, 3H), 2.87 (ddd, *J* = 13.6, 10.0, 5.3 Hz, 1H), 2.64 (ddd, *J* = 13.8, 9.7, 6.7 Hz, 1H), 2.42-2.26 (m, 2H), 1.87 (app. dtd, *J* = 13.5, 9.5, 5.3 Hz, 1H), 1.75-1.65 (m, 2H), 1.60 (br s, 1H), 0.90 (s, 9H), 0.07 (s, 3H), 0.06 (s, 3H); ^{13}C NMR (101 MHz, $CDCl_3$) δ 166.9, 148.2, 142.3, 128.6, 128.6, 126.0, 122.6, 74.1, 65.5, 51.6, 43.9, 36.2, 32.6, 28.0, 25.9, 18.2, -5.5; IR (ATR): ṽ = 3507, 2951, 2929, 2857, 1723, 1656, 1436, 1318, 1254, 1210, 1163, 1093, 1043, 1006, 980, 938, 833, 775, 748, 699; HRMS (ESI$^+$, *m*/*z*) calculated for $[C_{22}H_{36}O_4Si+Na]^+$ ($[M+Na]^+$) 415.2275, found 415.2274.

The ee was determined by HPLC analysis: Chiralpak 150 mm IC-3, 4.6 mm i.D., *n*-heptane/*i*-propanol = 95:5, v = 1.0 mL/min, λ = 220 nm, t(major) = 6.30 min, t(minor) = 8.86 min.

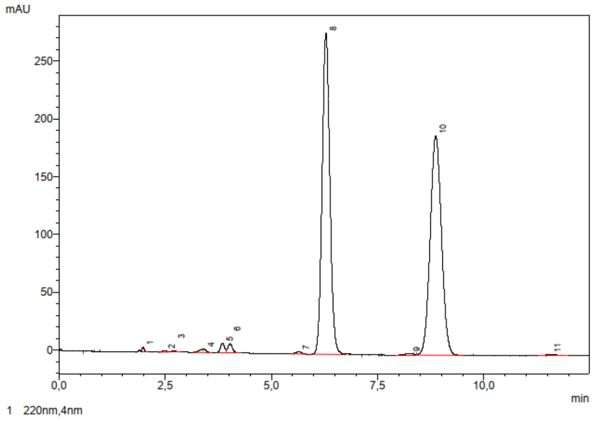

PDA Ch1 220nm

Peak #	Ret. Time	Area %	Name
1	1,97	0,23	
2	2,48	0,16	
3	2,71	0,14	
4	3,41	0,46	
5	3,85	0,77	
6	4,03	0,80	
7	5,65	0,27	
8	6,28	48,44	1. Enantiomer
9	8,25	0,30	
10	8,87	48,17	2. Enantiomer
11	11,56	0,27	
Total		100,00	

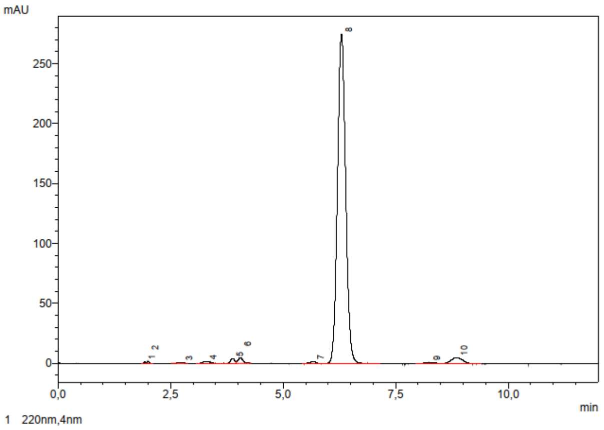

PDA Ch1 220nm

Peak #	Ret. Time	Area %	Name
1	1,92	0,12	
2	1,99	0,17	
3	2,75	0,14	
4	3,29	0,57	
5	3,88	0,73	
6	4,05	1,00	
7	5,67	0,51	
8	6,30	93,59	1. Enantiomer 94.6 % ee
9	8,25	0,58	
10	8,86	2,59	2. Enantiomer
Total		100,00	

Methyl (5*S*,6*S*,*E*)-6-hydroxy-5,8-diphenyloct-2-enoate (15d). Prepared analogously

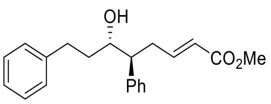

according to the representative procedure followed by an oxidative work-up. A ~1:1 mixture of regioisomers was obtained, which were separated by flash chromatography (SiO_2, toluene/methyl *tert*-butyl ether 98:2 to 95:5) to give the title compound as a colourless oil (43 mg, 46% yield, >20:1 dr, 76% ee). No attempt was made to optimise the enantioselectivity by e.g. lowering the temperature.

$[\alpha]_D^{20}$ = 13.5 (c = 0.51, $CHCl_3$); ^1H (400 MHz, $CDCl_3$) δ = 7.32-7.21 (m, 5H), 7.19-7.14 (m, 1H), 7.13-7.08 (m, 4H), 6.79 (dt, *J* = 15.5, 7.0 Hz, 1H), 5.73 (dt, *J* = 15.5, 1.5 Hz, 1H), 3.75 (ddd, *J* = 9.5, 7.0, 3.0 Hz, 1H), 3.65 (s, 3H), 2.88-2.75 (m, 3H), 2.64-2.54 (m, 2H), 1.72 (dddd, *J* = 14.0, 10.0, 7.0, 3.0 Hz, 1H), 1.64-1.54 (m, 2H); ^{13}C (101 MHz, $CDCl_3$) δ = 166.9, 147.8, 141.8, 141.0, 128.8, 128.5(4), 128.5(0), 128.4, 127.1, 126.0, 122.3, 74.8, 51.8, 51.5, 36.5, 34.1, 32.2; IR (ATR): ṽ = 3457, 3026, 2949, 2922, 2856, 1720, 1655, 1435, 1273, 1208, 1154, 1030, 750, 698; LRMS *m/z* (ESI^+) $[M+Na]^+$ 285; HRMS (ESI^+, *m/z*) calculated for $[C_{21}H_{24}O_3Na]^+$ 347.1618, found 347.1619.

The ee was determined by HPLC analysis: Chiralpak 150 mm IB-N3, 4.6 mm i.D., acetonitrile/water = 60:40, v = 1.0 mL/min, λ = 220 nm, t(major) = 6.16 min, t(minor) = 7.39 min.

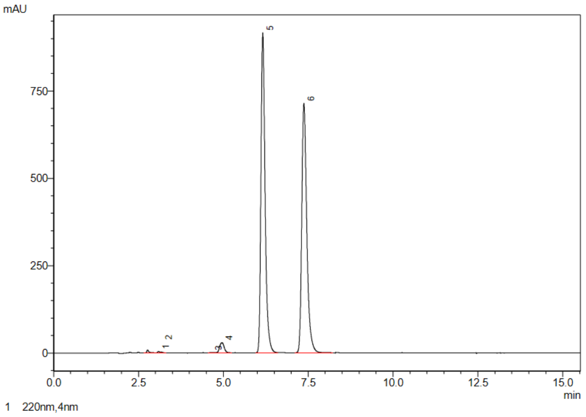

1 220nm,4nm

PDA Ch1 220nm

Peak #	Ret. Time	Area %	Name	Separation Factor	Resolution(DAB)
1	3.10	0.36		--	--
2	3.18	0.06		1.07	--
3	4.65	0.08		2.06	--
4	4.96	1.76		1.11	--
5	6.16	50.28	1. Enantiomer	1.38	5.19
6	7.37	47.46	2. Enantiomer	1.28	5.13
Total		100.00			

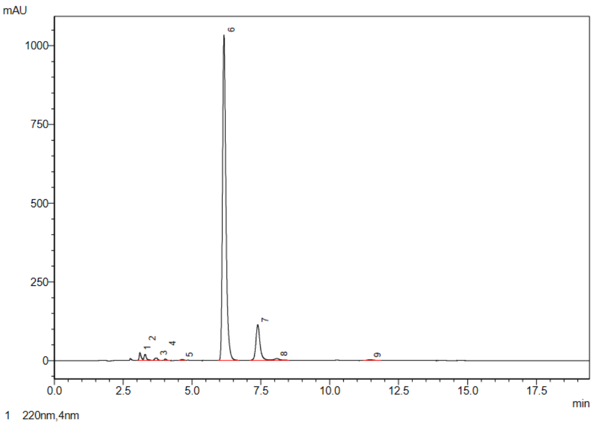

1 220nm,4nm

PDA Ch1 220nm

Peak #	Ret. Time	Area %	Name
1	3.11	1.17	
2	3.30	1.18	
3	3.71	0.61	
4	4.03	0.24	
5	4.65	0.20	
6	6.16	84.12	1. Enantiomer
7	7.39	11.29	2. Enantiomer
8	8.08	0.86	
9	11.47	0.35	
Total		100.00	

ee = 76.3 %

A second fraction contained the regioisomeric product **methyl (*R*,*E*)-2-((*S*)-1-hydroxy-3-phenylpropyl)-5-phenylpent-4-enoate (15d')**, which was separated by

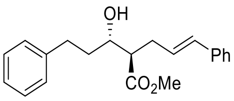

flash chromatography (SiO_2, toluene/methyl *tert*-butyl ether 98:2 to 95:5

and then re-purified (SiO_2, hexane/ethyl acetate 4:1); colourless oil (38 mg, 40% yield, >20:1 dr, 8% ee).

^1H (400 MHz, $CDCl_3$) δ = 7.33-7.27 (m, 6H), 7.24-7.18 (m, 4H), 6.45-6.39 (m, 1H), 6.19-6.11 (m, 1H), 3.91 (dt, *J* = 8.5, 4.0 Hz, 1H), 3.69 (s, 3H), 2.90 (ddd, *J* = 14.5, 9.5, 5.5 Hz, 1H), 2.73-2.51 (m, 4H), 1.91-1.73 (m, 2H); ^{13}C (101 MHz, $CDCl_3$) δ = 175.2, 141.8, 137.4, 132.2, 128.6(3), 128.6(1), 128.6(0), 127.4, 127.2, 126.3, 126.1, 71.2, 51.9, 51.3, 36.1, 32.3, 30.9; IR (ATR): ṽ = 3332, 3027, 2943, 1730, 1434, 1228, 1147, 1026, 965, 739, 692, 664; HRMS (ESI^+, *m*/*z*) calculated for $[C_{21}H_{24}O_3Na]^+$ 347.1618, found 347.1619.

The ee was determined by HPLC analysis: Chiralpak 150 mm IB-N3, 4.6 mm i.D., acetonitrile/water = 60:40, v = 1.0 mL/min, λ = 220 nm, t(major) = 8.07 min, t(minor) = 11.36 min.

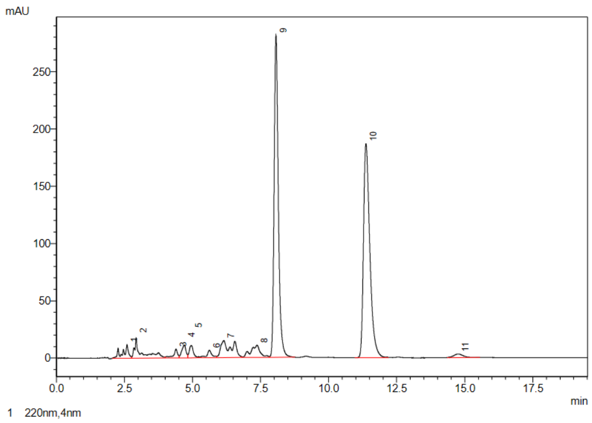

PDA Ch1 220nm

Peak #	Ret. Time	Area %	Name	Separation Factor	Resolution(DAB)
1	2.60	2.14		--	--
2	2.93	4.00		1.16	2.54
3	4.39	1.05		1.60	9.52
4	4.70	1.58		1.08	1.25
5	4.96	1.48		1.06	0.93
6	5.62	1.16		1.15	2.62
7	6.15	5.49		1.10	1.66
8	7.37	3.42		1.22	2.64
9	8.07	39.23	1. Enantiomer	1.10	1.76
10	11.37	39.47	2. Enantiomer	1.44	9.73
11	14.75	0.98		1.31	6.53
Total		100.00			

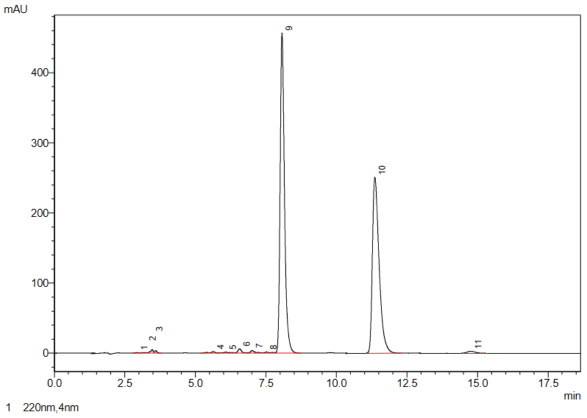

PDA Ch1 220nm

Peak #	Ret. Time	Area %	Name
1	2.93	0.06	
2	3.23	0.11	
3	3.47	0.56	
4	5.63	0.26	
5	6.07	0.22	
6	6.57	0.58	
7	7.01	0.43	
8	7.52	0.21	
9	8.07	52.44	1. Enantiomer
10	11.36	44.51	2. Enantiomer
11	14.77	0.62	
Total		100.00	

ee = 8.2%

Methyl (5*R*,6*R*,*E*)-6-cyclopropyl-6-hydroxy-5-methylhex-2-enoate (16). Prepared

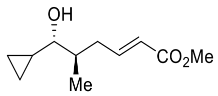

analogously according to the representative procedure followed by an oxidative work-up. Purified by flash chromatography (SiO_2, toluene/*tert*-butyl methyl ether, 95:5 to 88:12) to give the title compound as a colourless oil (36 mg, 63% yield, >20:1 dr, 94% ee).

$[\alpha]_D^{20}$ = 6.9 (c = 0.5, $CHCl_3$); ^{1}H NMR (400 MHz, $CDCl_3$) δ = 7.02-6.94 (m, 1H), 5.85 (dt, *J* = 15.5, 1.5 Hz, 1H), 3.72 (s, 3H), 2.65 (dd, *J* = 9.0, 6.5 Hz, 1H), 2.52 (dddd, *J* = 14.5, 6.5, 4.5, 1.5 Hz, 1H), 2.15 (dtd, *J* = 14.5, 8.5, 1.5 Hz, 1H), 1.93-1.82 (m, 1H), 0.99 (d, *J* = 7.0 Hz, 3H), 0.91 (dtt, *J* = 9.0, 8.0, 5.0 Hz, 1H), 0.64-0.56 (m, 1H), 0.53-0.46 (m, 1H), 0.31-0.20 (m, 2H); ^{13}C NMR (101 MHz, $CDCl_3$) δ = 167.2, 148.7, 122.3, 80.3, 51.5, 39.3, 35.7, 16.0, 15.9, 4.1, 2.0; IR (ATR): ṽ = 3466, 3003, 2955, 2880, 1720, 1706, 1654, 1436, 1270, 1174, 1025, 980; HRMS (ESI, *m*/*z*) calculated for $[C_{11}H_{18}O_3+Na]^+$ ($[M+Na]^+$) 221.1151, found 221.1148.

The ee was determined by 2D HPLC analysis

Step 1: Purification: 250 mm YMC PVA-SIL, 4.6 mm i.D., *n*-heptane/isopropanol 98:2, v = 1.0 mL/min, λ = 210 nm, t(major) = 13.23 min, 308 K.

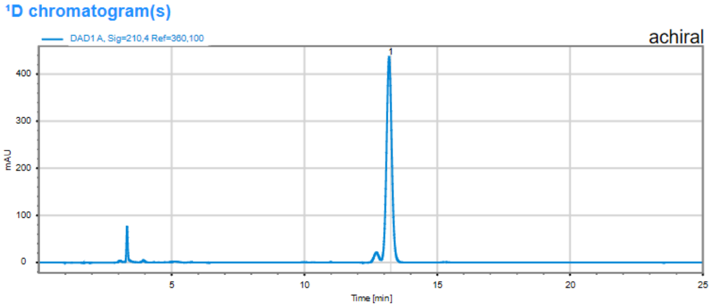

Sampling table (¹D)

Cut group	Cut #	¹D Cut start [min]	¹D Ret. time [min]	¹D Duration [min]	Trigger	²D Run start [min]	
	1	13.23	***	0.04	Time	13.28	major diastereomer

16: separation of impurities on achiral column

Step 2: Chiral resolution of the major diastereomer: Chiralcel 150 mm OD-3, 4.6 mm i.D., *n*-heptane/isopropanol = 95:5, v = 1.0 mL/min, λ = 210 nm, t(major) = 5.80 min, t(minor) = 6.59 min.

Cut# : 1

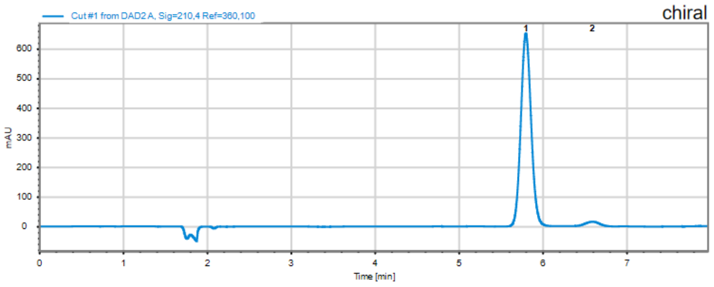

Signal: DAD2 A, Sig=210,4 Ref=360,100

Compound	Cut	Ret.Time	Area	Width	Height	Symmetry	
1	1	5.795	5554.372<br>**5554.372**	0.132	651.807	0.909	1st enantiomer
2	1	6.591	185.866<br>**185.866**	0.143	15.345	1.097	2nd enantiomer

ee = 93.5 %

Component table

Signal: DAD2 A, Sig=210,4 Ref=360,100

Component	¹D Sampling range [min]	Ret.Time ²D [min]	Area	Area%
1	13.23 - 13.27	5.795	5554.372	96.762
2	13.23 - 13.27	6.591	185.866	3.238

16: ee determination of pure major diastereomer

Cut# : 1

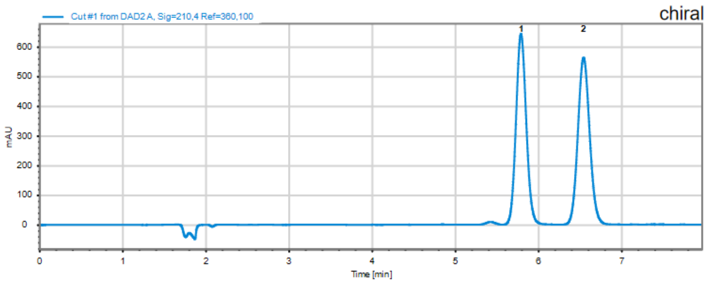

Signal: DAD2 A, Sig=210,4 Ref=360,100

Compound	Cut	Ret.Time	Area	Width	Height	Symmetry	
1	1	5.788	5500.581<br>**5500.581**	0.133	643.228	0.896	1st enantiomer
2	1	6.541	5574.800<br>**5574.800**	0.153	563.438	0.903	2nd enantiomer

rac-**16**: separation of enantiomers of pure major diastereomer

Methyl (5*R*,6*R*,*E*)-7-((*tert*-butyldiphenylsilyl)oxy)-6-hydroxy-5-methylhept-2-enoate (17).

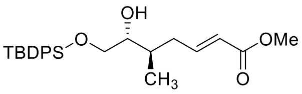

Prepared analogously according to the representative procedure followed by an oxidative work-up. Purified by flash chromatography (SiO_2, hexane/ethyl acetate 50:1 to 10:1) to give the title compound as a colorless oil (89 mg, 72% yield, >20:1 dr, 96% ee).

$[\alpha]_D^{20}$ = –2.2 (c = 0.50, $CHCl_3$) (lit. $[\alpha]_D^{19}$ = –3.8 (c = 0.48, $CHCl_3$));[10] ^1H NMR (400 MHz, $CDCl_3$) δ 7.71-7.61 (m, 4H), 7.47-7.36 (m, 6H), 6.94 (ddd, *J* = 15.3, 8.4, 6.7 Hz, 1H), 5.83 (dt, *J* = 15.6, 1.4 Hz, 1H), 3.74 (dd, *J* = 10.0, 3.4 Hz, 1H), 3.73 (s, 3H), 3.56 (dd, *J* = 10.1, 7.4 Hz, 1H), 3.46 (td, *J* = 7.5, 3.5 Hz, 1H), 2.62 (br s, 1H), 2.50 (dddd, *J* = 14.3, 6.7, 4.0, 1.7 Hz, 1H), 2.10 (app. dtd, *J* = 14.3, 8.6, 1.3 Hz, 1H), 1.80-1.70 (m, 1H), 1.07 (s, 9H), 0.77 (d, *J* = 6.8 Hz, 3H); ^{13}C NMR (101 MHz, $CDCl_3$) δ 167.1, 148.3, 135.7, 133.1, 130.1, 128.0, 122.5, 75.2, 66.1, 51.5, 35.5, 35.4, 27.0, 19.4, 15.6; IR (ATR): ṽ = 3503, 3071, 2955, 2931, 2857, 1722, 1656, 1428, 1314, 1268, 1171, 1108, 1044, 981, 822, 740, 700, 611; HRMS (ESI^+, *m/z*) calculated for $[C_{25}H_{34}O_4Si+Na]^+$ ($[M+Na]^+$) 449.2119, found 449.2118. The spectra match known data.[10]

The ee was determined by HPLC analysis: Chiralpak 150 mm IG-3, 4.6 mm i.D., acetonitrile/water = 60:40, v = 1.0 mL/min, λ = 220 nm, t(major) = 9.54 min, t(minor) = 10.72 min.

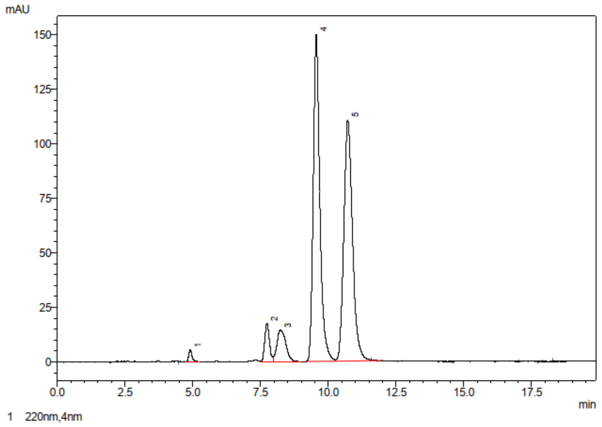

PDA Ch1 220nm

Peak #	Ret. Time	Area %	Name
1	4.91	0.79	
2	7.74	3.68	
3	8.24	5.85	
4	9.56	45.50	1st enantiomer
5	10.72	44.18	2nd enantiomer
Total		100.00	

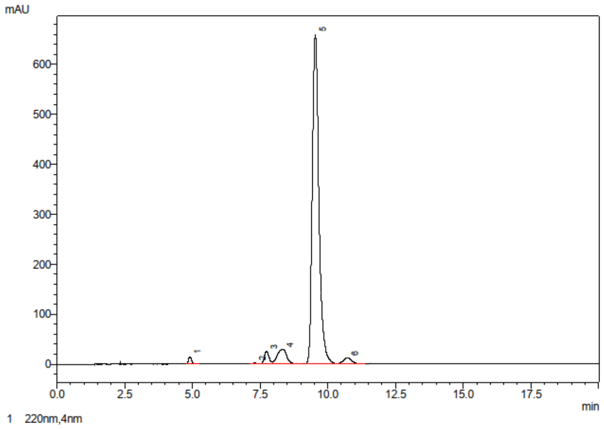

PDA Ch1 220nm

Peak #	Ret. Time	Area %	Name
1	4.91	0.94	
2	7.31	0.16	
3	7.74	2.44	
4	8.33	5.54	
5	9.54	88.87	1st enantiomer
6	10.72	2.05	2nd enantiomer
Total		100.00	

= 95.5 % ee

Methyl (5*R*,6*S*,*E*)-6-hydroxy-5,7-dimethyloct-2-enoate (18). Prepared analogously according to the representative procedure followed by an oxidative work-up. Purified by flash chromatography (SiO_2, toluene/*tert*-butyl methyl ether, 9:1) to give the title compound as a colourless oil (47 mg, 81% yield, >20:1 dr, 95% ee).

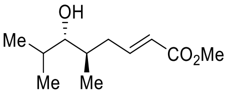

$[\alpha]_D^{20}$ = 9.6 (c = 0.5, $CHCl_3$); ^{1}H NMR (400 MHz, $CDCl_3$) δ = 6.99 (ddd, *J* = 15.5, 8.5, 6.5 Hz, 1H), 5.85 (dt, *J* = 15.5, 1.5 Hz, 1H), 3.72 (s, 3H), 3.11 (dd, *J* = 7.0, 5.0 Hz, 1H), 2.52 (dddd, *J* = 14.0, 6.5, 3.5, 1.5 Hz; 1H), 2.09 (app. dtd, *J* = 14.0, 9.0, 1.5 Hz, 1H), 1.86-1.72 (m, 2H), 0.94 (d, *J* = 7.0 Hz, 3H), 0.90 (d, *J* = 1.5 Hz, 3H), 0.88 (d, *J* = 1.5 Hz, 3H); ^{13}C NMR (101 MHz, $CDCl_3$) δ = 167.2, 148.9, 122.3, 80.4, 51.5, 35.7, 35.0, 30.1, 20.1, 16.5, 15.9; IR (ATR): ṽ = 3497, 2959, 2874, 1705, 1654, 1436, 1317, 1269, 1218, 1171, 1043, 979; HRMS (ESI, *m*/*z*) calculated for $[C_{11}H_{20}O_3+Na]^+$ ($[M+Na]^+$) 223.1305, found 223.1305.

The ee was determined by 2D HPLC analysis

Step 1: Purification: 250 mm YMC PVA-SIL, 4.6 mm i.D., *n*-heptane/isopropanol 98:2, v = 1.0 mL/min, λ = 210 nm, t(major) = 10.20 min, 308 K.

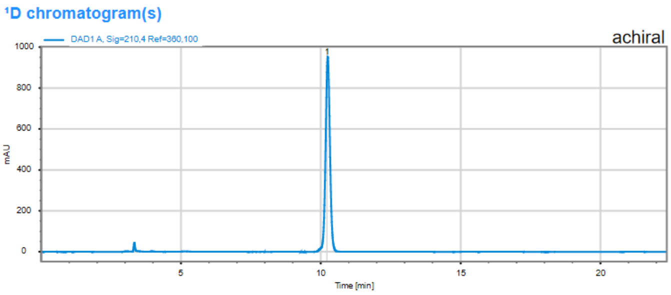

Sampling table (¹D)

Cut group	Cut #	¹D Cut start [min]	¹D Ret. time [min]	¹D Duration [min]	Trigger	²D Run start [min]	
	1	10.20	***	0.04	Time	10.25	major diastereomer

18: separation of impurities on achiral column

Step 2: Chiral resolution of the major diastereomer: Chiralcel 150 mm OD-3, 4.6 mm i.D., *n*-heptane/isopropanol = 95:5, v = 1.0 mL/min, λ = 210 nm, t(major) = 4.21 min, t(minor) = 5.07 min.

Cut# : 1

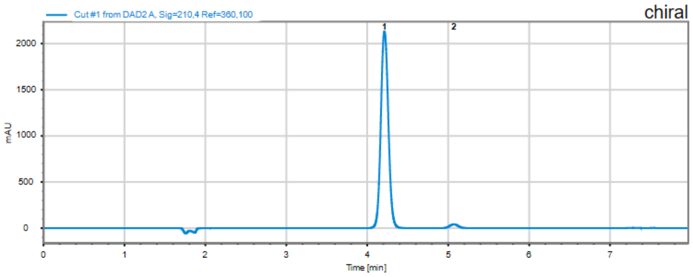

Signal: DAD2 A, Sig=210,4 Ref=360,100

Compound	Cut	Ret.Time	Area	Width	Height	Symmetry		
1	1	4.213	13738.150<br>**13738.150**	0.101	2130.377	0.920	1st enantiomer	ee = 95.4 %
2	1	5.070	321.228<br>**321.228**	0.116	42.035	0.883	2nd enantiomer	

Component table

Signal: DAD2 A, Sig=210,4 Ref=360,100

Component	¹D Sampling range [min]	Ret.Time ²D [min]	Area	Area%
1	10.20 - 10.24	4.213	13738.150	97.715
2	10.20 - 10.24	5.070	321.228	2.285

18: ee determination of pure major diastereomer

Cut# : 1

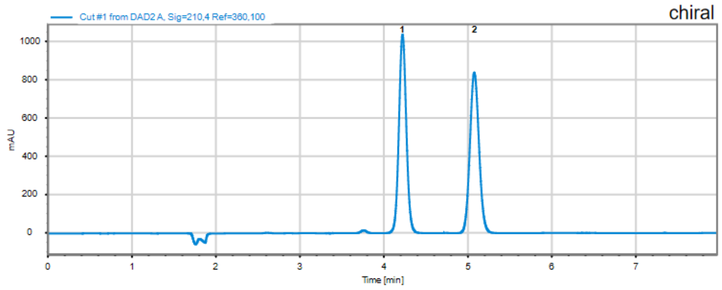

Signal: DAD2 A, Sig=210,4 Ref=360,100

Compound	Cut	Ret.Time	Area	Width	Height	Symmetry	
1	1	4.222	6375.557<br>**6375.557**	0.095	1037.297	0.927	1st enantiomer
2	1	5.076	6348.159<br>**6348.159**	0.118	839.594	0.909	2nd enantiomer

rac-**18**: separation of enantiomers of pure major diastereomer

(5*R*,6*R*,*E*)-6-Hydroxy-5-methyl-6-phenylhex-2-enoyl fluoride (20). Prepared analogously according to the representative procedure followed by an acidic work-up. Purified by flash chromatography (SiO_2, hexane/ethyl acetate 95:5 to 88:12 to 4:1) to give the title compound as a colourless oil (40 mg, 62% yield, >20:1 dr, 89% ee).

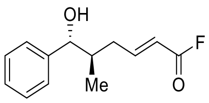

$[\alpha]_D^{20}$ = 25.2 (c = 0.53, $CHCl_3$); ^1H NMR (400 MHz, $CDCl_3$) δ = 7.39-7.27 (m, 5H), 7.22 (ddd, *J* = 15.5, 8.5, 6.5 Hz, 1H), 5.82 (ddt, *J* = 15.5, 8.5, 1.5 Hz, 1H), 4.43 (d, *J* = 7.5 Hz, 1H), 2.69-2.61 (m, 1H), 2.31-2.21 (m, 1H), 2.11-2.00 (m, 1H), 1.95 (br. s, 1H, -OH), 0.80 (d, *J* = 7.0 Hz, 3H); ^{13}C NMR (101 MHz, $CDCl_3$) δ = 156.7 (d, $^3J_{CF}$ = 5.7 Hz), 156.4 (d, $^1J_{CF}$ = 340.8 Hz), 143.0, 128.7, 128.1, 126.6, 117.5 (d, $^2J_{CF}$ = 65.3 Hz), 78.5, 39.7, 35.8, 16.4; ^{19}F NMR (376 MHz, $CDCl_3$) δ = 19.7; IR (ATR): ṽ = 3411, 3031, 2965, 2878, 1800, 1643, 1214, 1099, 1020, 981, 762, 701; HRMS (EI, *m/z*) calculated for $[C_{13}H_{15}FO_2]^+$ ($[M^+]$) 222.1051, found 222.1051.

The ee was determined by HPLC analysis: Chiralcel 150 mm IH-3, 4.6 mm i.D., *n*-heptane/isopropanol = 98:2, v = 1.0 mL/min, λ = 210 nm, t(major) = 11.63 min, t(minor) = 12.62 min.

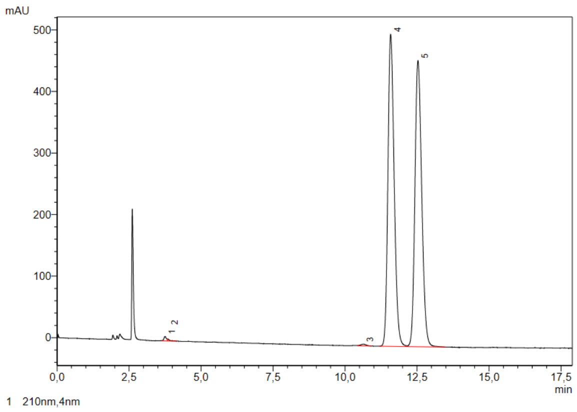

PDA Ch1 210nm

Peak #	Ret. Time	Area %	Name
1	3,75	0,29	
2	3,84	0,11	
3	10,63	0,25	
4	11,58	49,66	1. Enantiomer
5	12,53	49,69	2. Enantiomer
Total		100,00	

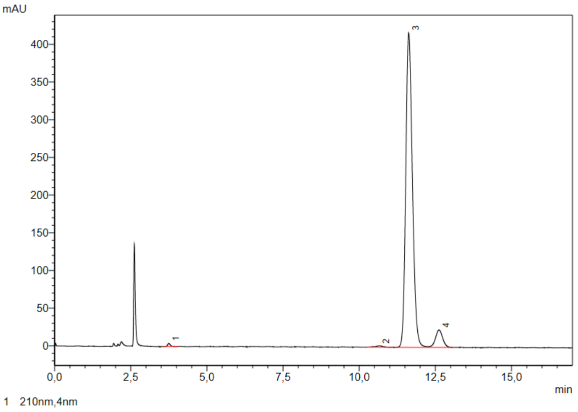

PDA Ch1 210nm

Peak #	Ret. Time	Area %	Name
1	3,75	0,40	
2	10,66	0,49	
3	11,63	93,42	1. Enantiomer 88.5 % ee
4	12,62	5,70	2. Enantiomer
Total		100,00	

(5*R*,6*S*,*E*)-6-Hydroxy-5-methyl-1-morpholino-8-phenyloct-2-en-1-one (22). Prepared analogously according to the representative procedure followed by an oxidative work-up. Purified by flash chromatography (SiO_2, hexane/ethyl acetate 1:1 to 1:9) to give the title compound as a colorless oil (64 mg, 70% yield, >20:1 dr, 96% ee).

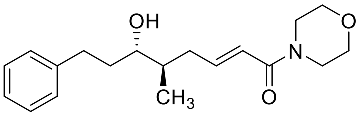

$[\alpha]_D^{20}$ = –18.4 (c = 0.50, $CHCl_3$); ^{1}H NMR (400 MHz, $CDCl_3$) δ 7.30-7.24 (m, 2H), 7.22-7.15 (m, 3H), 6.88 (ddd, *J* = 15.0, 8.1, 6.8 Hz, 1H), 6.19 (dt, *J* = 14.9, 1.4 Hz, 1H), 3.66 (br s, 6H), 3.52 (br s, 2H), 3.46 (ddd, *J* = 9.0, 5.8, 3.0 Hz, 1H), 2.84 (ddd, *J* = 13.7, 10.0, 5.4 Hz, 1H), 2.64 (ddd, *J* = 13.7, 9.7, 6.6 Hz, 1H), 2.43 (dddd, *J* = 14.1, 6.8, 4.3, 1.6 Hz, 1H), 2.08 (app. dtd, *J* = 14.2, 8.7, 1.3 Hz, 1H), 1.81 (dddd, *J* = 13.9, 9.9, 6.7, 3.0 Hz, 1H), 1.78-1.63 (m, 2H), 0.91 (d, *J* = 6.8 Hz, 3H); ^{13}C NMR (101 MHz, $CDCl_3$) δ 165.7, 146.1, 142.2, 128.5, 128.5, 126.0, 120.8, 74.9, 66.9 (2C), 46.2, 42.4, 38.8, 35.9, 35.5, 32.4, 15.9; IR (ATR): ṽ = 3411, 3025, 2960, 2922, 2857, 1736, 1654, 1601, 1495, 1433, 1373, 1300, 1265, 1236, 1113, 1043, 976, 918, 849, 700, 575; HRMS (ESI^+, *m*/*z*) calculated for $[C_{19}H_{27}NO_3]^+$ ($[M^+]$) 317.1985, found 317.1986.

The ee was determined by HPLC analysis: Chiralcel 150 mm OJ-3R, 4.6 mm i.D., methanol/water = 75:25, v = 0.5 mL/min, λ = 230 nm, t(minor) = 13.28 min, t(major) = 14.54 min.

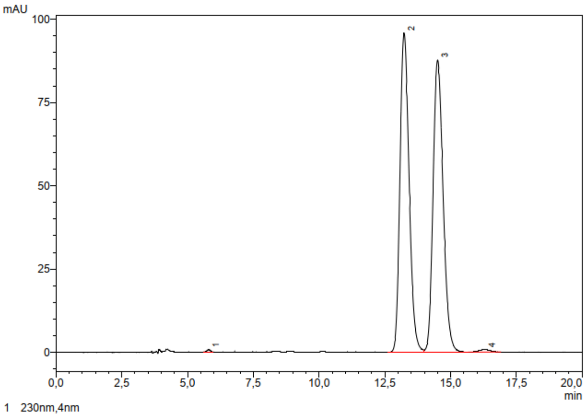

PDA Ch1 230nm

Peak #	Ret. Time	Area %	Name
1	5,80	0,14	
2	13,23	49,57	1. Enantiomer
3	14,51	49,81	2. Enantiomer
4	16,30	0,48	
Total		100,00	

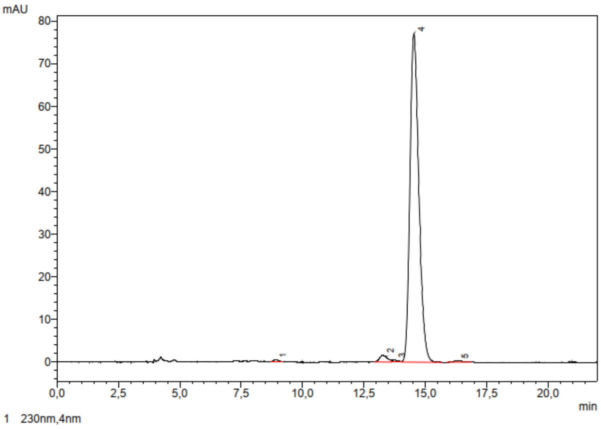

PDA Ch1 230nm

Peak #	Ret. Time	Area %	Name
1	8,90	0,35	
2	13,28	1,74	1. Enantiomer
3	13,75	0,40	
4	14,54	97,08	2. Enantiomer 96.5 % ee
5	16,32	0,44	
Total		100,00	

(5*R*,6*R*,*E*)-6-hydroxy-5-methyl-6-phenylhex-2-enoyl fluoride (**24a**). Prepared analogously according to the representative procedure (except at half scale with 5 mol% catalyst and **L1**) followed by an oxidative work-up. Purified by flash chromatography (SiO_2, hexane/ethyl acetate 1:1 to 1:2 to 1:3) to give the title compound as a colourless oil (33 mg, 61% yield, >20:1 dr, 93% ee).

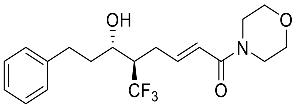

$[\alpha]_D^{20}$ = –7.2 (c = 0.5, $CHCl_3$); ^1H NMR (400 MHz, $CDCl_3$) δ = 7.31-7.25 (m, 2H), 7.23-7.16 (m, 3H), 6.83 (dt, *J* = 15.0, 7.5, Hz, 1H), 6.24 (dt, *J* = 15.0, 1.5 Hz, 1H), 4.03 (app. dt, *J* = 9.5, 3.0 Hz, 1H), 3.66 (br. s, 6H), 3.49 (br. s, 2H), 2.84 (app. ddd, *J* = 14.5, 9.5, 5.5 Hz, 1H), 2.66 (app. ddd, *J* = 14.0, 9.0, 7.0 Hz, 1H), 2.60-2.54 (m, 2H), 2.31 (app. qtt, *J* = 8.5, 6.0, 2.5 Hz, 1H), 1.91 (app. ddt, *J* = 14.5, 9.0, 4.5 Hz, 1H), 1.78 (dddd, *J* = 14.0, 9.5, 7.0, 3.5 Hz, 1H) (2 broad signals for morpholine protons due to restricted rotation about amide C–N bond); ^{13}C NMR (101 MHz, $CDCl_3$) δ = 165.3, 143.2, 141.2, 128.7, 128.5, 127.6 (q, $^1J_{CF}$ = 282.4 Hz), 126.3, 122.0, 68.3 (q, $^3J_{CF}$ = 2.6 Hz), 66.9 (br. s), 48.2 (q, $^2J_{CF}$ = 23.1 Hz), 46.2 (br. s), 42.4 (br. s), 36.7, 32.3, 26.7 (q, $^3J_{CF}$ = 2.5 Hz) (2 carbons attached to nitrogen appear in different environments due to restricted rotation about amide C–N bond); ^{19}F NMR (282 MHz, $CDCl_3$) δ = –67.4; IR (ATR): ṽ = 3407, 2824, 2859, 1658, 1603, 1436, 1266, 1238, 1154, 1113, 910, 729, 573; HRMS (ESI, *m*/*z*) calculated for $[C_{19}H_{25}NO_3F_3]^+$ ($[M^+]$) 372.1781, found 372.1779.

The ee was determined by HPLC analysis: Chiralcel 150 mm IB-N-3, 4.6 mm i.D., *n*-heptane/isopropanol = 70:30, v = 1.0 mL/min, λ = 210 nm, t(minor) = 5.24 min, t(major) = 7.40 min.

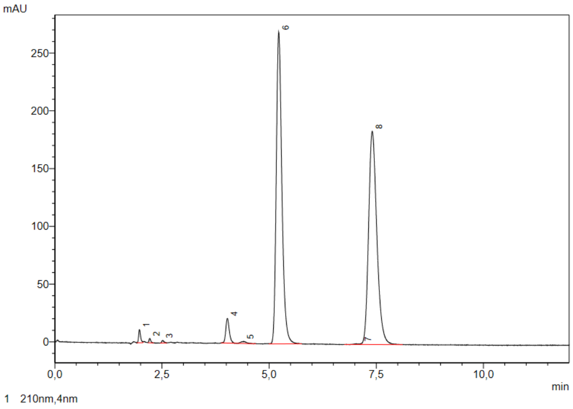

PDA Ch1 210nm

Peak #	Ret. Time	Area %	Name
1	1,97	0,71	
2	2,21	0,21	
3	2,51	0,15	
4	4,03	2,39	
5	4,40	0,32	
6	5,22	47,99	1. Enantiomer
7	7,15	0,12	
8	7,41	48,10	2. Enantiomer
Total		100,00	

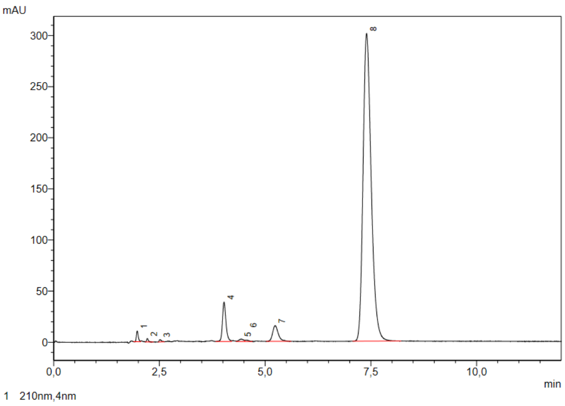

PDA Ch1 210nm

Peak #	Ret. Time	Area %	Name
1	1,97	0,73	
2	2,21	0,20	
3	2,52	0,18	
4	4,03	4,98	
5	4,43	0,38	
6	4,56	0,18	
7	5,24	3,19	1. Enantiomer
8	7,40	90,17	2. Enantiomer 93.2 % ee
Total		100,00	

(*R*,*E*)-6,6,6-trifluoro-5-((*R*)-hydroxy(phenyl)methyl)-1-morpholinohex-2-en-1-one (**24b**).

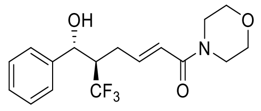

Prepared analogously according to the representative procedure (except that the mixture was stirred at –20 °C for 2 d at half scale with 10 mol% catalyst and **L1**) followed by an oxidative work-up. Purified by flash chromatography (SiO_2, hexane/ethyl acetate 3:2 to 1:1 to 1:3) to give the title compound as a light yellow oil (26 mg, 52% yield, >20:1 dr, 86% ee).

$[\alpha]_D^{20}$ = 9.3 (c = 0.55, $CHCl_3$); ^{1}H NMR (400 MHz, $CDCl_3$) δ = 7.97 (d, J = 8.5 Hz, 2H), 7.36 (d, J = 8.5 Hz, 2H), 6.93 (ddd, J = 15.5, 8.5, 7.0 Hz, 1H), 5.82 (dt, J = 15.5, 1.5 Hz, 1H), 4.49 (d, J = 7.0 Hz, 1H), 3.89 (s, 3H), 3.69 (s, 3H), 2.48 (dddd, J = 14.0, 7.0, 4.0, 1.5 Hz, 1H), 2.14 (dtd, J = 14.0, 8.5, 1.5 Hz, 1H), 2.03-1.94 (m, 1H), 0.76 (d, J = 7.0 Hz, 3H); ^{13}C NMR (101 MHz, $CDCl_3$) δ = 165.4, 143.1, 141.5, 128.7, 128.0, 127.3 (q, $^1J_{CF}$ = 283.0 Hz), 125.9, 121.6, 70.3 ($^3J_{CF}$ = 3.0 Hz), 66.8, 50.2 ($^2J_{CF}$ = 23.2 Hz), 46.2 (br. s), 42.3 (br. s), 25.8 ($^3J_{CF}$ = 2.3 Hz) (2 carbons attached to nitrogen appear in different environments due to restricted rotation about amide C–N bond); ^{19}F NMR (282 MHz, $CDCl_3$) δ = –66.9; IR (ATR): ṽ = 3328, 2971, 2861, 1657, 1574, 1458, 1436, 1255, 1161, 1111, 1067, 1025, 850, 704; HRMS (ESI, *m*/*z*) calculated for $[C_{17}H_{20}NO_3F_3+Na]^+$ ($[M+Na]^+$) 366.1288, found 366.1283.

The ee was determined by HPLC analysis: Chiralcel 150 mm IH-3, 4.6 mm i.D., n-heptane/isopropanol = 90:10, v = 1.0 mL/min, λ = 230 nm, t(major) = 18.44 min, t(minor) = 23.82 min.

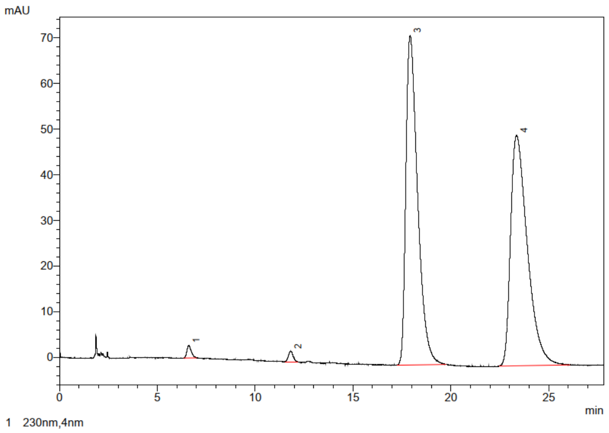

PDA Ch1 230nm

Peak #	Ret. Time	Area %	Name
1	6,61	0,71	
2	11,82	0,64	
3	17,92	49,29	1. Enantiomer
4	23,36	49,35	2. Enantiomer
Total		100,00	

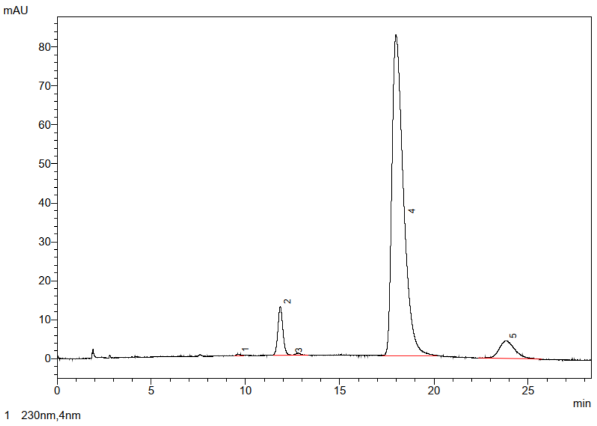

PDA Ch1 230nm

Peak #	Ret. Time	Area %	Name
1	9,61	0,16	
2	11,84	5,79	
3	12,45	0,25	
4	18,44	87,14	1. Enantiomer 85.8 % ee
5	23,82	6,66	2. Enantiomer
Total		100,00	

(3*S*,4*R*,*E*)-4-Methyl-1-phenyl-7-(4,4,5,5-tetramethyl-1,3,2-dioxaborolan-2-yl)hept-6-en-3-ol (26a). Prepared analogously according to the representative procedure (except that 1.5 equiv of the aldehyde and 1.0 equiv of diene **25** were used) followed by a basic work-up (stirring at 0 °C for 3 h). Purified by flash chromatography (SiO_2, hexane/ethyl acetate 50:1 to 5:1) to give the title compound as a colorless oil (43 mg, 45% yield, >20:1 dr, 92% ee).

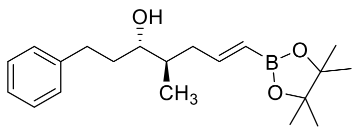

$[\alpha]_D^{20}$ = –24.2 (c = 0.40, $CHCl_3$); ^{1}H NMR (400 MHz, $CDCl_3$) δ 7.31-7.26 (m, 2H), 7.23-7.18 (m, 3H), 6.60 (ddd, *J* = 17.9, 7.5, 6.3 Hz, 1H), 5.45 (dt, *J* = 17.9, 1.5 Hz, 1H), 3.48 (ddd, *J* = 8.9, 5.5, 3.0 Hz, 1H), 2.84 (ddd, *J* = 13.7, 10.1, 5.3 Hz, 1H), 2.64 (ddd, *J* = 13.7, 9.8, 6.6 Hz, 1H), 2.37 (dddd, *J* = 14.2, 6.3, 4.6, 1.7 Hz, 1H), 2.08-1.98 (m, 1H), 1.81 (dddd, *J* = 13.9, 9.9, 6.6, 3.0 Hz, 1H), 1.75-1.66 (m, 2H), 1.26 (s, 12H), 0.90 (d, *J* = 6.9 Hz, 3H); ^{13}C NMR (101 MHz, $CDCl_3$) δ 153.1, 142.4, 128.6, 128.6, 126.0, 83.2, 75.2, 39.1, 38.7, 35.7, 32.5, 24.9, 15.7; IR (ATR): ṽ = 3444, 3026, 2976, 2929, 1636, 1454, 1360, 1317, 1265, 1142, 1097, 1031, 996, 969, 848, 747, 698; HRMS (ESI$^+$, *m*/*z*) calculated for $[C_{20}H_{31}BO_3+Na]^+$ ($[M+Na]^+$) 353.2258, found 353.2261.

The ee was determined by 2D HPLC analysis

Step 1: Purification: 50 mm Zorbax Eclipse Plus C18, 4.6 mm i.D., acetonitrile/water gradient 50% to 80 over 5 minutes, v = 1.0 mL/min, λ = 220 nm, t(major) = 2.16 min, 308 K.

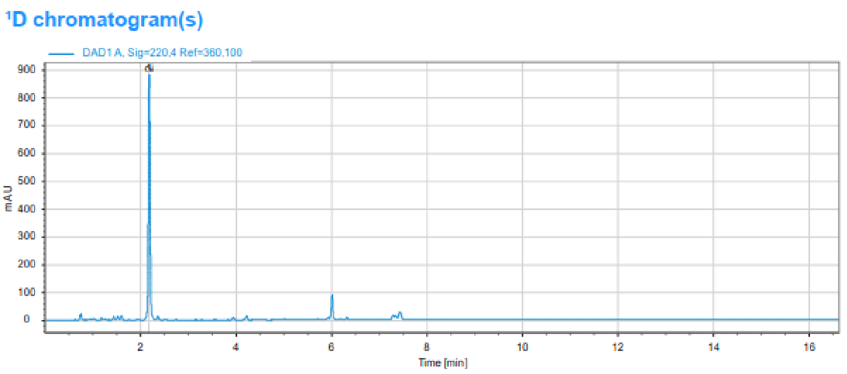

Sampling table (¹D)

Cut group	Cut #	¹D Cut start [min]	¹D Ret. time [min]	¹D Duration [min]	Trigger	²D Run start [min]
	1	2.16	2.189	0.04	Time	2.21

26a: separation of impurities on achiral column

Step 2: Chiral resolution of the major diastereomer: Daicel 150 mm Chiralpak IB-N3, 4.6 mm i.D., acetonitrile/water = 65:35, v = 1.0 mL/min, λ = 220 nm, t(major) = 4.74 min, t(minor) = 5.47 min.

Cut# : 1

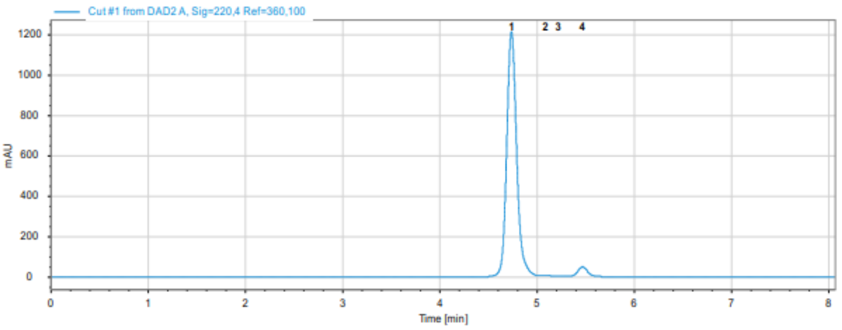

Signal: DAD2 A, Sig=220,4 Ref=360,100

Compound	Cut	Ret.Time	Area	Width	Height	Symmetry
1	1	4.738	8627.473<br>**8627.473**	0.110	1212.277	0.843
2	1	5.086	44.269<br>**44.269**	0.092	6.946	0.849
3	1	5.219	7.739<br>**7.739**	0.056	1.651	0.727
4	1	5.470	340.569<br>**340.569**	0.110	47.746	0.880

Component table

Signal: DAD2 A, Sig=220,4 Ref=360,100

Component	¹D Sampling range [min]	Ret.Time ²D [min]	Area	Area%	
1	2.16 - 2.20	4.738	8627.473	95.648	1. enantiomer
2	2.16 - 2.20	5.086	44.269	0.491	
3	2.16 - 2.20	5.219	7.739	0.086	
4	2.16 - 2.20	5.470	340.569	3.776	2. enantiomer

ee = 92.4%

26a: ee determination of pure major diastereomer

Cut# : 1

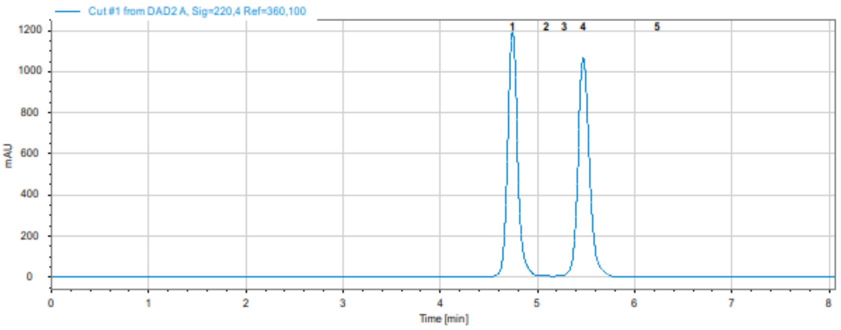

Signal: DAD2 A, Sig=220,4 Ref=360,100

Compound	Cut	Ret.Time	Area	Width	Height	Symmetry
1	1	4.747	8428.767<br>**8428.767**	0.110	1190.766	0.859
2	1	5.093	33.745<br>**33.745**	0.079	5.510	0.914
3	1	5.277	48.879<br>**48.879**	0.080	10.176	0.000
4	1	5.476	8711.042<br>**8711.042**	0.136	1065.108	0.857
5	1	6.233	24.177<br>**24.177**	0.103	3.122	0.932

rac-**26a**: separation of enantiomers of pure major diastereomer

(1*R*,2*R*,*E*)-2-Methyl-1-phenyl-5-(4,4,5,5-tetramethyl-1,3,2-dioxaborolan-2-yl)pent-4-en-1-ol (26b). Prepared analogously according to the representative procedure (except that the mixture was stirred at –20°C) followed by a basic work-up (stirring at 0 °C for 3 h). Purified by flash chromatography (SiO_2, hexane/ethyl acetate 50:1 to 10:1) to give the title compound as a pale yellow oil (54 mg, 62% yield, >20:1 dr, 92% ee).

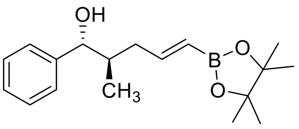

$[\alpha]_D^{20}$ = 5.9 (c = 0.51, $CHCl_3$); ^1H NMR (400 MHz, $CDCl_3$) δ 7.36-7.24 (m, 5H), 6.64 (ddd, *J* = 17.9, 7.4, 6.2 Hz, 1H), 5.48 (dt, *J* = 17.8, 1.5 Hz, 1H), 4.44 (d, *J* = 6.8 Hz, 1H), 2.56-2.47 (m, 1H), 2.10-2.03 (m, 1H), 2.03-1.95 (m, 1H), 1.26 (s, 12H), 0.76 (d, *J* = 6.6 Hz, 3H); ^{13}C NMR (101 MHz, $CDCl_3$) δ 153.0, 143.3, 128.3, 127.6, 126.8, 83.1, 78.5, 39.8, 39.1, 24.9, 24.9, 16.0; IR (ATR): ṽ = 3441, 2978, 2929, 2874, 1637, 1455, 1362, 1317, 1216, 1143, 996, 969, 848, 749, 701, 667; HRMS (ESI$^+$, *m*/*z*) calculated for $[C_{18}H_{27}O_3B]^+$ ($[M^+]$) 302.2048, found 302.2048.

The ee was determined by HPLC analysis: Chiralpak 150 mm IC-3, 4.6 mm i.D., *n*-heptane/*i*-propanol = 98:2, v = 1.0 mL/min, λ = 210 nm, t(major) = 7.63 min, t(minor) = 8.99 min.

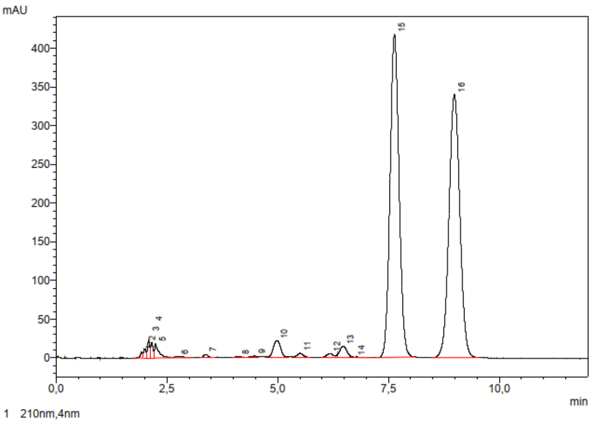

PDA Ch1 210nm

Peak #	Ret. Time	Area %	Name
1	1,93	0,23	
2	2,00	0,36	
3	2,09	0,63	
4	2,16	0,63	
5	2,24	0,98	
6	2,74	0,20	
7	3,38	0,29	
8	4,11	0,13	
9	4,48	0,18	
10	4,98	2,03	
11	5,51	0,45	
12	6,18	0,39	
13	6,48	1,38	
14	6,73	0,06	
15	7,63	46,46	1. Enantiomer
16	8,99	45,59	2. Enantiomer
Total		100,00	

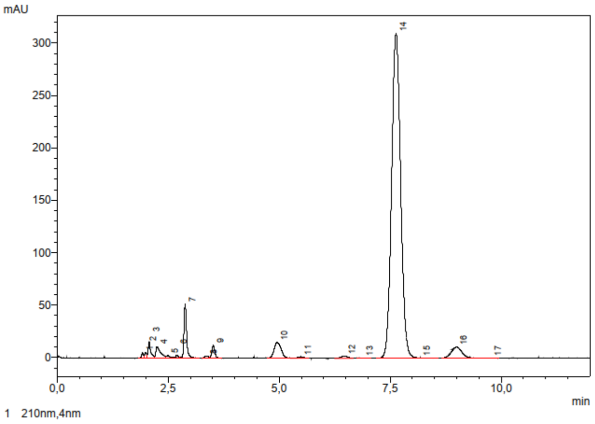

PDA Ch1 210nm

Peak #	Ret. Time	Area %	Name
1	1,92	0,32	
2	2,00	0,35	
3	2,07	1,17	
4	2,24	1,59	
5	2,49	0,29	
6	2,69	0,25	
7	2,88	4,08	
8	3,36	0,24	
9	3,51	1,12	
10	4,95	3,17	
11	5,48	0,25	
12	6,47	0,45	
13	6,87	0,13	
14	7,63	82,94	1. Enantiomer 92.0 % ee
15	8,16	0,10	
16	8,99	3,44	2. Enantiomer
17	9,76	0,11	
Total		100,00	

Preparation of the Diene Substrates

(2*E*,4*Z*)-Hexa-2,4-dienoic acid (S1). Palladium acetate (0.37 g, 1.6 mmol, 5.0 mol%) was added to a stirred solution of *tert*-butyl acrylate (9.6 mL, 66 mmol), *cis*-bromopropene (2.8 mL, 33 mmol), K_2CO_3 (11 g, 82 mmol), triphenylphosphine (0.86 g, 3.3 mmol, 0.10 equiv) and tetrabutylammonium chloride (9.1 g, 33 mmol) in CH_3CN/H_2O (10:1, 50 mL). The mixture was stirred at 50 °C (bath temperature) for 48 h, then allowed to cool to room temperature and filtered through a pad of Celite. The filtrate was washed with water (150 mL) and brine (150 mL), dried over Na_2SO_4, filtered and concentrated *in vacuo* to afford a dark-red oil. The residue was purified by flash chromatography (SiO_2, *tert*-butyl methyl ether/pentane 1:100) to afford (2*E*,4*Z*)-*tert*-butyl hex-2,4-dienoate as a colorless oil (1.5 g, 27% yield).

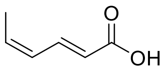

Trifluoroacetic acid (10 mL) was added dropwise to a stirred solution of (2*E*,4*Z*)-*tert*-butyl hex-2,4-dienoate (2.52 g, 15.0 mmol) in anhydrous dichloromethane (10 mL) at 0 °C, and stirring was continued for 30 min at room temperature before all volatile materials were removed under reduced pressure to afford an orange oil. The residue was purified by flash chromatography (SiO_2, *tert*-butyl methyl ether/hexane 1:20 to 1:4) to afford the title acid as colorless oil (1.45 g, 86% yield). ^{1}H NMR (400 MHz, $CDCl_3$) δ 7.73 (ddd, *J* = 15.2, 11.6, 1.1 Hz, 1H), 6.25-6.13 (m, 2H), 6.00 (dqt, *J* = 10.7, 7.3, 0.9 Hz, 1H), 5.87 (dq, *J* = 15.3, 0.7 Hz, 1H), 1.91 (ddd, *J* = 7.3, 1.7, 0.5 Hz, 3H); ^{13}C NMR (101 MHz, $CDCl_3$) δ 172.2, 141.6, 137.3, 127.4, 120.1, 14.3. Matches known data.[11]

Methyl (2*E*,4*Z*)-hexa-2,4-dienoate (*E,Z*-5). $TMSCHN_2$ (0.6 M in hexanes, 24 mL, 14 mmol) was added dropwise to a stirred solution of (2*E*,4*Z*)-hexa-2,4-dienoic acid **S1** (1.4 g, 13 mmol) in anhydrous toluene and methanol (3.5:1 *v*/*v*, 45 mL). After stirring for 75 min at room temperature, the mixture was concentrated *in vacuo* to give a yellow oil. The residue was purified by flash chromatography (SiO_2, hexane 100%) to afford the desired product as a colorless oil (0.72 g, 44% yield; mixture of isomers).

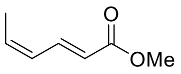

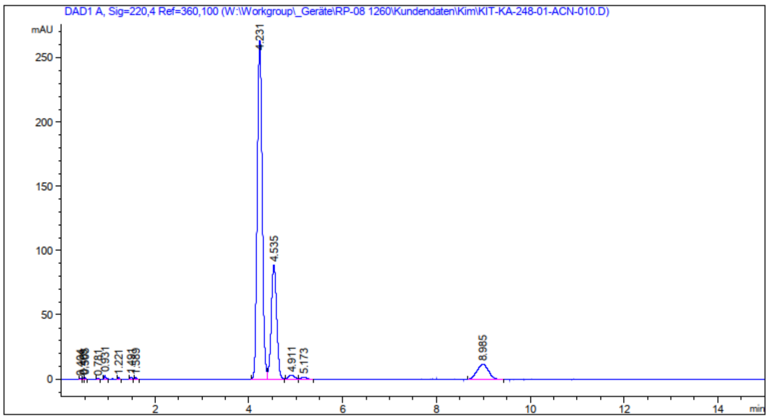

Peak #	RetTime [min]	Type	Width [min]	Area [mAU*s]	Height [mAU]	Area %
1	0.404	BV	0.0295	1.47236	7.45932e-1	0.0512
2	0.469	VV	0.0165	1.32172	1.23679	0.0460
3	0.505	VB	0.0185	1.86363	1.44967	0.0649
4	0.781	BB	0.0282	1.03190	5.42277e-1	0.0359
5	0.931	BB	0.0285	5.44127	2.94997	0.1894
6	1.221	BB	0.0287	1.95726	1.07683	0.0681
7	1.491	BV	0.0370	4.01936	1.64551	0.1399
8	1.589	VB	0.0398	4.00497	1.54198	0.1394
9	4.231	BV	0.1120	1898.49292	263.61636	66.0677
10	4.535	VB	0.1241	710.12347	89.03059	24.7124
11	4.911	BV	0.1340	31.06180	3.26355	1.0810
12	5.173	VB	0.1159	15.87358	1.70844	0.5524
13	8.985	BB	0.2592	196.89070	11.67767	6.8518
Totals :				2873.55495	380.48558	

Isomerically pure ***E*/*Z*-5** was obtained by preparative HPLC: 50 mm Zorbax Eclipse Plus C18, 4.6 mm i.D., acetonitrile/water = 30:70, v = 1.0 mL/min, λ = 220 nm, t(major) = 4.23 min, t(minor) = 4.54 min.

^{1}H NMR (400 MHz, $CDCl_3$) δ 7.64 (ddd, *J* = 15.3, 11.6, 1.0 Hz, 1H), 6.19-6.10 (m, 1H), 5.99-5.90 (m, 1H), 5.89-5.83 (m, 1H), 3.75 (s, 3H), 1.89 (ddd, *J* = 7.3, 1.7, 0.6 Hz, 3H); ^{13}C NMR (101 MHz, $CDCl_3$) δ 167.9, 139.6, 136.1, 127.5, 120.7, 51.7, 14.2; IR (ATR): ṽ = 3026, 2991, 2950, 2853, 1715, 1638, 1607, 1434, 1309, 1267, 1173, 1132, 1036, 993, 867, 691, 616; HRMS (ESI$^+$, *m*/*z*) calculated for $[C_7H_{10}O_2]^+$ ([M$^+$]) 126.0675, found 126.0678.

Methyl (2*E*,4*E*)-hepta-2,4-dienoate (S2). To a stirred solution of lithium chloride (0.51 g, 12 mmol) in CH_3CN (50 mL) was added trimethyl phosphonoacetate (2.2 mL, 15 mmol) and 1,8-diazabicyclo(5.4.0)undec-7-ene (1.8 mL, 12 mmol). The mixture was stirred for 10 min at room temperature before a solution of (*E*)-2-pentenal (0.98 mL, 10 mmol) in CH_3CN (10 mL) was added and stirring was continued for 3 h. The reaction was quenched with saturated NH_4Cl (50 mL) and the aqueous layer was extracted with ethyl acetate (2 × 75 mL). The organic layer was dried over Na_2SO_4, filtered and concentrated under reduced pressure to afford a pale yellow oil, which was purified by flash chromatography (SiO_2, ethyl acetate/hexane 1:100 to 1:20) to afford the title compound as a pale yellow oil (0.53 g, 38% yield). ^{1}H NMR (400 MHz, $CDCl_3$) δ 7.32-7.22 (m, 1H), 6.19-6.16 (m, 2H), 5.79 (d, *J* = 15.5 Hz, 1H), 3.74 (s, 3H), 2.23-2.16 (m, 2H), 1.05 (t, *J* = 7.4 Hz, 3H); ^{13}C NMR (101 MHz, $CDCl_3$) δ 167.9, 146.3, 145.6, 127.5, 118.9, 51.6, 26.2, 13.0; IR (ATR): ṽ = 3022, 2967, 2876, 1716, 1643, 1617, 1434, 1348, 1329, 1303, 1260, 1233, 1192, 1140, 1040, 997, 875, 722; HRMS (ESI$^+$, *m*/*z*) calculated for $[C_8H_{12}O_2]^+$ ([M$^+$]) 140.0832, found 140.0833. Matches known data.[12]

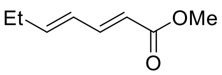

***tert*-Butyldimethyl(pent-4-yn-1-yloxy)silane (S3).** *tert*-Butyldimethylsilyl chloride (2.2 mL, 18 mmol) and triethylamine (3.1 mL, 22 mmol) were added to a stirred solution of 4-pentyn-1-ol (1.4 mL, 15 mmol) in dichloromethane (19 mL) at 0 °C. The mixture was stirred for 20 h at room temperature before it was filtered through a pad of Celite and the filtrate was concentrated under reduced pressure to afford yellow oil. The residue was purified by flash chromatography (SiO_2, hexanes) to afford the desired product as a colorless oil (2.4 g, 81% yield). ^{1}H NMR (400 MHz, $CDCl_3$) δ 3.70 (t, *J* = 6.0 Hz, 2H), 2.27 (td, *J* = 7.1, 2.7 Hz, 2H), 1.93 (t, *J* = 2.7 Hz, 1H), 1.73 (tt, *J* = 7.1, 6.0 Hz, 2H), 0.89 (s, 9H), 0.06 (s, 6H); ^{13}C NMR (101 MHz, $CDCl_3$) δ 84.4, 68.4, 61.6, 31.7, 26.1, 18.5, 15.0, –5.2. Matches known data.[13]

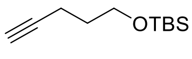

Methyl 6-((*tert*-butyldimethylsilyl)oxy)hex-2-ynoate (S4). *n*-BuLi (1.6 M in hexane. 9.9 mL, 16 mmol) was added to a solution of the *tert*-butyldimethyl(pent-4-yn-1-yloxy)silane **S3** (2.4 g, 12 mmol) in THF (35 mL) at –78 °C. The mixture was stirred for 30 min prior to slow addition of ethyl chloroformate (1.4 mL, 18 mmol). The mixture was then warmed gradually to room temperature and stirring was continued for 15 h. The reaction was quenched with sat. NH_4Cl solution (5 mL), the aqueous phase was extracted with *tert*-butyl methyl ether (3 × 50 mL), the combined organic layers were washed with water (20 mL) and brine (20 mL), dried over Na_2SO_4, filtered and concentrated *in vacuo* to give a pale yellow oil. The residue was purified by flash chromatography (SiO_2, *tert*-butyl methyl ether/hexane 1:100 to 1:20) to afford the title compound as a colorless oil (2.9 g, 94% yield). ^1H NMR (400 MHz, $CDCl_3$) δ 3.75 (s, 3H), 3.72-3.65 (m, 2H), 2.43 (t, *J* = 7.1 Hz, 2H), 1.77 (tt, *J* = 7.2, 5.9 Hz, 2H), 0.89 (s, 9H), 0.05 (s, 6H); ^{13}C NMR (101 MHz, $CDCl_3$) δ 154.4, 89.7, 73.0, 61.3, 52.7, 35.0, 30.7, 26.0, 15.3, –5.3.

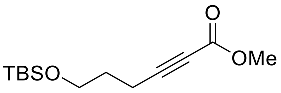

Methyl (2*E*,4*E*)-6-((*tert*-butyldimethylsilyl)oxy)hexa-2,4-dienoate (S5). Triphenylphosphine (3.0 g, 11 mmol) and phenol (1.0 mL, 11 mmol) were added to a stirred solution of methyl 6-((*tert*-butyldimethylsilyl)oxy)hex-2-ynoate **S4** (2.9 g, 11 mmol) in anhydrous benzene (30 mL). After stirring for 12 h, the mixture was diluted with diethyl ether (10 mL) before aq. NaOH solution (1 M, 10 mL) was slowly added. The aqueous layer was extracted with diethyl ether (3 × 40 mL). The combined organic phases were dried over Na_2SO_4, filtered and concentrated in vacuo to afford a red-brown oil. The residue was purified by flash chromatography (SiO_2, ethyl acetate/hexane 1:100) to afford title compound as a pale yellow oil (0.57 g, 19% yield). ^1H NMR (400 MHz, $CDCl_3$) δ 7.30 (ddd, *J* = 15.4, 11.1, 0.7 Hz, 1H), 6.40 (ddtd, *J* = 15.0, 11.0, 1.9, 0.7 Hz, 1H), 6.17 (dtt, *J* = 15.1, 4.3, 0.7 Hz, 1H), 5.87 (dt, *J* = 15.3, 0.8 Hz, 1H), 4.30 (ddt, *J* = 4.3, 1.9, 0.7 Hz, 2H), 3.74 (s, 3H), 0.92 (s, 9H), 0.08 (s, 6H); ^{13}C NMR (101 MHz, $CDCl_3$) δ 167.7, 144.5, 142.2, 126.9, 120.4, 63.0, 51.7, 26.0, 18.5, –5.2; IR (ATR): ṽ = 2953, 2930, 2886, 2856, 1720, 1649, 1619, 1435, 1380, 1324, 1256, 1226, 1177, 1124, 1061, 998, 962, 833, 775, 672; HRMS (ESI$^+$, *m*/*z*) calculated for $[C_{13}H_{24}O_3Si]^+$ ($[M^+]$) 256.1489, found 256.1485.

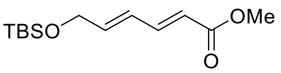

(**2*E*,4*E*)-Hexa-2,4-dienoyl fluoride** (**19**). **CAUTION**: Compounds which contain HF or can decompose to release HF should be handled carefully with appropriate gloves and other protective equipment to avoid any possibility of contact with skin. Acyl fluorides are more stable than acid chlorides, but may still hydrolyse under certain conditions to release HF.

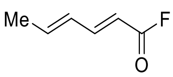

A solution of sorbic acid (2.24 g, 20.0 mmol) and pyridine (4.04 mL, 50.0 mmol) in dichloromethane (40 mL) was added to a stirred solution of HF-pyridine (0.60 mL, 4.76 mmol) and DCC (4.13 g, 20.0 mmol) in dichloromethane (10 mL) in a plastic container. The mixture was stirred at RT for 2 h, then filtered through silica (in a plastic funnel

blocked with cotton wool; washed with dichloromethane) and the combined filtrates were concentrated under reduced pressure (30 °C, 400 mbar). The residue was dissolved in *tert*-butyl methyl ether and transferred to a separating funnel, the solution was washed with cold HCl (1 M), cold aq. $NaHCO_3$ (saturated solution diluted to 5 x its original volume) and cold brine, dried over Na_2SO_4, filtered and concentrated under reduced pressure. Purification of the crude material by flash chromatography (SiO_2, pentane/diethyl ether 98:2) gave the product containing <10% sorbic acid as an impurity. Distillation under vacuum (~70 °C, 14 mbar) gave the title compound in analytically pure form as a colourless liquid (750 mg, 33% yield; Note: the yield was not optimised). ^{1}H NMR (400 MHz, $CDCl_3$) δ = 7.39 (dd, J = 15.5, 10.0 Hz, 1H), 6.37-6.21 (m, 2H), 5.70 (ddq, J = 15.5, 8.5, 0.5 Hz, 1H), 1.91 (d, J = 6.0 Hz, 3H); ^{13}C NMR (101 MHz, $CDCl_3$) δ = 157.5 (d, $^1J_{CF}$ = 336.8 Hz), 151.8 (d, $^3J_{CF}$ = 5.7 Hz), 144.1, 129.4, 112.9 ($^2J_{CF}$ = 66.8 Hz), 19.0; ^{19}F NMR (282 MHz, $CDCl_3$) δ = 24.3; IR (ATR): ṽ = 2954, 2932, 2856, 1816, 1251, 1103, 837.

(2*E*,4*E*)-1-Morpholinohexa-2,4-dien-1-one (21). $SOCl_2$ (0.87 mL, 12 mmol) was added

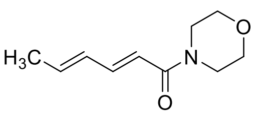

dropwise to a stirred solution of sorbic acid (1.1 g, 10 mmol) in dichloromethane (8.0 mL) at room temperature, followed by DMF (0.10 mL). The resulting suspension was stirred for 2 h before all volatile materials were evaporated. A solution of morpholine (1.4 g, 16 mmol) in THF (8 mL) and triethylamine (1.8 mL, 13 mmol) were added and the resulting mixture was stirred at room temperature for 2 h. The mixture was concentrated under reduced pressure to afford a yellow solid, which was purified by flash chromatography (SiO_2, ethyl acetate/hexane 1:1) to give the title compound as a white solid material (1.4 g, 77% yield). ^{1}H NMR (400 MHz, $CDCl_3$) δ 7.28 (dd, J = 14.7, 10.4 Hz, 1H), 6.26-6.04 (m, 3H), 3.72-3.56 (m, 8H), 1.84 (d, J = 6.1 Hz, 3H); ^{13}C NMR (101 MHz, $CDCl_3$) δ 166.1, 143.8, 138.2, 130.2, 117.3, 67.0, 67.0, 18.7; IR (ATR): ṽ = 3014, 2962, 2930, 2861, 1654, 1619, 1596, 1435, 1379, 1314, 1299, 1264, 1241, 1191, 1154, 1114, 1073, 1039, 990, 943, 926, 855, 793, 580; HRMS (ESI$^+$, *m*/*z*) calculated for $[C_{10}H_{15}NO_2]^+$ ($[M^+]$) 181.1097, found 181.1099. Matches known data.[14]

(2*E*,4*E*)-6,6,6-Trifluoro-1-morpholinohexa-2,4-dien-1-one (**23**).[15] A round bottom flask

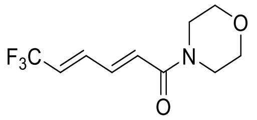

equipped with a reflux condenser was charged with 4-acryloylmorpholine (1.41 g, 1.26 mL, 10.0 mmol), α-trifluoromethylacrylic acid (350 mg, 2.50 mmol), (pentamethylcyclopentadienyl)rhodium(III) dichloride dimer (30.9 mg, 0.05 mmol), AgOAc (835 mg, 5.00 mmol) and DMF (12.5 mL). The resulting mixture was stirred at 120 °C under argon for 16 h. After cooling, the mixture was diluted with ethyl acetate (150 mL) and transferred to a separating funnel. The organic layer was washed with aq. HCl solution (1 M, 150 mL), water and brine, dried over Na_2SO_4, filtered and concentrated under reduced pressure. The residue was purified by flash chromatography (SiO_2, hexane/ethyl

acetate 1:1) to give the product with small isomeric impurities. Re-purification by flash chromatography (SiO_2, hexane/*tert*-butyl methyl ether 1:4) afforded the title compound in analytically pure form as a yellow-orange oil, which solidified upon standing in the fridge (6 °C) to an off-white solid (210 mg, 36% yield). ^{1}H NMR (400 MHz, C_6D_6) δ = 7.21-7.13 (m, 1H), 6-54-6.45 (m, 1H), 5.80 (dq, *J* = 15.0, 1.0 Hz, 1H), 5.35 (app. ddt, *J* = 15.5, 8.0, 6.5 Hz, 1H), 3.44 (br. s, 2H), 3.24 (br. s, 2H), 3.12 (br. s, 2H), 2.69 (br. s, 2H) (4 broad signals for morpholine protons due to restricted rotation about amide C–N bond); ^{13}C NMR (101 MHz, C_6D_6) δ = 163.4, 137.9, 135.6 (q, *J* = 6.9 Hz), 127.4, 123.8 (q, *J* = 33.7 Hz), 123.6 (q, *J* = 270.1 Hz), 66.6, 45.8, 42.5 (2 carbons attached to nitrogen appear in different environments due to restricted rotation about amide C–N bond); ^{19}F NMR (282 MHz, C_6D_6) δ = –63.9; IR (ATR): ṽ = 3039, 2982, 2930, 2862, 1633, 1608, 1442, 1269, 1240, 1109, 1090, 995, 851, 680, 561, 454; HRMS (EI, *m*/*z*) calculated for $[C_{10}H_{12}NO_2F_3]^+$ ($[M^+]$) 235.0815, found 235.0814.

4,4,5,5-Tetramethyl-2-((1*E*,3*E*)-penta-1,3-dien-1-yl)-1,3,2-dioxaborolane (25). *n*-BuLi (1.6 M in hexane, 9.8 mL, 16 mmol) was added to a stirred solution of 2,2,6,6-tetramethylpiperidine (2.6 mL, 16 mmol) in THF (16 mL) at −78 °C. After stirring for 10 min, bis[(pinacolato)boryl]methane (4.2 g, 16 mmol) was added at 0 °C and the resulting mixture was stirred for 10 min. (*E*)-Crotonaldehyde (1.0 mL, 12 mmol) was introduced and stirring continued at room temperature for 1 h. The mixture was diluted with diethyl ether (10 mL) and the reaction quenched by slow addition of water (10 mL). The aqueous phase was extracted by diethyl ether (2 × 10 mL). The combined organic layers were washed with brine (10 mL), dried over Na_2SO_4, filtered and concentrated under reduced pressure to afford a yellow oil. The residue was purified by flash chromatography (SiO_2, hexane 100%) to give the title compound as a colorless oil (0.74 g, 32% yield). ^{1}H NMR (400 MHz, $CDCl_3$) δ 6.97 (dd, *J* = 17.7, 10.4 Hz, 1H), 6.21-6.08 (m, 1H), 5.96-5.84 (m, 1H), 5.40 (dq, *J* = 17.6, 0.7 Hz, 1H), 1.78 (dd, *J* = 6.8, 1.6 Hz, 3H), 1.26 (s, 12H); ^{13}C NMR (101 MHz, $CDCl_3$) δ 150.4, 134.3, 134.0, 83.2, 24.9, 18.4. Matches known data.[16]

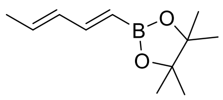

Assignment of the Absolute and Relative Configuration

The absolute and relative configuration of product **15b** was determined by X-ray crystallography after conversion into the corresponding osmate ester (see Figures S1-S3).[17]

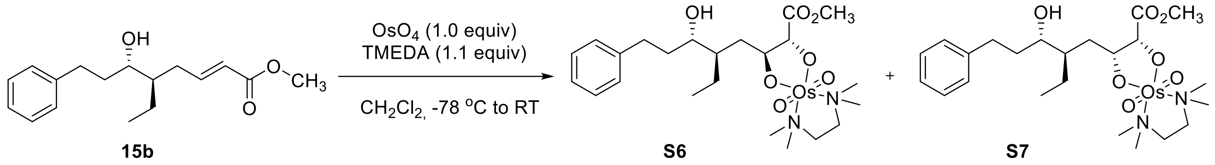

N,*N*,*N′*,*N′*-Tetramethylethylenediamine (TMEDA) (10 µL, 0.68 mmol) was added to a stirred solution of compound **15b** (17 mg, 0.62 mmol) in dichloromethane (1.0 mL). The resulting solution was cooled to –78 °C before a solution of osmium tetroxide (0.1 M in dichloromethane, 0.62 mL, 0.62 mmol) was added. The mixture was stirred for 30 min at –78 °C before it was allowed to reach room temperature. The solvent was evaporated to afford a brown solid residue, which was purified by flash chromatography (SiO_2, ethanol/dichloromethane 1:10) to give the desired osmate ester as a brown oil (39 mg, 98% yield, dr ≈ 1.5:1). The mixture of diastereomers was separated by preparative TLC (acetone/hexane 2:1) to provide **S6** (17 mg) and **S7** (10 mg) as a brown solid each. Single crystals suitable for X-ray diffraction analysis were obtained by diffusing pentane into a saturated solution of complex **S6** in dichloromethane at room temperature. $[\alpha]_D^{20}$ = –637.1 (c = 0.51, $CHCl_3$); ^1H NMR (400 MHz, $CDCl_3$) δ 7.25-7.22 (m, 2H), 7.21-7.17 (m, 2H), 7.17-7.12 (m, 1H), 4.48 (d, *J* = 8.8 Hz, 1H), 4.39 (td, *J* = 9.3, 1.7 Hz, 1H), 3.77 (s, 3H), 3.51 (tt, *J* = 8.6, 4.2 Hz, 1H), 3.13-3.05 (m, 4H), 2.93 (s, 3H), 2.91 (s, 3H), 2.87 (s, 3H), 2.86 (s, 3H), 2.86-2.80 (m, 1H), 2.65 (ddd, *J* = 13.7, 10.1, 6.5 Hz, 1H), 2.18 (ddd, *J* = 15.2, 5.3, 1.8 Hz, 1H), 2.12-2.05 (m, 1H), 1.93 (dddd, *J* = 13.9, 10.2, 8.9, 5.1 Hz, 1H), 1.86-1.71 (m, 2H), 1.69-1.63 (m, 1H), 1.60-1.46 (m, 2H), 0.89 (t, *J* = 7.4 Hz, 3H); ^{13}C NMR (101 MHz, $CDCl_3$) δ 172.0, 143.2, 128.6, 128.3, 125.6, 92.9, 86.7, 77.4, 72.6, 65.0, 64.5, 52.3, 52.3, 52.2, 52.0, 43.8, 38.0, 33.1, 29.7, 23.3, 12.2; IR (ATR): ṽ = 3245, 3022, 2931, 2873, 1736, 1474, 1456, 1280, 1214, 1173, 1040, 1013, 958, 843, 800, 749, 668, 615, 496; HRMS (ESI^+, *m/z*) calculated for $[C_{23}H_{40}N_2O_7Os+Na]^+$ ($[M+Na]^+$) 671.2342, found 671.2337.

All other compounds were assigned by analogy; in two cases, this tentative assignment was independently confirmed by comparison with data of literature-known compounds:

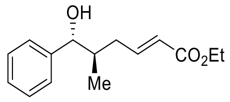

6b (>20:1 dr, 97% ee): $[\alpha]_D^{25}$ = +14.8 (c = 1.2, $CHCl_3$) (ref.[9]: $[\alpha]_D^{25}$ of the antipode: –11.8); the recorded spectral data match the literature.

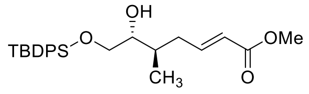

17 (>20:1 dr, 96% ee): $[\alpha]_D^{20}$ = –2.2 (c = 0.50, $CHCl_3$) (lit.[10]: $[\alpha]_D^{19}$ = –3.8 (c = 0.48, $CHCl_3$); the recorded spectral data match the literature.

NMR Spectra

^{1}H NMR (400 MHz, $CDCl_3$; top) and ^{13}C NMR (101 MHz, $CDCl_3$; bottom) of compound **6a**

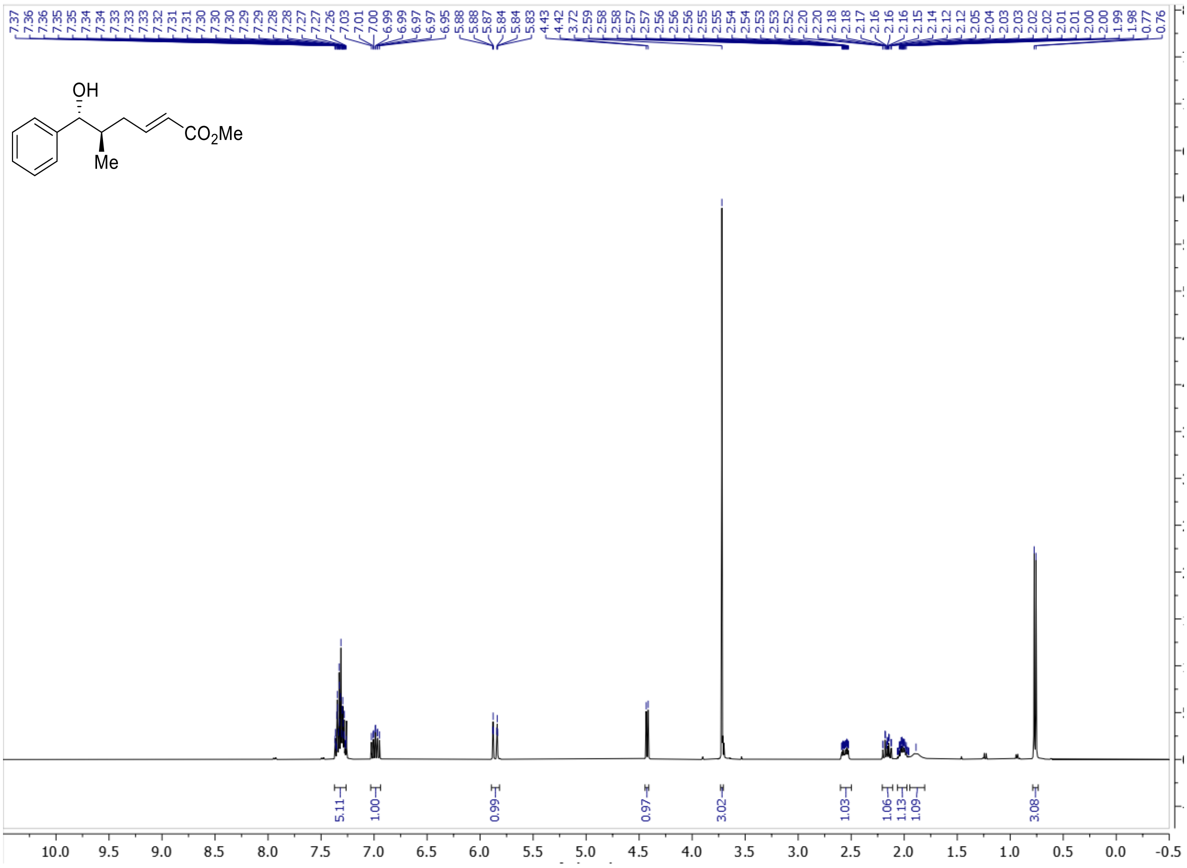

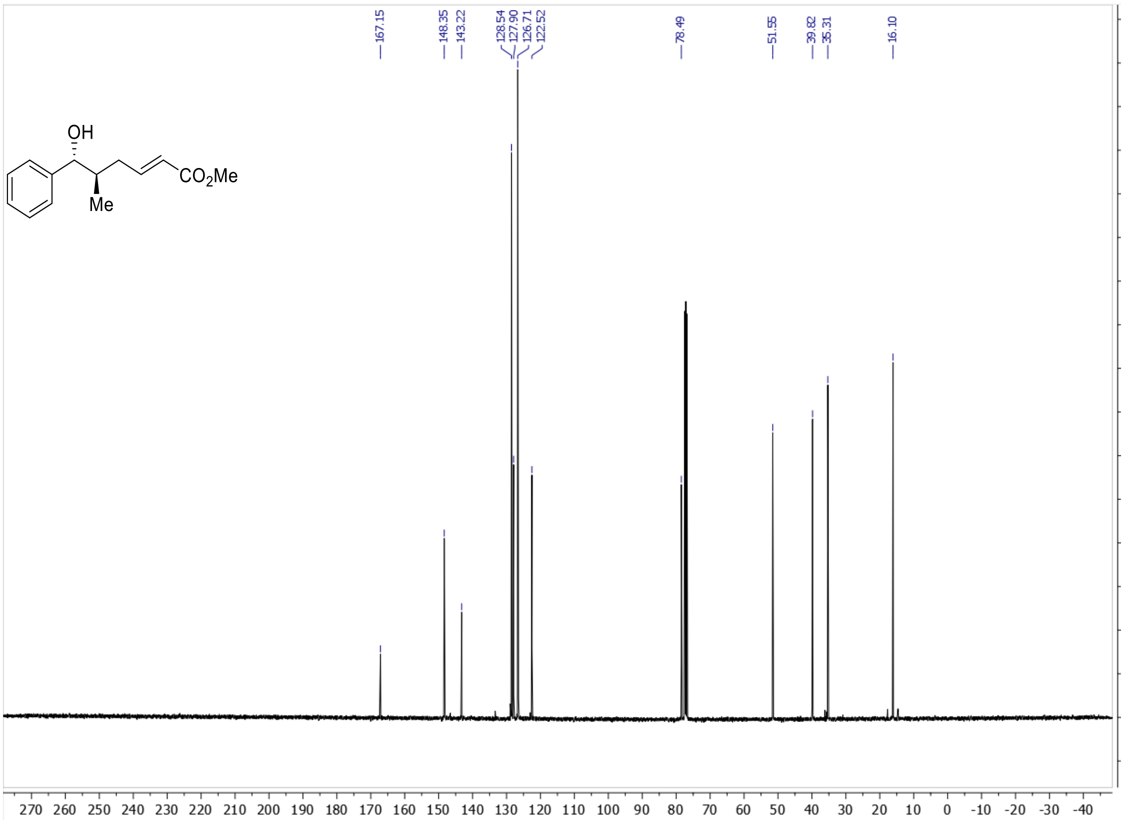

^{1}H NMR (400 MHz, $CDCl_3$; top) and ^{13}C NMR (101 MHz, $CDCl_3$; bottom) of compound **6b**

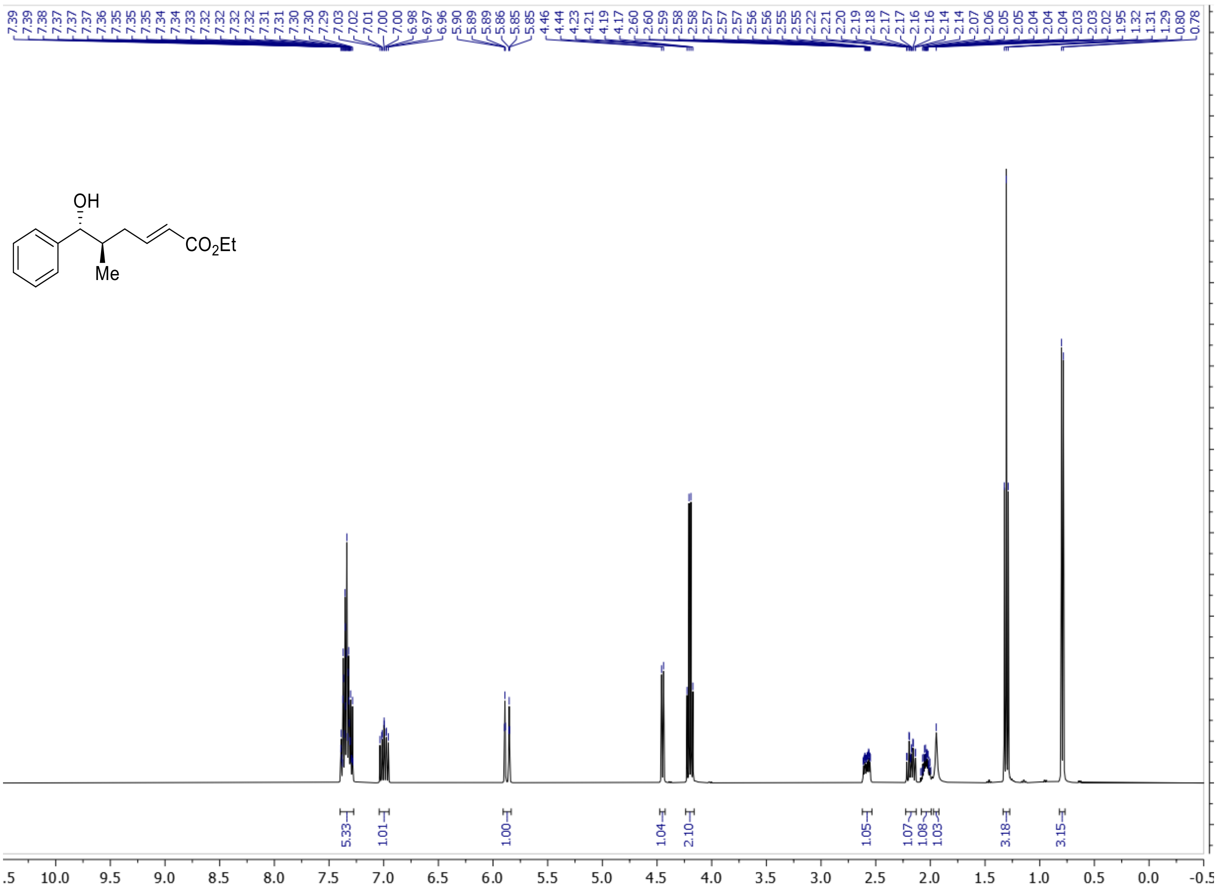

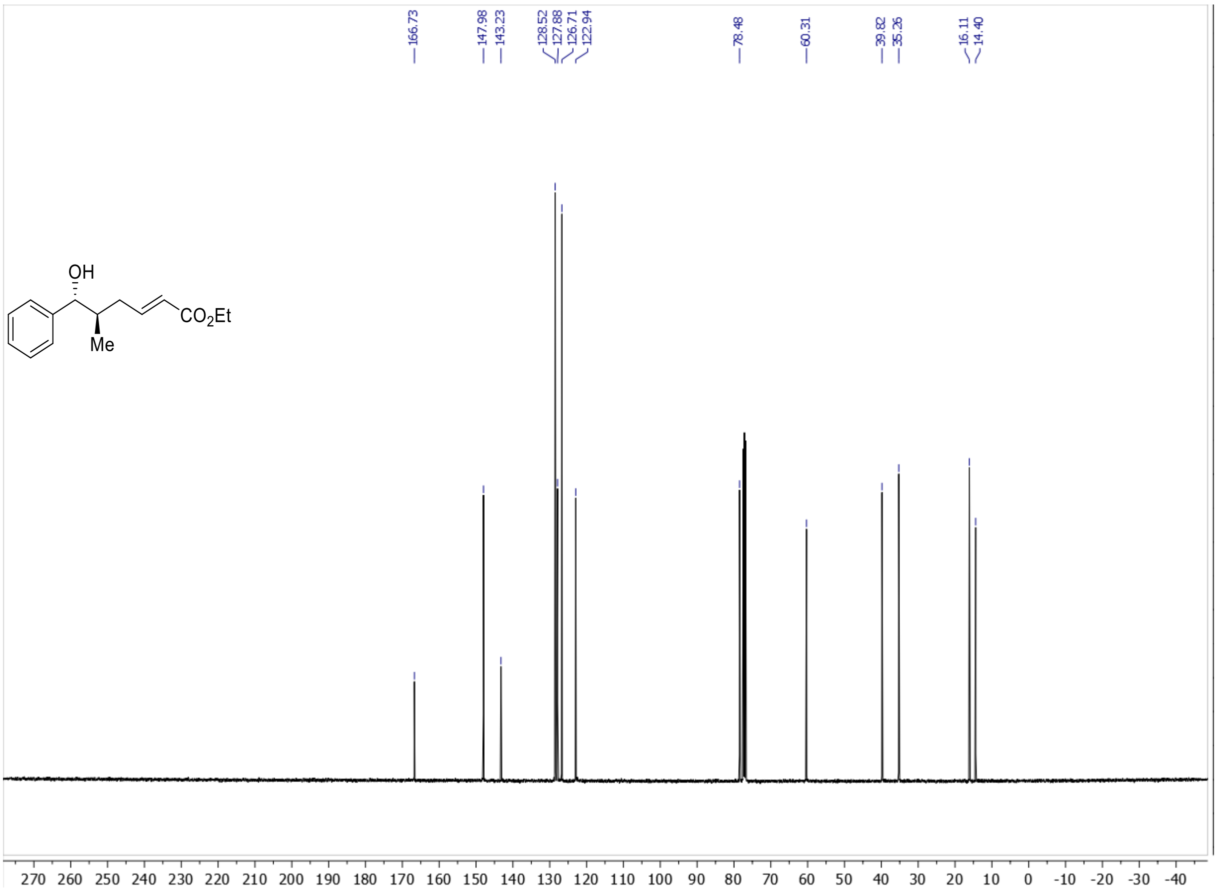

^{1}H NMR (400 MHz, $CDCl_3$; top) and ^{13}C NMR (101 MHz, $CDCl_3$; bottom) of compound **7a**

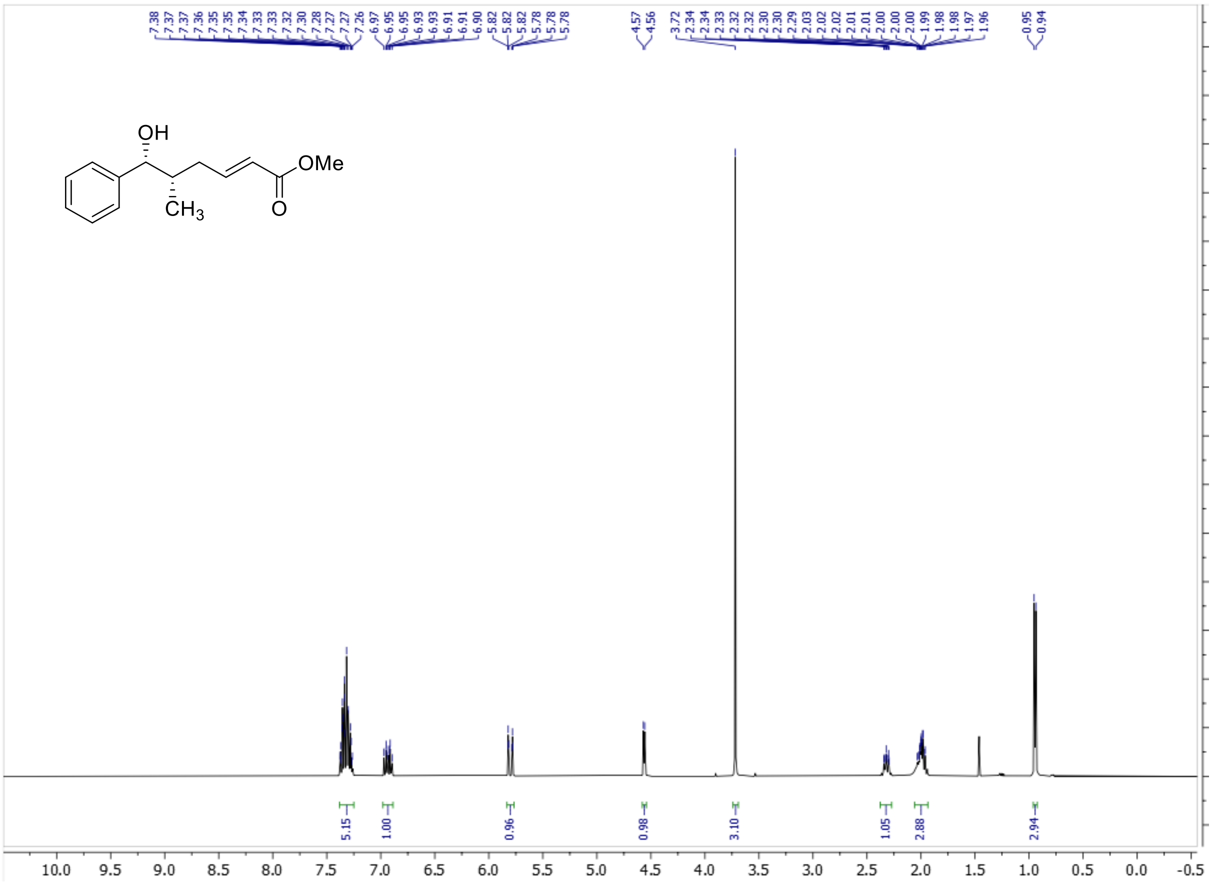

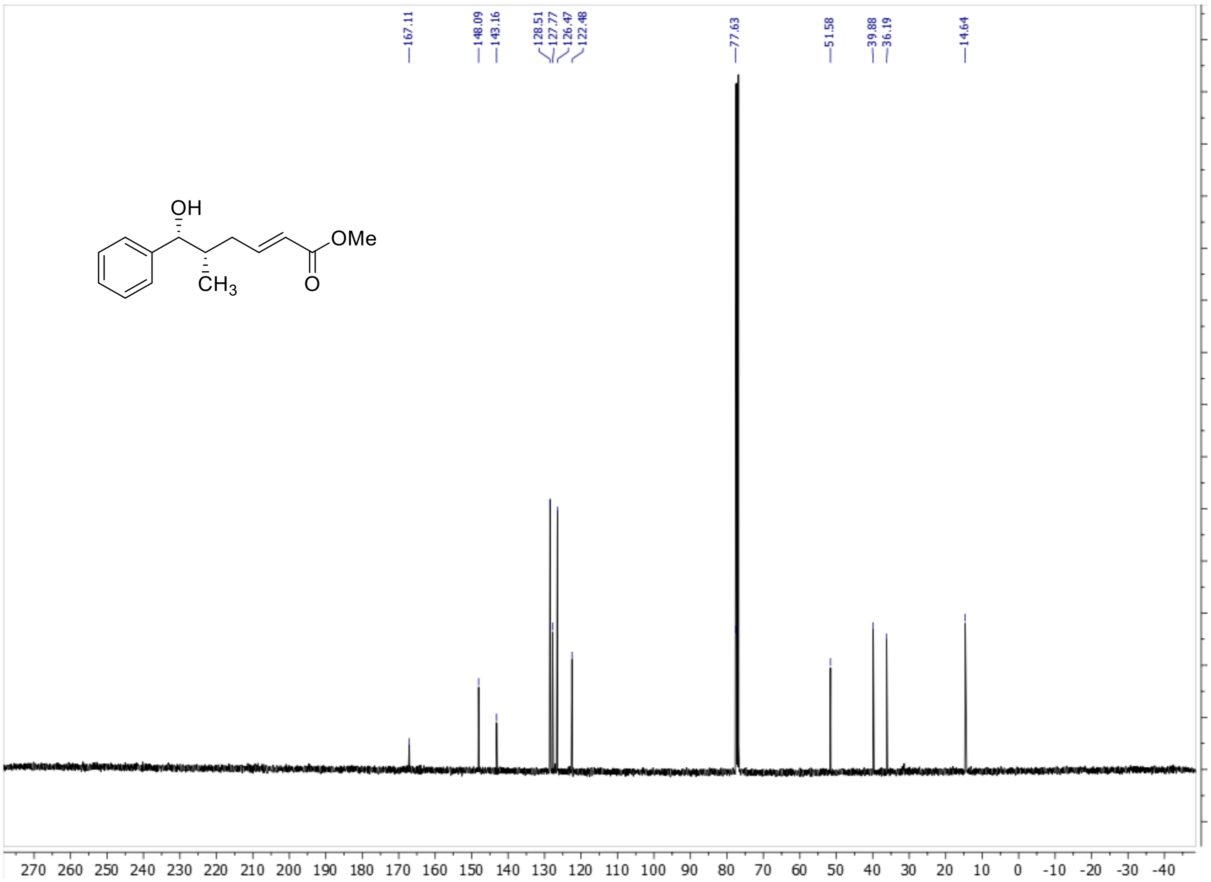

^{1}H NMR (400 MHz, $CDCl_3$; top) and ^{13}C NMR (101 MHz, $CDCl_3$; bottom) of compound **7b**

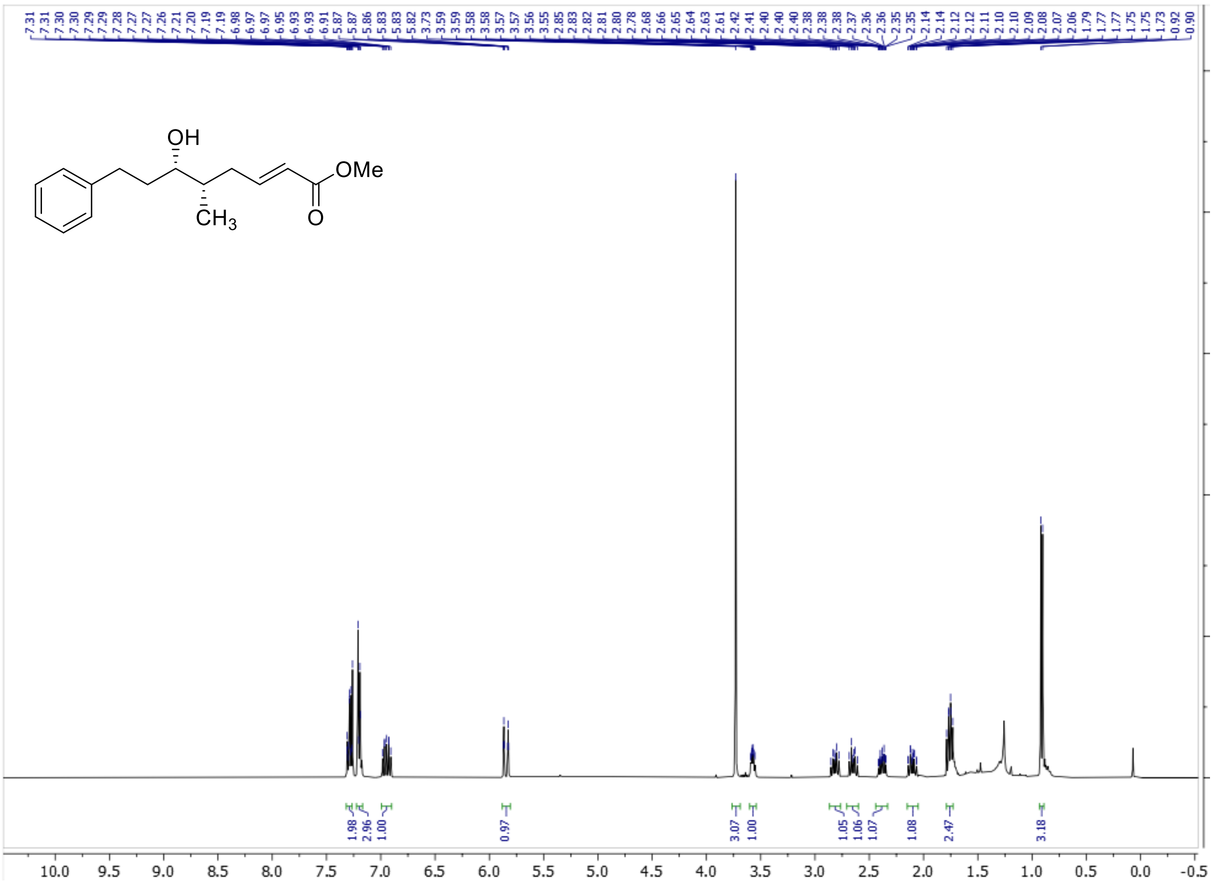

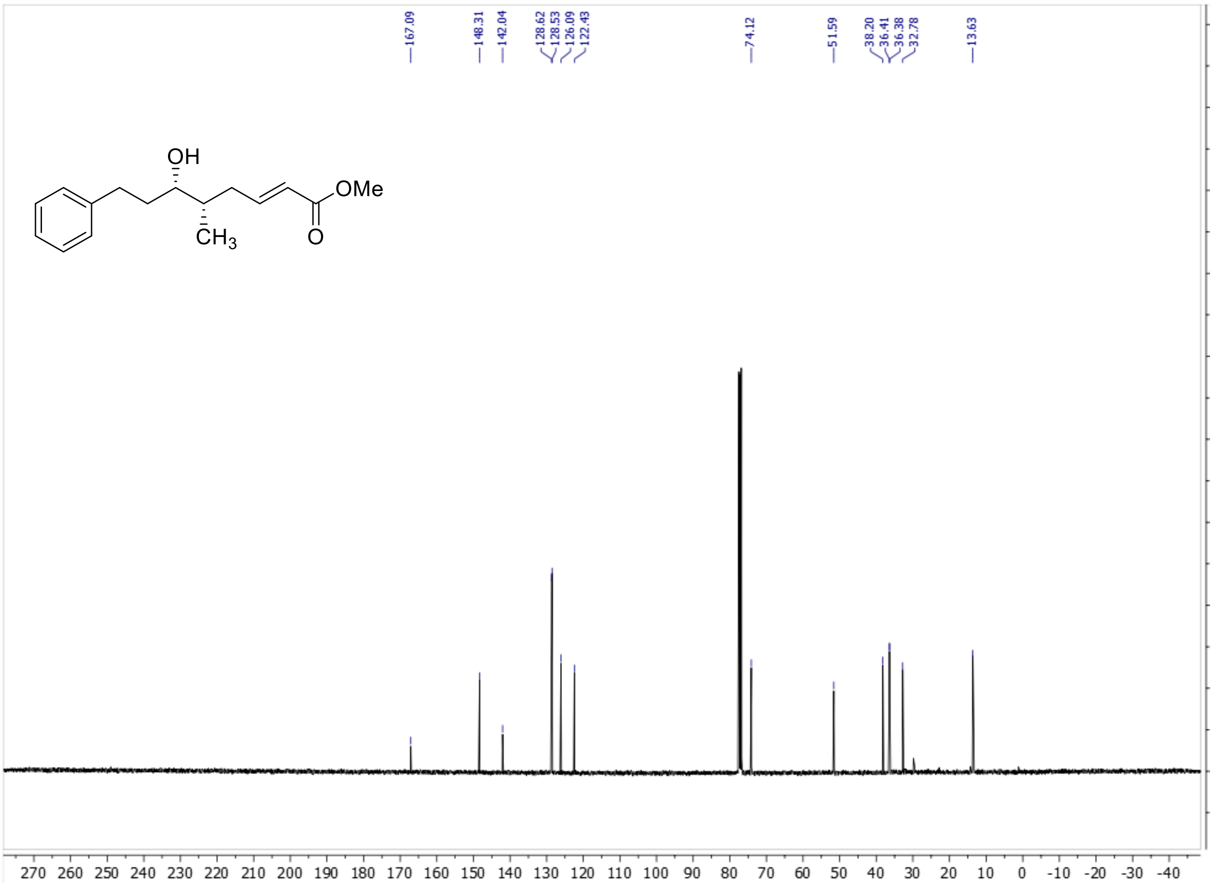

^{1}H NMR (400 MHz, $CDCl_3$; top) and ^{13}C NMR (101 MHz, $CDCl_3$; bottom) of compound **8a**

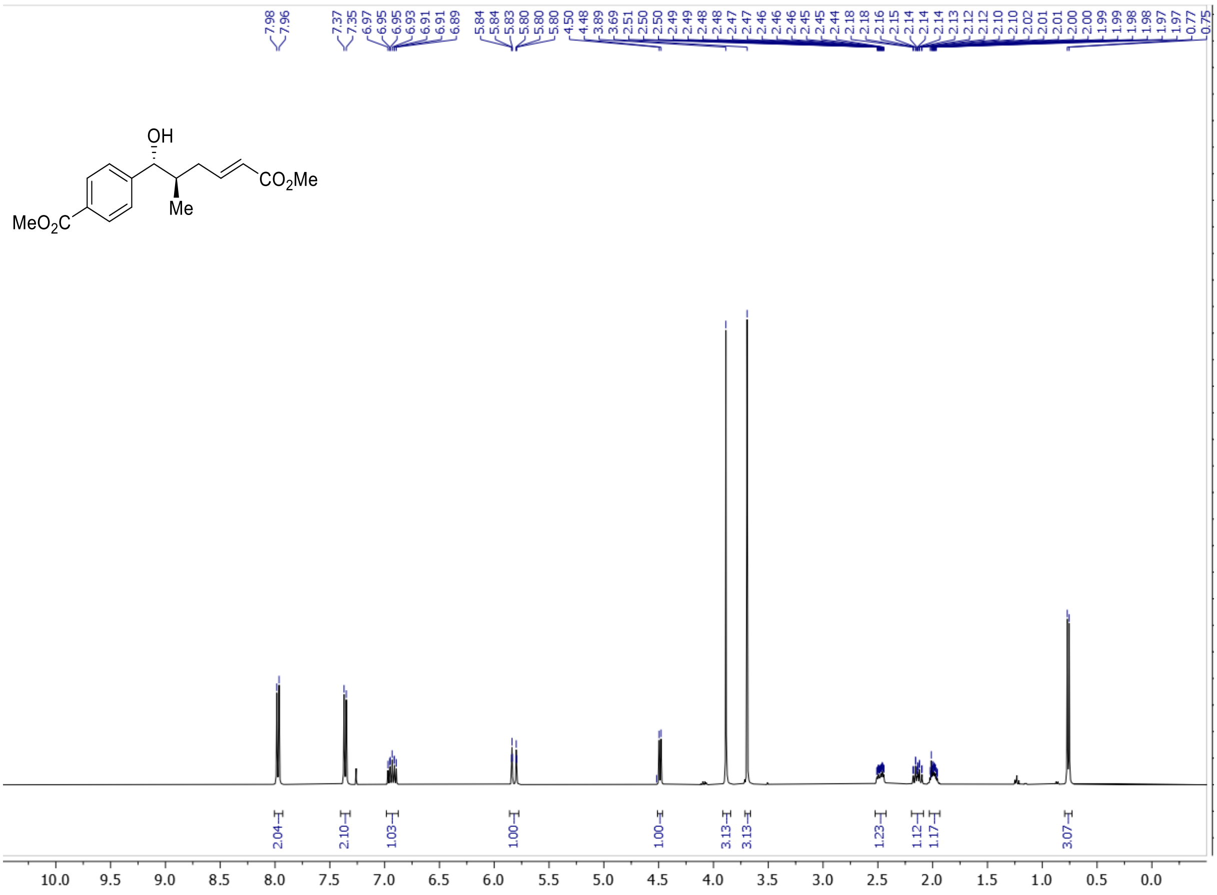

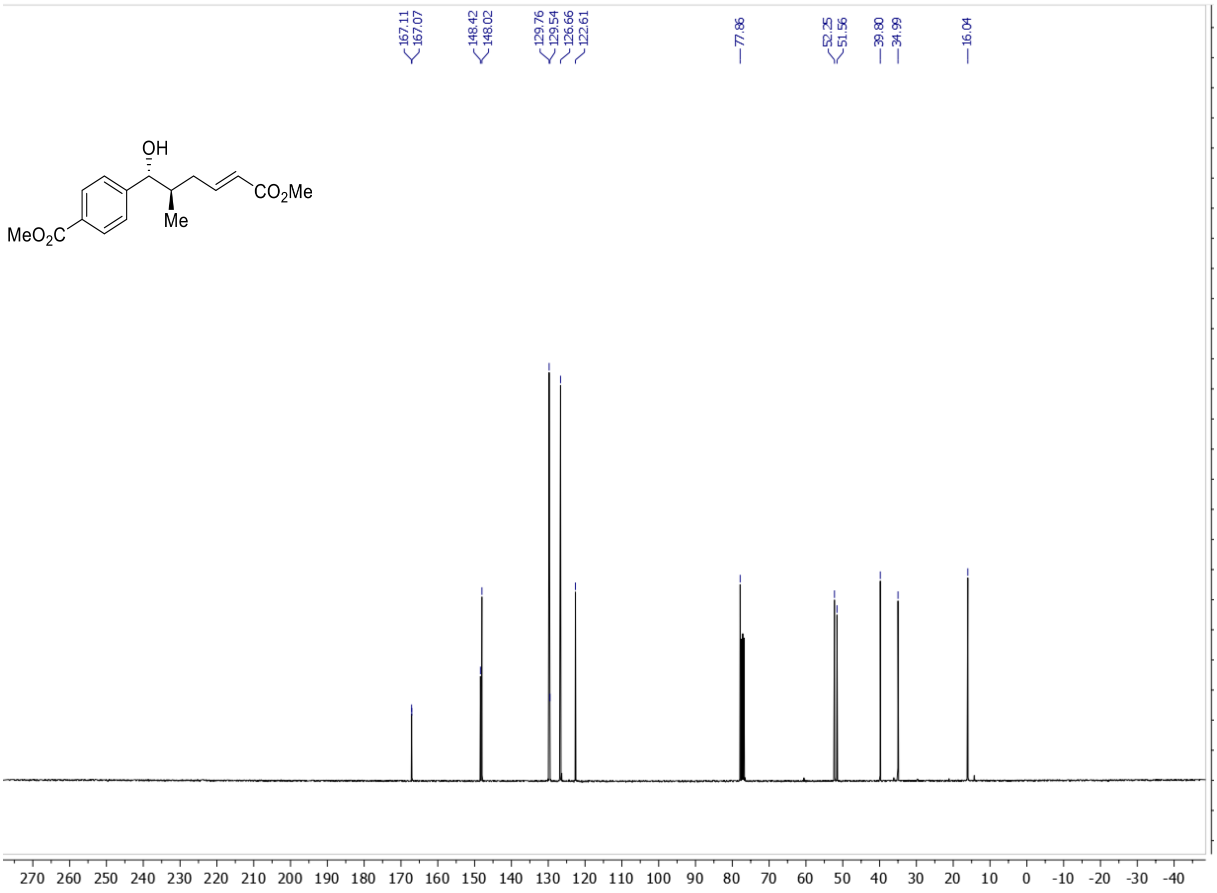

^{1}H NMR (400 MHz, $CDCl_3$; top) and ^{13}C NMR (101 MHz, $CDCl_3$; bottom) of compound **8b**

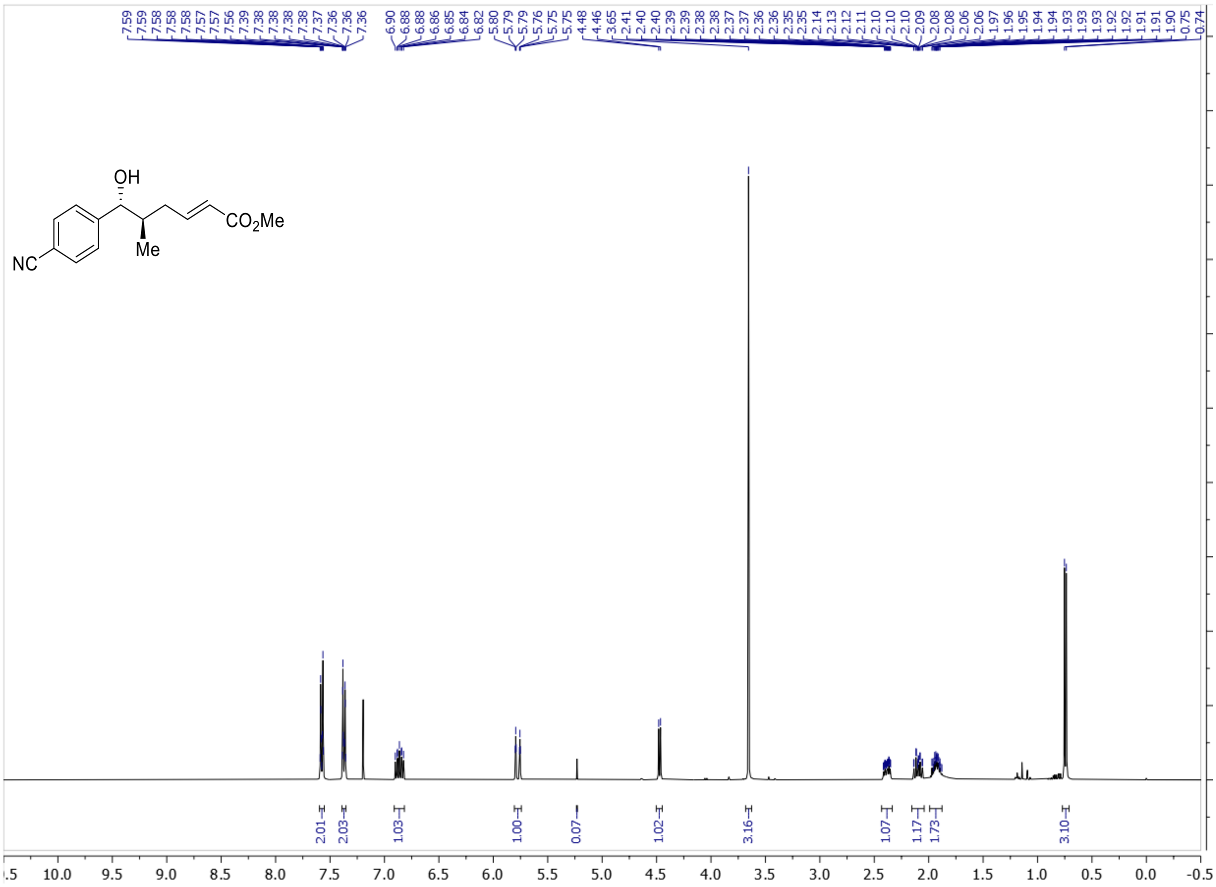

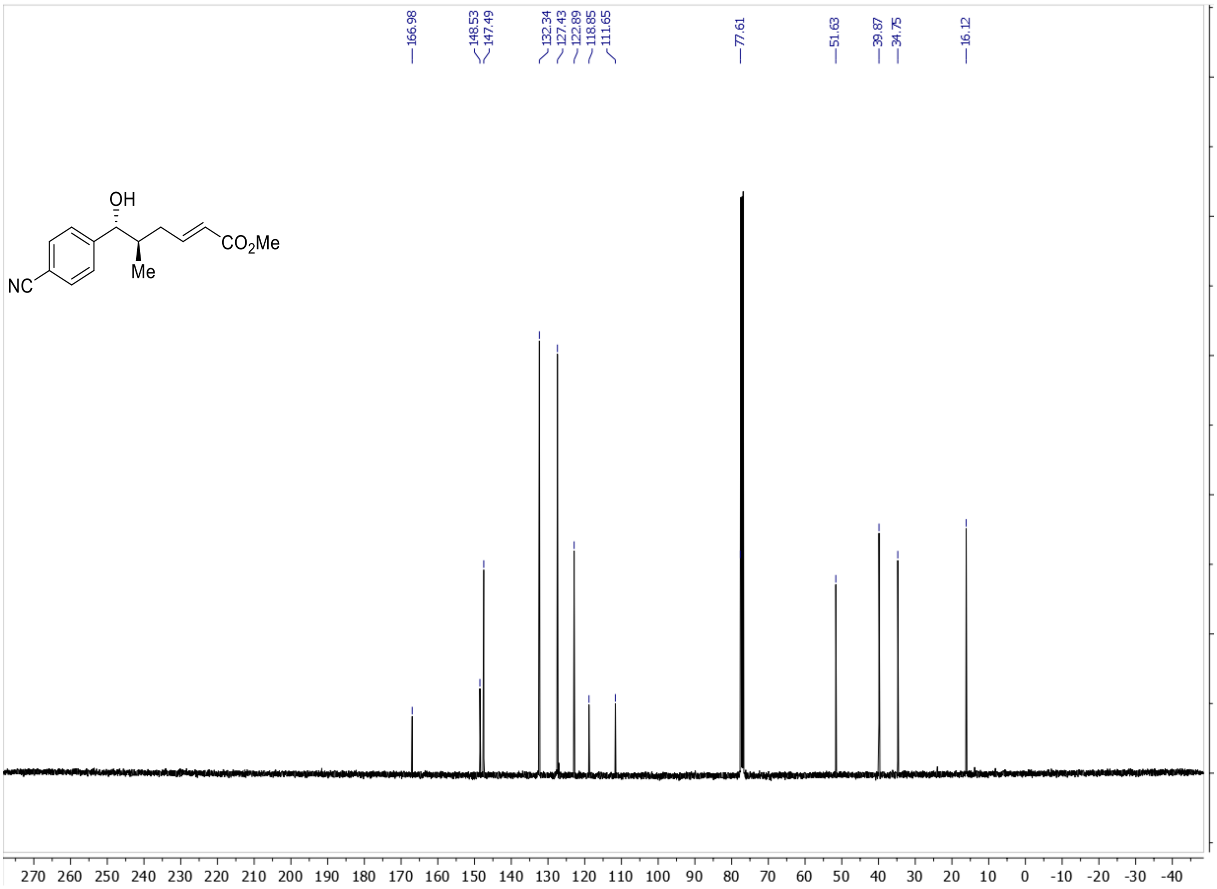

^1H NMR (400 MHz, $CDCl_3$; top) and ^{13}C NMR (101 MHz, $CDCl_3$; bottom) of compound **8c**

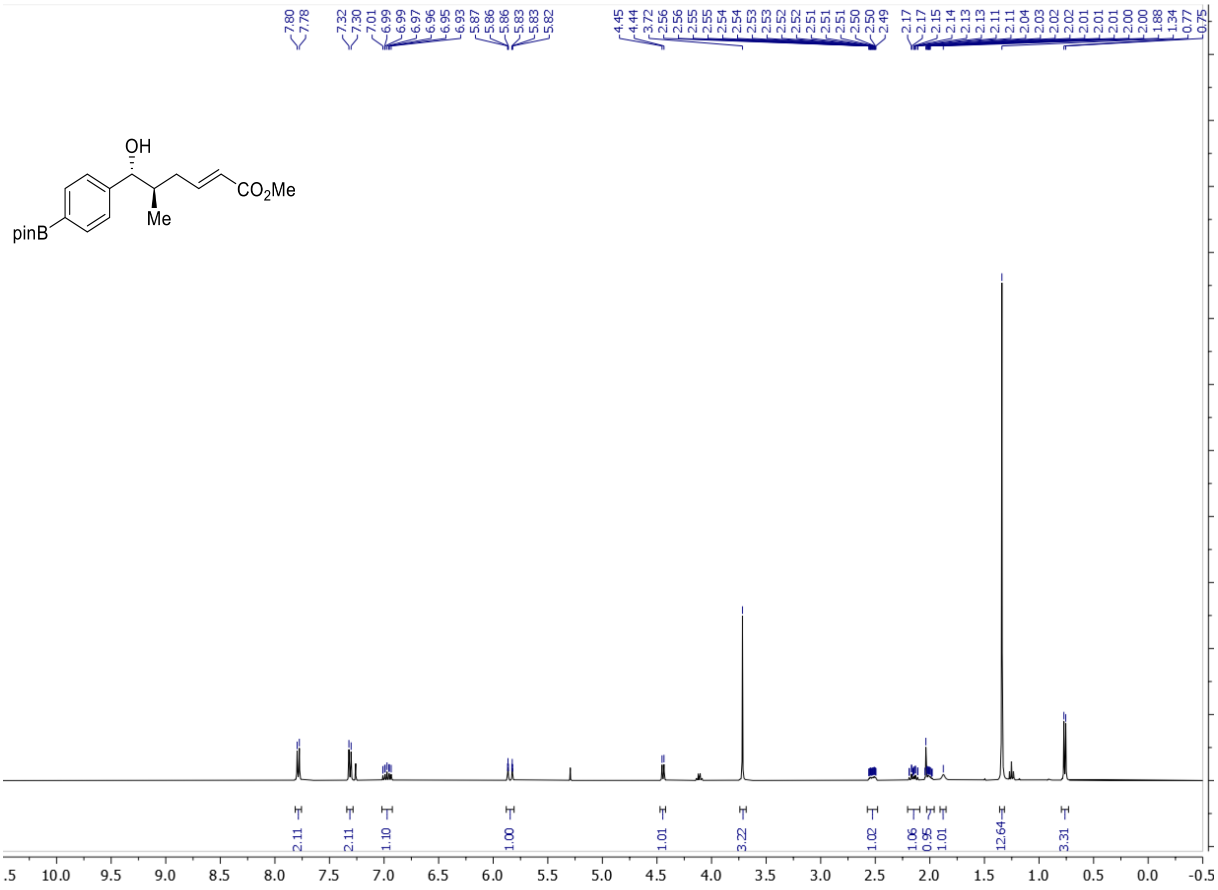

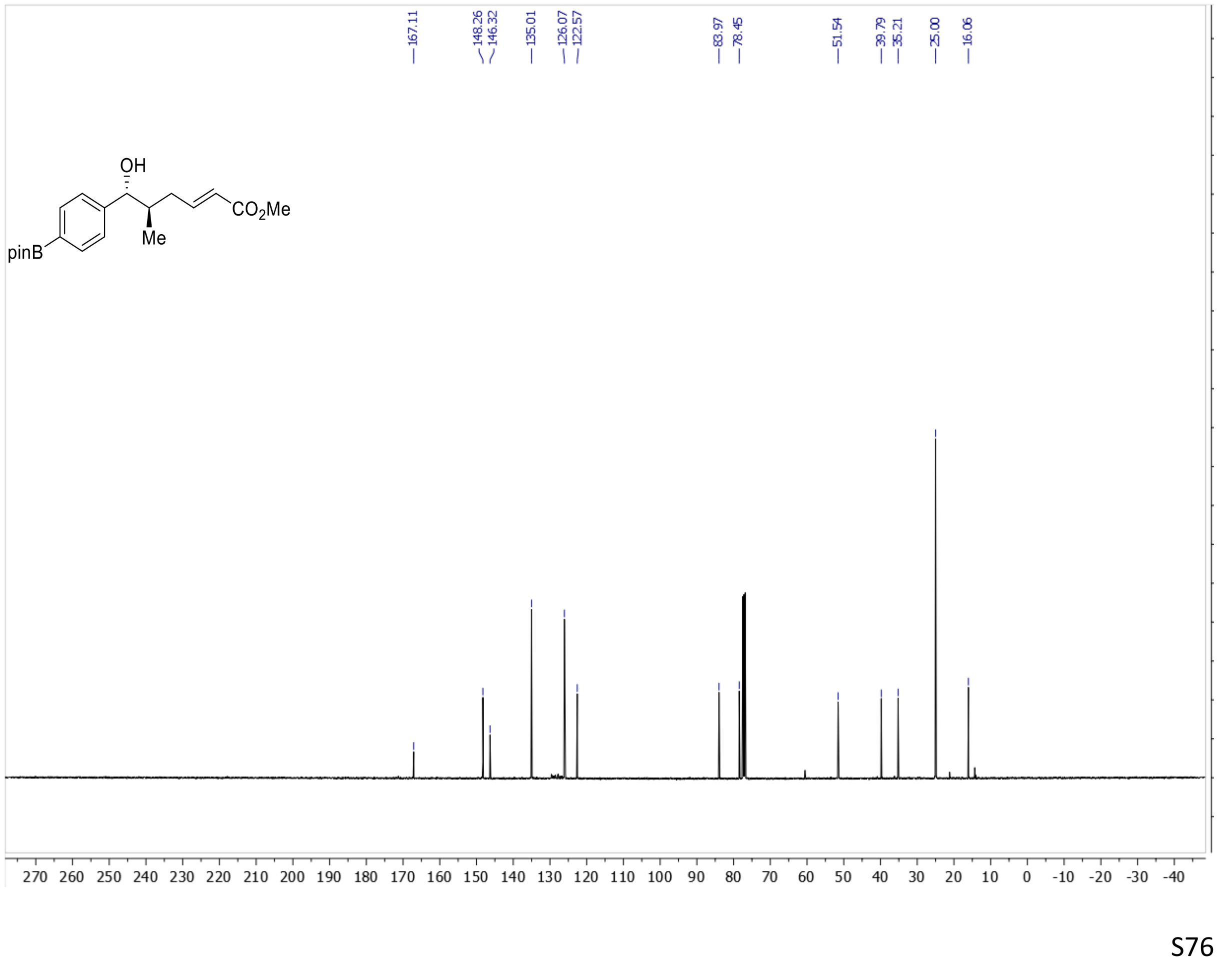

^{1}H NMR (300 MHz, $CDCl_3$; top) and ^{13}C NMR (75 MHz, $CDCl_3$; bottom) of compound **8d**

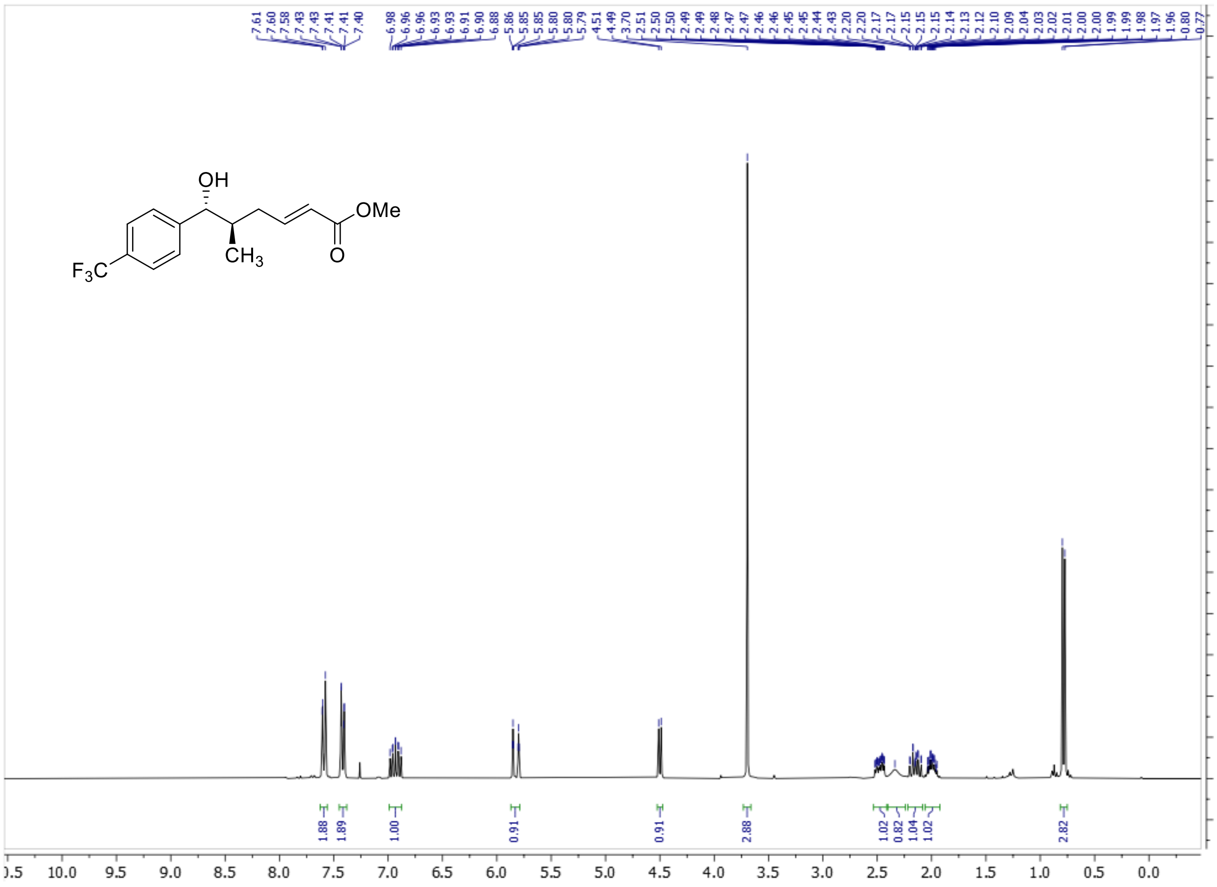

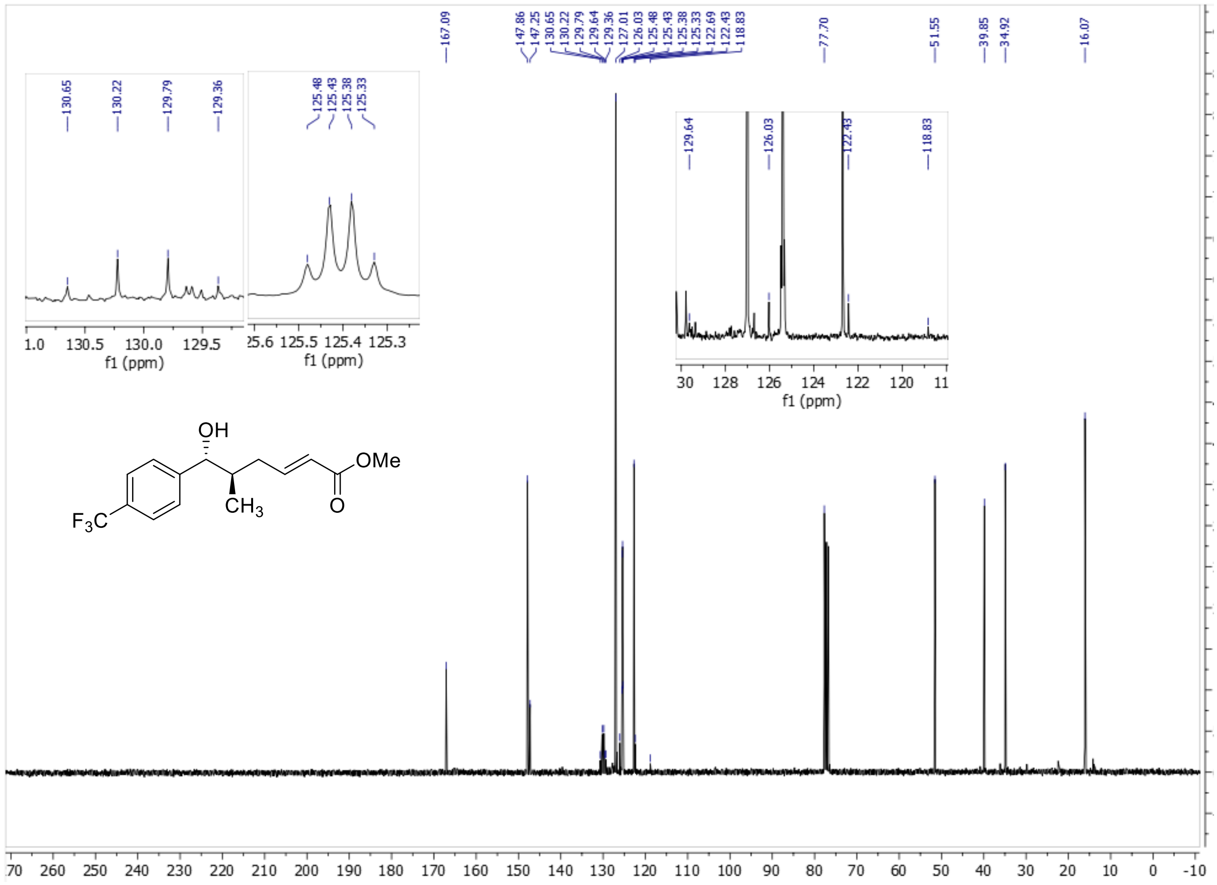

^{19}F NMR (282 MHz, $CDCl_3$) of compound **8d**

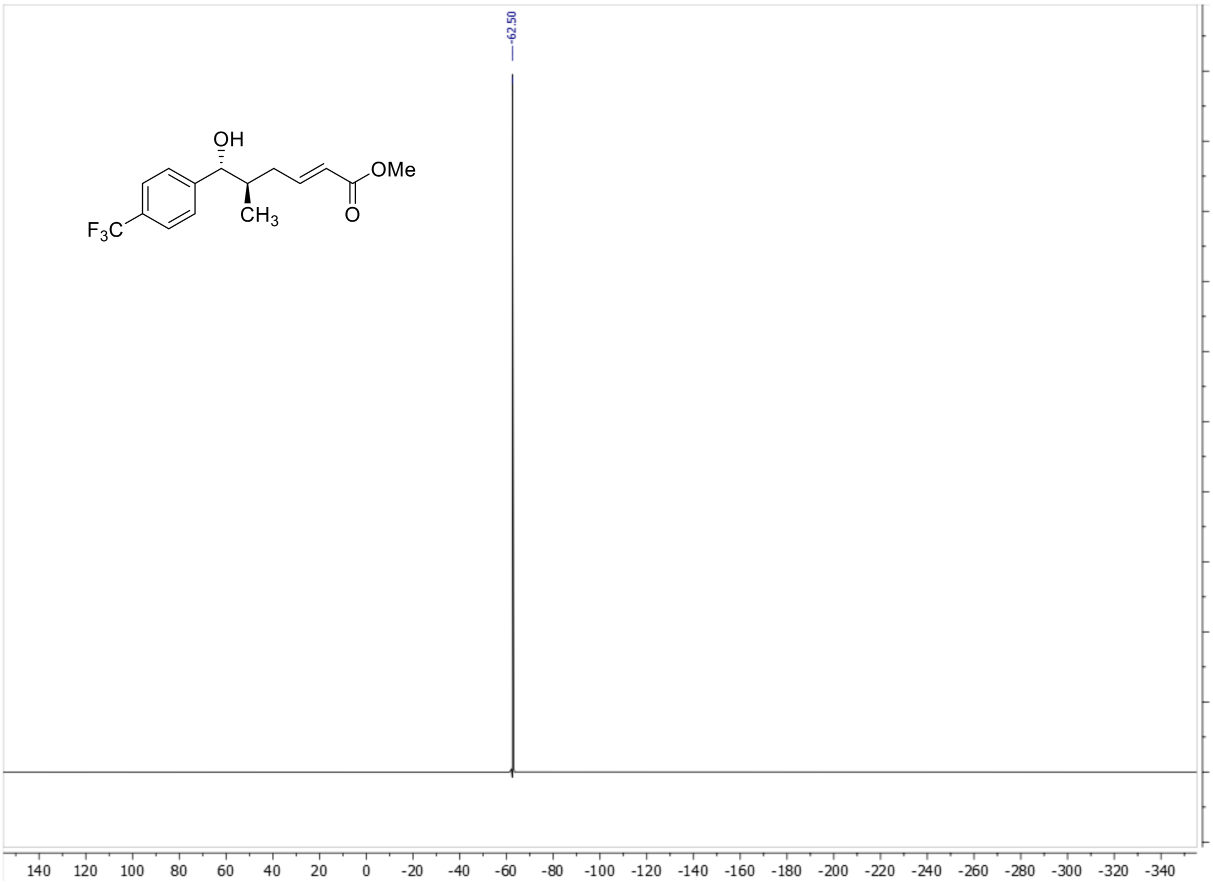

^{1}H NMR (400 MHz, $CDCl_3$; top) and ^{13}C NMR (101 MHz, $CDCl_3$; bottom) of compound **8e**

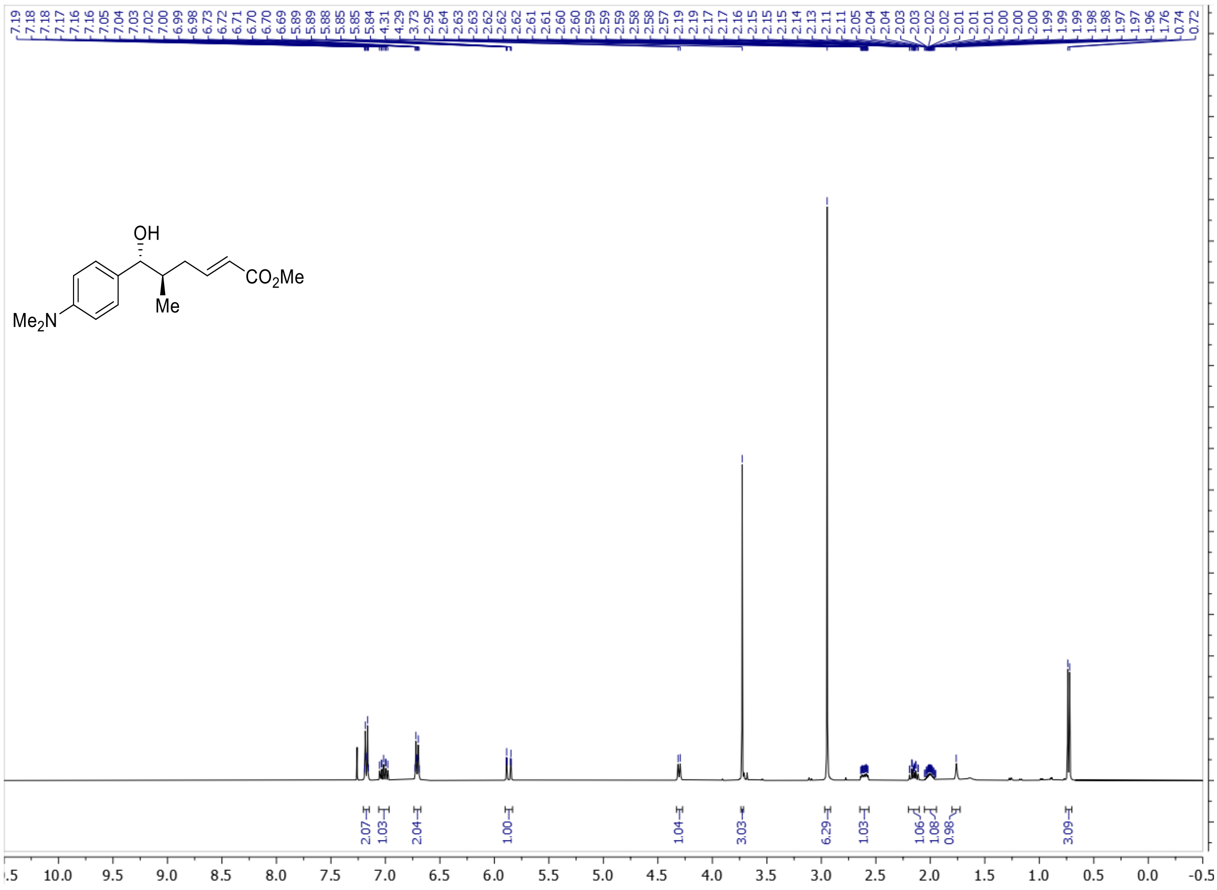

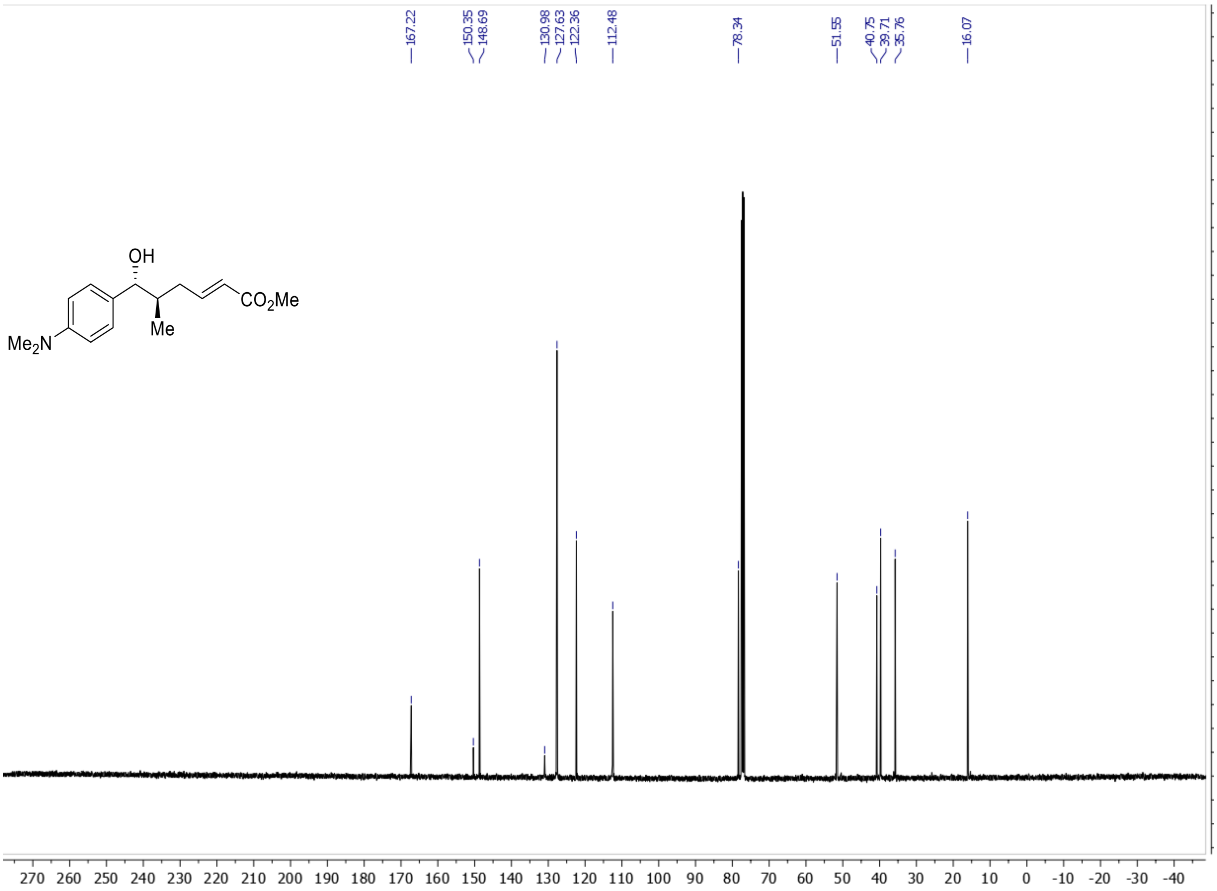

^{1}H NMR (400 MHz, $CDCl_3$; top) and ^{13}C NMR (101 MHz, $CDCl_3$; bottom) of compound **9**

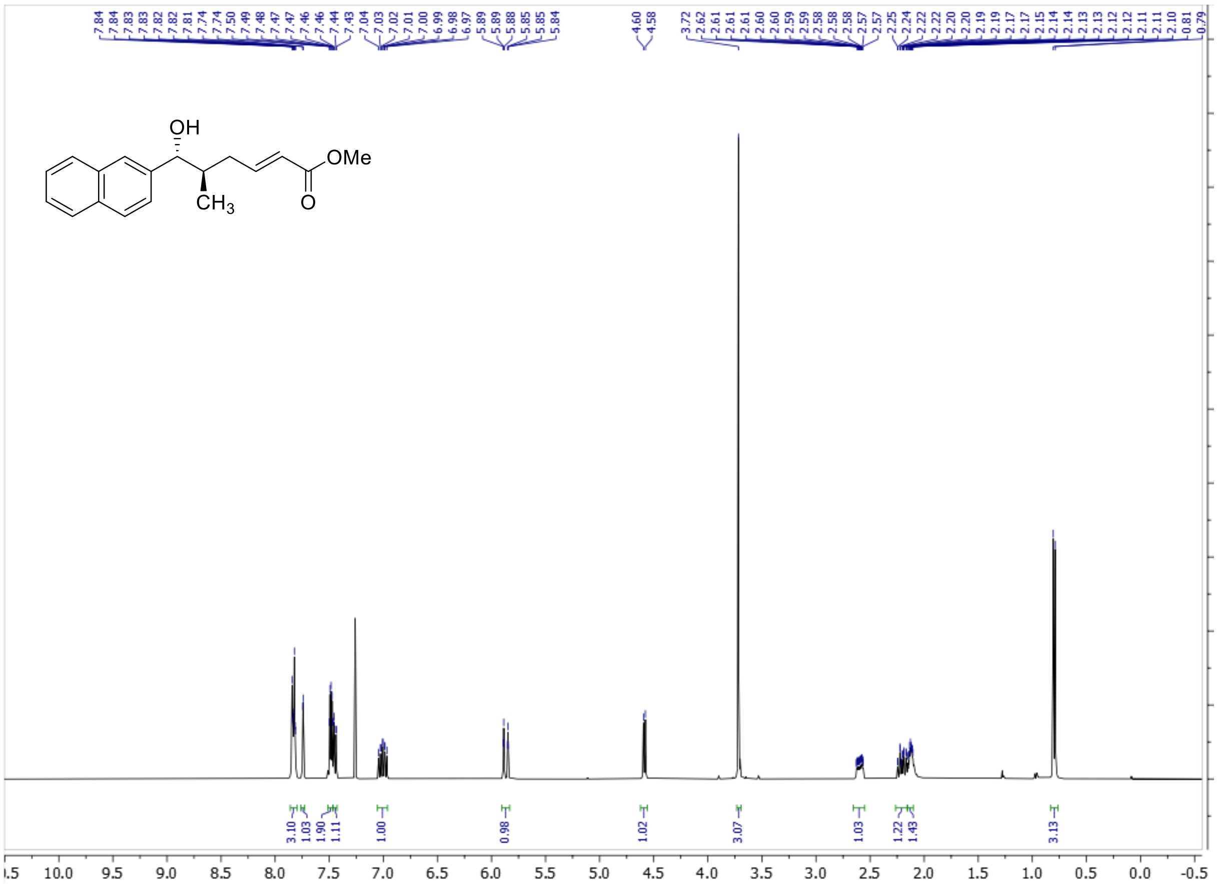

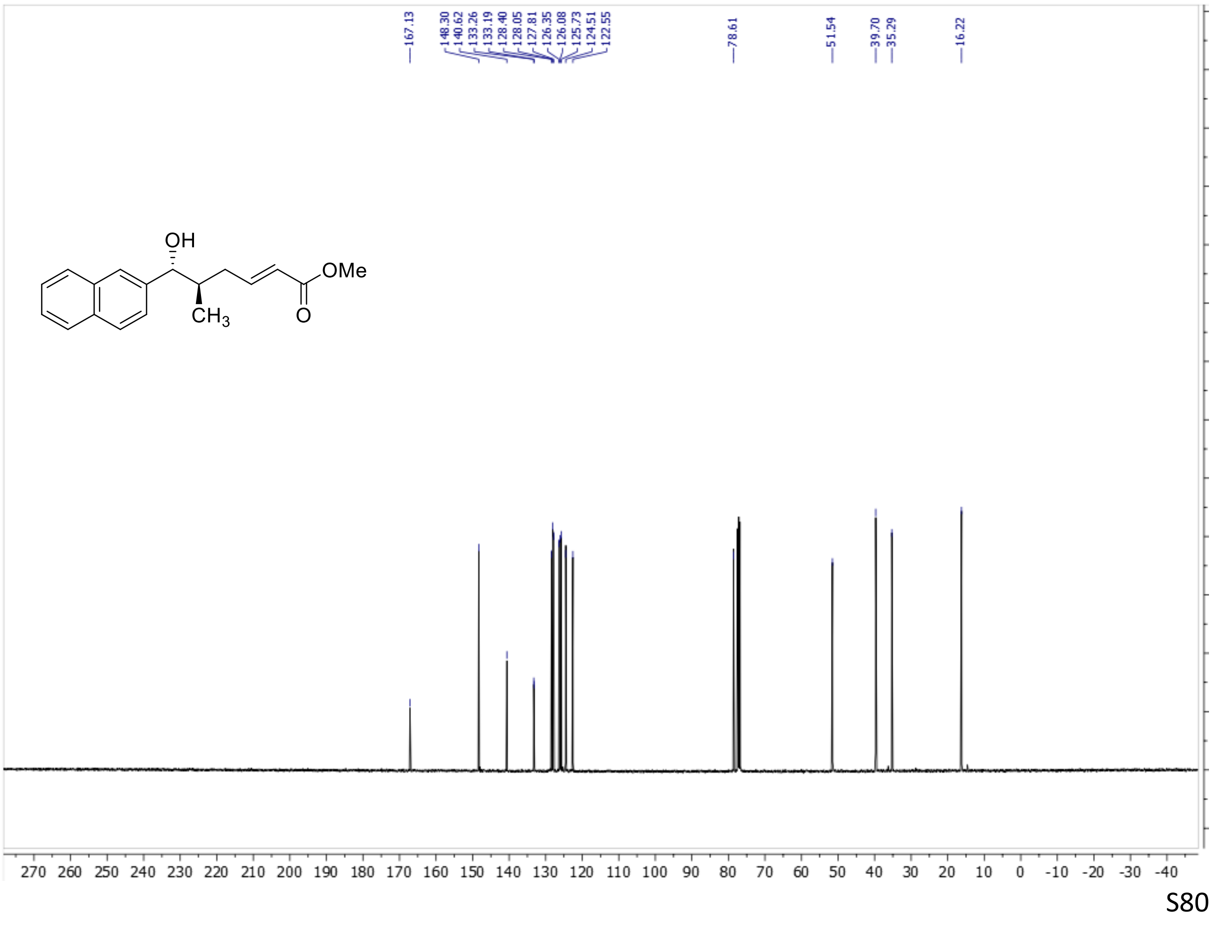

^{1}H NMR (400 MHz, $CDCl_3$; top) and ^{13}C NMR (101 MHz, $CDCl_3$; bottom) of compound **10**

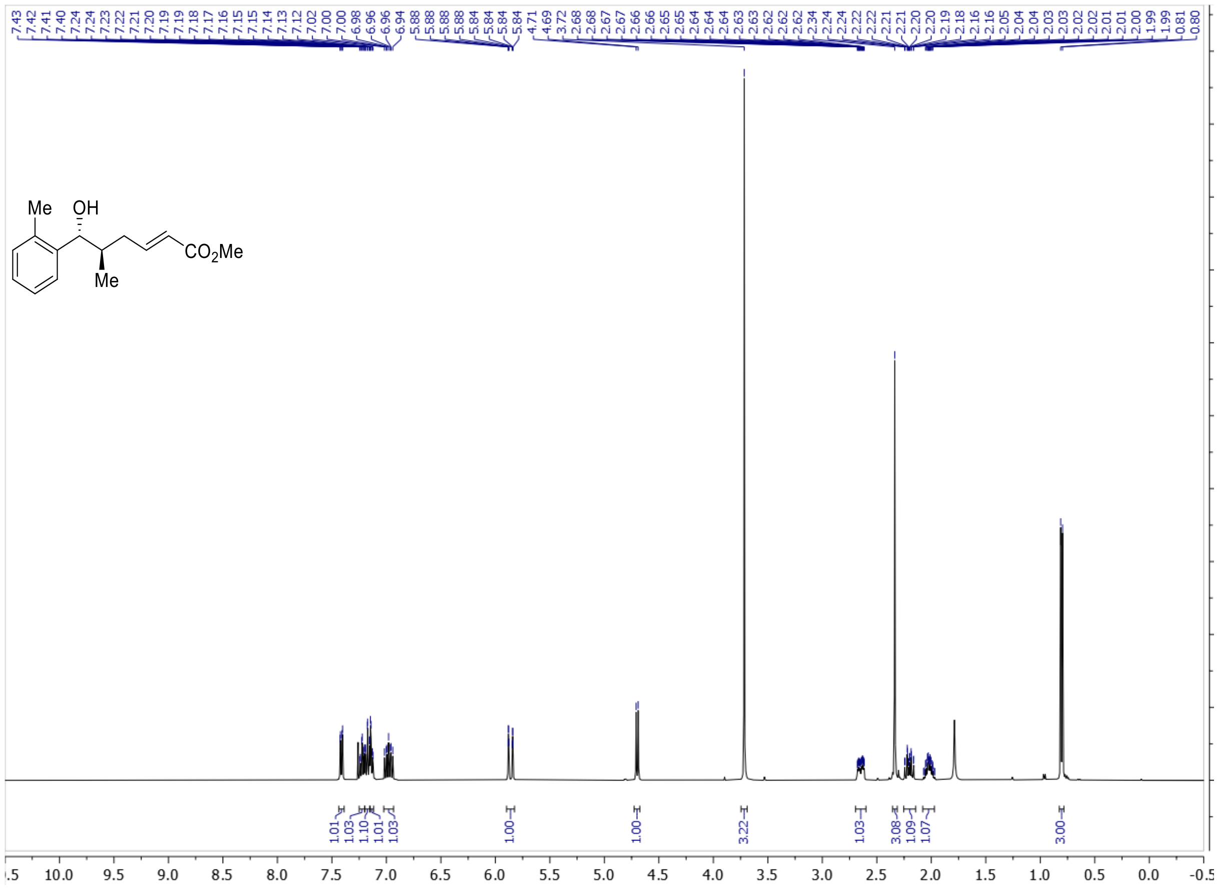

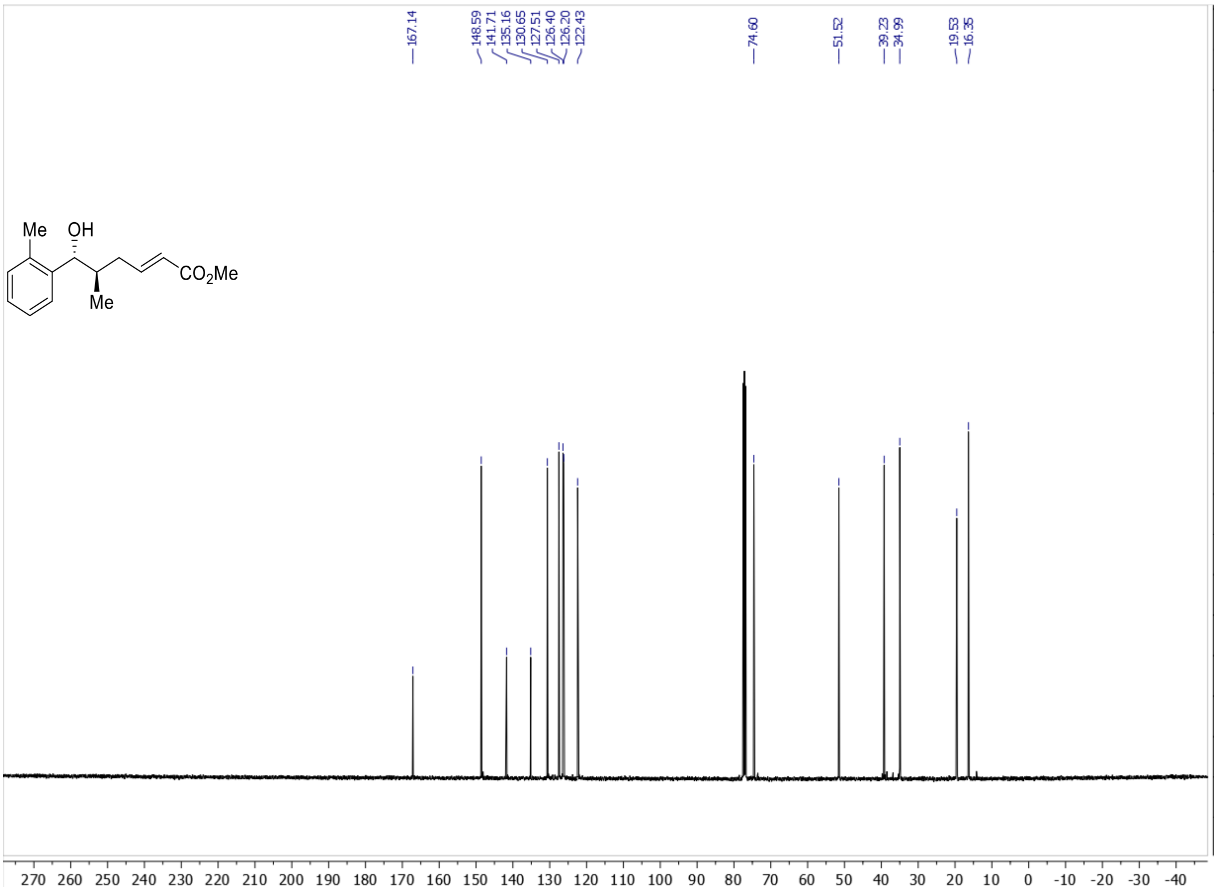

^{1}H NMR (400 MHz, $CDCl_3$; top) and ^{13}C NMR (101 MHz, $CDCl_3$; bottom) of compound **11**

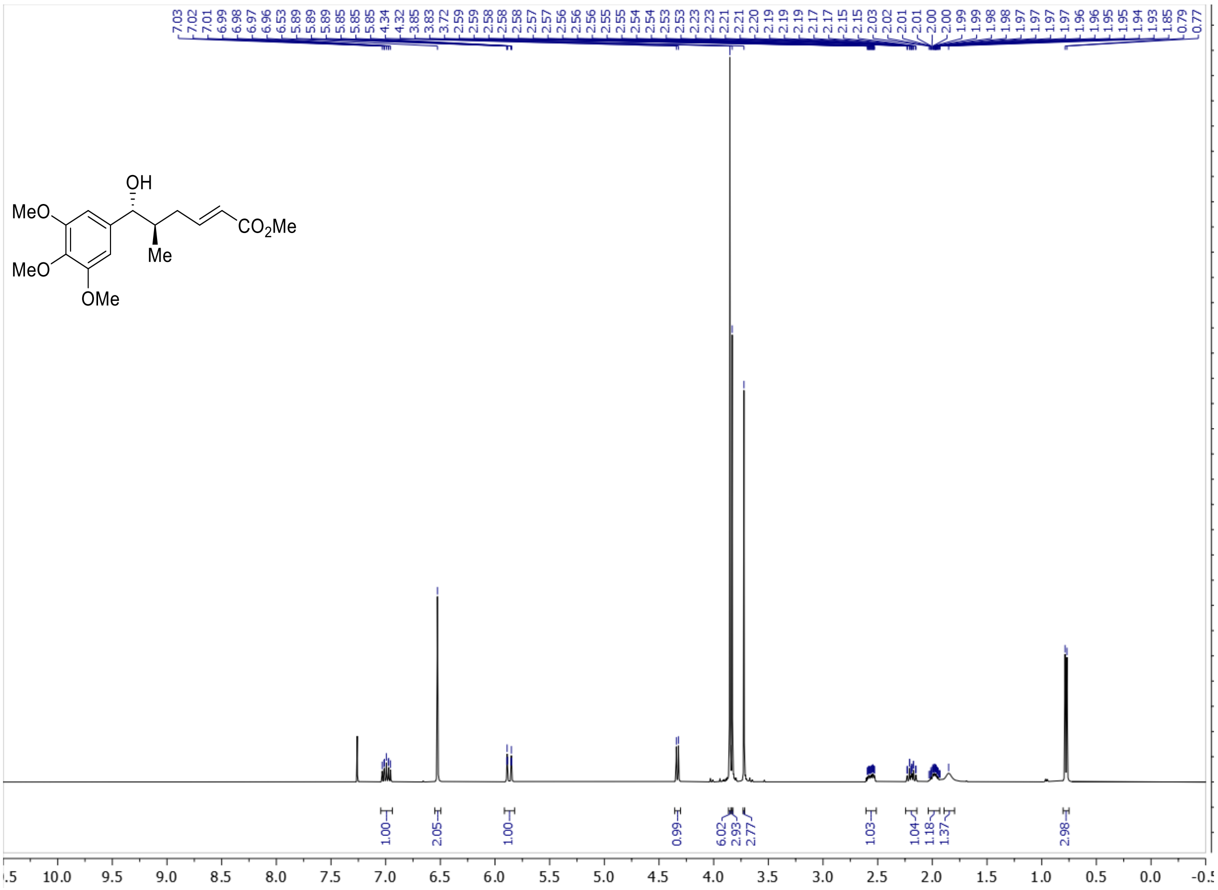

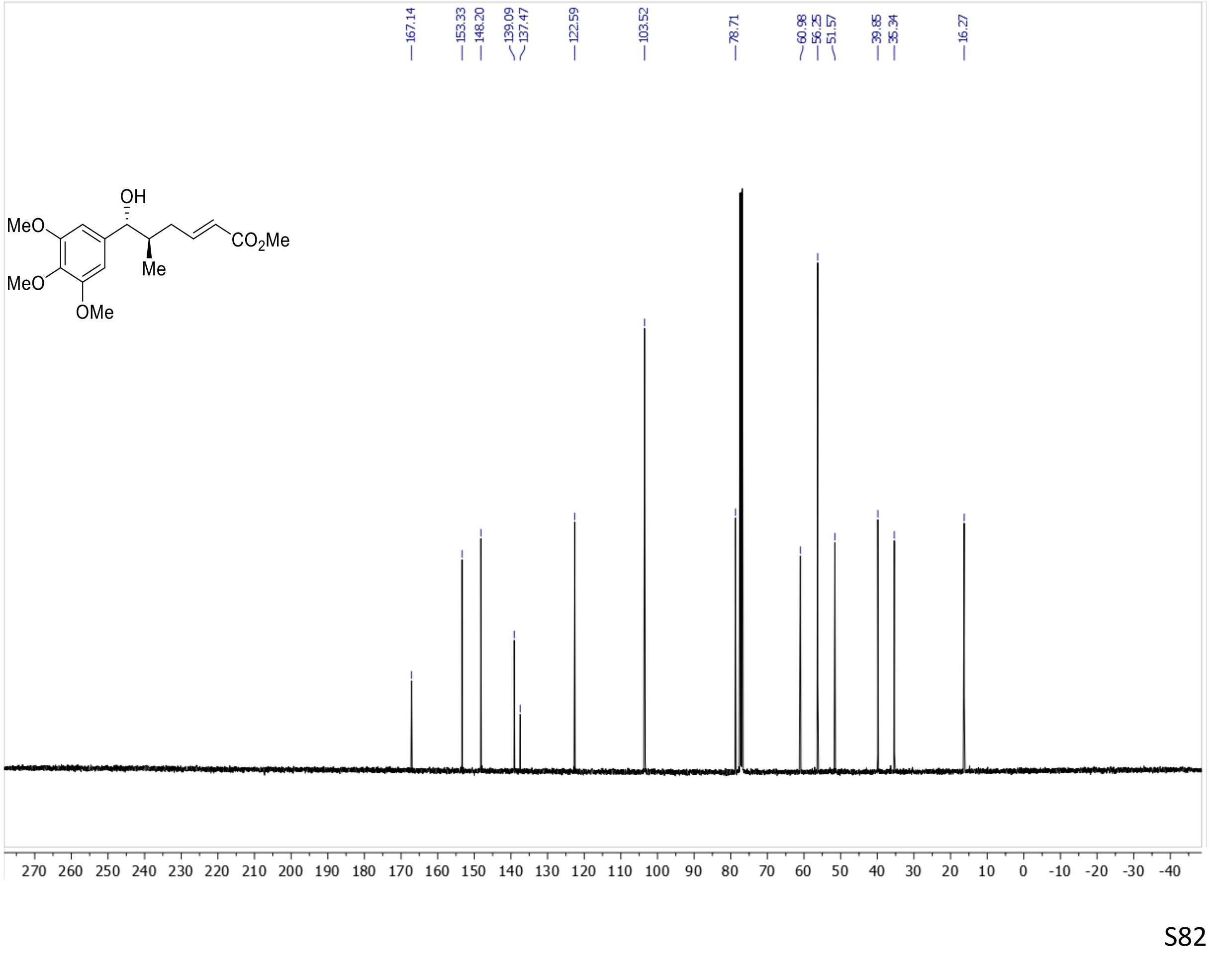

^1H NMR (400 MHz, $CDCl_3$; top) and ^{13}C NMR (101 MHz, $CDCl_3$; bottom) of compound **12**

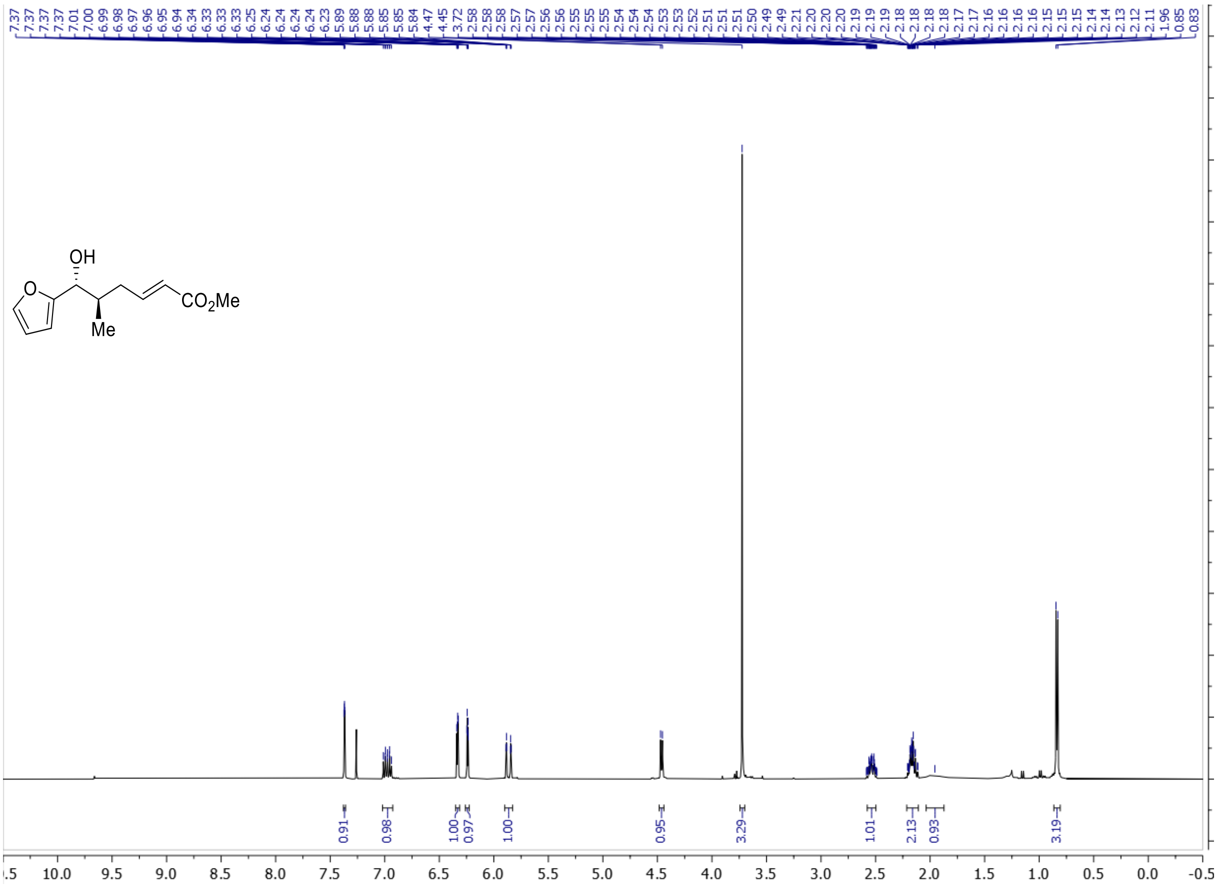

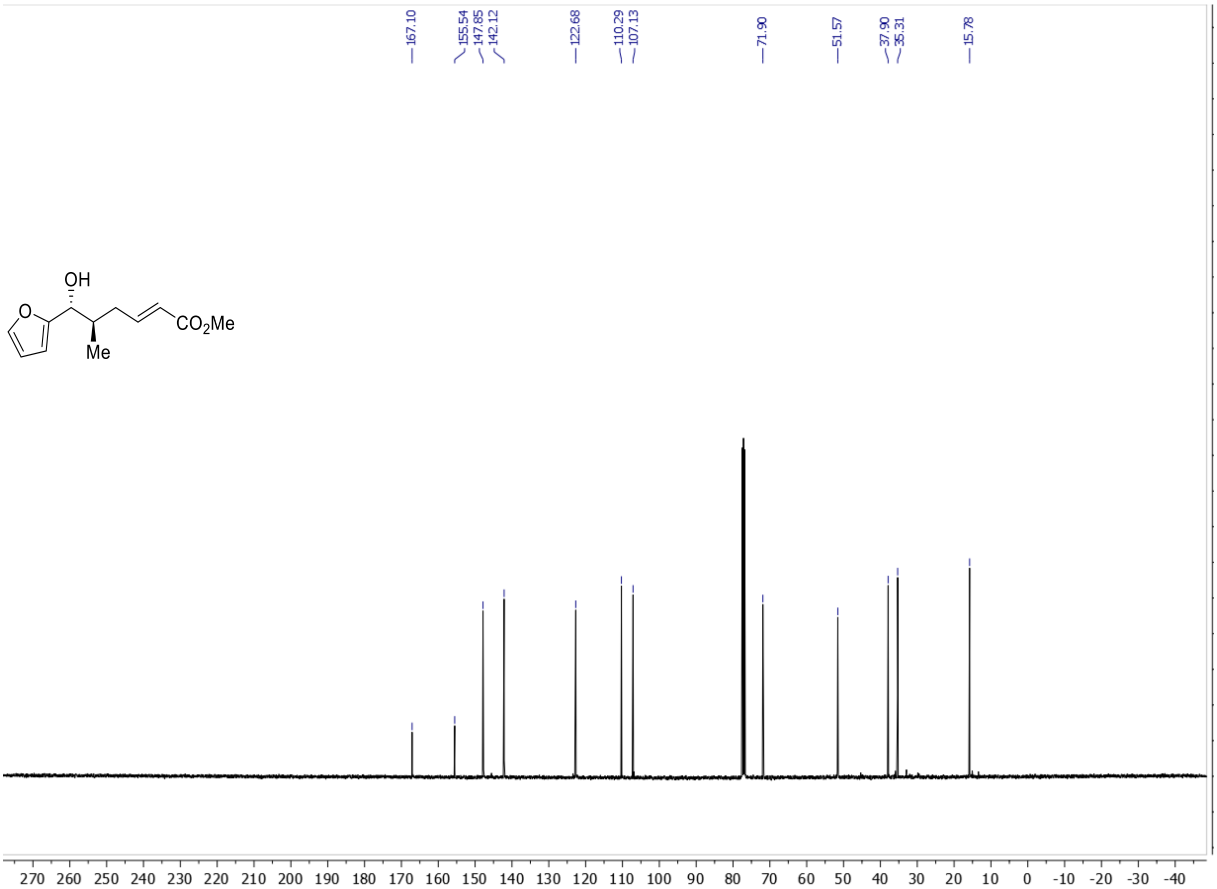

^{1}H NMR (400 MHz, $CDCl_3$; top) and ^{13}C NMR (101 MHz, $CDCl_3$; bottom) of compound **13**

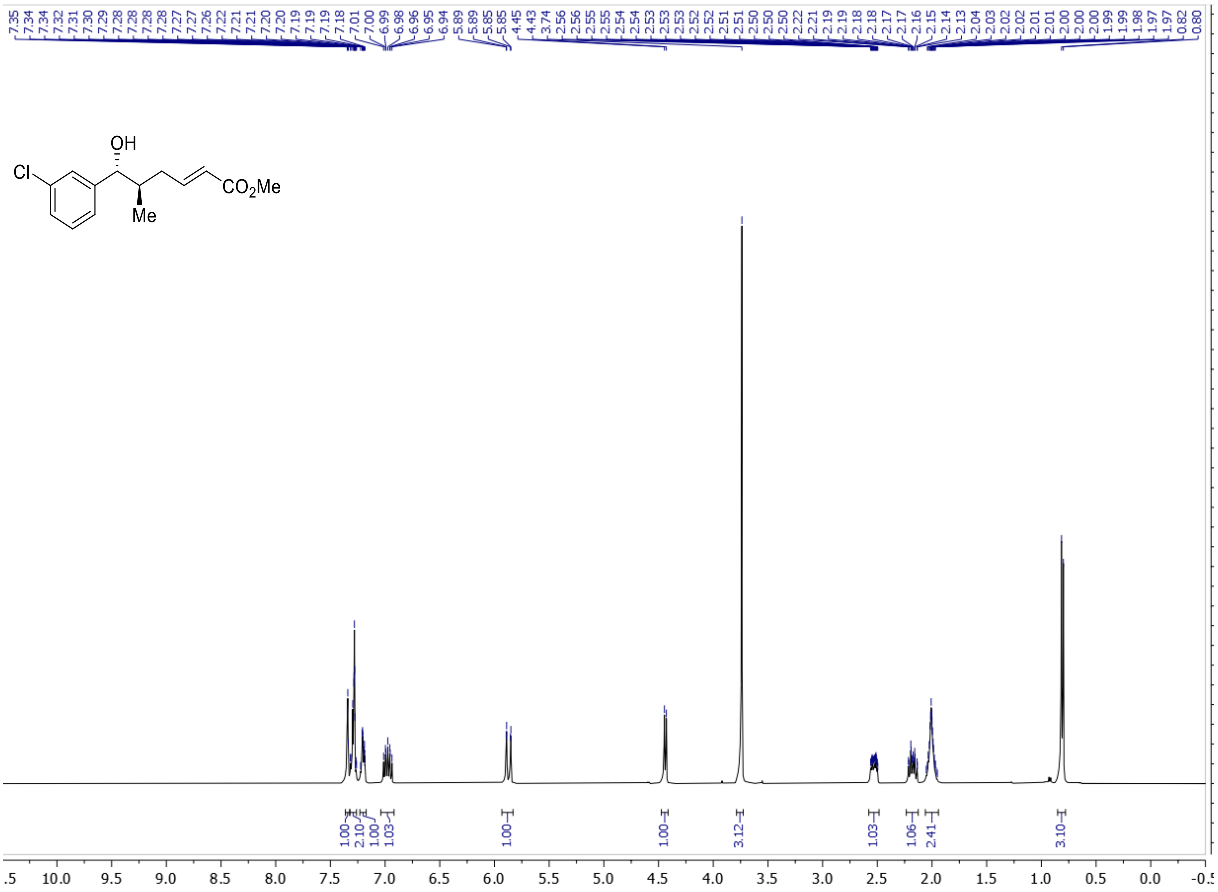

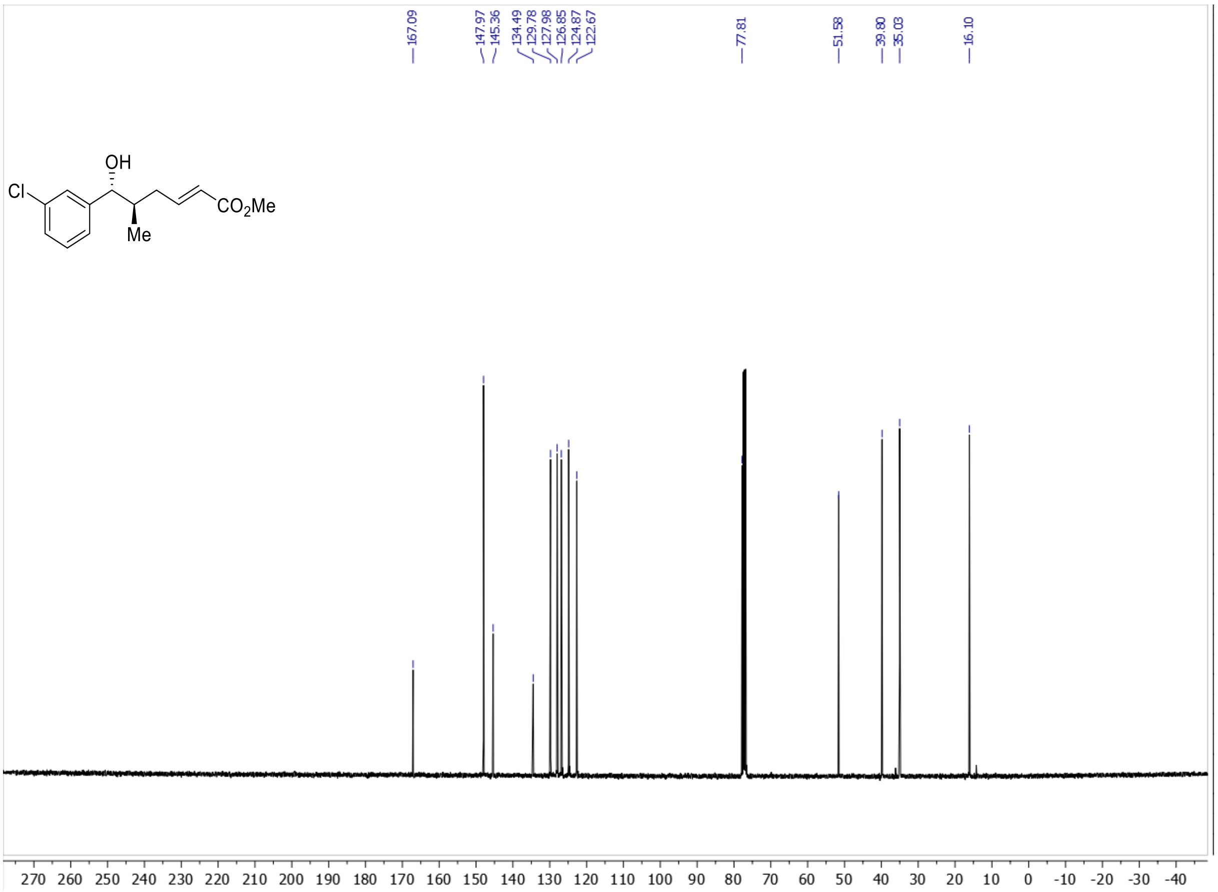

^{1}H NMR (400 MHz, $CDCl_3$; top) and ^{13}C NMR (101 MHz, $CDCl_3$; bottom) of compound **14**

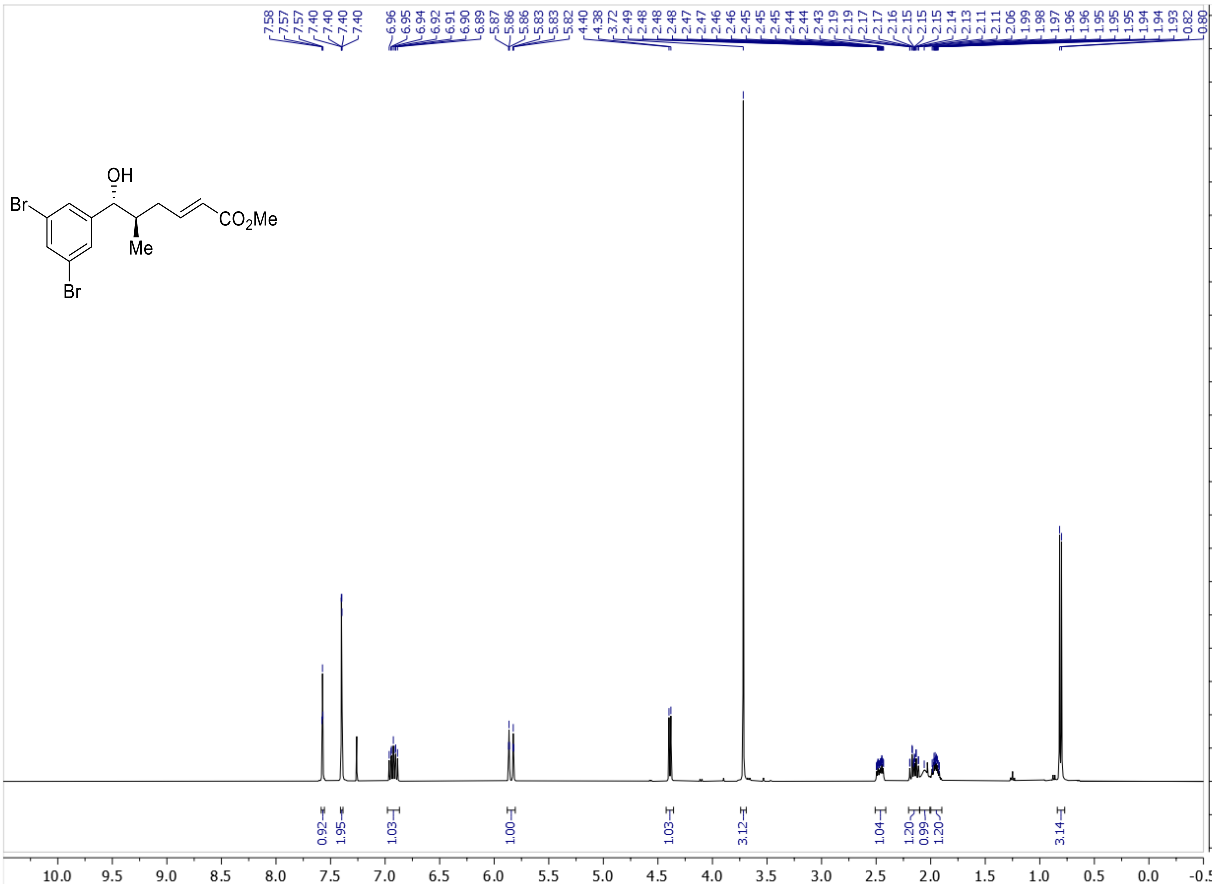

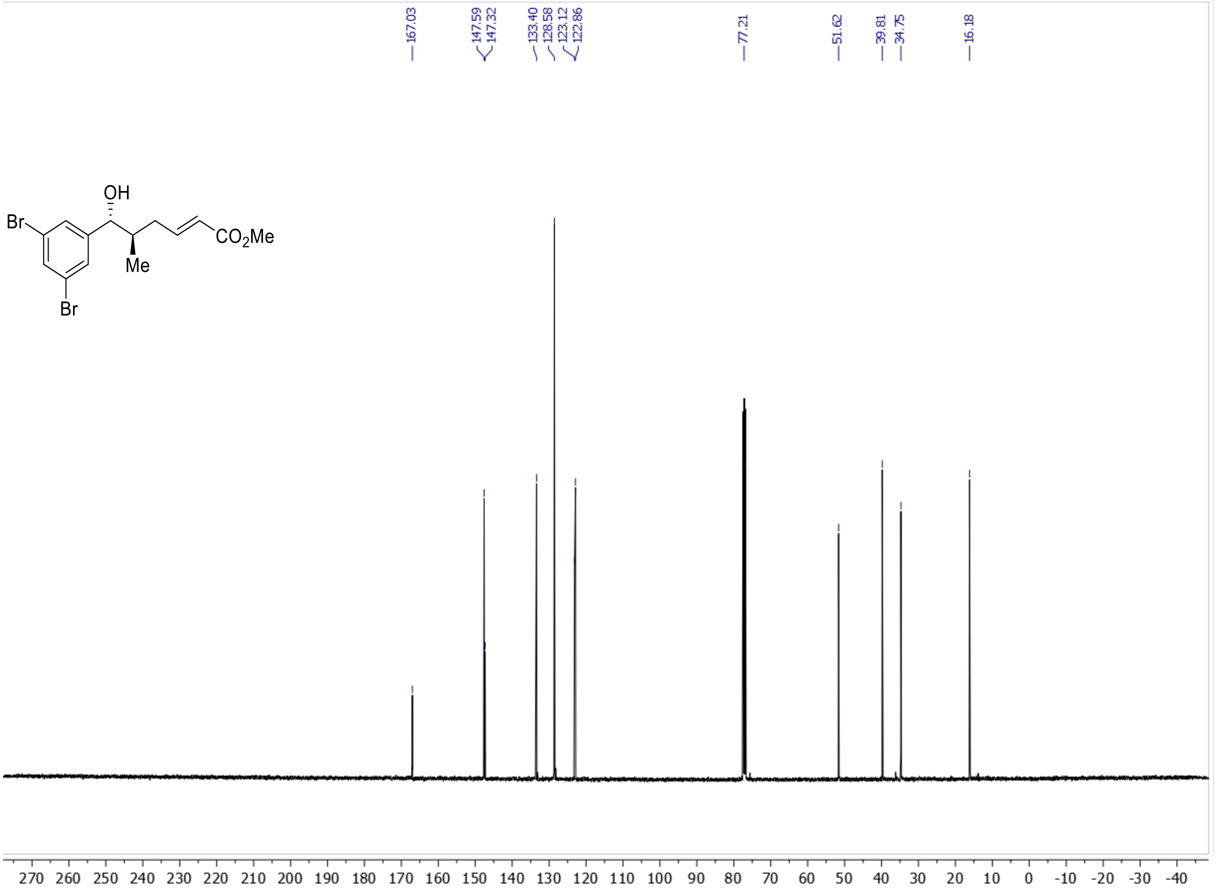

^{1}H NMR (400 MHz, $CDCl_3$; top) and ^{13}C NMR (101 MHz, $CDCl_3$; bottom) of compound **15a**

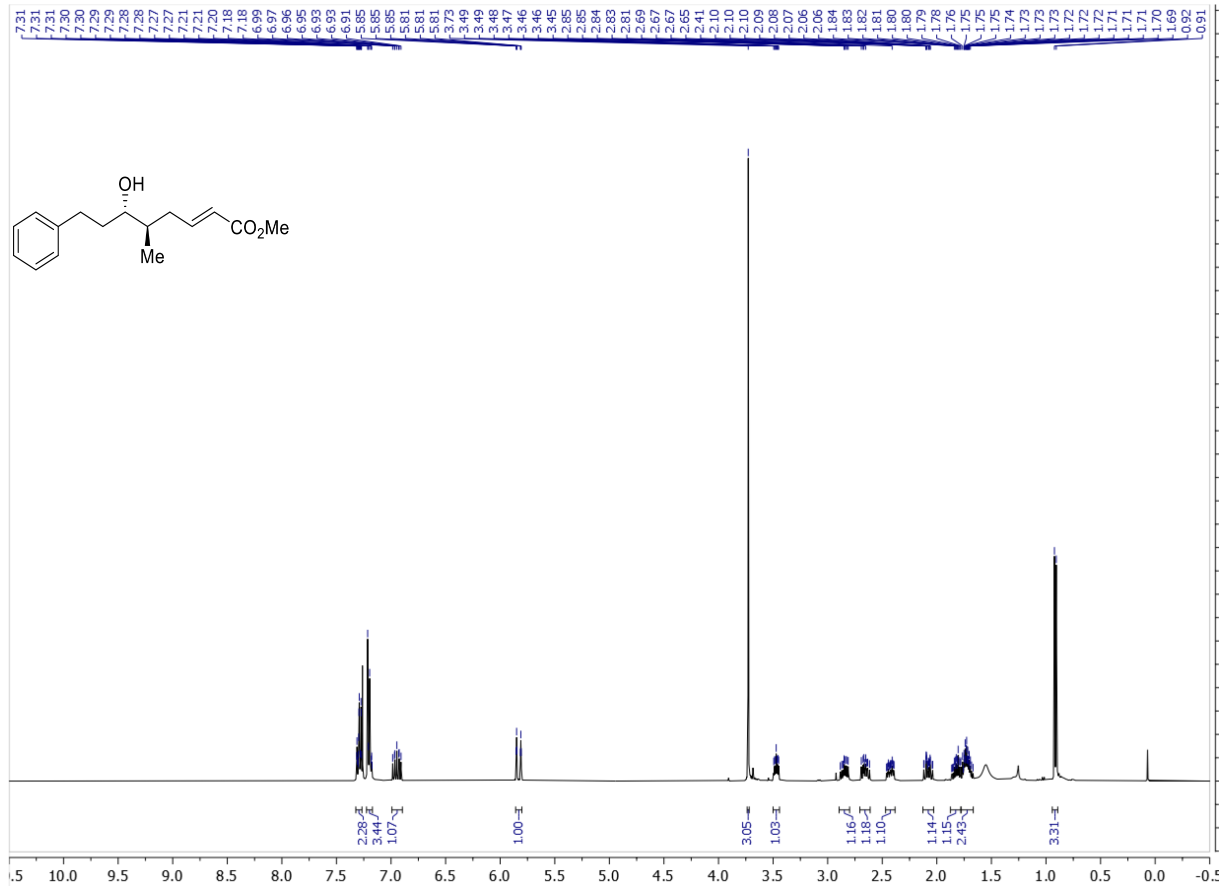

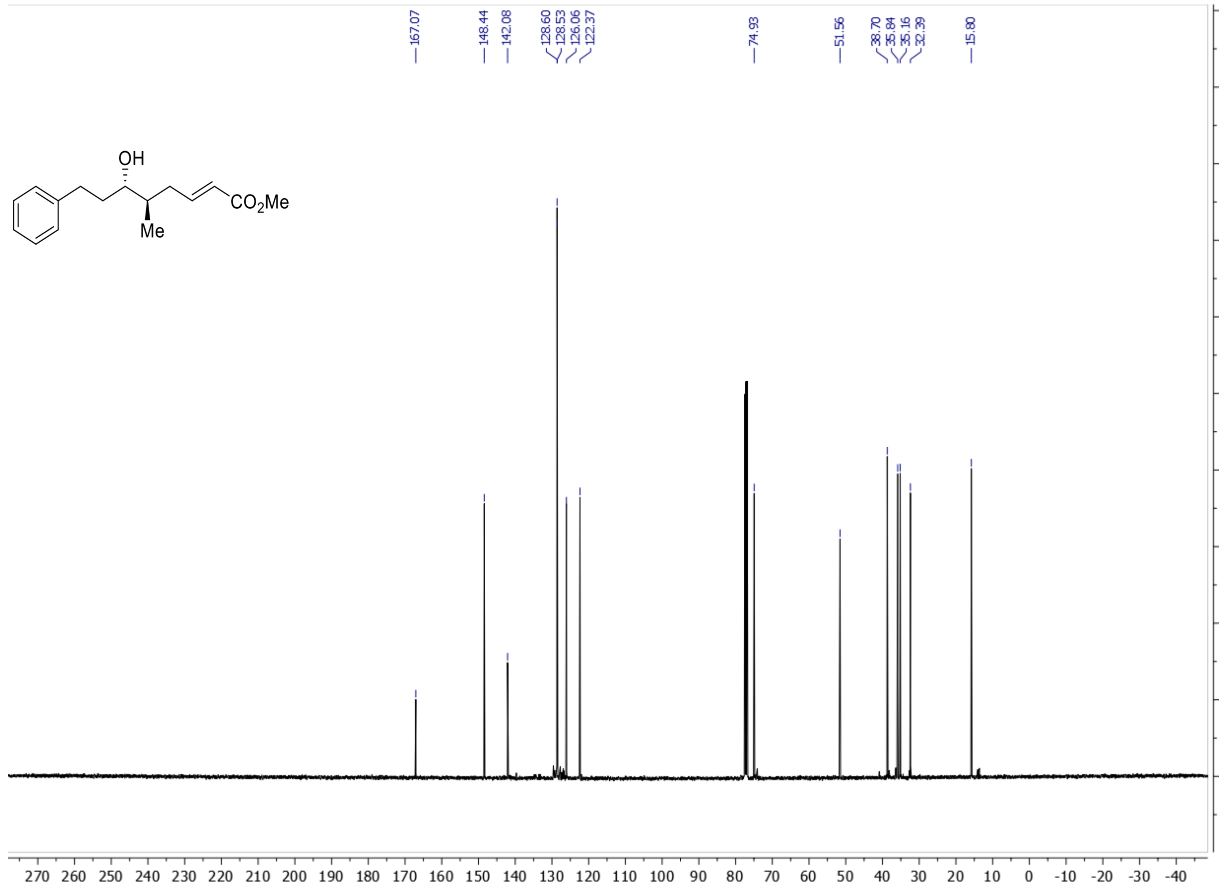

^{1}H NMR (400 MHz, $CDCl_3$; top) and ^{13}C NMR (101 MHz, $CDCl_3$; bottom) of compound **15b**

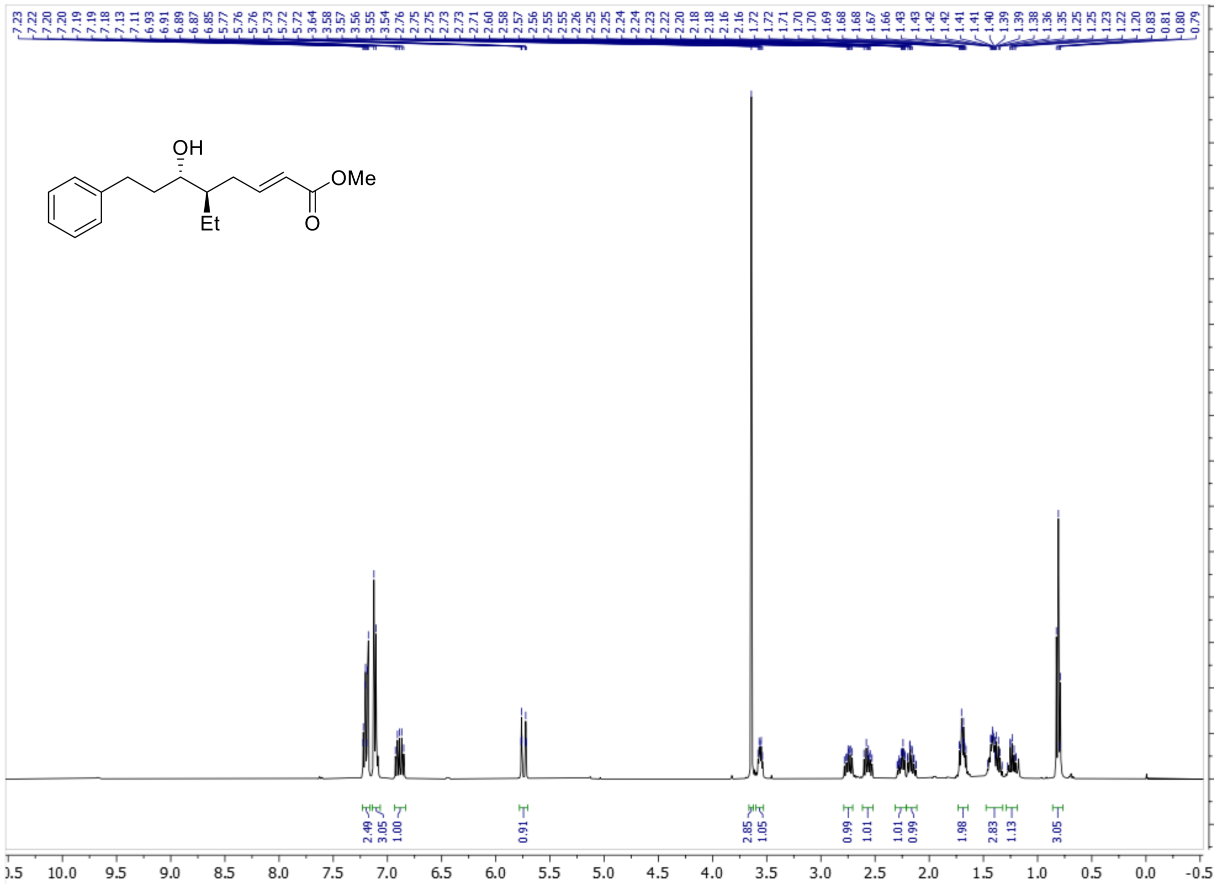

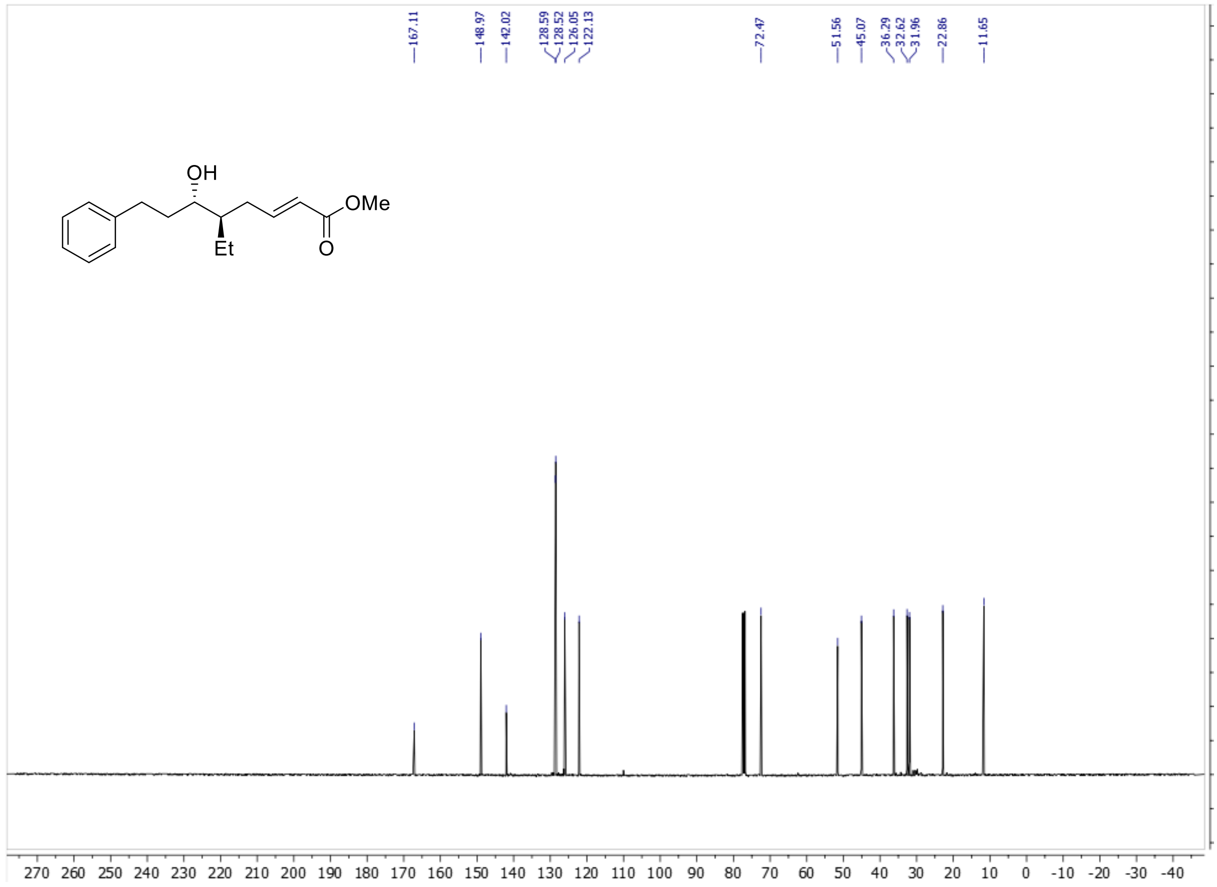

^{1}H NMR (400 MHz, $CDCl_3$; top) and ^{13}C NMR (101 MHz, $CDCl_3$; bottom) of compound **15c**

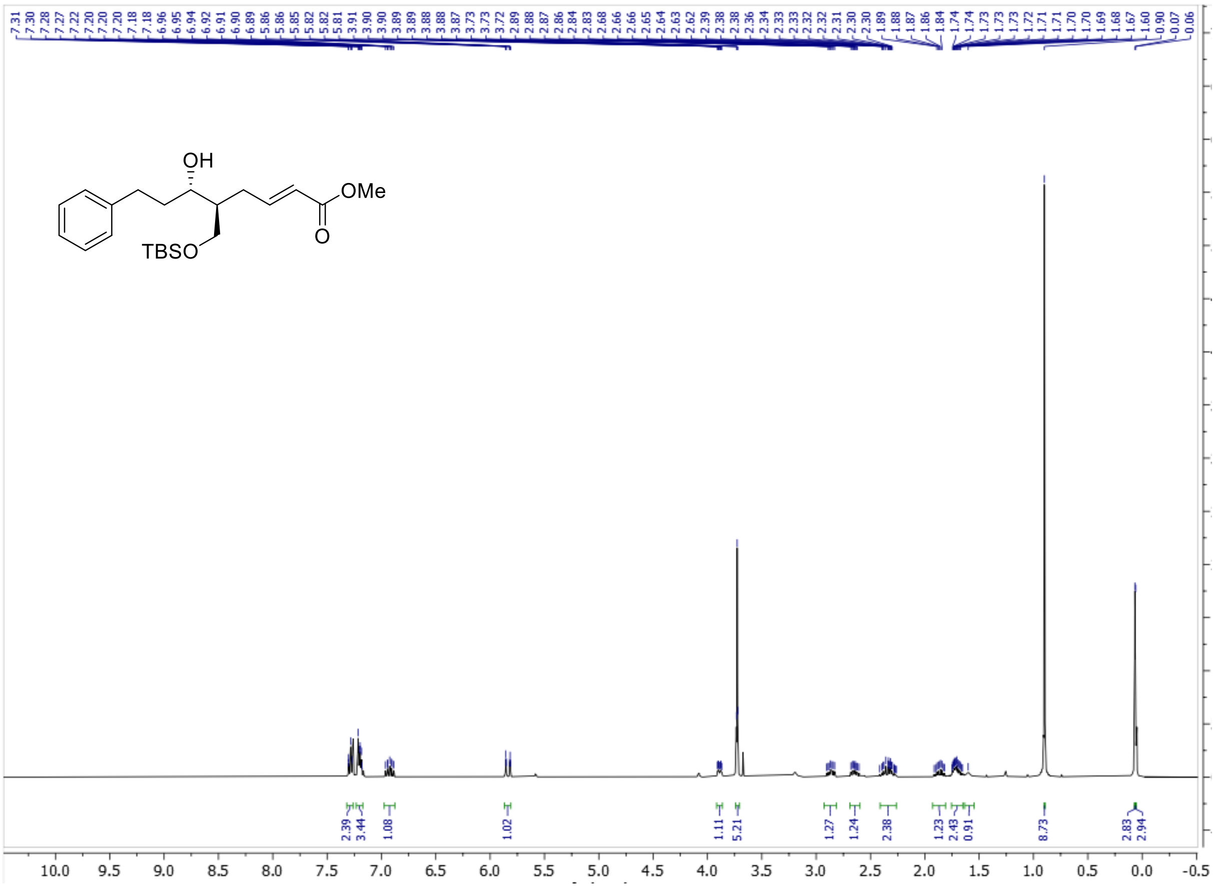

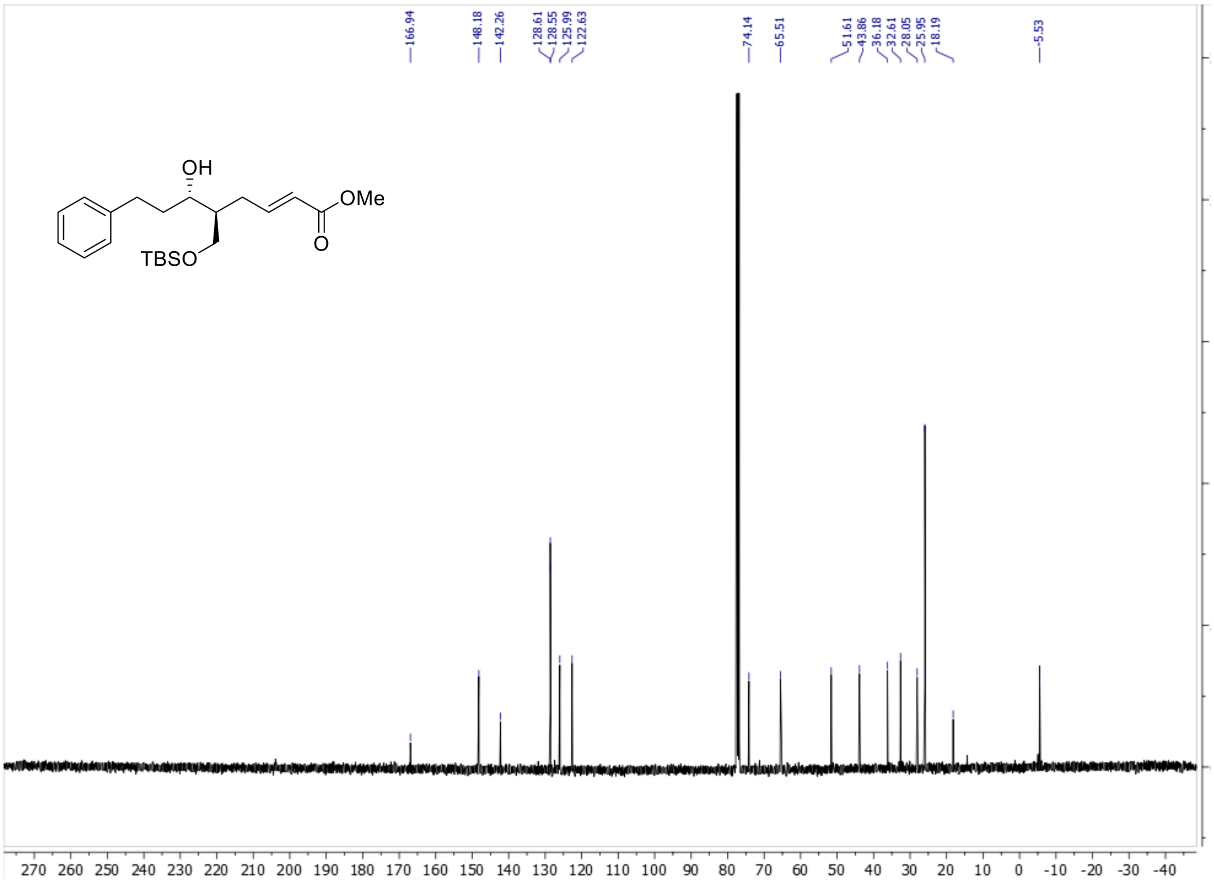

^{1}H NMR (400 MHz, $CDCl_3$; top) and ^{13}C NMR (101 MHz, $CDCl_3$; bottom) of compound **15d**

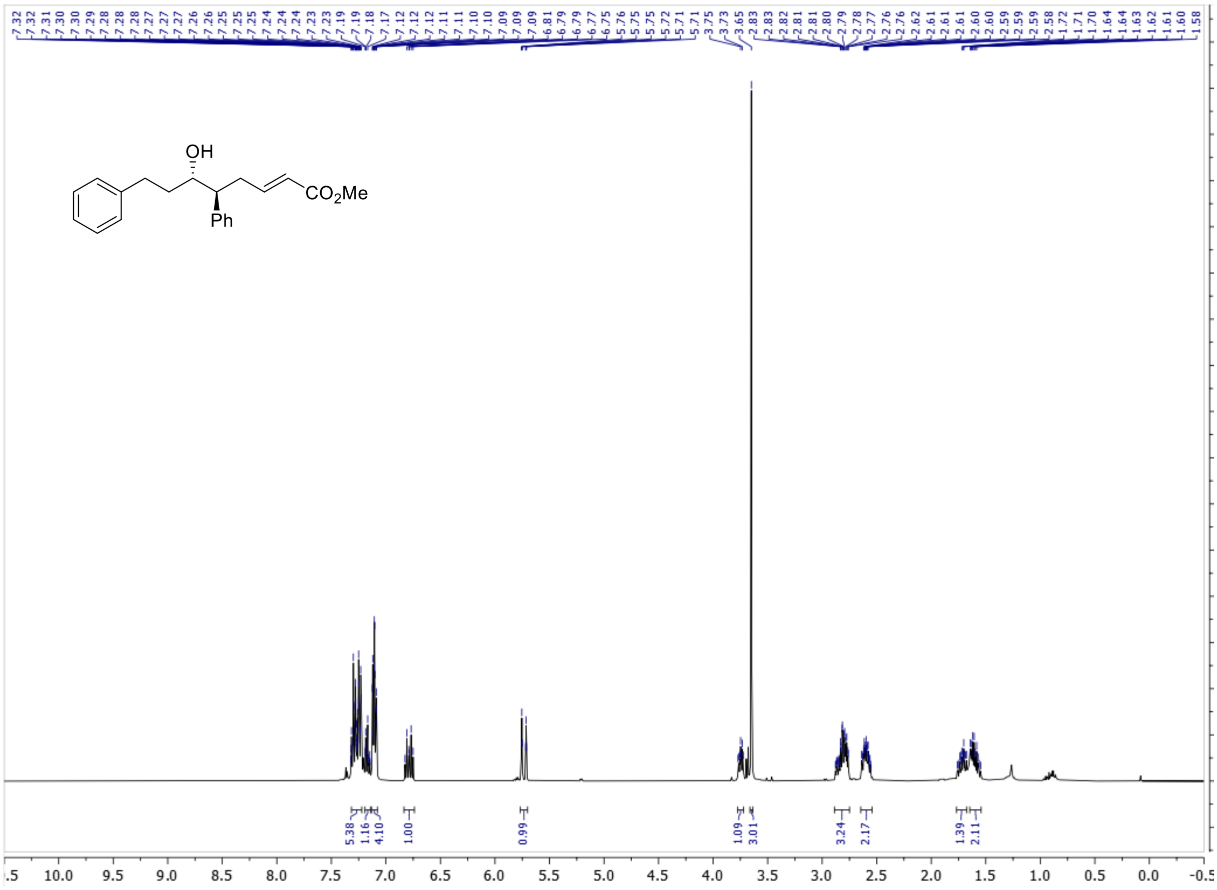

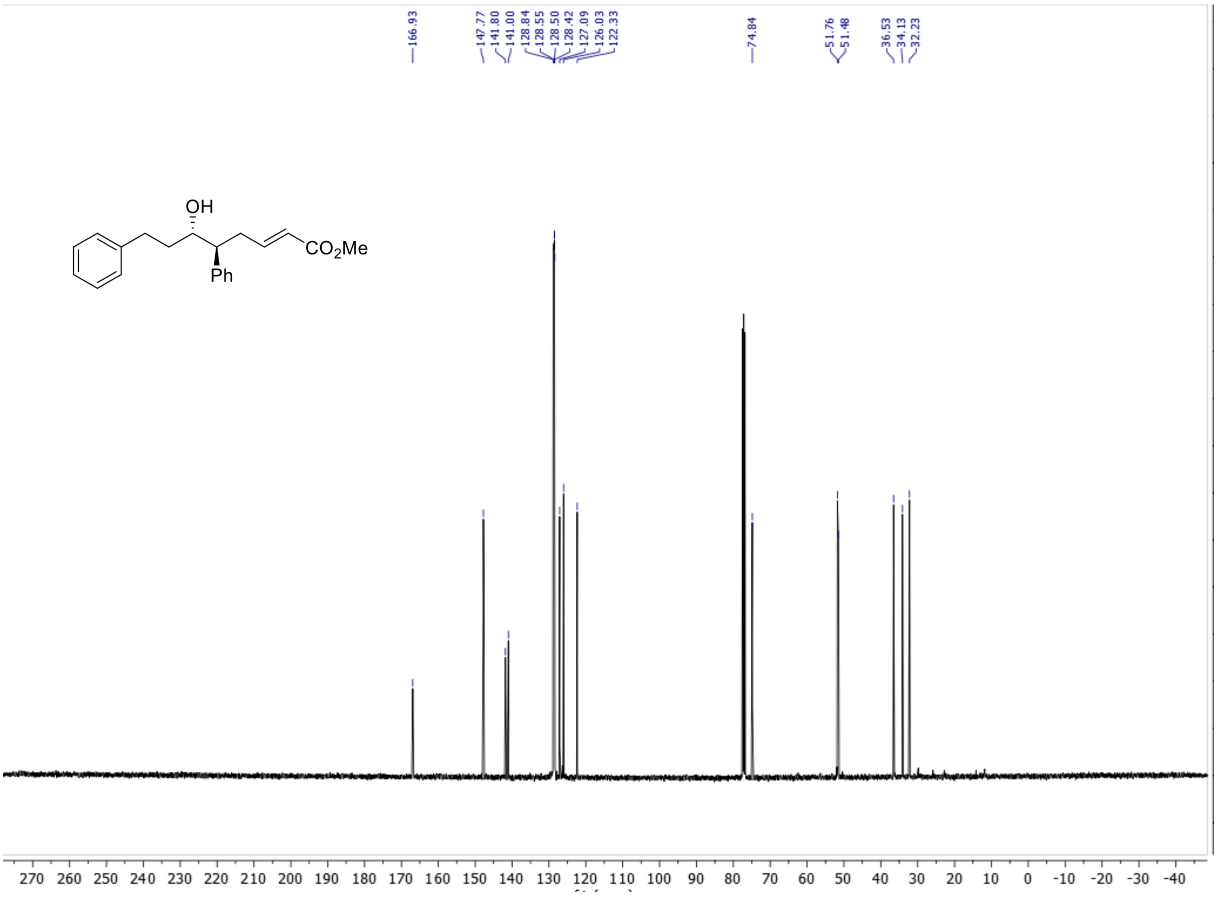

^{1}H NMR (400 MHz, $CDCl_3$; top) and ^{13}C NMR (101 MHz, $CDCl_3$; bottom) of compound **15d'**

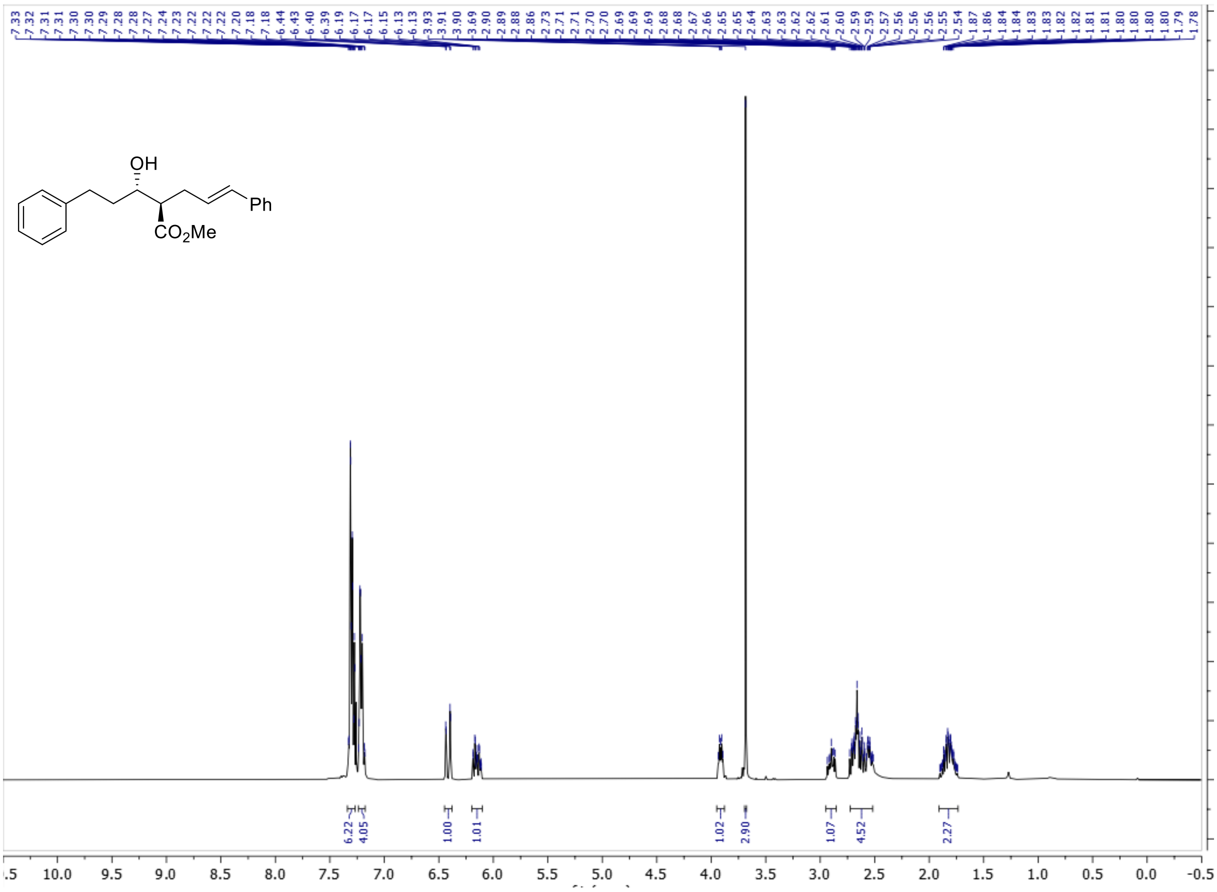

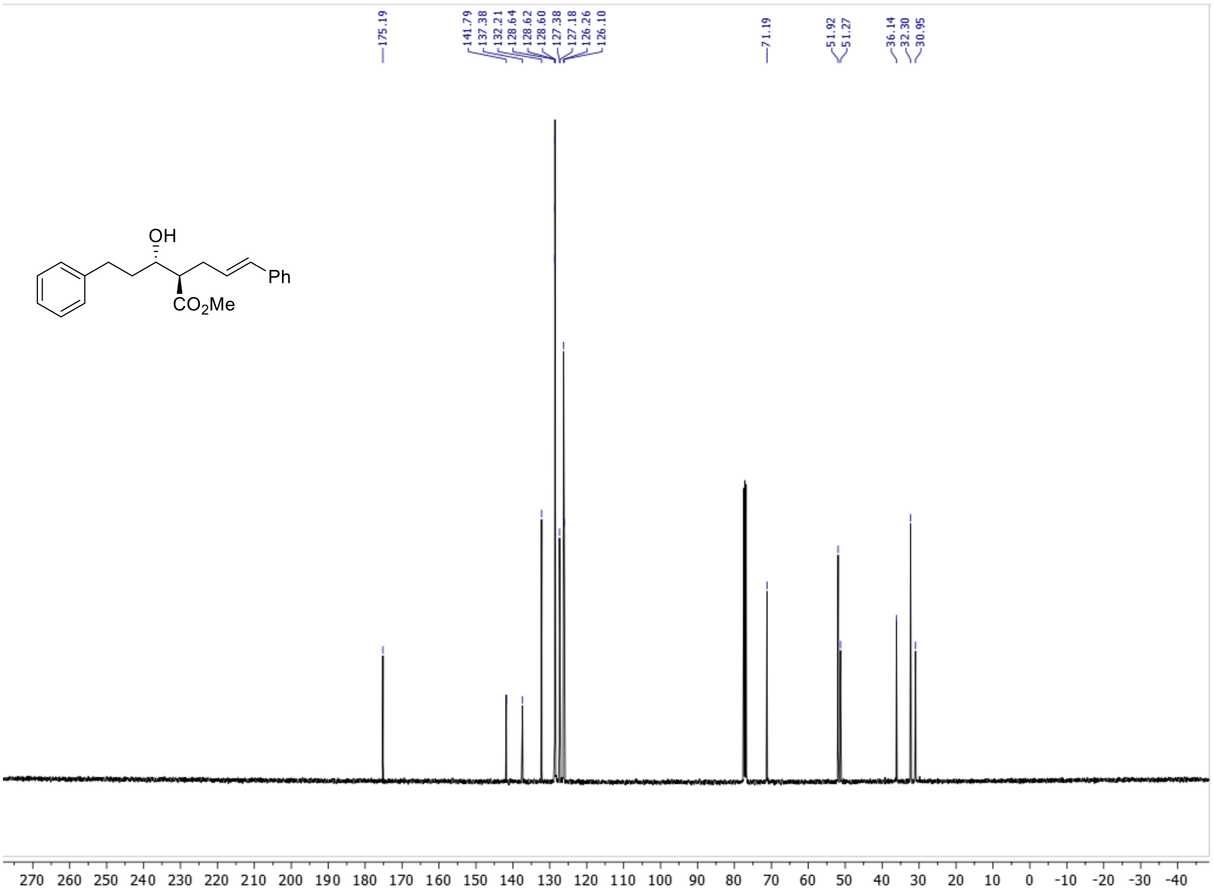

^{1}H NMR (400 MHz, $CDCl_3$; top) and ^{13}C NMR (101 MHz, $CDCl_3$; bottom) of compound **16**

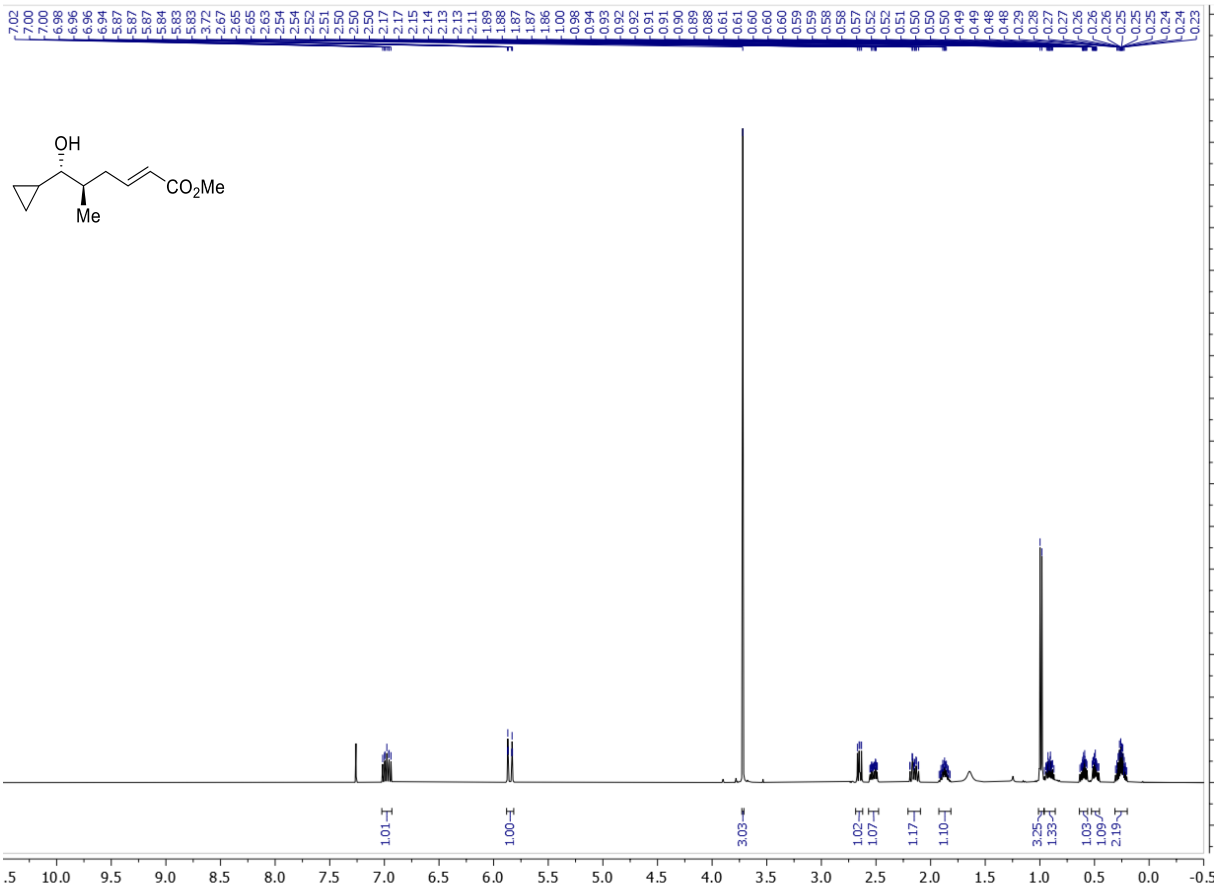

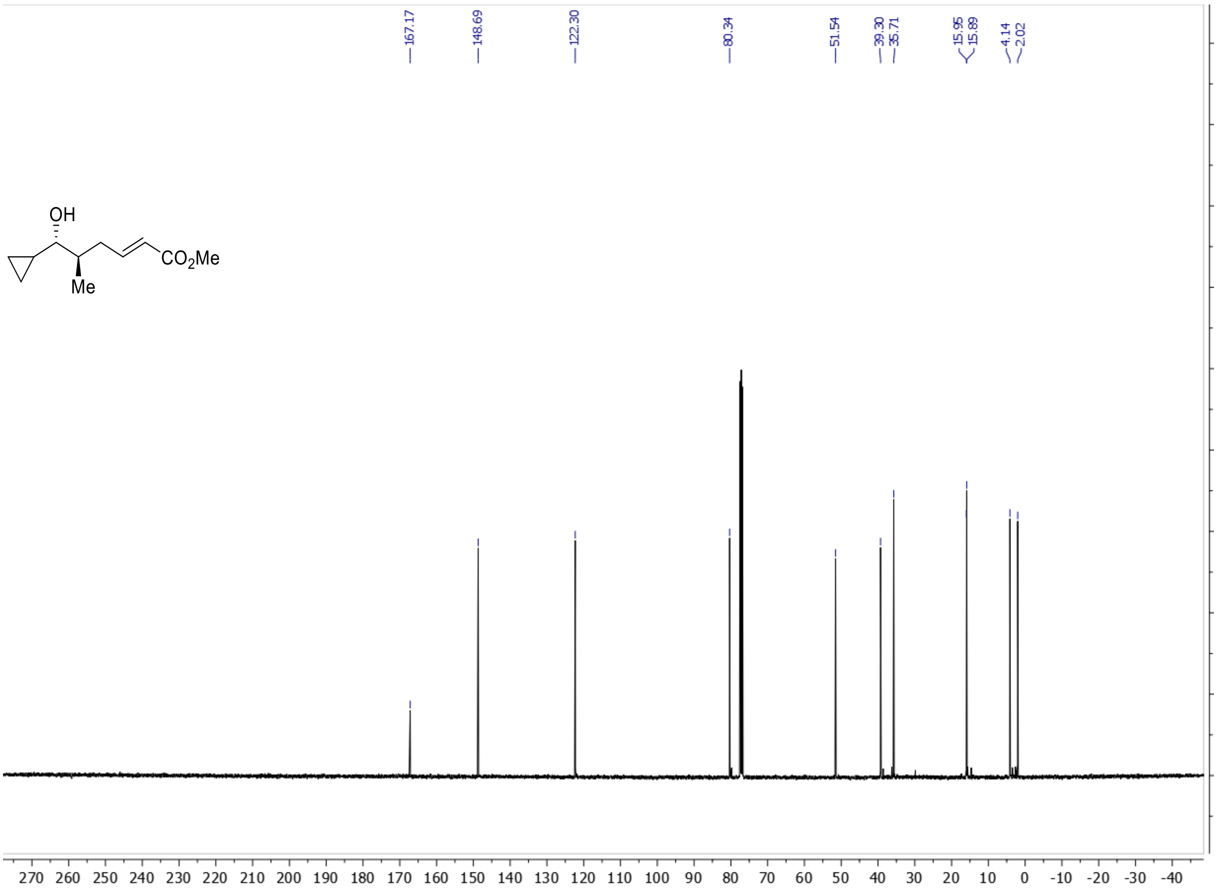

^{1}H NMR (400 MHz, $CDCl_3$; top) and ^{13}C NMR (101 MHz, $CDCl_3$; bottom) of compound **17**

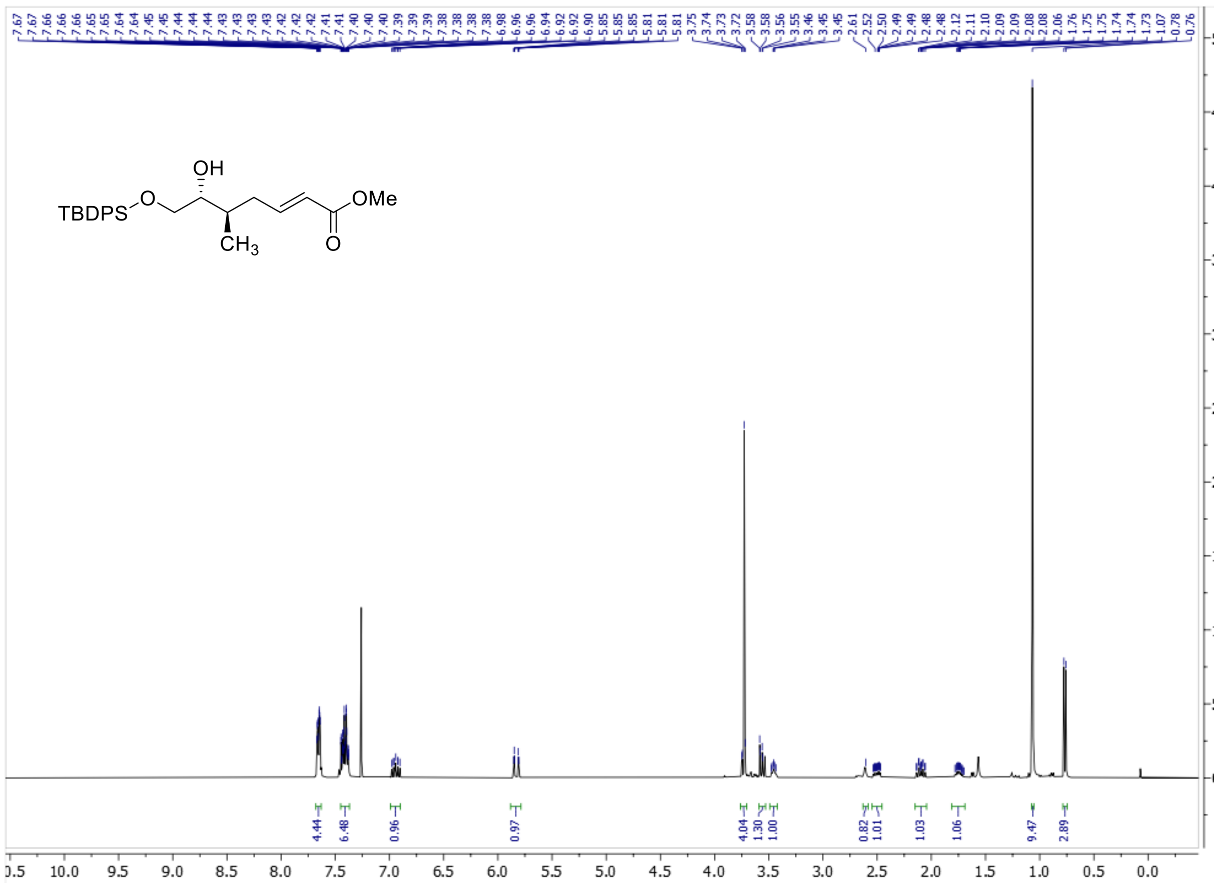

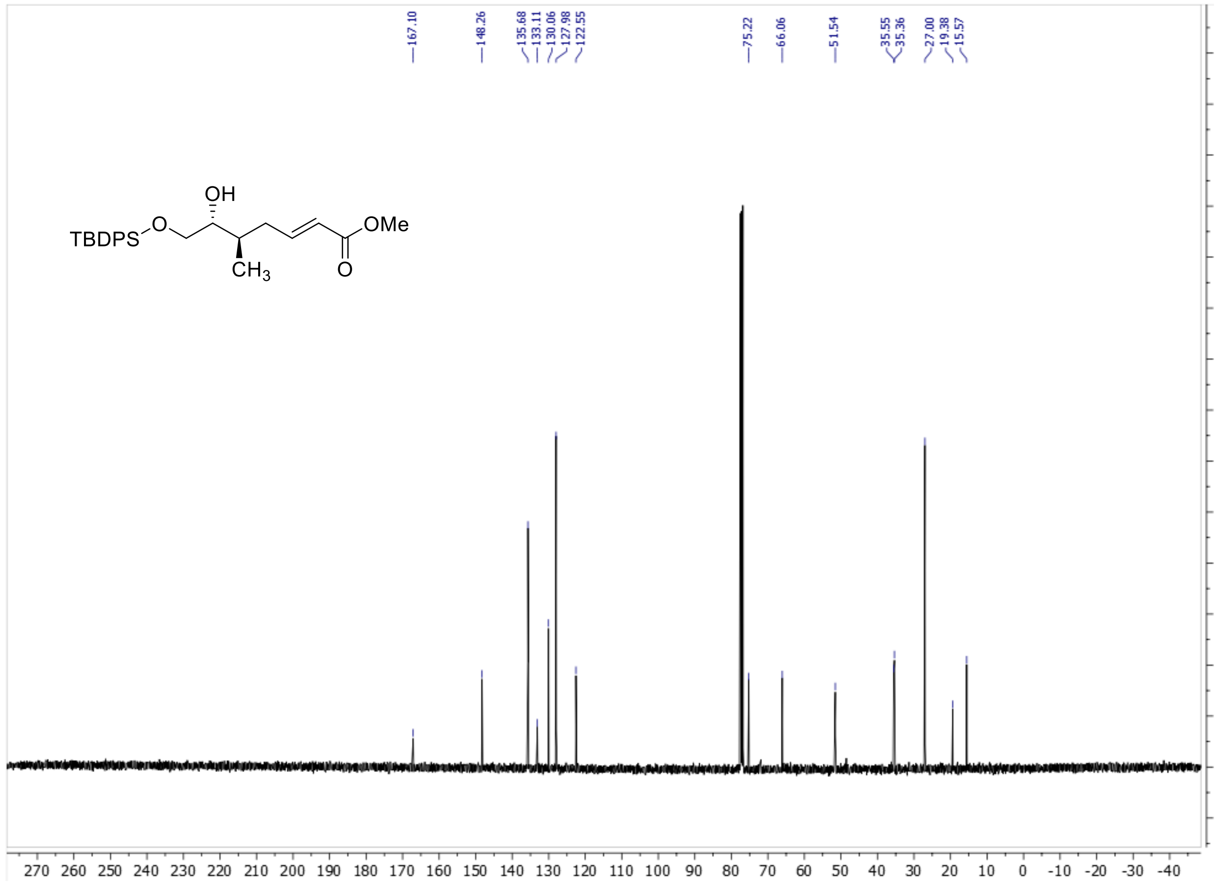

^{1}H NMR (400 MHz, $CDCl_3$; top) and ^{13}C NMR (101 MHz, $CDCl_3$; bottom) of compound **18**

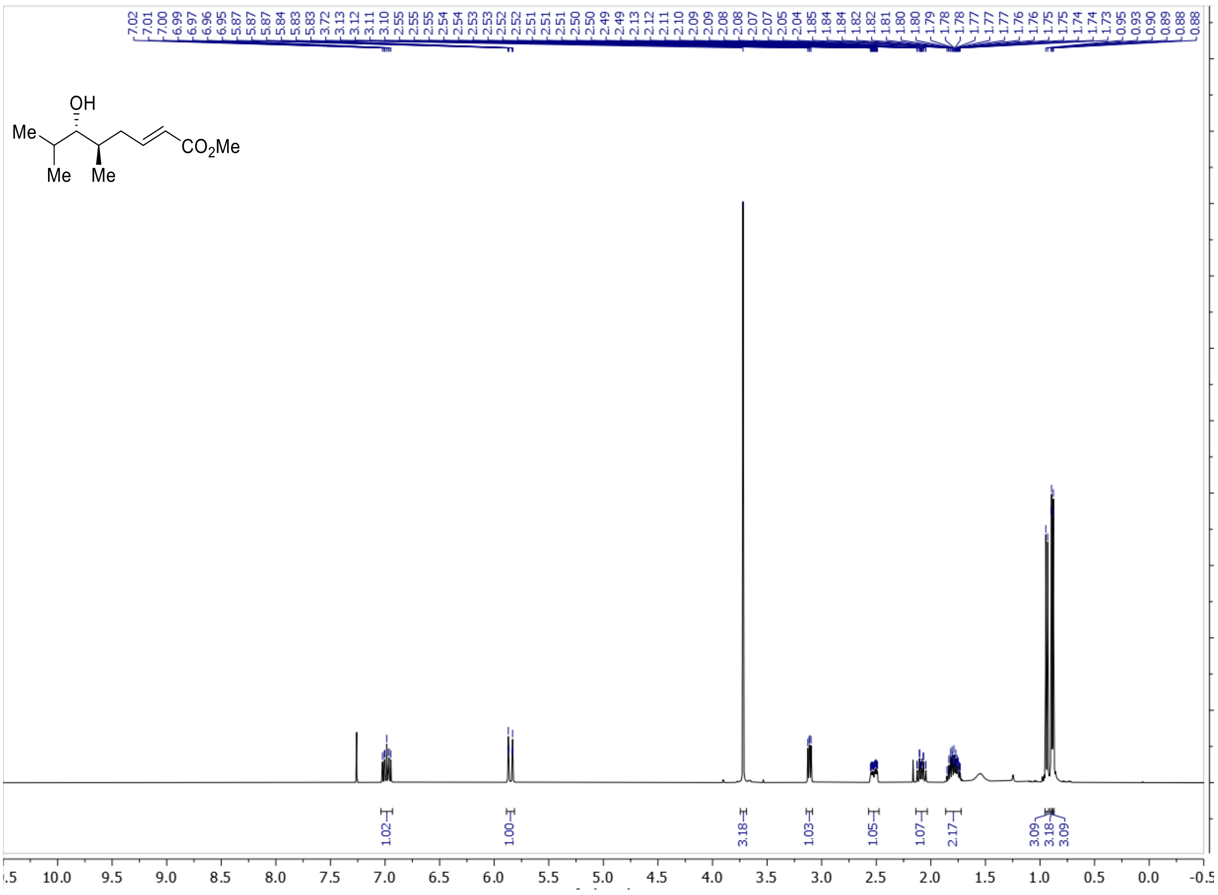

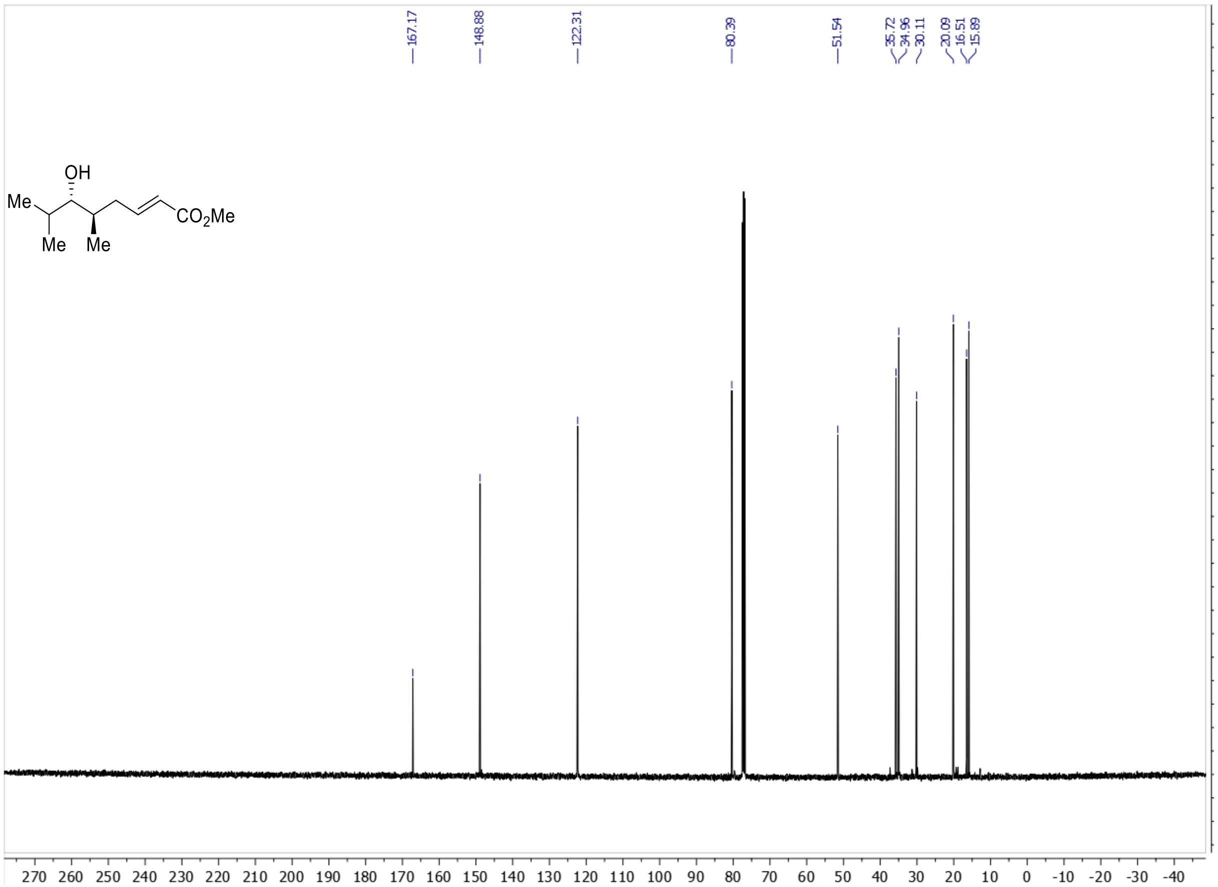

^{1}H NMR (400 MHz, $CDCl_3$; top) and ^{13}C NMR (101 MHz, $CDCl_3$; bottom) of compound **19**

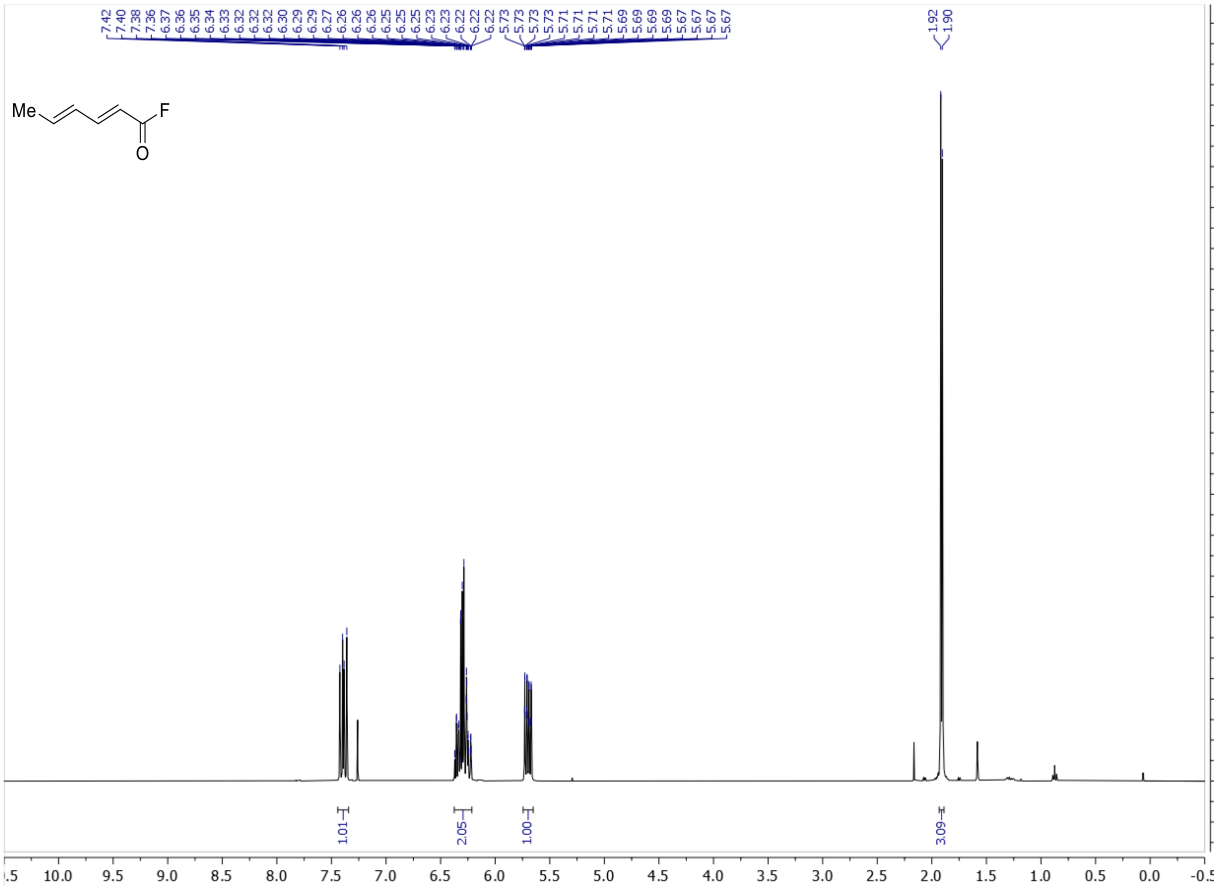

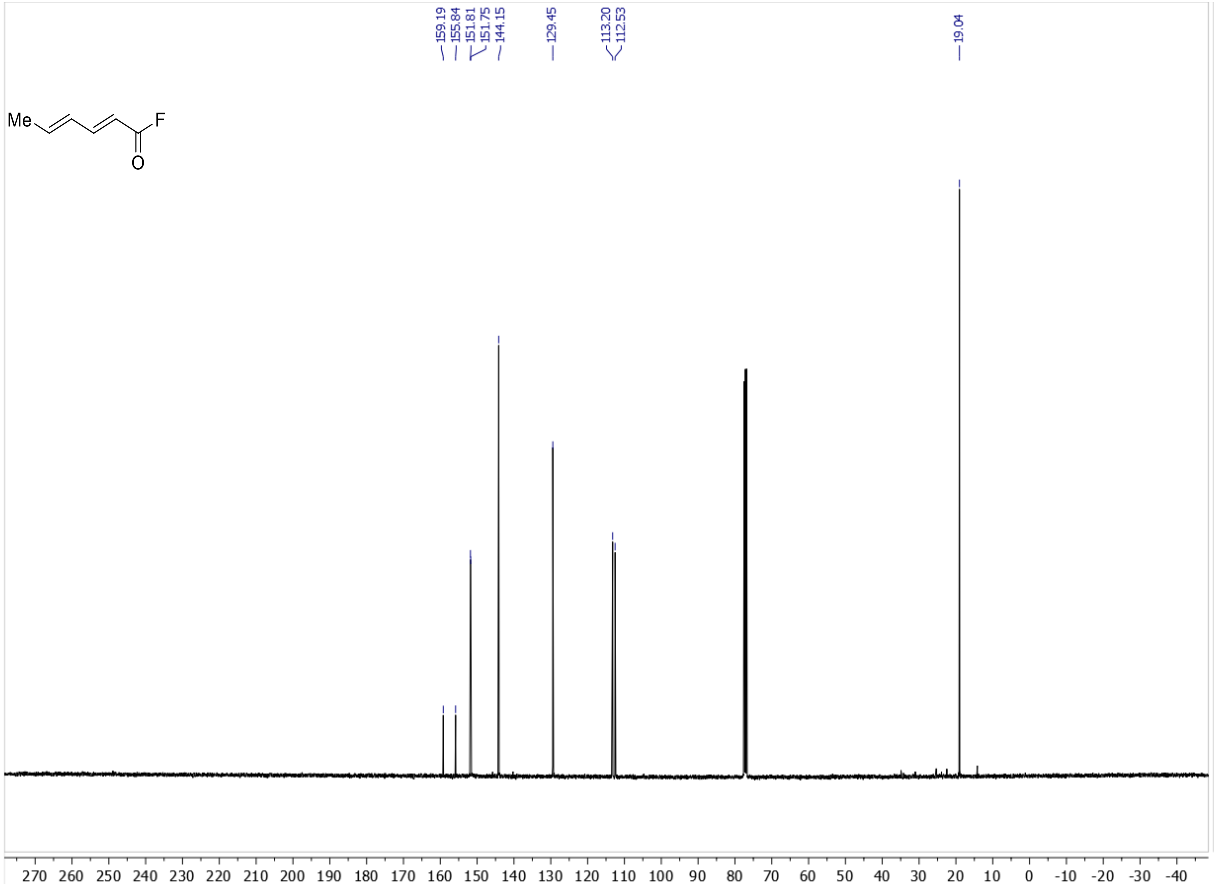

^{19}F NMR (282 MHz, $CDCl_3$) of compound **19**

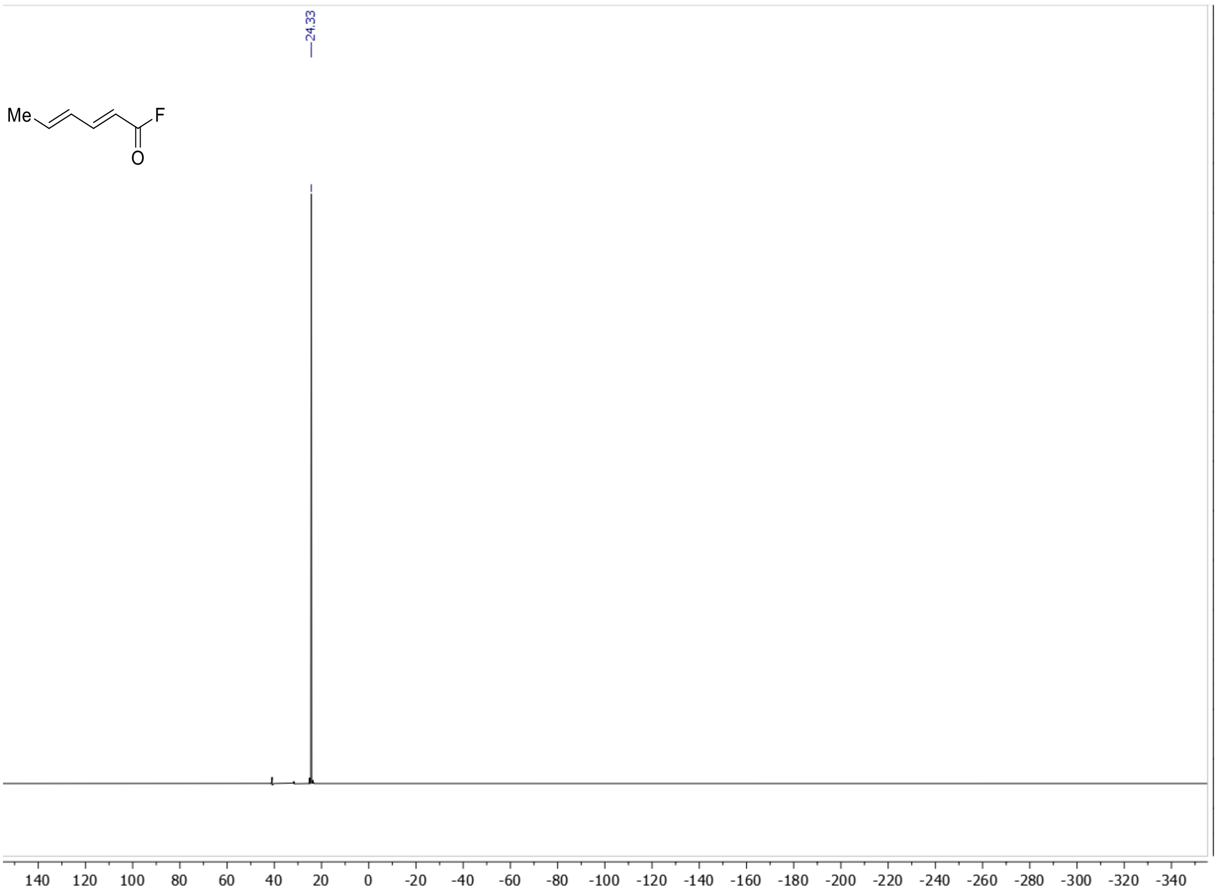

^{1}H NMR (400 MHz, $CDCl_3$; top), ^{13}C NMR (101 MHz, $CDCl_3$; bottom) of compound **20**

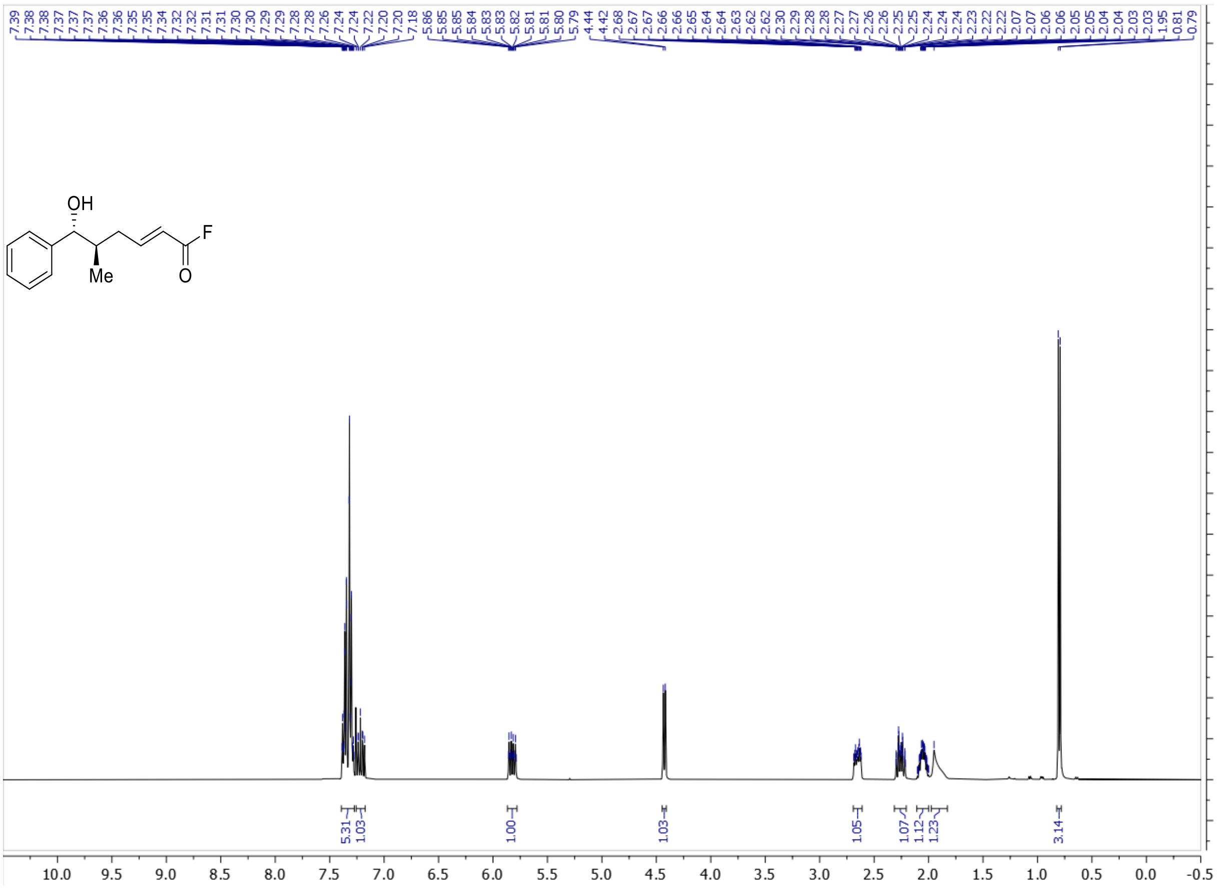

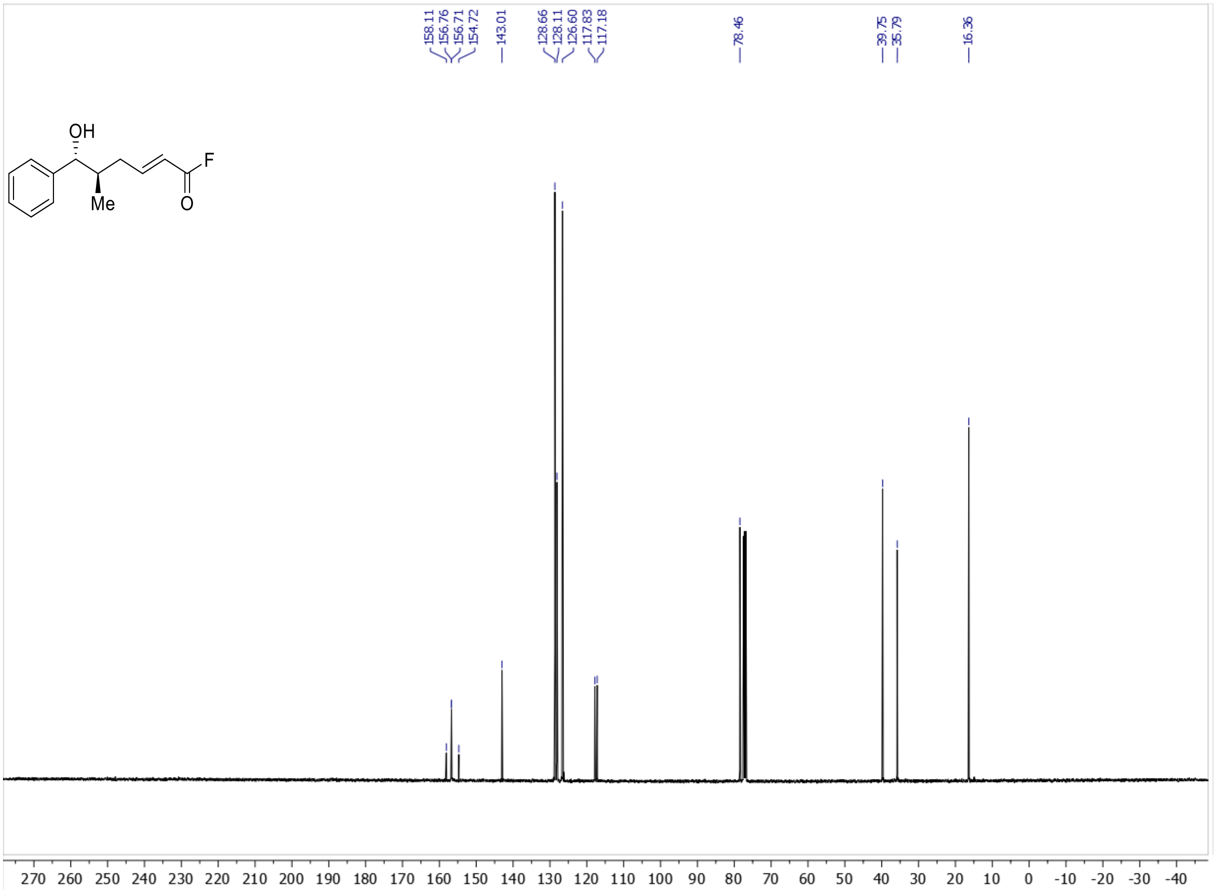

^{19}F NMR (282 MHz, $CDCl_3$) of compound **20**

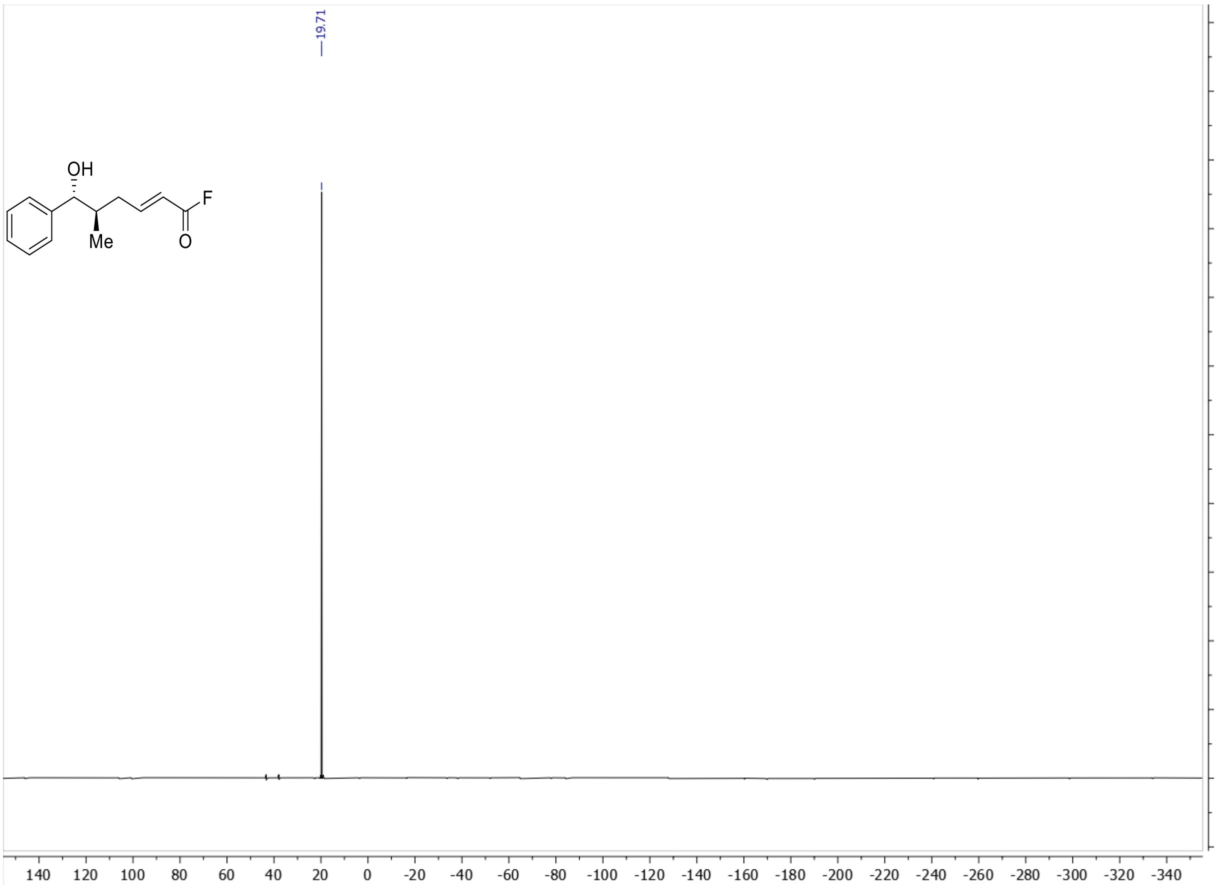

^{1}H NMR (400 MHz, $CDCl_3$; top) and ^{13}C NMR (101 MHz, $CDCl_3$; bottom) of compound **22**

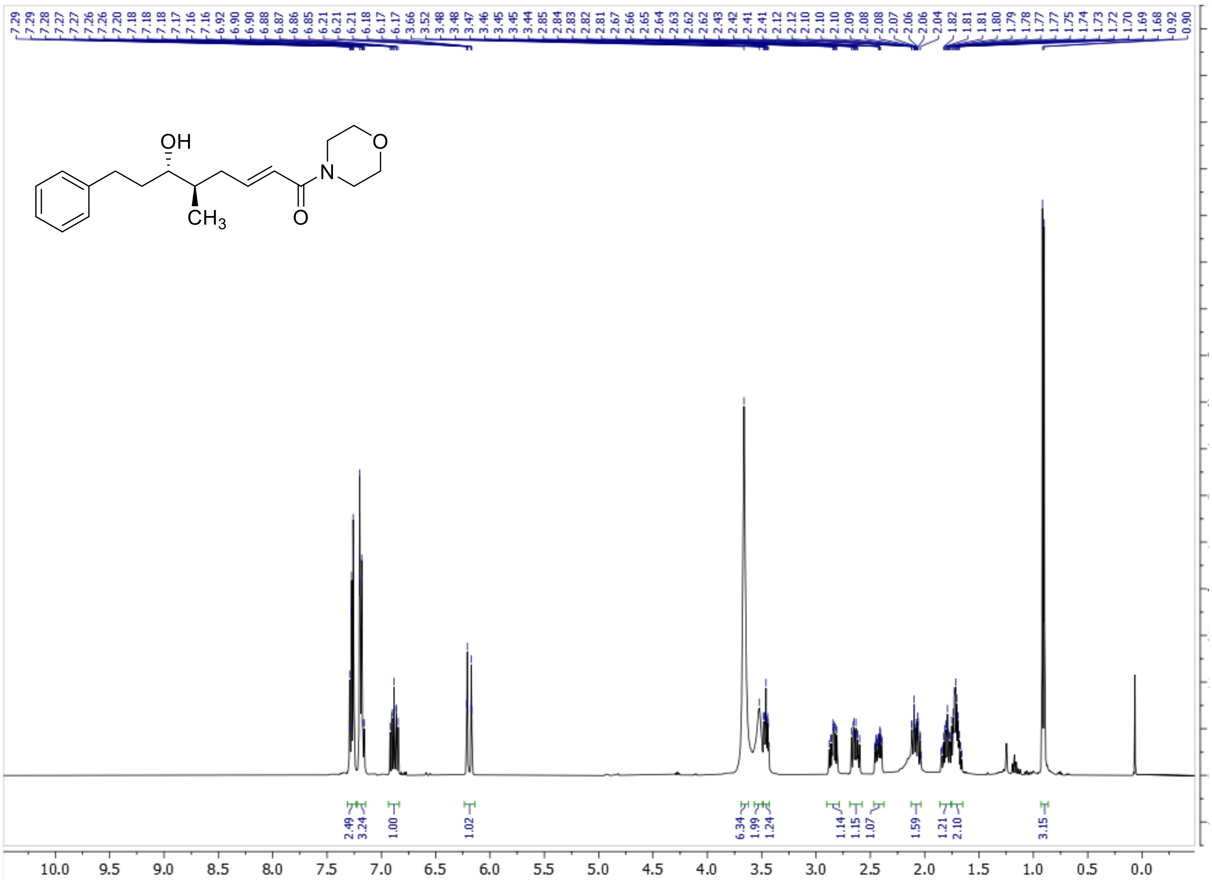

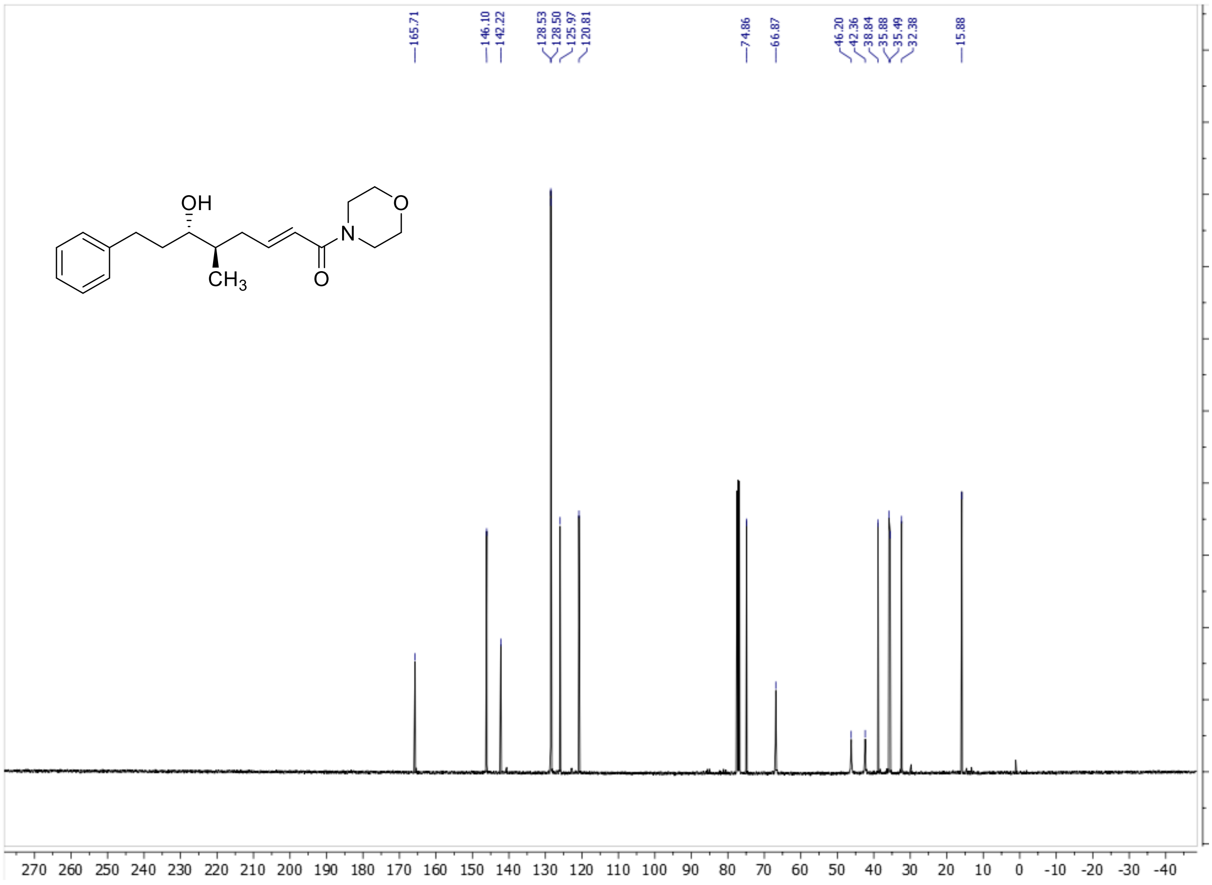

^{1}H NMR (400 MHz, C_6D_6; top), ^{13}C NMR (101 MHz, C_6D_6; bottom) of compound **23**

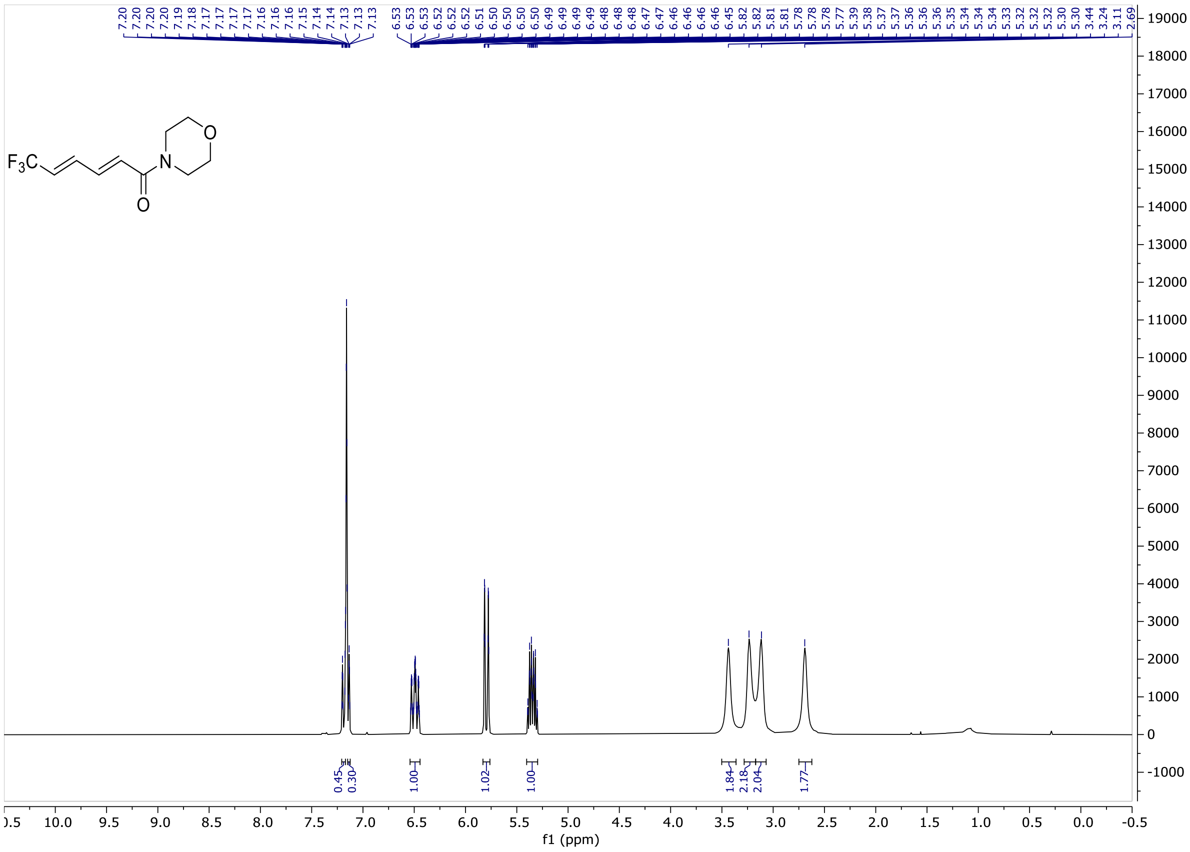

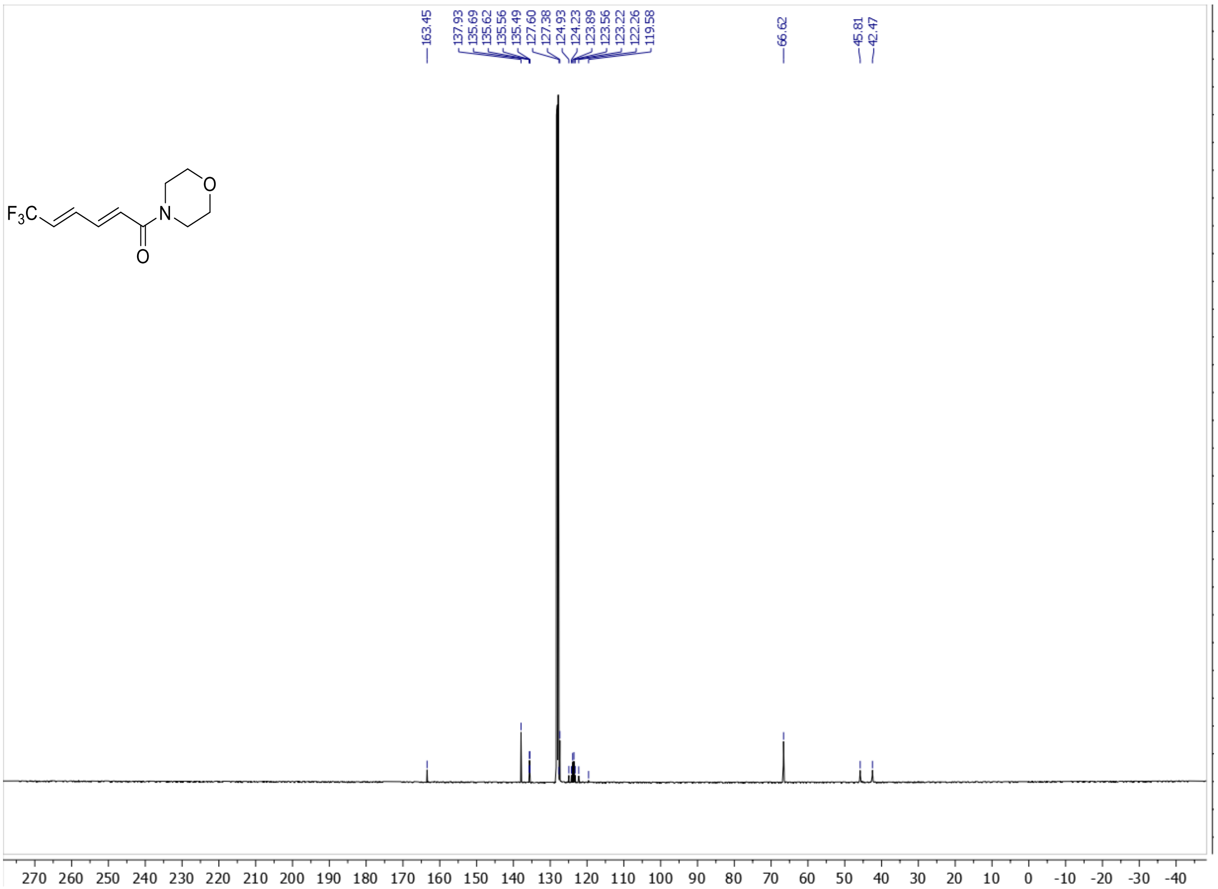

^{19}F NMR (282 MHz, C_6D_6) of compound **23**

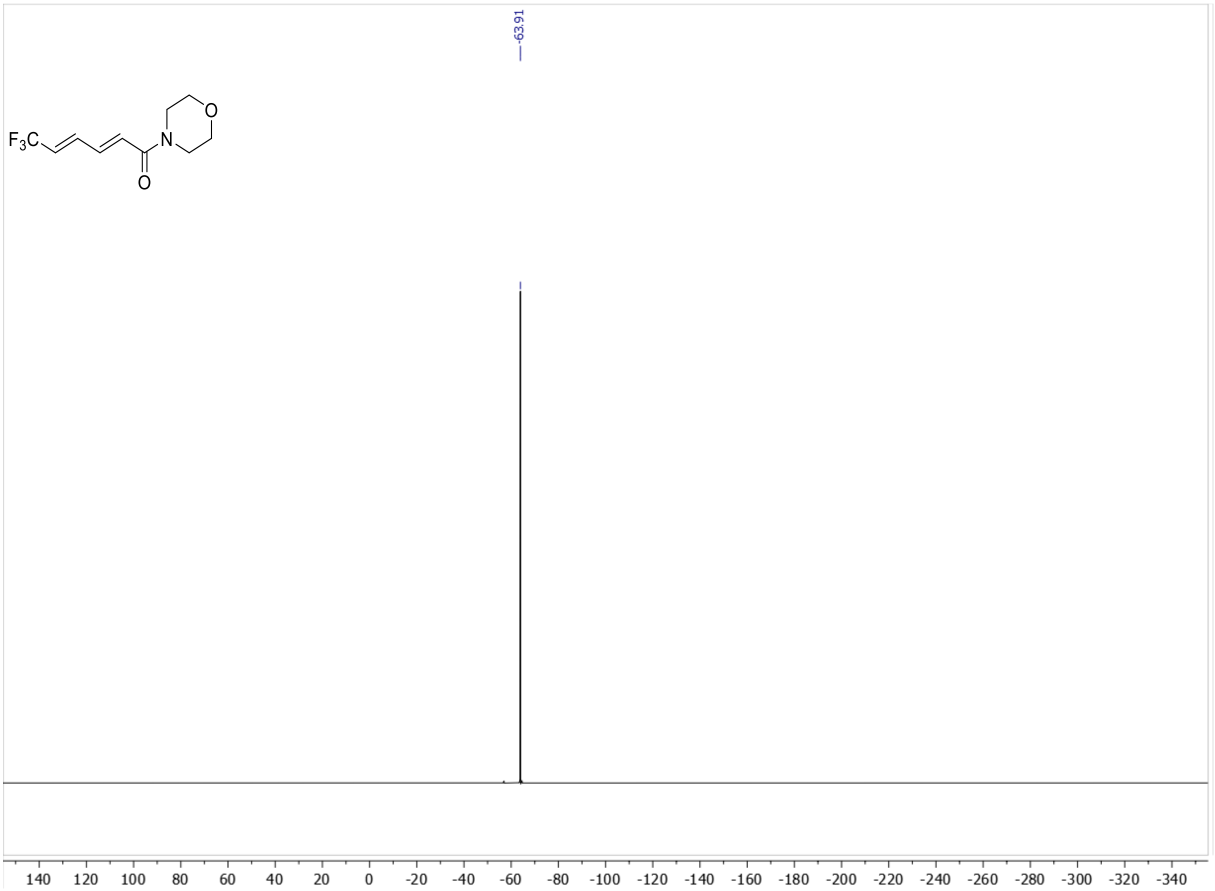

^{1}H NMR (400 MHz, $CDCl_3$; top) and ^{13}C NMR (101 MHz, $CDCl_3$; bottom) of compound **24a**

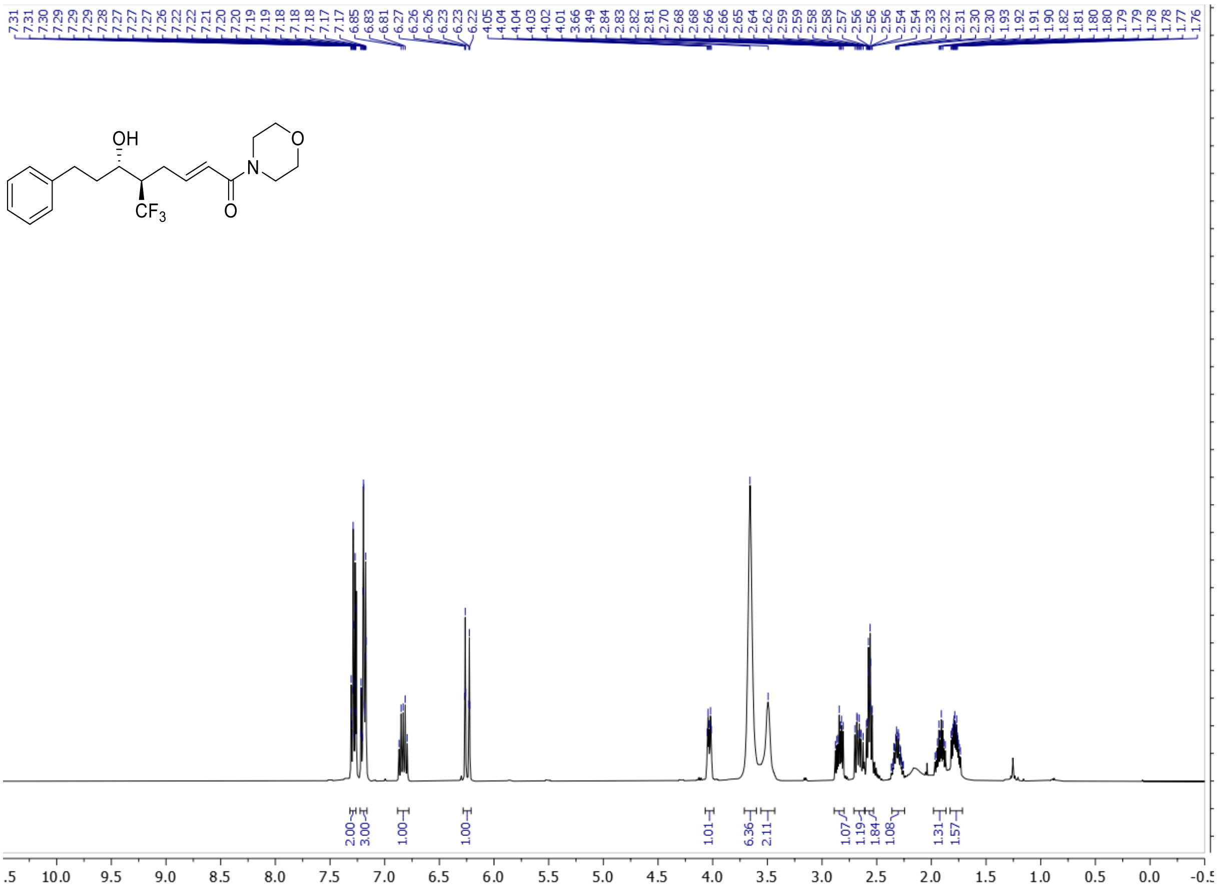

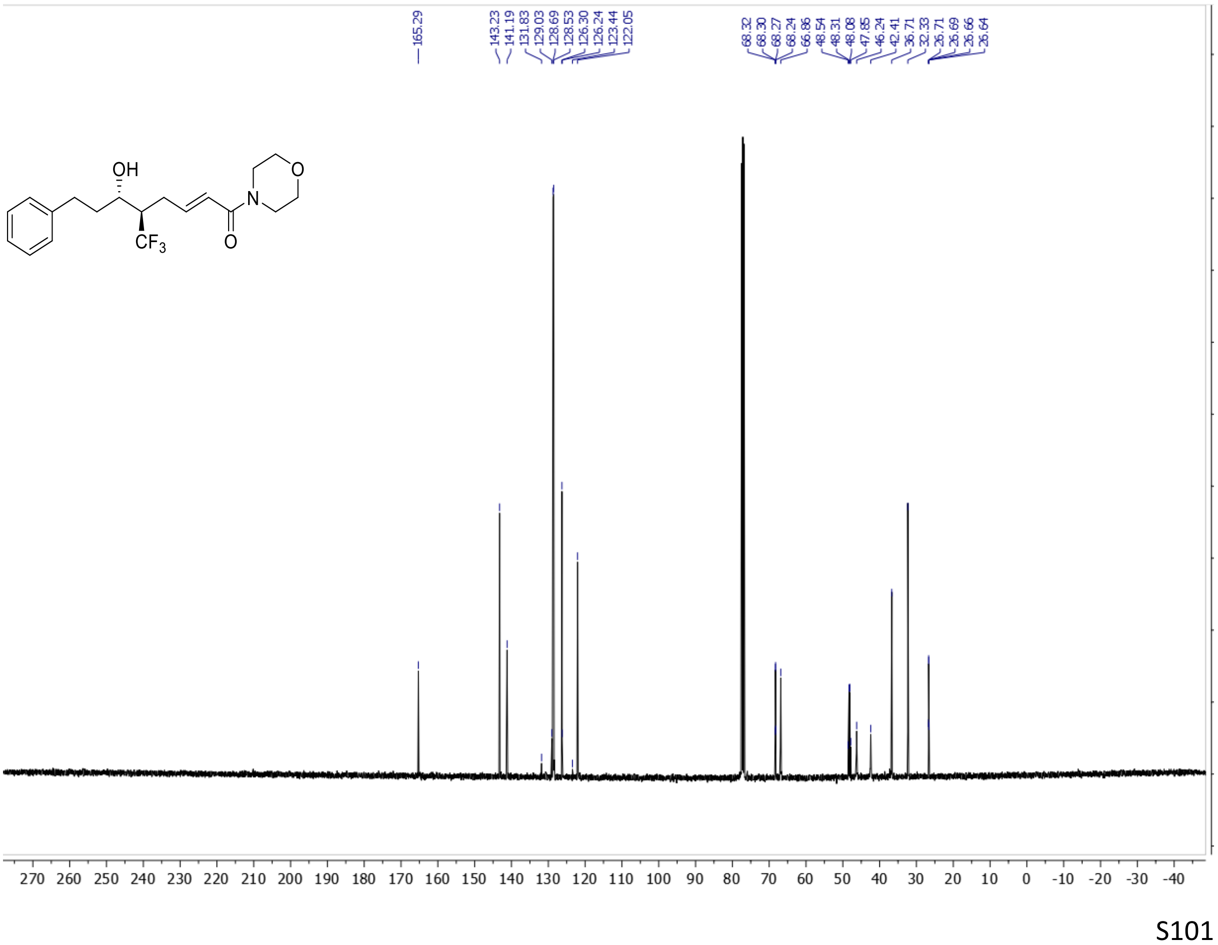

^{19}F NMR (282 MHz, $CDCl_3$) of compound **24a**

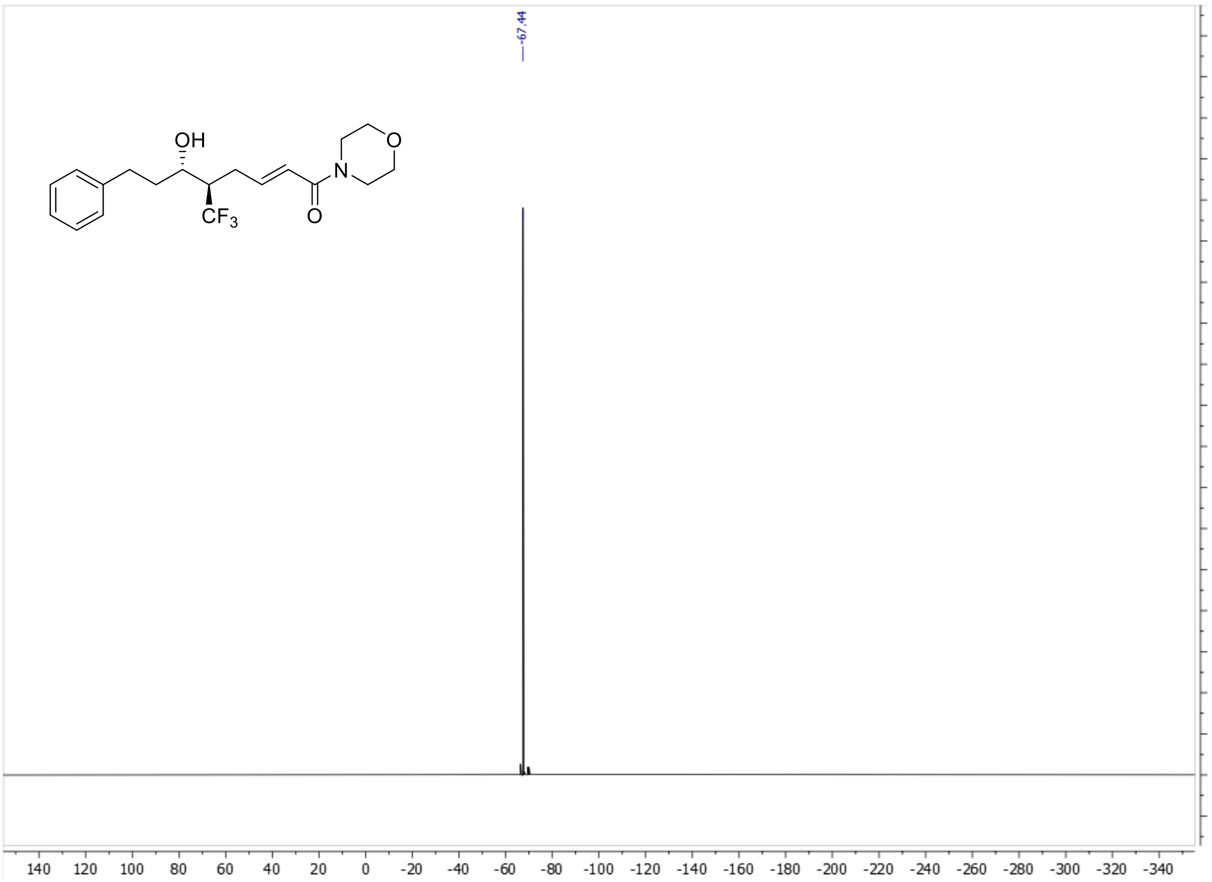

^{1}H NMR (400 MHz, $CDCl_3$; top) and ^{13}C NMR (101 MHz, $CDCl_3$; bottom) of compound **24b**

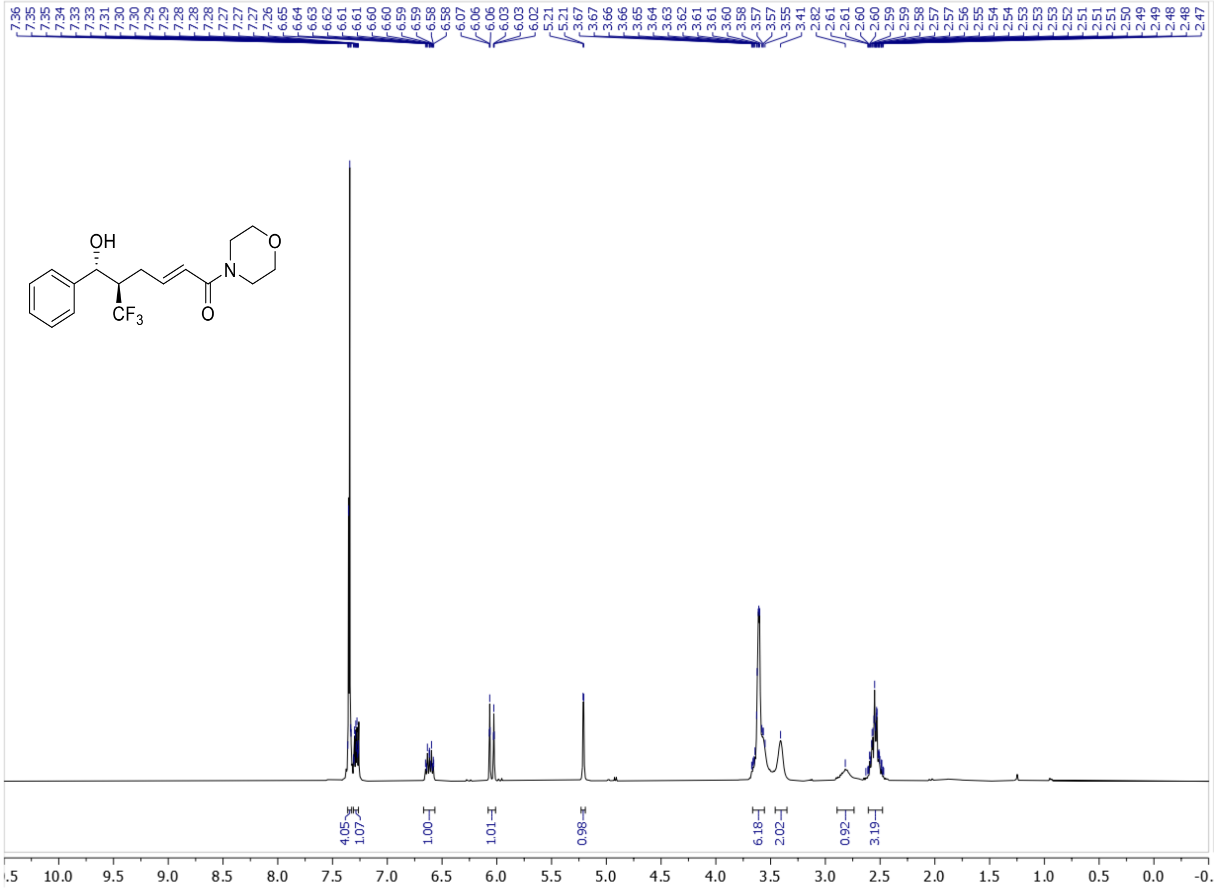

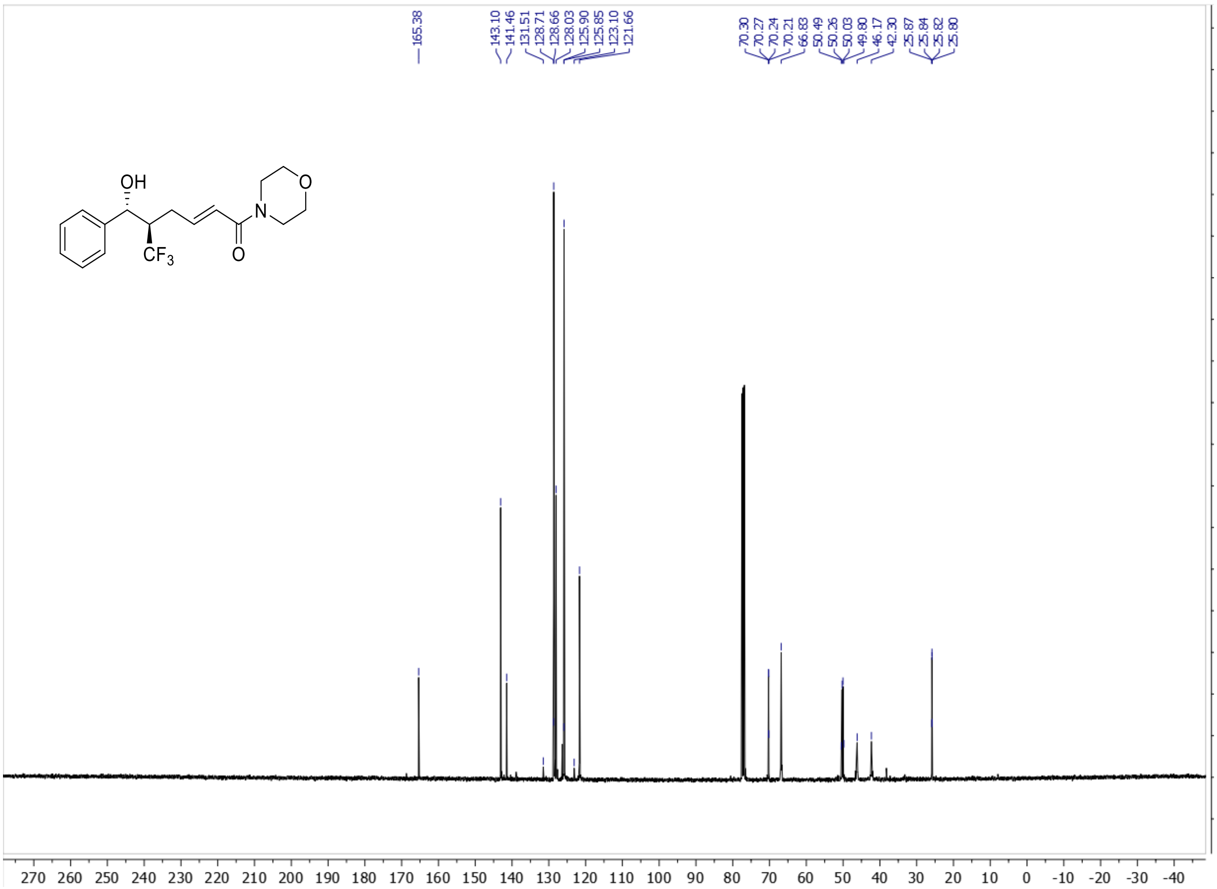

^{19}F NMR (282 MHz, $CDCl_3$) of compound **24b**

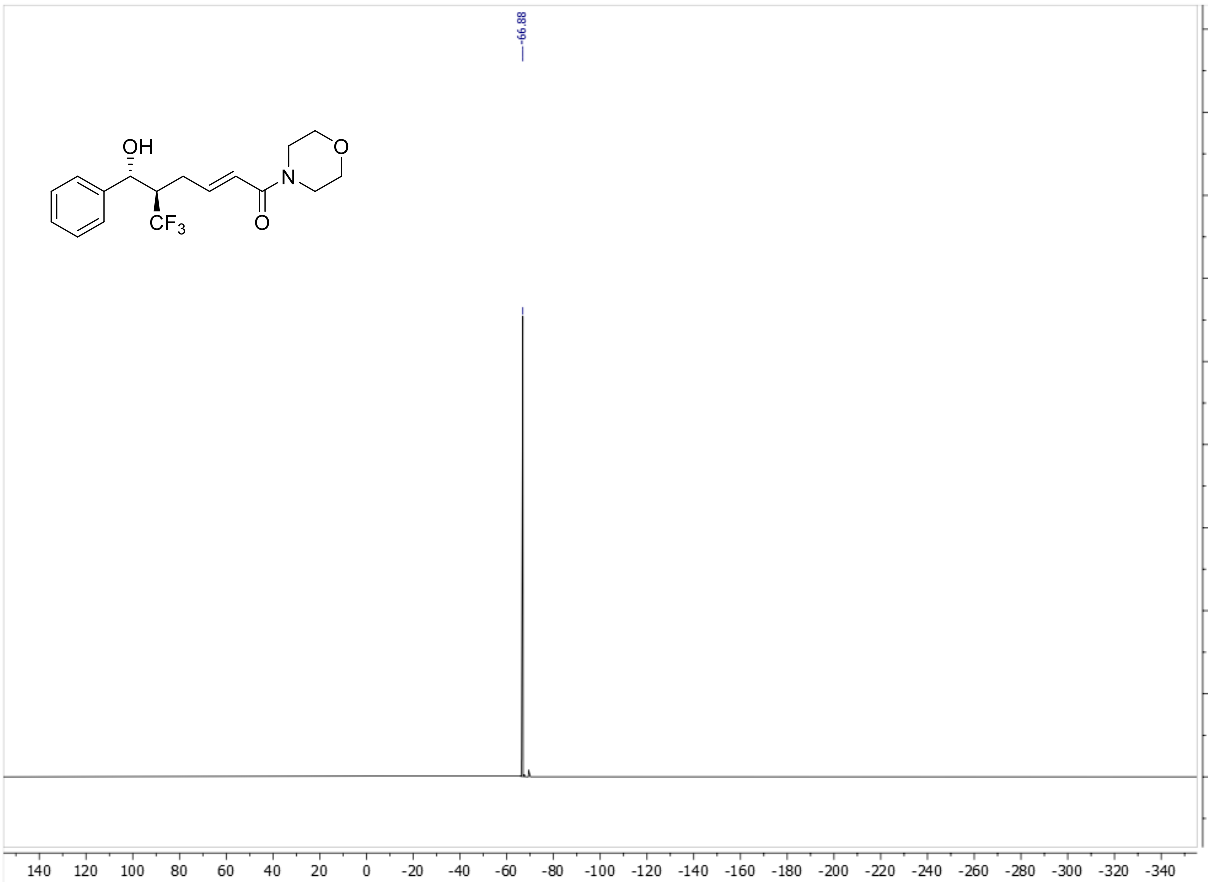

^{1}H NMR (400 MHz, $CDCl_3$; top) and ^{13}C NMR (101 MHz, $CDCl_3$; bottom) of compound **26a**

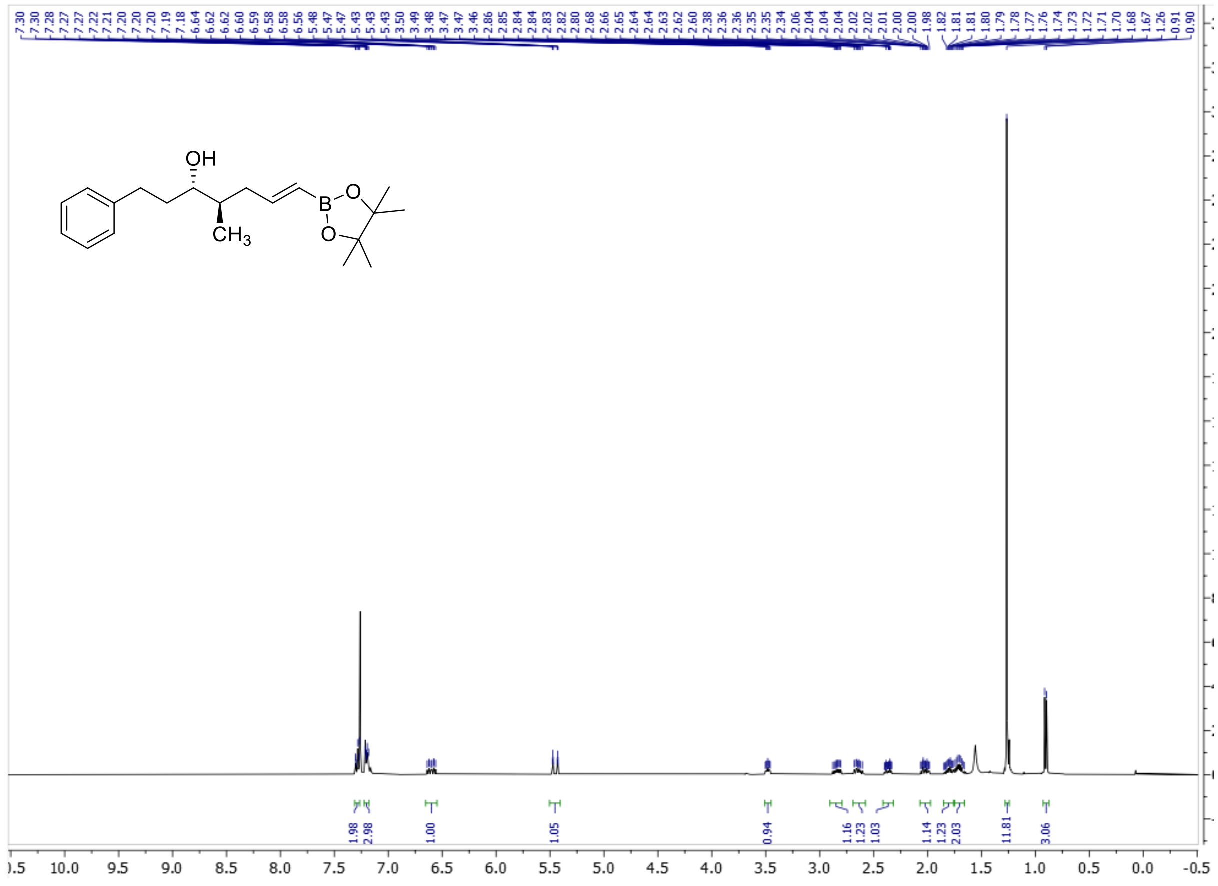

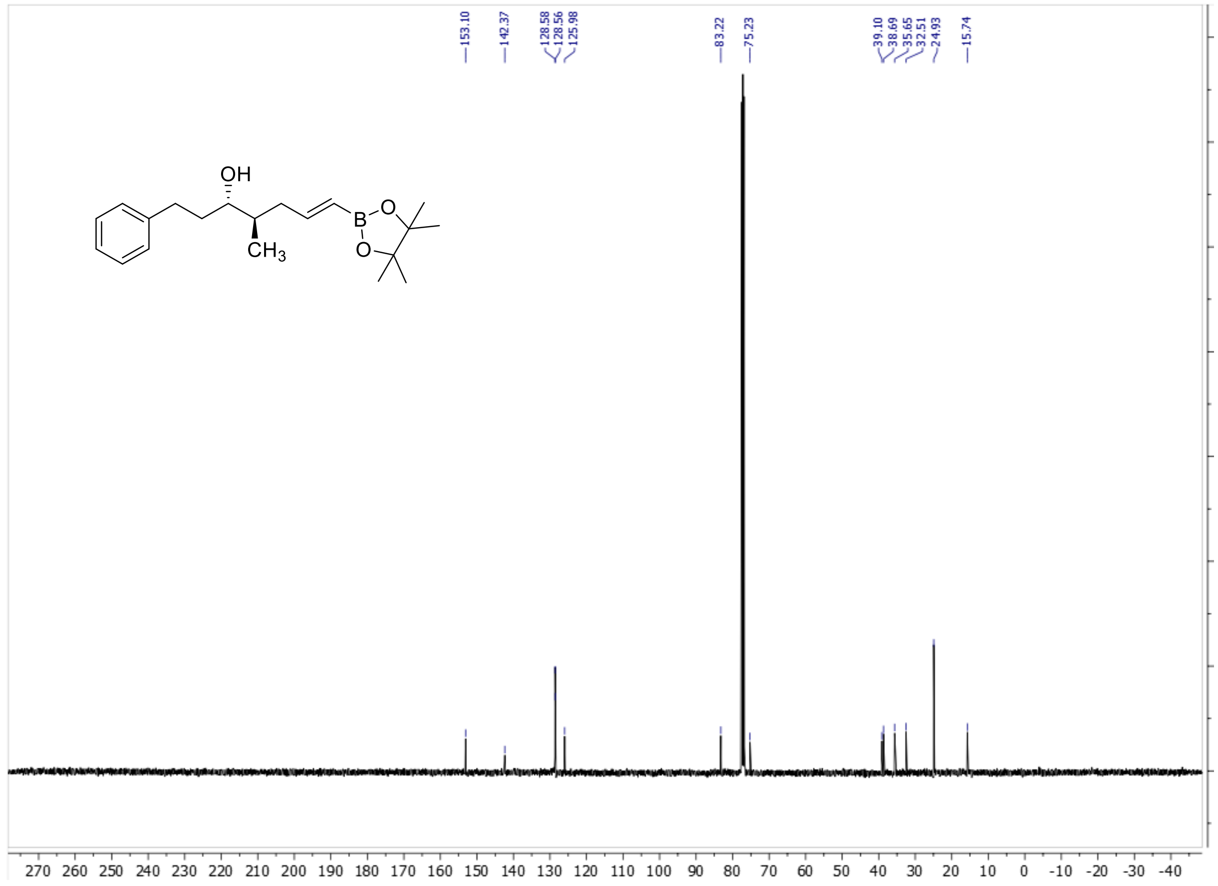

^{1}H NMR (400 MHz, $CDCl_3$; top) and ^{13}C NMR (101 MHz, $CDCl_3$; bottom) of compound **26b**

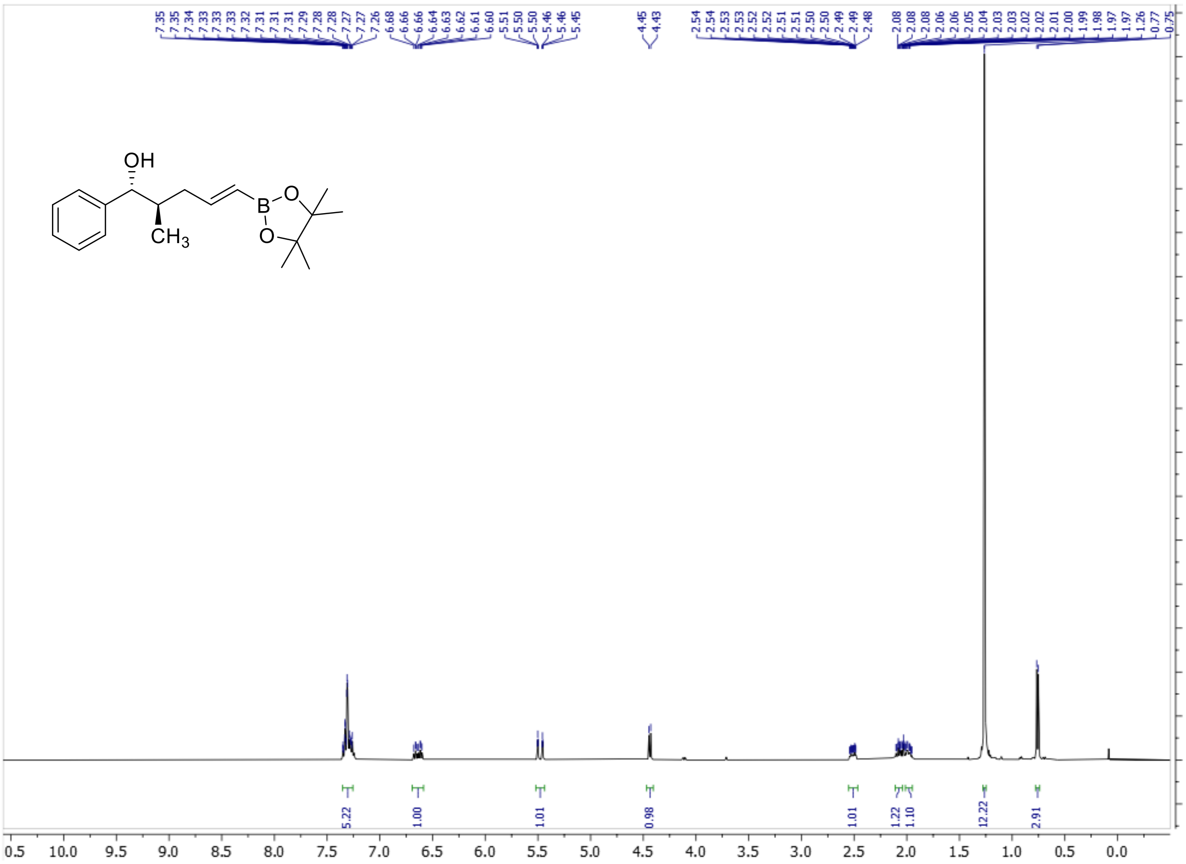

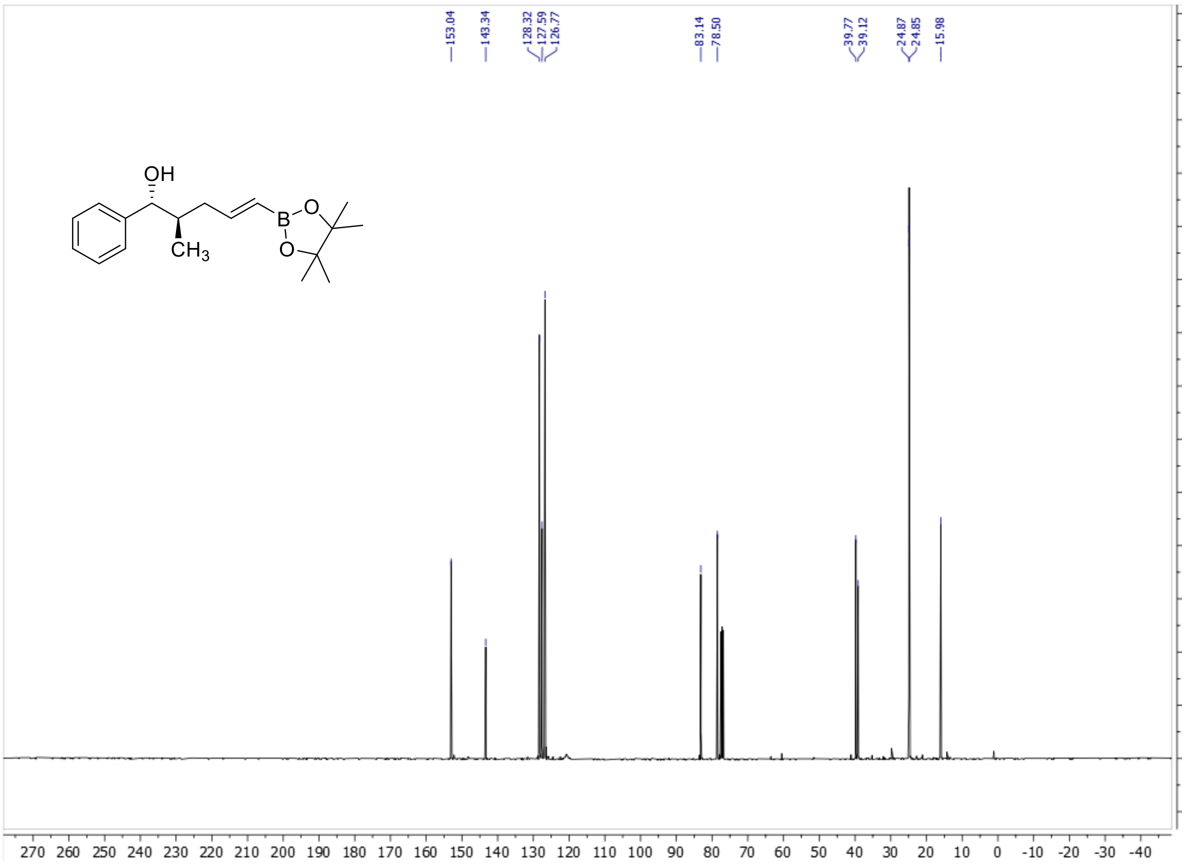

^{1}H NMR (400 MHz, $CDCl_3$; top) and ^{13}C NMR (101 MHz, $CDCl_3$; bottom) of compound **S6**

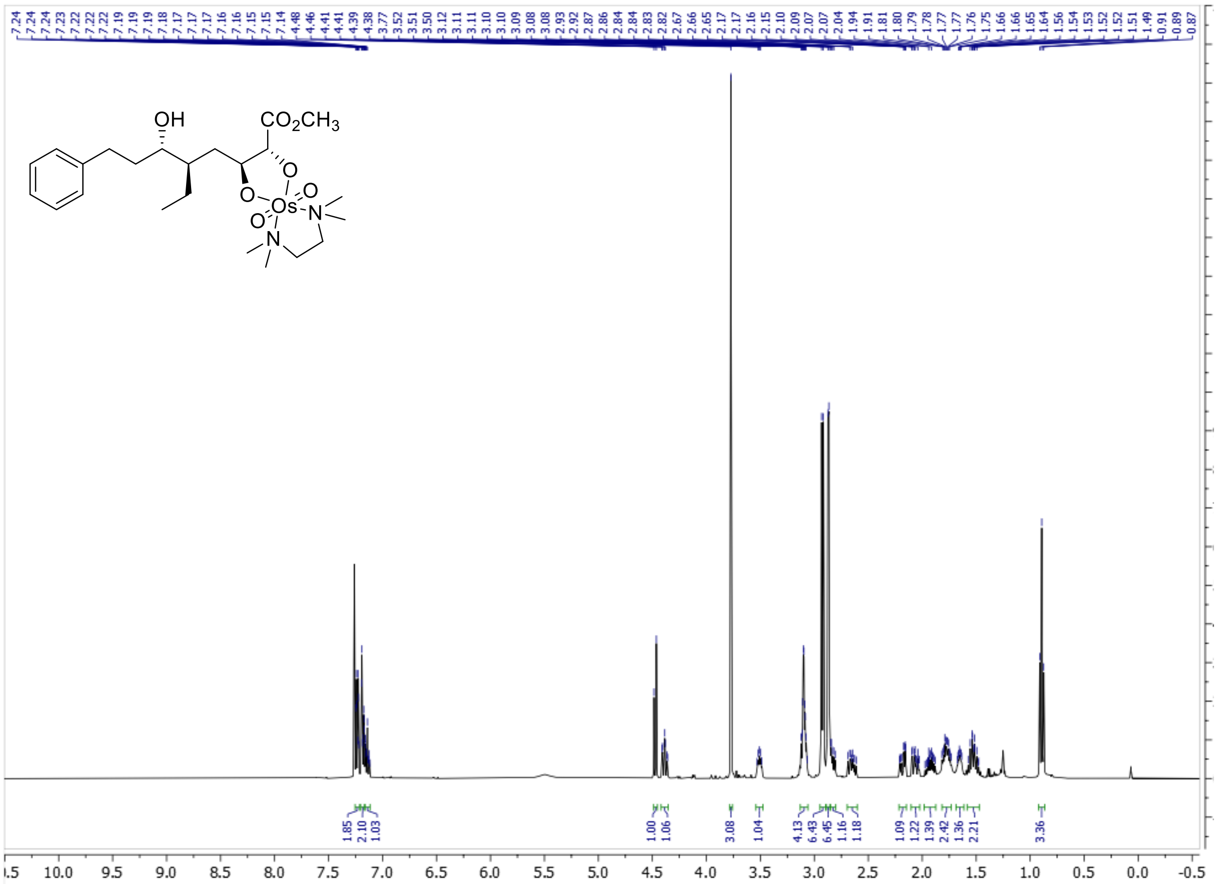

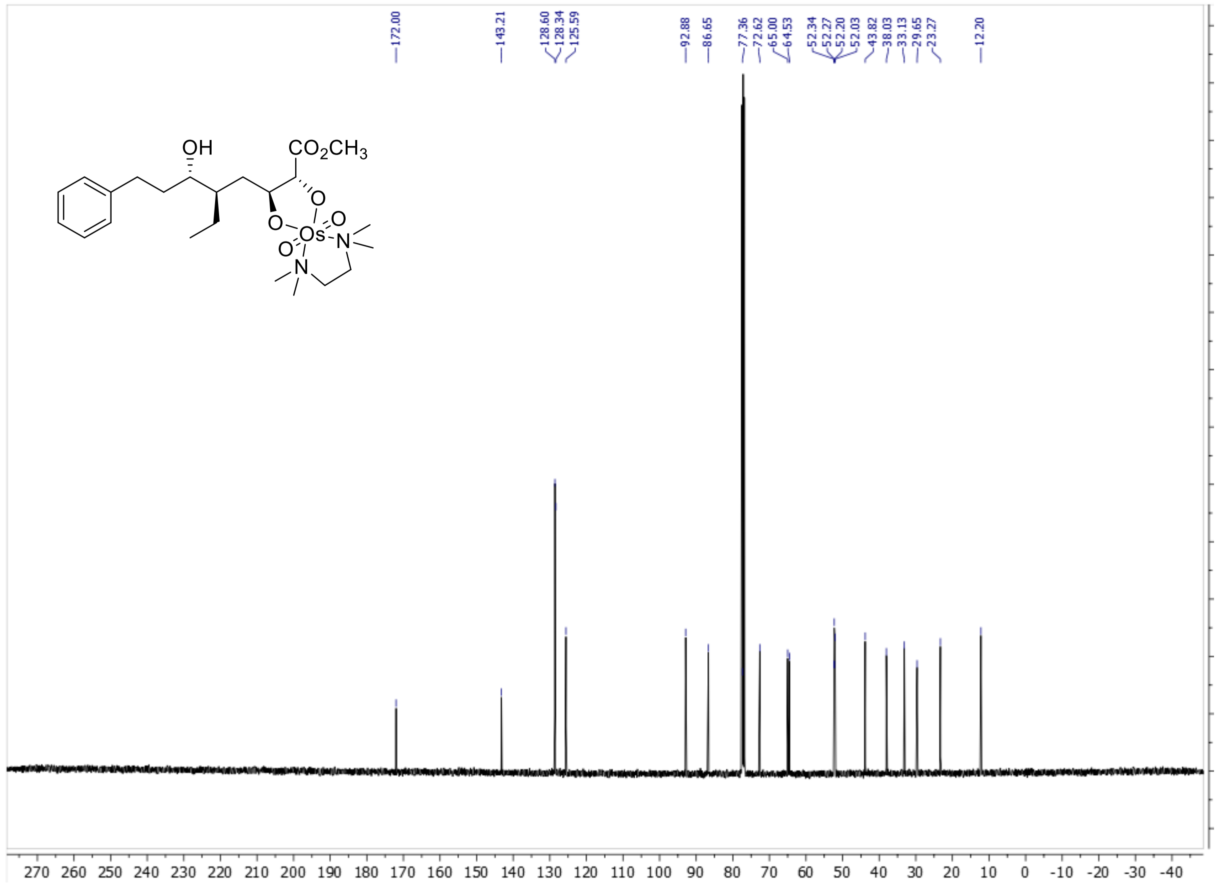

References

1. Davies, T. Q.; Murphy, J. J.; Dousset, M.; Fürstner, A., Nickel-Catalyzed Enantioselective Synthesis of Pre-Differentiated Homoallylic syn- or anti-1,2-Diols from Aldehydes and Dienol Ethers. *J. Am. Chem. Soc.* **2021,** *143* (34), 13489-13494.
2. Nattmann, L.; Cornella, J., Ni(4-tBustb)3: A Robust 16-Electron Ni(0) Olefin Complex for Catalysis. *Organometallics* **2020,** *39* (18), 3295-3300.
3. Nattmann, L.; Saeb, R.; Nöthling, N.; Cornella, J., An air-stable binary Ni(0)–olefin catalyst. *Nat. Catal.* **2020,** *3* (1), 6-13.
4. Kimura, M.; Ezoe, A.; Mori, M.; Iwata, K.; Tamaru, Y., Regio- and Stereoselective Nickel-Catalyzed Homoallylation of Aldehydes with 1,3-Dienes. *J. Am. Chem. Soc.* **2006,** *128* (26), 8559-8568.
5. Ogoshi, S.; Tonomori, K.; Oka, M.; Kurosawa, H., Reversible Carbon−Carbon Bond Formation between 1,3-Dienes and Aldehyde or Ketone on Nickel(0). *J. Am. Chem. Soc.* **2006,** *128* (21), 7077-7086.
6. Yang, Y.; Zhu, S.-F.; Duan, H.-F.; Zhou, C.-Y.; Wang, L.-X.; Zhou, Q.-L., Asymmetric Reductive Coupling of Dienes and Aldehydes Catalyzed by Nickel Complexes of Spiro Phosphoramidites: Highly Enantioselective Synthesis of Chiral Bishomoallylic Alcohols. *J. Am. Chem. Soc.* **2007,** *129* (8), 2248-2249.
7. Neel, A. J.; Hilton, M. J.; Sigman, M. S.; Toste, F. D., Exploiting Non-Covalent π-Interactions for Catalyst Design. *Nature* **2017,** *543*, 637.
8. Meyer, E. A.; Castellano, R. K.; Diederich, F., Interactions with Aromatic Rings in Chemical and Biological Recognition. *Angew. Chem. Int. Ed.* **2003,** *42*, 1210.
9. Liang, T.; Zhang, W.; Chen, T.-Y.; Nguyen, K. D.; Krische, M. J., Ruthenium Catalyzed Diastereo- and Enantioselective Coupling of Propargyl Ethers with Alcohols: Siloxy-Crotylation via Hydride Shift Enabled Conversion of Alkynes to π-Allyls. *J. Am. Chem. Soc.* **2015,** *137* (40), 13066-13071.
10. Paquette, L. A.; Chang, J.; Liu, Z., Synthetic Studies Aimed at (−)-Cochleamycin A. Evaluation of Late-Stage Macrocyclization Alternatives. *J. Org. Chem.* **2004,** *69* (19), 6441-6448.
11. Davies, S. G.; Haggitt, J. R.; Ichihara, O.; Kelly, R. J.; Leech, M. A.; Price Mortimer, A. J.; Roberts, P. M.; Smith, A. D., Asymmetric total synthesis of sperabillins B and D via lithium amide conjugate addition. *Org. Biom. Chem.* **2004,** *2* (18), 2630-2649.
12. Czyzewski, M.; Sellars, J. D.; Guliashvili, T.; Tibbelin, J.; Johnstone, L.; Bower, J.; Box, M.; Davies, R. D. M.; Ottosson, H.; Steel, P. G., The first intramolecular silene Diels–Alder reactions. *Chem. Commun.* **2014,** *50* (22), 2919-2921.
13. Das, S.; Goswami, R. K., Total synthesis of marine natural products separacenes A and B. *Org. Biom. Chem.* **2017,** *15* (22), 4842-4850.
14. Guo, Y.; Kootstra, J.; Harutyunyan, S. R., Catalytic Regio- and Enantioselective Alkylation of Conjugated Dienyl Amides. *Angew. Chem. Int. Ed.* **2018,** *57* (41), 13547-13550.
15. The preparation follows a literature procedure for analogous esters, see: Yoshimoto, R.; Usuki, Y.; Satoh, T., Rhodium(III)-catalyzed β-Arylation and -Alkenylation of α-Trifluoromethylacrylic Acid. Chem. Lett. 2019, 48 (5), 461-464.
16. Coombs, J. R.; Zhang, L.; Morken, J. P., Synthesis of Vinyl Boronates from Aldehydes by a Practical Boron–Wittig Reaction. *Org. Lett.* **2015,** *17* (7), 1708-1711.
17. Burns, A. S.; Dooley, C.; Carlson, P. R.; Ziller, J. W.; Rychnovsky, S. D., Relative and Absolute Structure Assignments of Alkenes Using Crystalline Osmate Derivatives for X-ray Analysis. *Org. Lett.* **2019,** *21* (24), 10125-10129.
